# Taxonomic revision of Chenopodiaceae in Himalaya and Tibet

**DOI:** 10.3897/phytokeys.116.27301

**Published:** 2019-01-31

**Authors:** Alexander P. Sukhorukov, Pei-Liang Liu, Maria Kushunina

**Affiliations:** 1 Department of Higher Plants, Biological Faculty, Lomonosov Moscow State University, 119234, Moscow, Russia; 2 College of Life Sciences, Northwest University, 710069, Xi’an, Shaanxi, China; 3 Department of Plant Physiology, Biological Faculty, Lomonosov Moscow State University, 119234, Moscow, Russia

**Keywords:** Bhutan, China, distribution, ecology, India, Nepal, new species, typification

## Abstract

The composition of many Chenopodiaceae genera in different parts of Himalaya and Tibet has been insufficiently known or contradictory. A revision of the family in Himalaya including Bhutan, Nepal, parts of India (Himachal Pradesh, Jammu and Kashmir, Sikkim and Uttarakhand) and Tibet (Xizang, China) is presented for the first time. Altogether, 57 species from 20 genera are reported, including three species new to science (*Agriophyllumtibeticum*, *Salsolaaustrotibetica* and *Salsolahartmannii*). *Atriplexcentralasiatica*, *Corispermumdutreuilii* and *Salsolamonoptera* are identified as new records for India and *Chenopodiumpamiricum* is recorded in China for the first time. *Dysphaniaambrosioides* and *Sympegmaregelii* are recorded for Xizang. The generic and species keys, species distributions (including maps) and taxonomic notes are provided. We indicate for the first time that the presence of short yellow hairs is the remarkable morphological characteristic of the genus *Grubovia*. Evident heterocarpy and heterospermy is found in *Dysphania* for the first time (*Dysphaniatibetica*). *Agriophyllumpungens*, *Atriplexcrassifolia*, *Atriplexlaciniata*, *Atriplexsagittata*, *Axyrisamaranthoides*, *Axyrishybrida*, *Bassiaindica*, *Corispermumkorovinii*, *Dysphaniaschraderiana* (=*Chenopodiumfoetidum* auct.), *Halocharisviolacea* and *Suaedamicrosperma* are excluded from the species list. *Neobotrydiumcorniculatum* is synonymised with *Dysphaniakitiae*, *Neobotrydiumlongii* with *Dysphaniahimalaica* and *Neobotrydiumornithopodum* seems to be conspecific with *Dysphanianepalensis*. *Corispermumladakhianum* is a new synonym of *Corispermumtibeticum*. *Amaranthusdiandrus* is added to the synonyms of *Acroglochinpersicarioides*, and *Bassiafiedleri*, previously considered as conspecific with *Gruboviadasyphylla*, is added to the synonymy of *Bassiascoparia*. Lectotypes of *Anabasisglomerata* (≡*Halogetonglomeratus*), *Halogetontibeticus* (=*Halogetonglomeratus*), *Amaranthusdiandrus* (=*Acroglochinpersicarioides*), *Chenopodiumtibeticum* (≡*Dysphaniatibetica*), *Corispermumdutreuilii*, *Corispermumfalcatum*, *Corispermumlhasaense*, Corispermumpamiricumvar.pilocarpum (=*Corispermumgelidum*, syn. nov.), *Corispermumtibeticum*, *Kochiaindica*(≡*Bassiaindica*), *Kochiaodontoptera* (≡*Bassiaodontoptera*) and *Salsolamonoptera* are selected. Out of 53 native elements, 42 are restricted in their distribution to Himalaya and Tibet at altitudes 2000–4500 m above sea level. The greatest taxonomic diversity of the Chenopodiaceae is represented in Jammu and Kashmir (India) and Xizang (China) with a continuous decrease in the number of species southwards.

## Introduction

The family Chenopodiaceae is one of the most difficult groups of flowering plants in terms of its taxonomy and diagnostics. In the last two decades, our knowledge of the relationships in the family, its placement within the order Caryophyllales and the physiological or anatomical peculiarities of its species have greatly increased. However, there have only been a limited number of taxonomic revisions of the family based on recent taxonomy and critical analysis of collections.

### Taxonomy and generic delimitation

The Chenopodiaceae is a part of the large Chenopodiaceae-Amaranthaceae clade within the core order of Caryophyllales and it is a sister group to the Amaranthaceae family (Cuénoud et al. 2002, Kadereit et al. 2003, Brockington et al. 2009). Although all morphological characters seem to overlap in Chenopodiaceae and Amaranthaceae s. str., the familial status of the Chenopodiaceae is accepted in recent taxonomic treatments (e.g. Sukhorukov 2014, Hernández-Ledesma et al. 2015). The carpological differences that have been discovered (Sukhorukov et al. 2015a) have filled the gaps in the character set of both Chenopodiaceae and Amaranthaceae s. str., but they are not strongly specific at the familial level except the horizontal embryo position which evolved in many Chenopodiaceae.

When separated from Amaranthaceae, the Chenopodiaceae includes ~100 genera and 1600 species that are mostly distributed in the deserts and steppes in the temperate regions of the world (Sukhorukov 2014). During the last fifteen years, the generic composition of many groups in the family has undergone extensive changes following molecular phylogenetic revisions, with the splitting or merging of many genera. *Chenopodium* was found to be polyphyletic and its members occupy different phylogenetic positions within Chenopodioideae (Fuentes et al. 2012; Sukhorukov et al. 2018). In the previous broader sense, *Salsola* appeared to be strongly polyphyletic (Akhani et al. 2007), but in contrast, the formerly accepted genus *Kochia* is now merged with *Bassia* (Kadereit & Freitag, 2011). The monophyly of many other traditional genera, e.g. *Agriophyllum* (Kadereit et al., 2003), *Suaeda* (Schütze et al., 2003), *Atriplex* except *Halimione* (Kadereit et al., 2010), *Corispermum* (Xue & Zhang, 2011) and *Dysphania*, including *Neobotrydium* and *Ambrina* (Uotila et al. in prep.) is confirmed.

Subsequently, the generic status of some groups in Tibet and Himalaya has been changed compared with previous taxonomy accepted in many floristic works including comprehensive treatments of the family in adjacent territories like “Flora Iranica” (Rechinger 1997), “Flora of Pakistan” (Ali and Qaiser 2001), as well as “Flora of China” (Wu and Raven 2003) that emcompasses a part of the territory under study. Of all the known Chenopodiaceae species in the study area, only the position of *Haloxylonthomsonii* is still pending within the tribe Salsoleae and its placement with tree-like species within the Saharo-Arabian and Irano-Turanian genus *Haloxylon* may be incorrect due to differences in life history and stem anatomy (Sukhorukov, unpubl.).

Progress towards disentangling the generic composition of Chenopodiaceae in Himalaya and Tibet began with the critical revisions and additions to *Axyris* (Sukhorukov, 2011), *Dysphania* (Sukhorukov 2012, 2014, Uotila 2013, Sukhorukov et al. 2015b, Nobis et al. 2015, 2017) and part of *Chenopodium* s. str. (Sukhorukov and Kushunina 2014, 2015).

### Study premise

The most recent taxonomic revision of the Chenopodiaceae was performed for Nepal (Sukhorukov and Kushunina 2014), but the species composition of some genera and their distributions are still insufficiently known in other parts of the entire Himalayas and Tibet. The latest Chenopodiaceae treatment for India (Paul 2012) contained many taxa that are, in fact, distributed only in Central Asia (e.g. *Atriplexcrassifolia*, *Axyrisamaranthoides*, *Chenopodiastrumhybridum* and *Suaedamicrosperma*), Eastern Europe/North Kazakhstan (*Atriplexsagittata*) or the Mediterranean (*Atriplexrosea*). Taxonomic novelties to the chenopodiaceous flora of China were provided by Zhu and Sanderson (2017) and some new species (i.e. *Neobotrydiumcorniculatum*, *N.longii*) were also reported from Xizang. All these taxa reported from India or China are discussed in our article.

The present revision is a part of the treatment of the family in the Old World. Until now, the Chenopodiaceae had been revised for the flora of European Russia (Sukhorukov 2014), Iraq (Sukhorukov et al. 2016) and historical Palestine (Danin et al. in press). In addition to Himalaya and Tibet, further contributions are planned for all of Africa, Russia and Uzbekistan.

## Materials and methods

### Choosing the study territory

**Figure 1.**
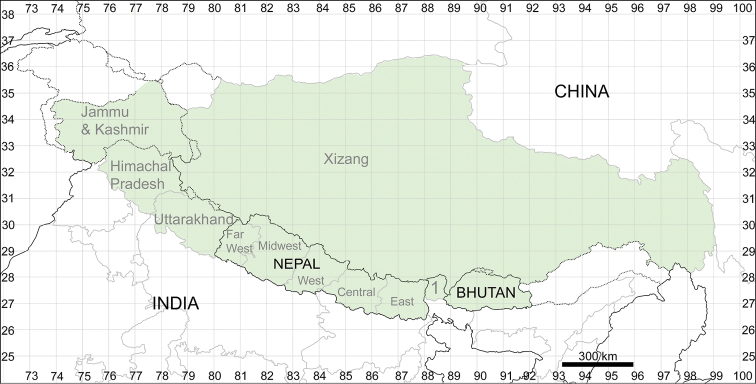
The territory included in the present investigation (highlighted in green). Abbreviations: 1 – Sikkim.

Geographically, the Himalayan Mountains and the Tibetan Plateau (sometimes called Pan-Himalaya) encompass a large territory bordered by the Karakoram Range to the north, the Indian plate to the west, the Central China plain to the east and Mainland Southeast Asia to the south. The territory included in the present investigation (Fig. 1) covers most parts of Himalaya and the Tibetan Plateau within the following countries: Bhutan, Nepal, parts of China (Xizang or the Tibet Autonomous Region) and India (states of Uttarakhand, Himachal Pradesh, Sikkim and Jammu and Kashmir, excluding the politically disputed territory in North Jammu and Kashmir with a poorly investigated flora that is probably rich in Central Asian species). Arunachal Pradesh (India) is not included in this treatment due to an absence of specimens seen. This treatment is a major contribution to Chenopodiaceae under the project “Flora of Pan-Himalaya”.

### Materials investigated

The field investigations were conducted by Alexander Sukhorukov in different provinces of Nepal in 2005 and 2008–2015 and in Dehradun and its surroundings in 2018 (Uttarakhand, India). The main collection is in MW with duplicates in B, BM, BR, E, G, H, K, M and W. The revision of the herbarium material was undertaken by the first author (AS) at B, BM, BR, BSD, DD, E, G, H, HUJ, K, KATH, L (including U and WAG), LE, LY, M, MHA, MSB, MW, P, PE, PRA, SHI, TO, TUCH, W, WU, WUK, XIA and XJBI (herbarium abbreviations according to Thiers 2018+) and the *Axyris* and many *Dysphania* specimens were revised by the first author in CAL in 2011. The Chinese Virtual Herbarium (CVH, http://www.cvh.ac.cn/) and the herbarium database of the Kunming Institute of Botany of the Chinese Academy of Sciences (Kingdonia, http://db.kun.ac.cn/) were used as citations for some specimens kept in HNWP, NAS and KUN if their identification were possible using the images. The great number of examined specimens was mostly provided by the following collectors or collection teams.

**Adolph, Hermann and Robert Schlagintweit.** Expeditions to Himalaya 1854–1857 including West/Central Himalaya, Tibet and adjacent territories (Büchler 1990). Many interesting facts about the lives of these brothers, their relationships with other botanists and the destiny of their herbarium can be found in the book by von Brescius et al. (2015). The examined collections are available from the year 1856 in the BM, E, G and LE herbaria.

**Thomas T. Thomson.** Expeditions to West Himalaya, Tibet and then to Sikkim (Woodward 1898). Chenopodiaceae collections are available from 1845 and 1847–1848, mostly from North India (Jammu and Kashmir, Himachal Pradesh and Uttarakhand). The sheets often labelled as “collected in Thibet occ.” [West Tibet] were probably collected in West Himalaya (former British India), but without precise locations, therefore these records could not be mapped.

**Walter Koelz.** Expeditions to North India (Jammu and Kashmir). Although the main goal of the expeditions was ornithology, he gathered many plant species including Chenopodiaceae. The collections, kept at the herbarium of Michigan University (n.v.), are also stored in herb. P (1930; Himachal Pradesh) and dispersed in E, G, H, K, L, LE and W (1931, 1933; Jammu and Kashmir).

**Francis Ludlow** (often collected together with **George Sherriff** and other scientists). The Tibetan and North Himalayan collections are available from 1928, 1939, 1940–1943 in BM and E with some duplicates in G and LE. The explorations of F. Ludlow and G. Sherriff are described by Fletcher (1975).

**Andrew John Charles Grierson and David Geoffrey Long.** Their joint collections of Chenopodiaceae are from Bhutan (1979, 1982, 1984) and are kept in Edinburgh (E) with duplicates in Kew (K). Additionally, D.G. Long collected some material in Nepal (1991–1992, kept in E).

**Hans Hartmann.** Worked in Jammu and Kashmir (India) and adjacent territories from 1976 until 1997; the Chenopodiaceae collections are available from 1976, 1979, 1992, 1995 and 1997 in G and MSB.

**Brij Mohan Wadhwa.** Had special interest in the Chenopodiaceae of Indian Himalaya. His collections from Jammu and Kashmir (1976 and 1986) and Himachal Pradesh (1974) are mostly kept in BSD.

**Upendra Chandra Bhattacharyya.** Collected many Chenopodiaceae taxa in India: Jammu and Kashmir (1970, 1980), Himachal Pradesh (1970–1972) and Uttarakhand (1961, 1970, 1983). His collections are deposited in BSD.

**Georg and Sabine Miehe.** Mostly worked in Tibet (Xizang, China) from 1977 to 2005. The main collections are in Marburg (“High Asia Project Herbarium G. & S. Miehe”, Faculty of Geography, University of Marburg, Deutschhausstr. 10, D-35032 Marburg, Germany). The herbarium does not have an acronym. Instead, “herb. Miehe” was used when citing their specimens in this paper.

**Bernhard Dickoré.** Worked in Tibet (Xizang) in 1989, 1994 and 1998. The main collections are in Munich (MSB with some duplicates in K and KAS). Some specimens were not seen by the first author (A.S.) during his visit to Munich in May 2016.

**Leoš Klimeš** (Leosh Klimesh). Collections available from Ladakh, North India, collected in 1989 (MSB) and 1999–2007 (PRA). He has been missing in Ladakh since September 2007. His collections in PRA are the most complete recent specimens of Chenopodiaceae from Ladakh. However, not all specimens are present or mounted in PRA (pers. comm. Jan Štepanek).

**Tibet Chinese Herbal Medicine Survey Team.** Collected in Lhasa and Xigazê (Rikaze) Prefectures (Xizang) in 1972. More than 6000 collections were made and the specimens were deposited in PE and HNWP. Collectors on this team included Wang Jin-Ting, Lang Kai-Yong, Ma Cheng-Gong and Bao Xian-Cheng (Wang et al. 2004).

**Qinghai-Tibet Complex Expedition Team**, abbreviated **Qinghai-Tibet Team.** Collected throughout Xizang in 1973–1976. The team also consisted of several subdivisions, such as the “Vegetation Group”, “Meadow Group” and “Additional Group”. In total, nearly 40000 collections on approximately 150000 sheets were made and these specimens were deposited in PE, KUN and HNWP. Collectors worked in the Team were Wu Su-Gong, Ni Zhi-Cheng, Lang Kai-Yong, Chen Shu-Kun, He Guan-Fu, Cheng Shu-Zhi, Gu Li-Min, Nan Yong, Luosangxi’nao, Xiao Yonghui, Yang Yong-Chang, Huang Rong-Fu, Tao De-Ding, Zang Mu, Yin Wen-Qing, Su Zhi-Yun, Wu Zheng-Yi, Du Qing, Yang Chong-Ren, Guan Kai-Yun, Chen Wei-Lie, Li Bo-Sheng and Wang Jin-Ting (Wang et al. 2004).

The recent collections from Nepal (2005, 2008–2015) were made by **Alexander Sukhorukov** (see above).

### Preparation of the material for carpological studies

The perianth, fruit and seed characters play significant roles in the identification of some large genera, e.g. *Atriplex*, *Chenopodium*, *Corispermum* and *Dysphania* (e.g. Uotila 2001, Sukhorukov 2006, 2007a, 2007b, Sukhorukov and Kushunina 2014) and we used scanning electron microscopy (SEM) to show the important differences in the reproductive traits. The hard tissues (seed coat) do not require any special preparation prior to SEM, but other objects that contain living cells, such as hairs or papillae (on perianth or pericarp), were dehydrated in aqueous ethyl alcohol solutions of increasing concentration, then in alcohol-acetone solutions and pure acetone and then finally critical-point dried. After sputter coating the material with gold-palladium, all SEM observations were made with a JSM–6380 microscope (JEOL Ltd., Japan) in the Laboratory of Electron Microscopy of Moscow State University.

The anatomy of the fruit and seed is important for the diagnostics and taxonomy of many Chenopodiaceae (for more see, e.g. Sukhorukov 2008, 2014, Sukhorukov and Zhang 2013, Sukhorukov et al. 2013, 2014, 2015a). The fruit anatomy appears crucial to the diagnostics of many *Corispermum* and the fruits of almost all members were anatomically investigated in previous studies (Sukhorukov 2007a, 2007b, 2014, Sukhorukov et al. 2014). Here, we present the fruit anatomy of *Corispermumtibeticum* Iljin and add the most important data to the description of the species.

The list of specimens used for SEM is given below.

*Chenopodiumalbum*: Nepal, Karnali zone, Jumla vill., 23 Sep 2010, *A. Sukhorukov 463* (MW);

*C.pamiricum*: Eastern Kazakhstan, 1944, *Goloskokov* s.n. (LE);

*C.ficifolium*: Central Nepal, Narayani zone, Chitwan [distr.], 4 Mar 2008, *A. Sukhorukov* s.n. (MW);

*C.karoi*: India, Jammu & Kashmir, Ladakh, Zanskar Region, S of Padum, 4150 m alt., 29 Aug 2003, *L. Klimeš 3322* (PRA);

*C.bengalense*: Nepal, Dhaulagiri zone, Annapurna conservation area, near Tikhedhunga vill., 1700 m a.s.l., 13 Nov 2008, *A. Sukhorukov* s.n. (MW);

*C.novopokrovskyanum*: India, Jammu & Kashmir, Ladakh, Leh, 3400 m a.s.l., 20 Aug 1979, *Bruhn* s.n. (MSB144211);

*C.atripliciforme*: Afghanistan, Konar, Dewagal Darrah, Aug 1973, *O. Anders 11078* (W06301);

*C.perttii*: Nepal, Karnali zone, Jumla vill., 29 Sep 2014, *A. Sukhorukov 62* (MW);

*Chenopodiastrummurale*: Uzbekistan, Tashkent, 1921, *Ovchinnikov* s.n. (MW);

*C.badachschanicum*: Nepal, Karnali zone, [Mugu distr.] Purana Mugu, Mugu Khola, 13000 ft a.s.l., 23 Aug 1952, *O. Polunin, W.R. Sykes & L.H.J. Williams 3010* (LE);

*Dysphaniaambrosioides*: Nepal, Bagmati zone, [Kathmandu distr.] Godawari, 1400 m a.s.l., 15 Nov 2005, *A. Sukhorukov* s.n. (MW);

*D.himalaica*: 1) China, Xizang, Ngari Prefecture, Gêrzê (Gaize) County, 4250 m a.s.l., Aug 1972, *Li 069* (PE00510702); 2) Gêrzê (Gaize) County, 4500 m a.s.l., 8 Sep 1976, *K.-Y*. *Lang 10153* (PE00511004);

*D.bhutanica*: Bhutan, Thimphu distr., Chapcha, 2200–2400 m a.s.l., 1 Jul 1992, *C. Parker 7270* (E00051982);

*D.kitiae*: China, [Shaanxi prov.] Wuqi County, 13 Sep 1984, *Yang Jinxiang 5115* (WUK);

*D.botrys*: China, Xinjiang, Urumqi, Sep 2014, *A. Sukhorukov* s.n. (MW);

*D.geoffreyi*: China, Xizang, [Doilungdêgên County] Kui Chu, Nyimathang, 29°30'N, 91°02'E, 1 Oct 2001, *G. & S. Miehe 01-141-10* (herb. Miehe); Yunnan prov., Deqin [Diqing] County, Adunzi, 2700 m a.s.l., 26 Sep 1935, Wang Qiwu 70221 (WUK);

*D.tibetica*: India, Jammu & Kashmir, Leh, Lato, 33°40'42"N, 77°43'48"E, 4020 m a.s.l., 5 Sep 2001, *L. Klimeš 1562* (PRA);

*D.neglecta*: Nepal, Karnali zone, border of Jumla & Mugu distr., 1 km from Naurigar vill., 2300 m a.s.l., 26 Sep 2013, *A. Sukhorukov* s.n. (G);

*D.nepalensis*: 1) Nepal, Karnali zone, Jumla vill., 29°17'N, 82°05'E, 2400 m a.s.l., 3 Oct 2010, *A. Sukhorukov 464* (MW); 2) China, Dechin prefecture, W of Dechin near Marbating, 28°33'3"N, 98°47'4"E, 2230 m a.s.l., 16 Sep 1995, *D. Chamberlain & al. 275* (H1684317);

*Corispermumfalcatum*: China, Xizang prov., Gar (Gaer) County, 4250 m a.s.l., 29 Jul 1976, *Qinghai-Tibet Team 76-8613* (PE00540486);

*C.nanum*: China, Xizang prov., Central Plateau, Nagqu to Siling Co (Lake Siling Tso), 31°28'N, 91°0'E, 4580 m a.s.l., lake terraces, 16 Aug 1993, *G. & S. Miehe 9488/03* (MSB147355);

*C.pamiricum*: [Tajikistan] Buchara prov. [Gorno-Badakhshan Autonomous province], Wachan [Vakhan], Pamir river valley near Langar-Gisht fortress, 9500 ft a.s.l., 27 Jul 1901, *Th. Alexeenko 3217* (LE);

C.dutreuiliivar.dutreuilii: India, Jammu & Kashmir, Ladakh, 6 km W of Pangong Tso [Lake], 4250 m a.s.l., 13 Aug 1997, *H. Hartmann 6083* (MSB137929);

C.dutreuiliivar.montanum: China, Xizang, Shiquan [Sênggê] river, alt. 3800 m a.s.l., 3 Aug 1984, *Zhengxi An 1-10092* (XJA);

*C.pseudofalcatum*: China, Xizang, vicinity of Xigazê (Rikaze), 3800 m a.s.l., [no exact date] 1960, *Fu Guo-Xun 789* (PE00934050);

*C.lhasaense*: China, Xizang, Lhasa, the Sera Monastery, Liushahe river, 3760 m a.s.l., 10 Sep 1960, *Fu Guo-Xun 658* (PE00540404);

*C.lepidocarpum*: China, Xizang, Temo, Tsangpo valley, 9500 ft a.s.l., 6 Sep 1938, *F. Ludlow*, *G. Sherriff & G. Taylor 6227* (LE);

*C.gelidum*: [Tajikistan] nr Rang-kul Lake, Zor-Burlyuk valley, 29 Aug 1935, *H. Raikova* s.n. (LE);

*C.tibeticum*: 1) [Afghanistan or Pakistan] Karakoram, Aug 1876, *Falconer* s.n. (K); 2) China [Ngari Pref., Burang County], nr Manasarovar Lake, Langa Tso [Lake], 4680 m a.s.l., 29 Aug 1993, *G. & S. Miehe 9598/03* (MSB147354); 3) India, Ladakh, Pangong Region, 4300 m a.s.l., 10 Sep 2002, *L. Klimeš 10* (PRA);

*Agriophyllumtibeticum*: China, Xizang, Kongbo [Kongpo] County, Temo, Tsangpo valley, 8 Sep 1938, *F. Ludlow & al.6238* (BM);

*Suaedaolufsenii*: India, Jammu & Kashmir, Ladakh, Rupshu Region, Chumur, 4660–4860 m a.s.l., 21 Aug 2001, *L. Klimeš 1382* (PRA).

### Morphological description

Where not stated otherwise, the generic and species descriptions are based on specimens of various origins. The habitats and altitudes relate to populations growing in Himalaya and Tibet.

## Results and discussion

### Ecology and altitudinal gradient of the Himalayan and Tibetan Chenopodiaceae

**Figure 2.**
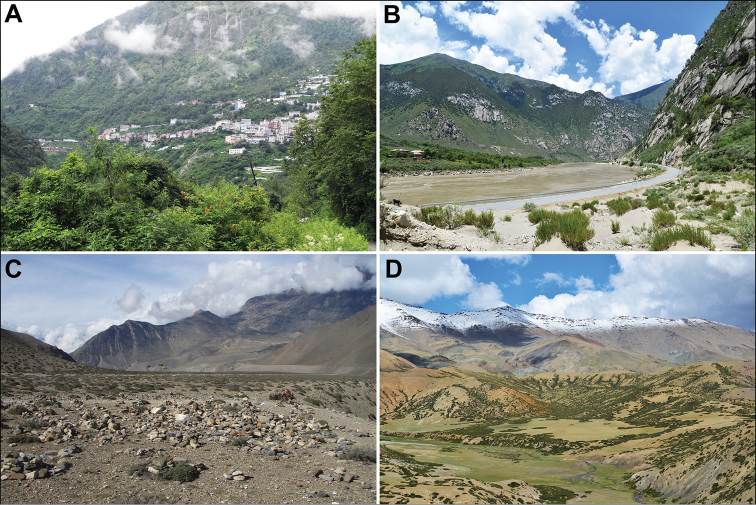
Typical habitats of Himalayan and Tibetan Chenopodiaceae: **A** Subtropical forest in Zham, Xigazê Prefecture, Xizang (2000 m a.s.l.) **B** Tarlung Tsangpo Valley in Nang County, Nyingchi Prefecture, Xizang (3100 m a.s.l.) **C** stony desert near Jomosom, Dhaulagiri zone, Nepal (ca. 3000 m a.s.l.) **D** alpine desert in Zanda county, Ngari Prefecture, Xizang (4800 m a.s.l.). Photographs by A. Sukhorukov (**C**) and P.-L. Liu (**A, B, D**).

In general, the taxonomic diversity of the Chenopodiaceae is low in mountainous regions of the tropics compared with the steppes and deserts of temperate regions. The altitudes, where the Chenopodiaceae can be found, range from 0 to 5500 m above sea level. The diversity of representatives of Chenopodiaceae and their phytosociological role differ amongst three major altitudinal belts (Fig. 2).

The altitudes from 0 to 2200 m above sea level. The lower altitudes are appropriate only for several species that occupy (semi-)disturbed places. In Central and Eastern Himalaya, the annuals *Chenopodiumalbum* and *C.ficifolium* are common ruderal and segetal plants that vegetate in late winter and spring. *Dysphaniaambrosioides*, the only alien naturalised species from the tropical Americas, usually colonises dump sites along roads, but it can also be found on semi-disturbed river banks. *Chenopodiastrummurale* occupies various disturbed habitats, although its presence in Tibet and many regions of Himalaya has not yet been confirmed.

**Figure 3.**
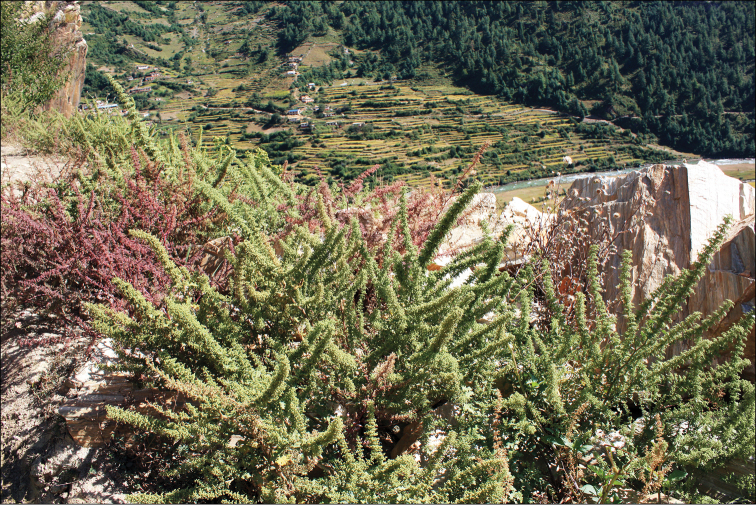
*Dysphanianepalensis* (red plant) at fruiting stage and *D.neglecta* (green plant) at blooming stage. Photograph by A. Sukhorukov (Midwestern Nepal, Mugu distr., October 2014).

The altitudes from 2200 to 3500 m above sea level. Most annual Chenopodiaceae growing at these altitudes are found on the screes, on limestone or sand or often in disturbed habitats (being apophytic). In North Himalaya and Tibet, some annuals that usually grow at 3500–5000 m above sea level can be found at lower altitudes (*Chenopodium* sp. div., *Corispermum* sp. div. and *Dysphania* spp.). In Central Himalaya, 2200–3500 m is the most appropriate altitude for *Acroglochinpersicarioides* and *Dysphanianepalensis*, where they are abundant, weedy species (Sukhorukov and Kushunina 2014). In West Nepal, *Chenopodiumperttii* and *Dysphanianeglecta* are common. Different *Chenopodium* species often grow together, e.g. *Dysphanianeglecta* and *D.nepalensis* in Nepal (Fig. 3).

The altitudes from 3500 to 5000(5500) m above sea level. This altitude range constitutes a zone of alpine steppes and cold deserts (Fig. 2) where three sub-shrubby Chenopodiaceae members play a significant role as the dominants of different plant communities. *Krascheninnikoviaceratoides* (s.l.) is one of the dominants of stony plant formations of North Himalaya (Hartmann 1983, 2009) and Tibet (Namgail et al. 2012, Hong and Blackmore 2015), with local distribution in Nepal, Central Himalaya (Yonekura 2008, Sukhorukov and Kushunina 2014). Other subshrubs have limited distribution patterns in the territory under study. *Bassiaprostrata* is mostly distributed in Jammu and Kashmir (India), where it can be abundant in subalpine deserts at altitudes of 3500–4000 m (Hartmann 2009). Another dwarf subshrub, *Haloxylonthomsonii*, is localised only in Ladakh and adjacent territories in Pakistan at altitudes of (3200)3500–4000 m, where it forms characteristic alpine semi-desert and steppe landscapes together with some *Artemisia*, *Caragana, Potentilla* and *Ptilotrichium* species (Dhar and Kachroo 1983, Hartmann 1983, 2009, Eberhardt et al. 2007, Gruber and Peer 2008). *Sympegmaregelii*, a common plant on the screes in Tian-Shan and Kunlun (Grubov 1966, Eberhardt et al. 2007), has been found in northwest Tibet during the last several decades.

Amongst the annuals, *Axyrismira*, *Axyrisprostrata*, *Corispermum* sp. div. and some *Dysphania* (*D.himalaica*, *D.nepalensis* and *D.tibetica*) are widespread in stony deserts, river basins and disturbed areas. *Microgynoeciumtibeticum*, a small, often-overlooked herb, reaches altitudes up to 5500 m, and this altitudinal zone appears to be the highest level at which only a few flowering plants are observed (Sukhorukov 2014). All Chenopodiaceae at the higher altitudes are native species growing in natural or degraded communities (Klimeš and Dickoré 2005, Miehe et al. 2009).

### Taxonomy and geographical subdivision of the species

In their recent circumscriptions, 20 genera are recognised in Himalaya and West Tibet, representing all subfamilies of Chenopodiaceae and 57 species have been recorded. The best-represented genera are *Chenopodium*, *Corispermum* and *Dysphania* (9 species each). None of the taxa can be recommended for inclusion in the IUCN Red List (IUCN 2017) since they are either dominant species, occupy ruderal habitats or are insufficiently studied.

In the past, very few species were recorded for the entirety of Himalaya and Tibet (within the borders of Xizang) and the number of Chenopodiaceae species reported for Tibet was only 15 (Grubov 1966) or 29 (Zhu et al. 2003). Fifteen species were reported for Lower Ladakh, India (Klimeš and Dickoré 2005). Here, we present the first attempt to evaluate the number of species in the main divisions of the region under study. The number of species was counted for all the countries of Bhutan and Nepal, the major administrative subdivisions of Xizang (prefectures) and the Indian states of Jammu and Kashmir, Himachal Pradesh, Uttarakhand and Sikkim (Fig. 1).

**Bhutan – 9; Nepal – 25; Xizang, China** – **42** species in total (Ngari Prefecture – 26, Nagqu Prefecture – 12, Xigazê Prefecture – 24, Lhasa City – 19, Shannan Prefecture – 16, Nyingchi Prefecture – 19, Qamdo Prefecture – 11); **India** – **43** species in total (Jammu & Kashmir – 41, Himachal Pradesh – 21, Uttarakhand – 15, Sikkim – 6).

Although phylogeographic analysis has yet to be conducted for the Asian Chenopodiaceae, the origin and migration routes of the widely distributed taxa may be more complex than previously thought (Li et al. 2010, Jia et al. 2012, Zhang et al. 2014, 2017). However, we note here that many taxa (42%) are clearly autochthonous, distributed only or predominantly in Himalaya and Tibet (*Acroglochinpersicarioides*, *Agriophyllumtibeticum*, *Axyrismira*, *Chenopodiumatripliciforme*, *C.bengalense*, *C.harae*, *C.perttii*, *Corispermumfalcatum*, *C.pseudofalcatum*, *C.lepidocarpum*, *C.lhasaense*, *C.nanum*, *C.tibeticum*, *Dysphaniabhutanica*, *D.himalaica*, *D.geoffreyi*, *D.neglecta*, *D.nepalensis*, *D.tibetica*, *Haloxylonthomsonii*, *Microgynoeciumtibeticum*, *Salsolaaustrotibetica, S.hartmannii*, *S.jacquemontii*). Many taxa (~28%) clearly demonstrate the distribution pattern across Central Asia (*Atriplexcentralasiatica*, *A.pallida*, *A.pamirica*, *Axyrisprostrata*, *A.sphaerosperma*, *Bassiascoparia*, *Chenopodiumkaroi*, *C.pamiricum*, *Corispermumdutreuilii*, *C.gelidum*, *Gruboviadasyphylla*, *Halogetonglomeratus*, *Salsolacollina*, *Suaedaolufsenii*, *Sympegmaregelii*, *Teloxysaristata*). *Bassiaodontoptera* has an Irano-Turanian origin.

Two species, *Atriplexhortensis* and *Dysphaniaambrosioides*, are adventive plants (ergasyophytes). The adventive status of *Chenopodiumalbum* and *C.ficifolium* is still not confirmed.

### Key to the genera

The key to the infrafamilial groups (subfamilies) and their characteristics are provided in some recent works (Sukhorukov 2014; Sukhorukov et al. 2015a, 2016b) and we chose not to repeat it in the present work. The generic key does not include *Spinaciaoleracea* L. (Chenopodioideae-Anserineae), which is often cultivated as a vegetable (especially in China) and *Beta vulgaris* L. (Betoideae), which is also sometimes present in vegetable gardens. Both species have no tendency to escape from cultivation.

**Table:** 

1	Plants with dendroid, branched (when main axis bears lateral outgrowths arranged one above the other) or stellate hairs (sometimes intermixed with simple hairs) covering all the plant or the young parts only	**2**
–	Plant glabrous or covered with papillae, simple, bladder (‘white meal’), glandular hairs or subsessile glands	**5**
2	Plants with stellate hairs, sometimes intermixed with long simple hairs; flowers unisexual (male inflorescence terminal; female flowers located below in the bract axils)	**3**
–	Plants covered with dendroid or branched hairs; simple hairs absent; flowers bisexual	**4**
3	Plants subshrublets or subshrubs; female flowers without perianth, enclosed by two bracts that are fused at least halfway; at fruiting, bracts slightly enlarged and covered with long simple and short-rayed stellate hairs	**10. *Krascheninnikovia***
–	Plants annuals; female flowers with a bract and free hyaline not enlarging perianth segments covered with simple hairs	**9. *Axyris***
4(2)	Hairs dendroid (additional outgrowths are small) on the stem and branched on the leaves; leaves and bracts quickly hardened, persistent; fruit dehiscent in the lower half	**13. *Agriophyllum***
–	Hairs branched (lateral outgrowths large); leaves and bracts not hardened, partially caducous; bracts of the same consistency; fruit indehiscent	**12. *Corispermum***
5(1)	Plants covered (usually densely) with straight (sometimes with curved) simple multicellular hairs; the majority or all hairs 2.0–7.0 mm long	**6**
–	Plants glabrous or with diverse types of indumentum; if long hairs present, they are clearly curved	**7**
6	Stem and leaves covered with large horizontally spreading white hairs (which often turn brownish after drying) and short (up to 0.3 mm), scattered, bright-yellow hairs; leaves terete, fleshy; fruits with star-like subulate spines; annual tumble-weed	**16. *Grubovia***
–	Stem and leaves covered with (semi)appressed or obliquely directed white hairs; bright-yellow hairs absent; fruits with wing-like or tuberculate outgrowths, rarely without any projections; annuals, subshrubs or subshrublets	**15. *Bassia***
7(5)	Leaves subulate or semi-terete, stout or fleshy; seeds with scant or no perisperm; embryo coiled	**8**
–	Leaves flat; seeds with abundant perisperm; embryo horseshoe-shaped or annular	**12**
8	Plants subshrubs or subshrublets	**9**
–	Plants annuals	**10**
9	Sub-shrublet with several pairs of opposite leaves in the lower part of the stem and alternate leaves in the upper part and in the inflorescence	**19. *Haloxylon***
–	Subshrub with alternate leaves (only bracts opposite)	**20. *Sympegma***
10(8)	Leaves fleshy, not mucronate or with a tiny mucro; bracteoles small, white; perianth with short conical outgrowths; seeds heterospermous (dark red and brown)	**14. *Suaeda***
–	Leaves (or at least bracts) fleshy or stout, with caducous or persistent mucro; bracteoles green or with reddish stripes, similar in shape to the bracts; perianth segments mostly with short or clearly visible wings at the fruiting stage; seed covers transparent (whitish)	**11**
11	Leaf mucro falls off easily (sometimes all leaves with reduced or hardly noticeable mucro); seed embryo vertically orientated	**18. *Halogeton***
–	Leaf mucro always persistent, not caducous; seed embryo horizontally or obliquely orientated	**17. *Salsola***
12(7)	Plants usually aromatic, covered with different sets of trichomes (simple and/or white glandular hairs) and yellow or orange glands (glands are sometimes very scattered and the pubescence mostly consists of simple hairs, as in *Dysphaniatibetica*)	**7. *Dysphania***
–	Plants without strong aromatic scent, glabrous or with white (sometimes with brown) bladder hairs or short and curved simple hairs; rarely, yellowish glandular hairs are present in the inflorescence	**13**
13	Leaves short (up to 3.0 cm)	**14**
–	Leaves (at least lower ones) longer than 3.0 cm	**15**
14	Flowers unisexual: male flowers with perianth and female flowers subtended by a leaf-like bract; pericarp forms easily visible ear-like outgrowths, at least in the upper part of the fruit; plant green, glabrous or sparsely farinose	**5. *Microgynoecium***
–	Flowers bisexual with perianth consisting of 5 segments; pericarp papillate (reticulate in dry fruits) or almost smooth without ear-like appendages; plant greyish-farinose	**1. *Chenopodium* (part)**
15(13)	Leaves sessile or subsessile, cuneate, entire, mostly curved ventrally	**8. *Teloxys***
–	Leaves clearly short- or long-petiolate, at least lower leaves not entire	**16**
16	Branches with acicular apices (rarely without them); leaves glabrous or with simple short hairs with small mucronate teeth; fruits dehiscent by a lid	**11. *Acroglochin***
–	Branches without acicular apices; leaves without mucro; if present, hairs of other shape; fruit indehiscent or pericarp ruptures irregularly	**17**
17	All or most flowers unisexual; male (and bisexual, if present) flowers with small hyaline actinomorphic perianth; each female flower subtended by bract-like cover consisting of two ± accrescent segments	**4. *Atriplex***
–	All or almost all flowers bisexual; perianth actinomorphic, not accrescent	**18**
18	Plant with basal (“rosulate”) and cauline leaves; perianth segments at fruiting red and fleshy; pericarp tightly adhering to the seed coat; seeds ovoid	**6. *Blitum***
–	Plant with cauline leaves only; perianth segments green or hyaline, not fleshy; pericarp not tightly adherent to the seed and ruptures or separates easily; seeds depressed globular, rarely ovoid	**19**
19	Plant prostrate or with ascending stems; perianth of 3–5 segments; pericarp smooth; seeds with both horizontally and vertically orientated embryo on the same plant	**3. *Oxybasis***
–	Plant with prominent erect stem; perianth of (4)5 segments; pericarp papillate (often reticulate or smooth when dry); seeds with horizontally orientated embryo	**20**
20	Leaves slightly cordate at base; seed diameter 1.5–2.0 mm; yellowish glandular hairs present in the inflorescence	**2. *Chenopodiastrum* (*C.badachschanicum*)**
–	Leaves cuneate or broadly truncate; seed diameter less than 1.5 mm; yellowish glandular hairs absent	**21**
21	Inflorescence short (usually up to 10 cm), leafy; seeds prominently keeled; leaves rhombic, dentate or erose-dentate; plants growing below 2000 m a.s.l.	**2. *Chenopodiastrum* (*C.murale*)**
–	Inflorescence mostly larger and often leafless; seeds keeled or not; leaves trilobate, dentate or entire; plants growing at various altitudes	**1. *Chenopodium* s. str.**

### Subfam. Chenopodioideae

#### Tribe Chenopodieae

##### 1. Chenopodium

Taxon classificationPlantaeCaryophyllalesChenopodiacea

L.

###### Type.

*Chenopodiumalbum* L. (typ. cons. prop.)

###### Notes.

A formal proposal to conserve the name *Chenopodium* L. with the type *Chenopodiumalbum* L. has only recently been put forward (Mosyakin 2015). The conservation of the name with this type would be most desirable to maintain the recent taxonomy of *Chenopodium* and its relatives, especially *Oxybasis*.

###### Description.

Annuals, shrubs, or rarely small trees, covered with bladder hairs. Leaves petiolate, usually lobed or dentate (sometimes entire), very rarely semi-terete. Inflorescences paniculate, composed of small cymose clusters. Flowers sessile and pedicellate, hermaphrodite or some female. Perianth segments 5 (rarely 4), free or basally concrescent, green and unchanging at fruiting. Stamens usually 5, free or basally connate. Stigmas 2, free. Fruit depressed globular, falling off separately or together with perianth. Pericarp mostly thin, hyaline, of 1–2(3) parenchymatous layers, usually with small cylindrical or conical papillae (in dry fruits, the pericarp surface resembles honeycombs, but after soaking, the papillae retrieve their shape). In some species now transferred from *Einadia* to *Chenopodium*, the pericarp (at least in most fruits) is fleshy (berry), coloured, and many-layered, but some of the fruits remain dry (heterocarpy). Seeds black or rarely brown with horizontal embryo; the cell walls of testa (outer seed-coat layer) cells in black seeds with vertical stalactites, rarely the stalactites absent (three species from Juan-Fernández Archipelago).

In its current circumscription (Fuentes et al. 2012), the genus comprises more than 100 species, but the exact number is still unknown. The species are mostly distributed in extratropical parts of the world or in mountainous regions of the tropics.

##### Key to the species

**Table:** 

1	Plant usually robust, up to 2.5 m tall; young leaves trilobate, mature leaves up to 25(30) cm long	**6. *C.bengalense***
–	Plant 15–80(100–200) cm; leaves much smaller	**2**
2	Leaves lobate with the terminal lobe two to four times as long as the lateral ones	**3. *C.ficifolium***
–	Leaves without lobes or lobate; in the latter case, the apical lobe slightly (up to two times) or not larger than the lateral ones	**3**
3	Seeds evidently dimorphic: dark-red to blackish, subspherical, marginally keeled and brown, flattened and marginally rounded; one of the types often predominates on the plant	**2. *C.pamiricum***
–	All seeds black	**4**
4	Inflorescence “spiciform” in appearance (branches with loosely arranged clusters of 2–4 flowers); leaves triangular, basally truncate or broadly cuneate, blades not lobate	**8. *C.atripliciforme***
–	Inflorescence not spiciform, clusters prominent, of 4–10 flowers	**5**
5	Plant prostrate to ascending; leaves mostly entire, rhombic or slightly trilobate with indistinct lateral lobes	**5. *C.karoi***
–	Plant (sub)erect; at least the lower leaves dentate or lobate	**6**
6	Leaves truncate or slightly cuneate; at least middle and upper leaves lobate, middle lobe gradually tapering; pericarp almost smooth when dry and with minute papillae in fresh fruits or after soaking; seeds with distinct keel	**9. *C.perttii***
–	Leaves cuneate, without lobes or only slightly lobate; pericarp papillate, rarely mamillate or almost smooth; seeds without keel or with indistinct keel	**7**
7	Leaves (at least abaxially) white farinose	**7. *C.novopokrovskyanum***
–	Leaves green or greyish	**8**
8	Leaves not fleshy, rhombic to lanceolate, long-petiolate, not fleshy, abaxially green or greyish	**1. *C.album***
–	Leaves fleshy, oblong, short-petiolate (petioles to 1 cm), abaxially grey	**4. *C.harae***

###### 1. Chenopodium album

Taxon classificationPlantaeCaryophyllalesChenopodiacea

L., Sp. Pl.: 219 (1753)

Chenopodium album L., Sp. Pl.: 219 (1753). **Lectotype** (designated by Brenan 1954): Herb. Linn. 313.8 (LINN! image of the lectotype available at http://linnean-online.org/3082/).
≡Botrysalba (L.) Nieuwl., Am. Midl. Naturalist 3: 276 (1914).

####### Taxonomic notes.

The description of the species is based on the most characteristic features of the populations growing in West/Central Himalaya; this aggregate still requires further investigations in our territory and other parts of Eurasia. A strange specimen was seen from Ladakh (Zanskar, Tsarap, Kuru Village to Tetha Village, 3950 m alt., 21 Aug 2004, 33°10'18"N, 77°10'32"E, *L. Klimeš 4163*, PRA) bearing a small (up to 15 cm) plant with entire, ovate leaves up to 2 cm, a small inflorescence up to 5 cm long and pitted seeds that may represent a new taxon. Some plants of *C.album* from India are characterised by smooth pericarp, a trait of *C.perttii*, although they differ from the latter taxon in leaf shape.

####### Description.

**Figure 4.**
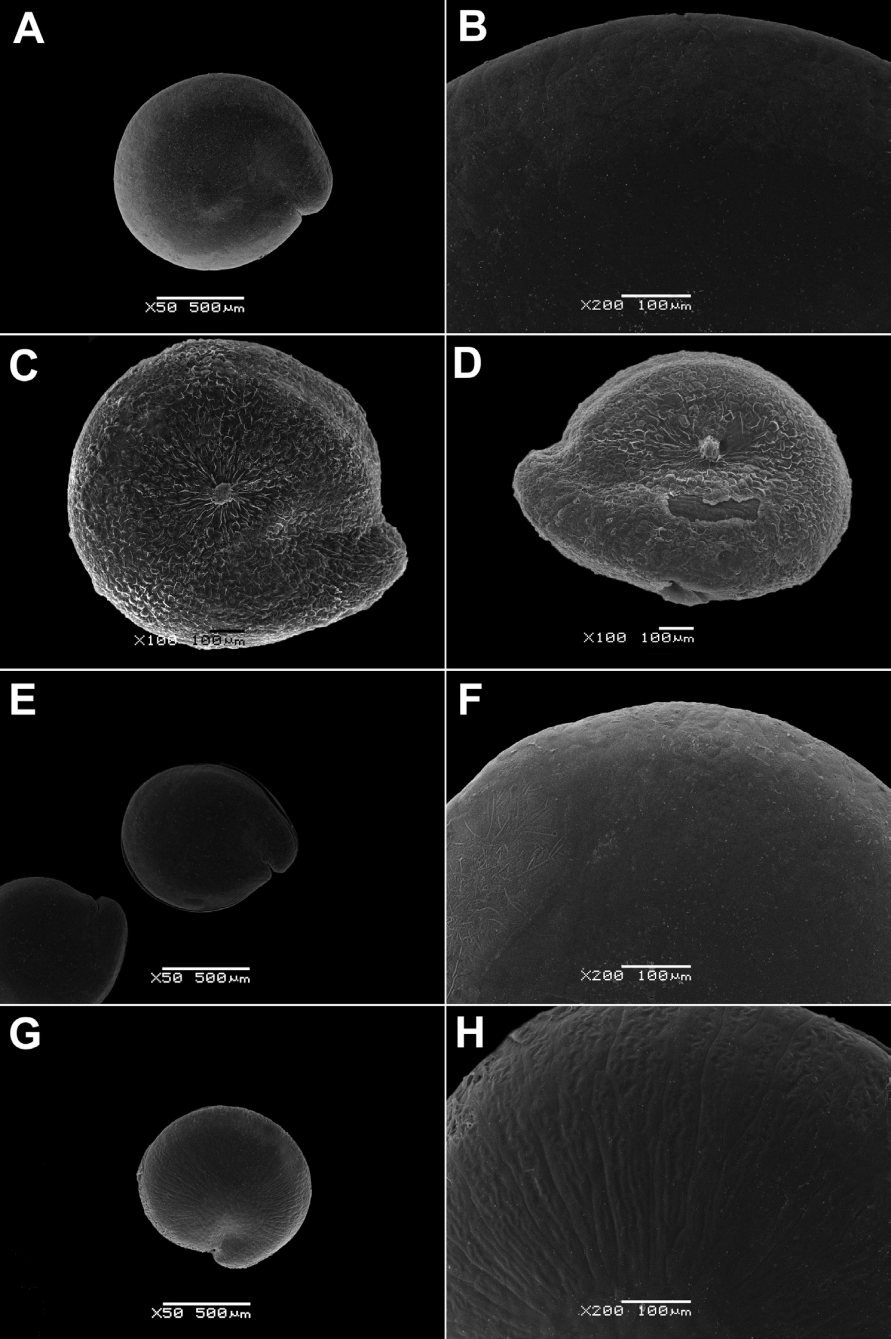
SEM micrographs of *Chenopodium* fruits and seeds. **A, B** seed of *C.album***C***C.pamiricum*, fruit with red seed **D***C.pamiricum*, fruit with brown seed **E, F***C.pamiricum*, red seed **G, H** seed of *C.ficifolium*. Magnification: 50× (**A, E, G**), 100× (**C, D**); 200× (**B, F, H**).

Annual, up to 150 cm, erect, branched, green. Leaves up to 10 × 4 cm, rhombic, oblong or lanceolate, entire or dentate, lower leaves often 3-lobed (terminal lobe tapering to apex). Inflorescence leafy or not. Flowers in glomerules arranged in loose inflorescence. Perianth farinose or glabrous. Fruit 1.3–1.5 mm, pericarp papillate (papillae up to 80 µm), can be scraped off the seed; seeds obtuse or acutish on margins, nearly smooth with shallow radial furrows (Fig. 4A, B), with structural heterospermy expressed in different thickness of the seed-coat testa.

####### Habitat.

Disturbed areas; 0–4500 m a.s.l. The most common chenopodiaceous species in Himalaya and Tibet.

####### Phenology.

Flowering: March-September; fruiting: April-November.

####### Distribution.

See Fig. 5.

**Figure 5.**
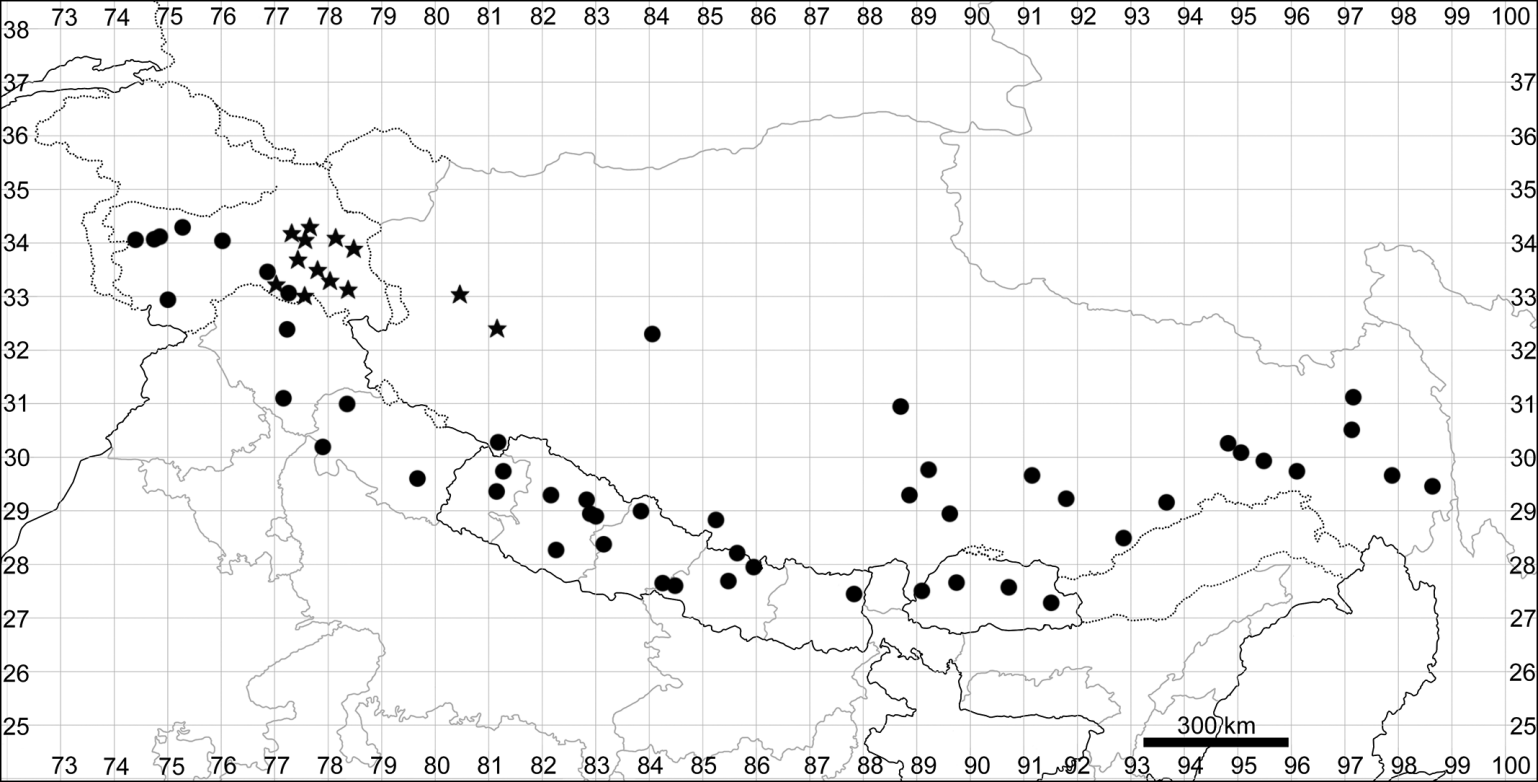
Distribution map of *Chenopodiumalbum* (circles) and *C.pamiricum* (stars).

####### Specimens examined.

**BHUTAN**: Charithang, Aug 1938, *B.J. Gould 1396* (DD); Byakar, 27°33'N, 90°44'E, 2700 m a.s.l., 9 Jun 1979, *A. Grierson & D. Long 1779* (K, E00168228); Upper Mo Chu distr., Chebesa, 3880 m a.s.l., 27 Sep 1984, *I. Sinclair & D. Long 5361* (E00168227); Tashigang distr., nr Rolong, 800 m a.s.l., 20 Mar 1991, *C. Parker 4926* (E00168225);

**CHINA**: **Xizang**: **Ngari Prefecture**: Gêrzê (Gaize) County, 4250 m a.s.l., Aug 1972, *Li 063* (PE00510181), *Li 070* (PE00510179); Burang (Pulan) County, 4100 m a.s.l., 14 Jul 1976, *Qinghai-Tibet Team 76-8449* (PE00510198); **Nagqu Prefecture**: Xainza (Shenzha) County, 4500 m a.s.l., 19 Sep 1976, *Qinghai-Tibet Team Vegetation Group 13764* (PE00235194); **Xigazê Prefecture**: Gyangtse (Gyangzê, Jiangzi), 4 Jul 1904, *H.J*. *Walton 38* (K); Xigazê (Rikaze), 3830 m a.s.l., 7 Jul 1960, *Fu 348* (PE00510208); Nyalam (Nielamu) County, Zham (Zhangmu), 2000 m a.s.l., 13 May 1966, *Zhang & Lang 3361* (PE00510188); Namling (Nanmulin) County, Gangba, 3 Aug 1972, *Tibet Chinese Herbal Medicine Survey Team 993* (PE00510200); Gyangzê (Jiangzi) County, 3960 m a.s.l., 5 Sep 1974, *Qinghai-Tibet Team 74-2072* (PE00510201, KUN0586975); Gyirong (Jilong) County, nr Mangmu, 2300 m a.s.l., 16 Jul 1975, *Qinghai-Tibet Team 6908* (PE00510190); Gyirong (Jilong) County, 4100 m a.s.l., 28 Jul 1975, *Qinghai-Tibet Team Vegetation Group 5635* (PE00510212); **Lhasa City**: Lhasa, 4000 m a.s.l., 20 Aug 1965, *Zhang & Lang 2055* (PE00510192); **Shannan Prefecture**: Lhünzê (Longzi) County, Sangngagqoiling (San’anqulin), 2710 m a.s.l., 15 Jul 1975, *Qinghai-Tibet Team Additional Group 750596* (PE00510175); [Nêdong (Naidong) County] Zêtang (Zedang), 3500 m a.s.l., 11 Aug 1977, *B.Z. Guo* et al. *22350* (WUK0345060); **Nyingchi Prefecture**: Bomi County, Guxiang, 29°55'N, 95°30'E, 2550 m a.s.l., 3 Jul 1965, *Ying & Hong 650415* (PE00510164); Bomi County, Tangmai (Tongmai), 2030 m a.s.l., 23 Jul 1965, *Zhang & Lang 892* (PE00510166); Bomi County, Sumzom (Songzong), 3500 m a.s.l., 4 Sep 1965, *Ying & Hong 651075* (PE00510171); Bomi County, Yi’ong (Yigong), 2400 m a.s.l., 19 Jun 1966, *Zhang & Wang 0401* (PE00510223); Mainling (Milin) County, Jiage, 3100 m a.s.l., 10 Jul 1972, *Tibet Chinese Herbal Medicine Survey Team 3660* (PE00510218); **Qamdo Prefecture**: Qamdo (Changdu) County, 23 Aug 1952, *Zhong 7203* (PE00510204); Qamdo (Changdu) County, 6 July 1965, *Zhang & Lang 230* (PE00510172), *Zhang & Lang 232* (PE00510174); Qamdo (Changdu) County to [Baxoi (Basu) County] Bamda (Bangda), 3150 m a.s.l., 6 Jul 1965, *Zhang & Lang 403* (PE00510173); Zogang (Zuogong) County, 3750 m a.s.l., 28 Jun 1976, *Qinghai-Tibet Team Vegetation Group 8689* (PE00510216); Markam (Mangkang) County, nr Dengba vill., 30 Aug 2011, *Yu* et al. *6149* (PE);

**INDIA**: **Jammu & Kashmir**: Pata, 7500 ft a.s.l., 28 May 1848, *T.T*. *Thomson* s.n. (K); Srinagar, [year] 1856, *Schlagintweit 4286* (G); Sonamarg, 1 Sep 1896, *C.B*. *Clarke 30890* (K); [Srinagar] Dal lake, 17 Aug 1917, *R.R*. *Stewart 3324* (K); Ladakh, Zanskar Region, S of Padum, Dibling to Barmi La, 3730 m a.s.l., 24 Aug 2003, 33°54'18"N, 76°41'6"E, *L. Klimeš 3227* (PRA); Ladakh, Zanskar, Tsarap, Kuru to Tetha, 3950 m a.s.l., 21 Aug 2004, 33°10'18"N, 77°10'32"E, *L. Klimeš 4163* (PRA); Ladakh, Suru Region, Parkachik, 3620–3740 m a.s.l., 25 Aug 2005, *L. Klimeš 6048* (PRA); **Himachal Pradesh**: Simla, 4 Jul 1877, *J.S*. *Gamble 4500* (K); Simla, 7000 ft a.s.l., 10 Jul 1886, *H. Collett 311* (K); Simla, 8 Oct 1890, *Watt 13674* (E); Lahaul, Koksu Lahaul, 10000 ft a.s.l., 7 Aug 1916, *R.E*. *Cooper 5283* (E00394442); Tangmang, 6000 ft a.s.l., 17 Aug 1956, *Polunin 56-363* (BM); **Uttarakhand state**: Kumaon, Almora, 5500 ft a.s.l., [without date] *S. Strachey & J.E. Winterbottom 5* (BR, BM); 20 km SW of Dehradun, 670 m a.s.l., 15 Jun 1972, *P. Uotila 17668* (H1154022); Tehri Garhwal Distr., Kharsali, 3 Oct 1991, *S.C. Majumdar & B. Balodi 81561* (BSD);

**NEPAL**: **Far-Western**: **Seti Zone**: Bajhang Distr., 19 Jul 1991, *Suzuki* et al. *9160766* (BM); Bajhang Distr., Khaptad National Park, trail from Ghoda Daune to Lokhada, 29°24'N, 81°8'E, 2559 m a.s.l., 3 Jul 2009, *H. Ikeda* et al. *20913048* (E00509893); **Midwestern**: **Karnali Zone**: [Dolpa Distr.] nr Tarakot, Bheri river, 10500 ft a.s.l., 11 Jul 1952, O. *Polunin*, *W.R*. *Sykes* & *L.H.J*. *Williams 2432* (BM, E); [Dolpa Distr.] Dunai, 28°55'N, 82°55'E, 2100 m a.s.l., 26 Apr 1974, *J.-F*. *Dobremez 2761* (BM, E00214373); Dolpa Distr., Polam, 29°10'N, 82°50'E, 3250 m a.s.l., 6 Oct 1991, *M. Minaki* et al. *9104404* (BM); [Jumla Distr.], Jumla vill., 23 Sep 2010, *A. Sukhorukov 463* (MW); **Rapti Zone**: [Rolpa Distr.] Phalabang, 4500 ft a.s.l., 27 Mar 1952, *O. Polunin, W.R. Sykes & L.H.J. Williams 654* (E); **Western**: **Dhaulagiri Zone**: [Baglung Distr.] nr Bongakhani, 500 ft a.s.l., 5 May 1954, *J. Stainton*, *W.R*. *Sykes* & *L.H.J*. *Williams 2708* (BM); Mustang Distr., 3185 m a.s.l., 22 Sep 1995, *M. Mikage* et al. *9550337* & *9550357* (BM); **Lumbini Zone**: Nawalparasi [Distr.], Beldiha, 150 m a.s.l., Nov 1970, *T. Makino 10* (BM); **Central**: **Bagmati Zone**: [Rasuwa Distr.] Langtang vill., 11500 ft a.s.l., 1 Aug 1949, *O. Polunin 1552* (BM); Langtang, 11500 ft a.s.l., 22 Jun 1949, *O. Polunin 490* (BM); [Bhaktapur Distr.] Nagarkot to Bhaktapur, Mar 2012, *A. Sukhorukov* s.n. (MW); **Narayani Zone**: Chitwan Distr., Sauraha, 160 m a.s.l., 19 Jan 1996, *M. Mikage* et al. *9614220* (E00152131); Chitwan National Park, Sauraha, 4 Mar 2008, *A. Sukhorukov 56* (MW, E00665443); **Eastern**: **Mechi Zone**: [Taplejung Distr.] Talung, 2600 m a.s.l., 3 Oct 1971, *C. Jest 71-18* (E00214371).

####### General distribution.

Subcosmopolitan.

###### 2. Chenopodium pamiricum

Taxon classificationPlantaeCaryophyllalesChenopodiacea

Iljin, Fl. SSSR 6: 873 (1936)

####### Holotype.

[TAJIKISTAN] Pamir Orientale, declivia ad confluentes Baital et Murgab [Bartang], [East Pamir, slopes near confluence of Baital and Murgab rivers], 1 September 1923, *H. Raikova 417* (TASH – photo seen (!) and fragments of type material (!); isotype – LE!).

####### Taxonomic notes.

Morphologically, *C.pamiricum* can be easily confused with *C.vulvaria*, but all data on the presence of *C.vulvaria* in mountainous regions of Central Asia belong to *C.pamiricum*. In contrast to the latter species, either fresh or dry leaves of *C.pamiricum* do not have a scent. Further investigations have shown that the type material and many other specimens of *C.pamiricum* have evident heterospermy, a rare characteristic in *Chenopodium* s. str. discovered by Sukhorukov and Zhang (2013).

####### Description.

Annual up to 25 cm with several or numerous prostrate or ascending stems. Leaves farinose, shortly petiolate (petioles up to 1.5 cm but usually much smaller) with blades up to 2 cm, rhombic or ovate, entire or subhastate. Inflorescence leafy almost to the top. Flowers in glomerules arranged in ± dense inflorescence. Perianth farinose. Fruits and seeds heteromorphic. The first type of fruits and seeds is formed in terminal flowers, the fruits ripen in July-August. These fruits are ca. 1 mm in diameter and 0.5 mm thick; pericarp with papillae up to 20(25) μm (Fig. 4C); seed dark red or blackish with median keel (Fig. 4E, F), testa 17–25 μm thick. Fruits of the second type (which dominates at the end of the vegetation period) are ovoid, 1.0–1.2 mm long, ca. 0.5 mm thick, pericarp papillate or not (Fig. 4D); seeds yellow-brownish, elongated.

####### Habitat.

Sandy or rocky places, 3400–5000 m a.s.l. (in Himalaya) and at lower altitudes in Central Asia.

####### Phenology.

Flowering: June-September; fruiting: August-October.

####### Distribution.

See Fig. 5.

####### Specimens examined.

**CHINA** (new records for China): **Xizang: Ngari Prefecture**: Gê’gyai (Geji) County, Yanhu Distr., 4450–4500 m a.s.l., 24 Aug 1976, *Qinghai-Tibet Team Vegetation Group 13541* (PE00235028); Rutog (Ritu) County, Rabang (Rebang), 4360 m a.s.l., 28 Aug 1976, *Qinghai-Tibet Team Vegetation Group 13653* (PE00235010).

**INDIA**: **Jammu & Kashmir**: [Ladakh] Nubra [valley], Kardong [La], Aug 1856, *Schlagintweit 2180* (BM); Rupshu, Rachogba, 13400 ft a.s.l., 24 Jun 1931, *W. Koelz 2105* (LE); [Ladakh] Taglang La, 4820 m a.s.l., 3 Aug 1992, *H. Hartmann 4014*(G455287); Ladakh, Rupshu [Plateau], Tso Kar [lake], 4600 m a.s.l., 22 Aug 1995, *H. Hartmann 5037* (MSB137926); Ladakh, Zanskar Region, Ruberung La, 4300 m a.s.l., Aug 1997, *L. Klimeš* s.n. (PRA); Ladakh, Zanskar, Tsarap, 4180 m a.s.l., 5 Sep 1998, *L. Klimeš 380* (PRA); Indus valley, Stot (E) [Stod River valley], Nyi, 4600–4700 m a.s.l., 2 Sep 2002, *L. Klimeš 6176* (PRA); Ladakh, Pangong Region, Spangmik, 4300 m a.s.l., 9 Sep 2002, *L. Klimeš 2676* (PRA); Indus valley, Stot (E), Sumdo Gonma to Kiagar La, 4690 m a.s.l., 7 Sep 2003, *L. Klimeš 3463* (PRA); Shyok (E), Laga vill., 4220–4230 m a.s.l., 12 Sep 2003, *L. Klimeš 2745* (PRA); Indus valley, Sham (W), Basgo to Nye, 3490–3590 m a.s.l., 7 Aug 2006, *L. Klimeš 6712* (PRA).

####### General distribution.

Central Asia south to North Himalaya and North Tibet. For a long time, *C.pamiricum* has been considered endemic to Pamir (Iljin 1936, Pratov 1972). The analysis of the material collected in the fruiting stage proved that the range of *Chenopodiumpamiricum* covers almost all parts of Central Asia with records in Mongolia, West China (Xizang and Xinjiang), North Pakistan, North India, Kyrghyzstan, Tajikistan, Russia (Altay Mountains) and Eastern Kazakhstan. The records from Pamir, Karakoram, NW Tibet and North Himalaya represent the southernmost boundary of the distribution of *C.pamiricum*.

###### 3. Chenopodium ficifolium

Taxon classificationPlantaeCaryophyllalesChenopodiacea

Sm., Fl. Brit. 1: 276 (1800)

####### Lectotype

(Al-Turki and Ghafoor 1996): figure 16 in Curtis, Fl. Londinensis 1, fasc. 2 (1778).

####### Taxonomic notes.

The species is described from Britain: “In fimetis et ruderatis [growing on wasteland and dungheaps] about London, *W. Curtis*” (Smith 1800). The material cited in the protologue by Smith (1800) is probably lost. The specimen collected in England [Yarmouth, Sep 1801, Mr. D. Turner] and kept at LINN-HS (sheet 459.24) is not a part of the original material used for the description. Citing the “type”, Al-Turki and Ghafoor (1996) refer to the drawing of Curtis (1778, fig. 16), but Curtis did not describe any *Chenopodium* in his work and erroneously labelled his fig. 16 as “*Chenopodiumviride*”, although the image of the plant completely corresponds with *Chenopodiumficifolium* later described by Smith (1800). Citing the type of *Chenopodiumficifolium*, Al-Turki and Ghafoor (1996) have unintentionally lectotypified the species with the drawing in Curtis (1778). The chosen lectotype shows the plant with clearly trilobate leaves with prominent and elongated middle lobe (Chenopodiumficifoliumsubsp.ficifolium: Uotila 1997, 2001).

####### Description.

Annual up to 60(80) cm, loosely branched, stem green but often with red stripes. Leaves bright green, long-petiolate, 2–7 × 1–3 cm, three-lobed; apical lobe 2–4 times longer than lateral lobes; lateral lobes in the lower part of leaf short, entire or sinuate; apical lobe long, narrow with ± parallel margins, entire or irregularly sinuate-dentate. Inflorescence leafy in the lower and middle parts, short (up to 15 cm). Perianth green, enclosing the fruit; dorsal segments slightly carinate. Fruit 1.2–1.5 mm in diameter, pericarp separating from the seed, papillate (honeycomb-like when dry). Seed marginally rounded or slightly keeled; testa longitudinally striate (Fig. 4G, H), slightly sinuate in cross-section.

####### Habitat.

Disturbed places, river-sides, sands; 0–2200(3000) m a.s.l. Common at least in many parts of Nepal and Bhutan. Numerous seedlings were observed by AS near Mussoorie (Uttarakhand, India) in mid-February 2018.

####### Phenology.

Flowering: February-May; fruiting: April-June. At higher altitudes (where the plant is rare), the blooming and fruiting times appear to be much later.

####### Distribution.

See Fig. 6.

**Figure 6.**
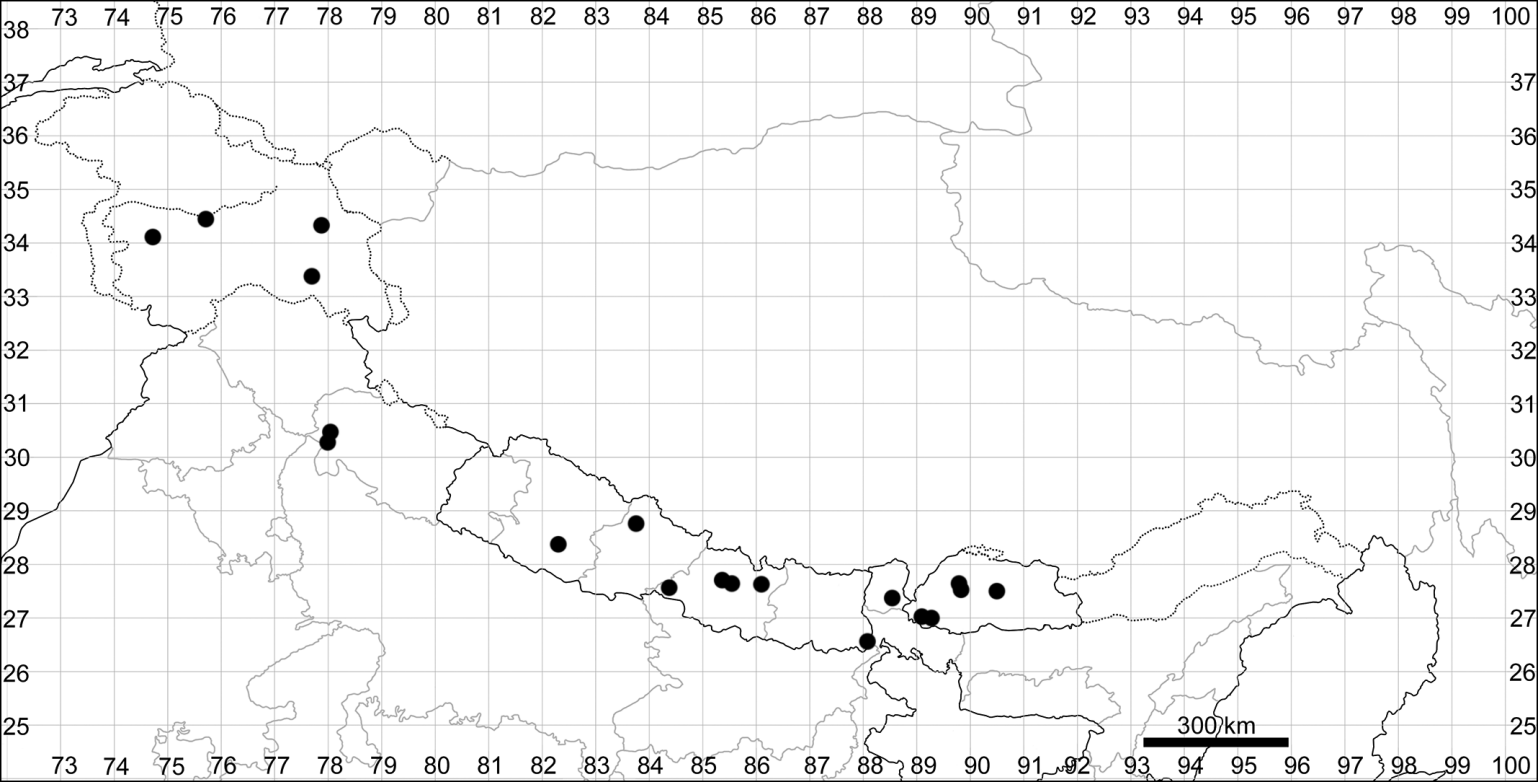
Distribution map of *Chenopodiumficifolium*.

####### Specimens examined.

**BHUTAN**: Torsa valley, Jan 1912, *anonym* s.n. (E00168226); Samchi Distr., Samchi [Samtse], 500 m a.s.l., 27 Feb 1982, *A. Grierson & D. Long 3267* (E00168235, K); Tongsa Distr., Pertimi, 1300 m a.s.l., 5 Apr 1982, *A. Grierson & D. Long 4336* (K); Punakha Distr., Toiberong Chu, 1310 m a.s.l., 21 Apr 1982, *A. Grierson & D. Long 4584* (E, K); Punakha Distr., Wangdi [Wangdue] Phodrang, 1200 m a.s.l., 12 Mar 1991, *C. Parker 4873* (E);

**INDIA**: **Jammu & Kashmir**: Srinagar, [year] 1856, *Schlagintweit 4286* (G); Ladakh, Zanskar Region, Zara, Sangtha vill., 4420–4430 m a.s.l., 31 Aug 2001, *L. Klimeš 1454* (PRA); Ladakh, Shyok Region, Hodtong, 3280–3320 m a.s.l., 26 Sep 2004, *L. Klimeš 5063* (PRA); Ladakh, Dras Region, Matayan to Pindras, 3220 m a.s.l., 15 Aug 2005, *L. Klimeš 5747* (PRA); **Sikkim**: 4000 ft a.s.l., 28 May 1874, *anonym 158* (K, LE); **Uttarakhand**: Dehradun, Niranjanpur, 700 m a.s.l., 25 May 1962, *N.P. Singh 19649* (BSD); Dehradun, 600 m a.s.l., 26 Mar 1964, *N.P. Singh 31695* (BSD); Dehradun city (Bot. Garden) and nr Mussoorie, obs. by AS in Feb 2018;

**NEPAL**: **Western**: Gandaki Zone: [Kaski Distr.] Burungdi Khola, 5000 ft a.s.l., 20 May 1954, *J. Stainton, W.R. Sykes & L.H.J. Williams 5344* (BM); **Midwestern**: Rapti Zone: [Salyan Distr.] Lawamjula, 3000 ft a.s.l., 28 Mar 1952, *O. Polunin, W.R. Sykes & L.H.J. Williams 686* (BM, LE); **Central**: Bagmati Zone: Kathmandu, 5000–7000 ft a.s.l., 4–8 Feb 1857, *anonym 13090* (BM); Kathmandu, 4500 ft a.s.l., 17 May 1969, *L.H.J*. *Williams 38* (BM); Kathmandu Distr., Kathmandu, N bank of Bagmati river, Thapathali, 1300 m a.s.l., 20 Apr 1992, *D.G*. *Long* & *S.E*. *McDermott 21909* (E); Janakpur Zone: [Dolakha Distr.] Namdu, Apr 1995, *Bhandari* s.n. (KATH); Narayani Zone: Chitwan [Distr.], 4 Mar 2008, *A. Sukhorukov* s.n. (MW); [Bhaktapur Distr.] Nagarkot to Bhaktapur, 1700 m a.s.l., 19 Mar 2012, *A. Sukhorukov* s.n. (MW); **Eastern**: Mechi Zone: [Jhapa Distr.] Bhadrapur, 1987, *anonym* s.n. (KATH).

####### General distribution.

Widespread in the tropical submountainous territories of SE Asia (e.g. China, Myanmar), where it may be native. Rarely found in the temperate climates of Eurasia and America.

###### 4. Chenopodium harae

Taxon classificationPlantaeCaryophyllalesChenopodiacea

Sukhor., Phytotaxa 226(3): 290 (2015)

=Chenopodiumpallidum sensu Sukhorukov (in Sukhorukov and Kushunina 2014), non Moquin-Tandon (1840).

####### Holotype.

NEPAL, [Dhaulagiri Zone], Mustang Prov. [Distr.], Annapurna Conservation area, Trekking route Jomosom-Muktinath, valley of Khali Gandaki river, between Jomosom and Kagbeni villages, riverside, 2700 m alt., 22 Sep 2009, *A. Sukhorukov 566* (MW!).

####### Taxonomic notes.

In the latest revision of the Chenopodiaceae in Nepal (Sukhorukov and Kushunina 2014), this species is cited as *Chenopodiumpallidum* Moq., but a closer look at the authentic specimens in P has proved that all plants (collected in early blooming stage) are indeed *Atriplexpallida*, now synonymised with *A.schugnanica* Iljin (Sukhorukov and Kushunina 2015).

####### Description.

Annual up to 40 cm with erect stem; lateral branches ascending if present. Leaves short-petiolate (petioles to 1 cm) with blades up to 2 × 1 cm, fleshy, ovate, slightly cuneate, green above, greenish below, dentate, margins often red. Inflorescence bracteate or aphyllous. Perianth green, enclosing the fruit; segments abaxially carinate. Fruit 1.3–1.5 mm, pericarp easily separating from the seed; papillae up to 25 µm. Seed blackish or reddish-black, surface striate with obtuse or acutish margin (immature seeds with acute margin) with small depression apically.

####### Habitat.

Disturbed areas; 1500–3200 m a.s.l.

####### Phenology.

Flowering: March-September; fruiting: April-November.

####### Distribution.

See Fig. 7.

**Figure 7.**
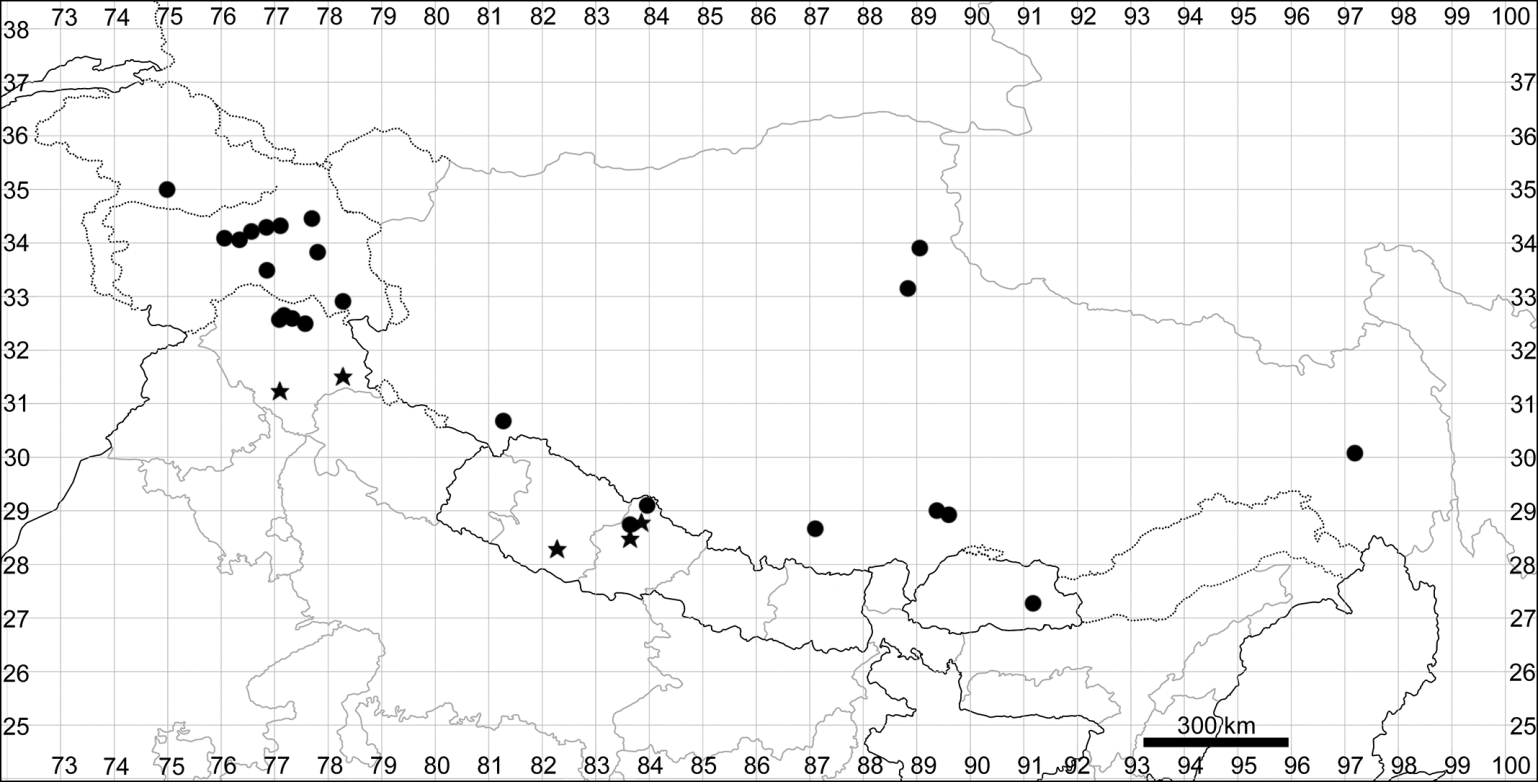
Distribution map of *Chenopodiumharae* (stars) and *C.karoi* (circles).

####### Specimens examined.

**INDIA: Himachal Pradesh**: Kinnaur Distr., Shongtong, 11 Jun 1972, *K.P. Janandhanan 48358* (BSD); Simla Distr., Seoni, 2 Jun 1986, *P.C. Pani 80903* (BSD);

**NEPAL**: **Midwestern**: **Rapti Zone**: [Rolpa Distr.] Phalabang, 4500 ft a.s.l., 24 Mar 1952, *O. Polunin, W.R. Sykes & L.H.J. Williams 654* (BM, LE); **Western**: **Dhaulagiri Zone**: Mustang [Mustang Distr.], Jomosom to Kagbeni, 22 Sep 2009, *A. Sukhorukov 566* (E00757737, MW); Myagdi Prov. [Distr.], nr Dana vill., 1500 m a.s.l., 11 May 2010, *A. Sukhorukov 510* (MW).

####### General distribution.

Central and Northern Himalaya (India, Nepal, Pakistan [Gilgit: E!]).

###### 5. Chenopodium karoi

Taxon classificationPlantaeCaryophyllalesChenopodiacea

(Murr) Aellen, Fedd. Repert. 26: 149 (1929)

≡C.albumsubsp.karoi Murr, Neu. Übers. Farn & Blütenpfl. Vorarlberg 1: 97 (1923). **Lectotype** (designated here by Sukhorukov): [RUSSIA] Nerczynsk [Nerchinsk], dump places, 1892, *Karo 169* (G00405813!).
=C.prostratum Bunge in Herder, Acta Hort. Petrop. 10: 594 (1889) nom. illegit. non Schultes (1820). The name is a later homonym (ICN, art. 53.1). **Lectotype** (Uotila and Lomonosova 2016): [RUSSIA, Saha Republic] Nizhnekolymsk, 6 July 1832, *Sharypov 20* (LE01015991!).
=(?)C.jenissejense Aellen & Iljin, Fl. SSSR 6: 873 (1936). **Lectotype** (Uotila and Lomonosova 2016): [RUSSIA] Yenisey Prov., Turukhanskiy Krai [Krasnoyarskiy Krai, Turukhanskiy distr.], Kureyka river mouth, 66°30'N, 87°17'E, sandy and pebbly river bank, 7 Sep 1914, *N.I. Kuznetsov & V. Reverdatto 4102* (LE01015992! for individuals at the bottom and to the right; isolectotypes – G! LE! NS). This species was only recently synonymised with Chenopodiumkaroi (Uotila and Lomonosova 2016). However, C.jenissejense is quite distinct in its morphological characters and needs to be studied in detail.

####### Description.

**Figure 8.**
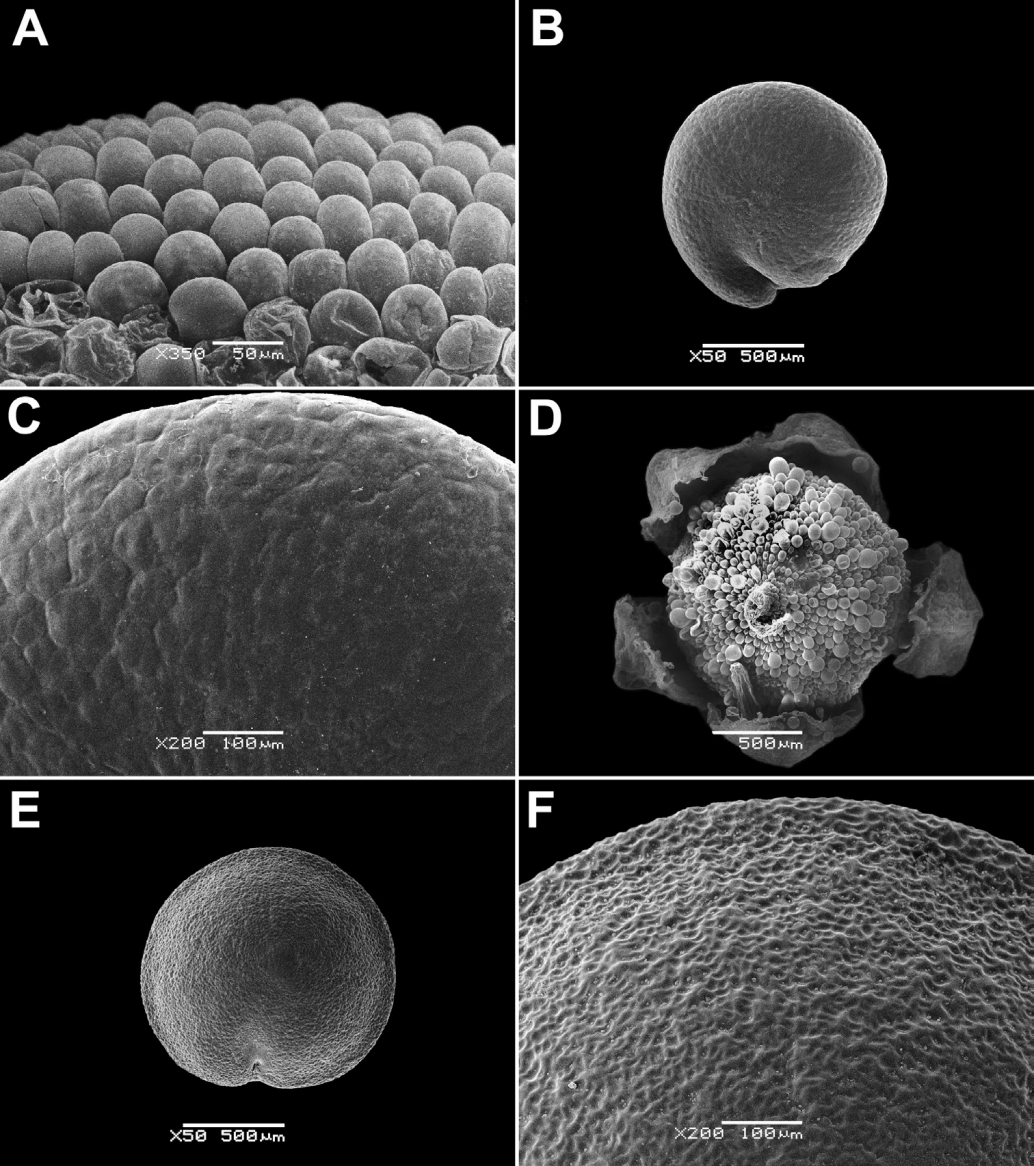
SEM micrographs of *Chenopodium* fruits and seeds. **A** pericarp surface of *C.karoi***B, C** seed of *C.karoi***D** fruit of *C.bengalense* enclosed in perianth **E, F** seed of *C.bengalense*. Magnification: 350× (**A**), 50× (**B, D, E**), 200× (**C, F**).

The species is similar to *C.album*, but plants are dark green, usually with ascendant or prostrate stems up to 25 cm. Leaves ± ovate, somewhat longer than broad, sometimes inconspicuously three-lobed. Fruit 1.1–1.3 mm, pericarp reticulate in dry fruits, soaked papillae up to 50 µm long (Fig. 8A). Seed margin obtuse, surface with sinuate furrows (Fig. 8B, C).

####### Habitat.

Disturbed areas, rocky habitats; 2500–5100 m a.s.l.

####### Phenology.

Flowering: May-August; fruiting: July-September.

####### Distribution.

Common plant in many parts of Himalaya and Tibet (Fig. 7).

####### Specimens examined.

**BHUTAN**: Mongar Distr., Lingmethang, 14 Jun 1992, *C. Parker 7241* (E);

**CHINA**: **Xizang**: **Ngari Prefecture**: Burang County, Langacuo lake, 4560 m a.s.l., 27 Aug 1990, *Y. Fei* et al. *667* (KUN0265513); **Nagqu Prefecture**: Shuanghu County, 4850 m a.s.l., 22 Jul 1976, *Qinghai-Tibet Team Lang 9780* (KUN0587328); N Xizang, Jialin, 5100 m a.s.l., 24 Jul 1976, *Gansu Agriculture University 088* (PE00541004); **Xigazê Prefecture**: Tingri, Jul 1921, *A. Wollaston 264* (BM); [Gyangste County] Kala [tso], 14000 ft a.s.l., 8 Aug 1936, *Chapman 499* (K); [Tingri County] Shelkar Dzong, 14000 ft a.s.l., 24 Jun 1938, *Lloyd 81* (K); [Gyantse] Gobshi, 14000 ft a.s.l., 16 Aug 1936, *Chapman 230* (K); Gyangzê (Jiangzi) County, 3960 m a.s.l., 5 Sep 1974, *Qinghai-Tibet Team 74-2070* (KUN0587330); **Qamdo Prefecture**: Baxoi (Basu) County, Nu river, 3000 m a.s.l., 5 Jul 1976, *Qinghai-Tibet Team 12208* (KUN0586973);

**INDIA**: **Jammu & Kashmir**: Ladakh, between Nubra & Leh, 4 Sep 1848, *T.T*. *Thomson* s.n. (K); Zanskar, Bok, 13 Sep 1931, *W. Koelz 2948* (LE); Ladakh, Uphse–Murul, 3500 m a.s.l., 7 Aug 1976, *B.M. Wadhwa 59711* (BSD); Ladakh, Saspole, 4200 m a.s.l., 29 Jul 1976, *B.M. Wadhwa 59341* (BSD); Ladakh, Rangdum, 4000 m a.s.l., 1980, *Southampton Univ. Team 126* (K); Ladakh, Kanji, 15300 ft a.s.l., 30 Jun 1984, *anonym 52* (K); Ladakh, Zanskar, Zara vill., 4330 m a.s.l., 31 Aug 2001, *L. Klimeš 1442* (PRA); Ladakh, Domkhar Dha, Kanji vill., 3900 m a.s.l., 18 Aug 2003, *L. Klimeš 3060* (PRA); Ladakh, Indus valley, Domkhar Dha, Kilchu vill., 4000 m a.s.l., 19 Aug 2003, *L. Klimeš* s.n. (PRA); Ladakh, Suru Region, Kanji Nullah, 4180 m a.s.l., 21 Aug 2003, *L. Klimeš 3161* (PRA); Ladakh, Zanskar Region, S of Padum, 4150 m a.s.l., 29 Aug 2003, *L. Klimeš 3322* (PRA); Ladakh, Rupshu region, Tso Moriri, 4550 m a.s.l., 13 Sep 2005, *L. Klimeš 6314* (PRA); **Himachal Pradesh**: Lahaul, Sep 1869, *anonym* s.n. (K); Lahaul, Jispa vill., 13000 ft a.s.l., 11 Aug 1930, *W. Koelz 1003* (P05158996); Badadara, 3975 m a.s.l., 11 Sep 1961, *N.C. Nair 16855* (BSD); Chandra Tal, 4 Jul 2003, *S.K. Srivastava 103111* (BSD);

**NEPAL**: **Western**: **Dhaulagiri Zone**: [Mustang Distr.] Kali Gandaki, ESE of Thini [Thinigaun], 12500 ft a.s.l., 19 Jul 1977, *G. Miehe 227* & *228* (BM); Mustang [Distr.] Lo Tsho Dhyun, Yara [Yara Gaun] area, 16 Jul 1998, *W.R*. *Sykes 331/98* (E00649126); Mustang Distr., Tangbe, 28°54'N, 83°49'E, 3065 m a.s.l., 31 Aug 2001, *G. & S. Miehe & Koch 01-098-02* (herb. Miehe); Mustang Distr., Marpha vill., 2600 m a.s.l., 17 Apr 2009, *A. Sukhorukov* s.n. (E, MW).

####### General distribution.

Himalaya, Central Asia, Siberia.

###### 6. Chenopodium bengalense

Taxon classificationPlantaeCaryophyllalesChenopodiacea

(Lam.) Spielm. ex Steud., Nomencl. Bot., ed. 2, 1: 348 (1840)

≡Atriplexbengalensis Lam., Encycl. 1(1): 276 (1783). **Lectotype** (designated by Sukhorukov and Kushunina 2014): “Plante potagére des Indes [vegetable plant from India], herb. De Lamarck” (P-LA00381128!). **Epitype** (designated by Sukhorukov and Kushunina 2014): NEPAL, [Dhaulagiri zone, Myagdi distr.] Annapurna conservation area, near Tikhedhunga vill., 1700 m alt., edge of vegetable garden, 13 Nov 2008, *A. Sukhorukov* s.n. (MW! isoepitype W2010-0007930!). All branches of the epitype and isoepitype are from one individual.
=Chenopodiumgiganteum D.Don, Prodr. Fl. Nepal.: 75 (1825). **Lectotype** (designated by Sukhorukov and Kushunina 2014): Herb. Wallich *6952 F* [N. Wallich] (K!).
=ChenopodiumalbumL.subsp.amaranticolor H.J.Coste & Reyn., Bull. Herb. Boissier, sér. 2, 5: 979 (1905). **Type**: n.v.
≡Chenopodiumamaranticolor (H.J.Coste & Reyn.) H.J.Coste & Reyn., Bull. Soc. Bot. France 54: 181 (1907).

####### Taxonomic notes.

The correct name for this taxon previously known as *Chenopodiumgiganteum* was reinstated by Sukhorukov and Kushunina (2014), but the year of Spielmark’s combination *Chenopodiumbengalense* (Lam.) Spielm. in Steudel (1821) is wrong, because Spielmark considered *Chenopodiumbengalense* (Lam.) Spielm. as a synonym of *Atriplexbengalense* Lam. The combination *Chenopodiumbengalense* is indeed validated in Steudel (1840).

####### Description.

Robust annual up to 2.5 m, branched from the base. Petioles of lower caducous leaves up to 10 cm, blades 10–25(30) × 5–10 cm, usually rhombic, dentate, erose or trilobate with elongated terminal lobe; middle leaves rhombic or ovate, smaller; upper leaves oblong or lanceolate, completely entire, abaxially grey; young leaves often reddish. Inflorescence whitish due to the presence of abundant bladder hairs on the branches and perianth. Fruit 1.2–1.5 mm, pericarp hyaline, adjoining to the seed coat, but separating from it when rubbed, long-papillate in fresh or even dry fruits (Fig. 8D). Seed black, ± keeled, with small crater-like micro-depressions (Fig. 8E, F).

####### Habitat.

Edges of vegetable gardens and other places with secondary vegetation; 700–2900 m a.s.l. Cultivated as a crop but very locally.

####### Phenology.

Flowering: June-September; fruiting: August-November.

####### Distribution.

See Fig. 9.

**Figure 9.**
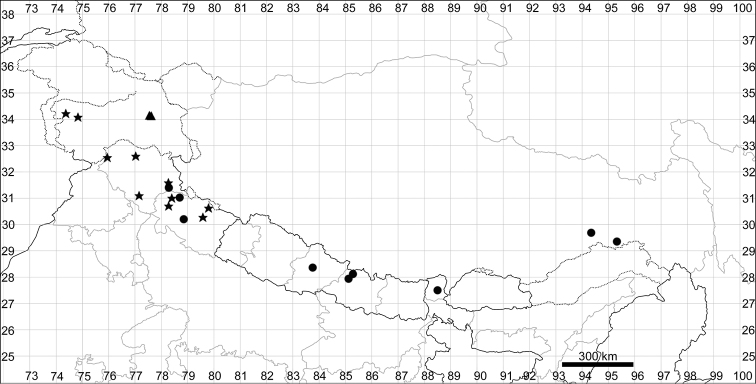
Distribution map of *Chenopodiumbengalense* (circles), *C.novopokrovskyanum* (triangles) and *C.atripliciforme* (stars).

####### Specimens examined.

**CHINA**: **Xizang**: **Nyingchi Prefecture**: Motuo [Mêdog] County, Galasha to Shuina, 1700 m a.s.l., 18 Nov 1982, *Cheng & Li 03053*(PE00510185, PE00510186); Nyingchi (Linzhi) County, Bayi Town, 2950 m a.s.l., 20 Jun 1989, *K.Yao* et al. *1298* (NAS00003754);

**INDIA**: **Himachal Pradesh**: Upper Kinnaur, Poari, 21 Sep 1890, *J.H. Lace 623* (E, G); **Uttarakhand**: Tehri Garhwal [Distr.], Jhala, 8000–9000 ft a.s.l., 25 Jun 1853, *J.F. Duthie* s.n. (DD, G); **Sikkim**: Sikkim Himal [without date and collector] (K);

**NEPAL**: **Western**: **Dhaulagiri Zone**: [Baglung Distr.] nr Lumsum, 8000 ft a.s.l., 10 Sep 1954, *J. Stainton, W.R. Sykes & L.H.J. Williams 4315* (BM; LE); [Myagdi Distr.] between Ramche & Gram, 1800–1900 m a.s.l., 20 Aug 1972, *Kanai* et al. *723625* (BM); [Myagdi Distr.] Annapurna conservation area, near Tikhedhunga vill., 1700 m a.s.l., 13 Nov 2008, *A. Sukhorukov* s.n. (MW, W2010-0007930); **Central**: **Bagmati Zone**: Nuwakot Distr., Bidur, *anonym* s.n. (KATH); [Rasuwa Distr.] Dhunche, 2000 m a.s.l., 22 Aug 1972, *Kanai* et al. *723624* (BM).

####### General distribution.

Himalaya and Tibet and as casual alien in some parts of Eurasia (Uotila 1997; 2001). The specimens from Eastern China labelled as *Chenopodiumgiganteum* (PE, see also Zhu et al. 2003) do not belong to *C.bengalense*. The easternmost records of *C.bengalense* are known from Sichuan, Guizhou, Shensi and Shaanxi provinces in China (PE!).

###### 7. Chenopodium novopokrovskyanum

Taxon classificationPlantaeCaryophyllalesChenopodiacea

(Aellen) Uotila, Ann. Bot. Fenn. 30: 192 (1993)

≡C.albumL.subsp.novopokrovskyanum Aellen, Trudy Rostov. Obl. Biol. Obsch. 2: 3 (1938). **Lectotype** (Uotila 1993): [TURKMENISTAN] Regio Transcaspica, Aschabad, in ruderatis [in dump areas], 25 Jun 1900, *P. Sintenis 527* (S, isolectotypes G-DC! G75415! G75426! K! LD, LE!).

####### Taxonomic notes.

This species was described from the deserts of Turkmenistan and the identity of the populations growing in the Central Asian plains and in the Himalayan Mountains is not obvious despite the morphological similarities. The material from Himalaya is scarce and collected in the blooming or early fruiting stages. Further investigations are needed to establish the taxonomic status of Himalayan plants named *C.novopokrovskyanum*.

####### Description.

**Figure 10.**
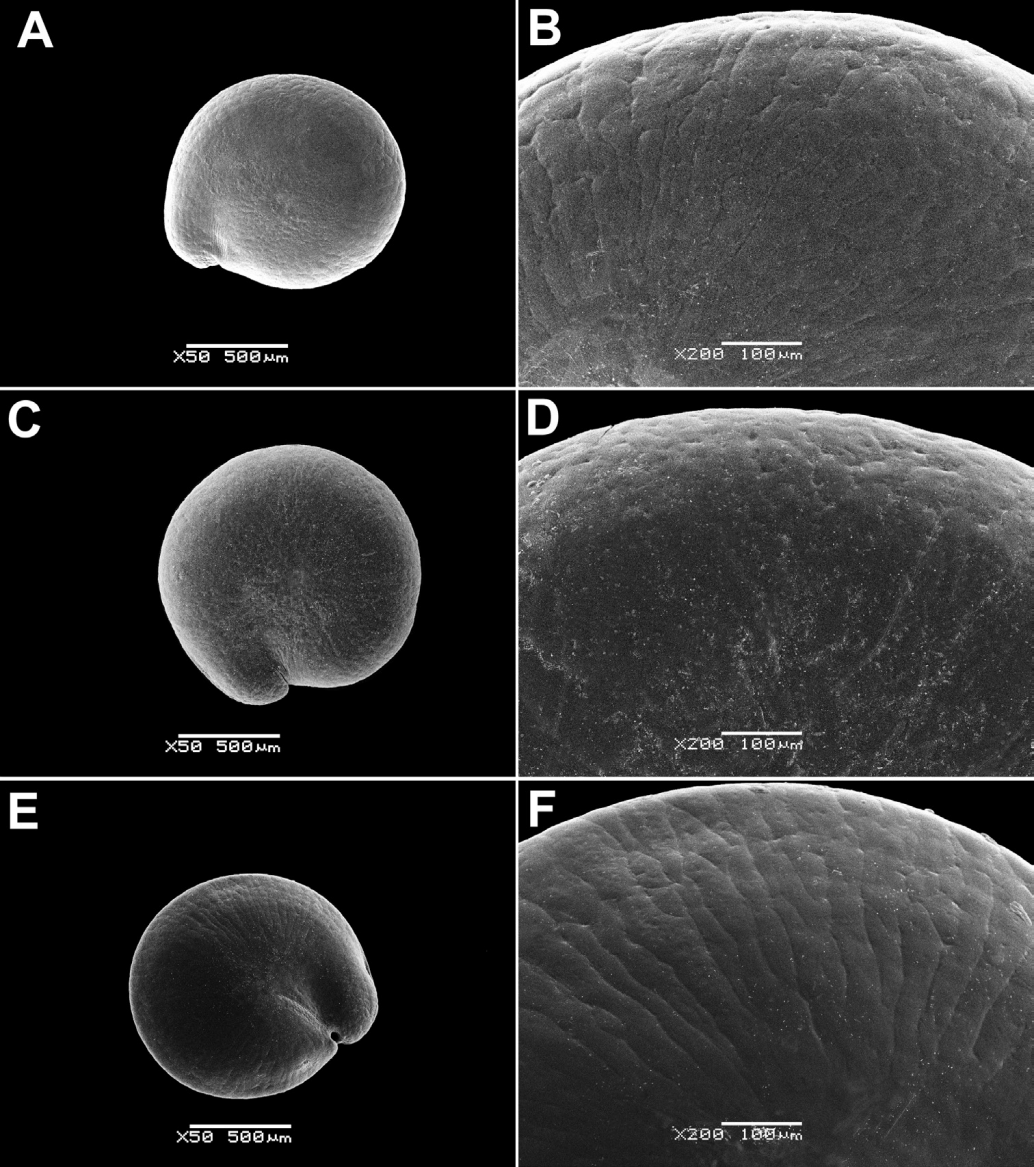
SEM micrographs of *Chenopodium* seeds. **A, B***C.novopokrovskyanum***C, D***C.atripliciforme***E, F***C.perttii*. Magnification: 50× (**A, C, E**), 200× (**B, D, F**).

The species is similar to *C.album*, but the leaves are bi-coloured (green or greyish adaxially and white-farinose abaxially), narrowly oblong to lanceolate. Fruit 1.0–1.1 mm in diameter, ca. 0.6 mm thick, seed almost smooth with indistinctly polygonal ultra-sculpture and indistinct pits (Fig. 10A, B).

####### Phenology.

Flowering: June-September; fruiting: August-October.

####### Distribution.

See Fig. 9.

####### Specimens examined.

**INDIA**: **Jammu & Kashmir**: Ladakh, Leh, 3400 m a.s.l., 20 Aug 1979, *Bruhn* s.n. (MSB144211); Ladakh, Sabu [Saboo village], 3680 m a.s.l., 15 Jul 1992, *H. Hartmann 4013* (MSB137924).

####### General distribution.

SE Europe, Central Asia, North Himalaya, Karakoram.

###### 8. Chenopodium atripliciforme

Taxon classificationPlantaeCaryophyllalesChenopodiacea

Murr, Magyar Bot. Lapok 1: 360 (1902).

####### Lectotype

(Uotila 1993): AFGHANISTAN, Kurrum valley, 1879, *J.E.T. Aitchinson 980* (G11875! isolectotypes BM000629112! C, G-BOISS! FI, K! S).

####### Taxonomic notes.

Murr (1902) cited two distant records (Russian Far East and Afghanistan) in the protologue of *C.atripliciforme*. We agree with Uotila (1993) that the specimen from the first location (Ussuri) probably belongs to *C.bryoniifolium* Bunge, which is similar in leaf shape to *C.atripliciforme*.

####### Description.

Annual up to 1 m, mostly branched; leaves petiolate (petioles up to 4 cm). Leaf blades triangular, basally truncate or broadly cuneate, entire to erose-dentate. Inflorescence branched, terminal and axillar, slender; clusters consisting of 2–4 flowers or rarely flowers solitary. Fruit 1.2–1.4 mm with papillate pericarp. Seed black, almost smooth with indistinct small pits (Fig. 10C, D).

####### Habitat.

Rocky places, forest margins; (1800)2000–3300 m a.s.l.

####### Phenology.

Flowering: June-September; fruiting: August-October.

####### Distribution.

See Fig. 9.

####### Specimens examined.

**INDIA**: **Jammu & Kashmir**: Baramulla, Aug 1880, *Young* s.n. (BM); Sind valley, 7500 ft a.s.l., 2 Sep 1876, *C.B*. *Clarke 30934* (LE); Srinagar, in monte Shankaracharya, 1770–2000 m a.s.l., 24 Aug 1986, *K.H. Rechinger 62146* (W1998-03383, WU021382); **Himachal Pradesh**: Simla, Aug 1849, *A. Fleming* s.n. (E); Dalhousie, 20 Sep 1874, *C.B*. *Clarke 22777* (K); Simla, 7000 ft a.s.l., 1 Sep 1877, *J.S. Gamble 4909* (K); Lahaul, Kyelang, 3100 m a.s.l., 11 Aug 1970, *U.C. Bhattacharyya 40754* (BSD); [Kinnaur Distr.] Purbani, 2500 m a.s.l., 7 Oct 1971, *K.P. Janardhanan 46510 & 46519* (BSD); **Uttarakhand**: Garhwal, Jumma area, 2800 m a.s.l., 14 Aug 1974, *B.D. Nailhani 53892* (BSD); [Uttarkashi Distr.] Kalyani, 3000 m a.s.l., 13 Aug 1978, *A.K. Goel 64457* (BSD); Chamoli Distr., Aug 1988, *P.K. Hajra 87078* (BSD); Uttarkashi Distr., Janki Chatti, 7 Oct 1993, *S.C. Majumdar & S. Singh 88100* (BSD).

####### General distribution.

East Afghanistan, North Pakistan, North India (Jammu & Kashmir, Himachal Pradesh, Uttarakhand and Punjab).

###### 9. Chenopodium perttii

Taxon classificationPlantaeCaryophyllalesChenopodiacea

Sukhor., Phytotaxa 191(1): 21 (2014)

####### Holotype.

NEPAL, [Dhaulagiri Zone] Mustang [Distr.], 13000 ft alt., at edge of field, 3 Aug 1954, *J. Stainton, W.R. Sykes & L.H.J. Williams 2159* (BM000832654! isolectotype LE!).

####### Taxonomic notes.

Morphologically, *Chenopodiumperttii* resembles a Far Eastern and Siberian species, *C.bryoniifolium* Bunge, but the species have different distributions and reproductive characters (Sukhorukov and Kushunina 2014). The recently identified chromosome number for *C.perttii* is 54, so it is genetically distant from other species previously identified as *C.album* (Bohumil Mandak, pers. comm.). However, we assume that *C.perttii* is more closely related to Chenopodiumalbumsubsp.yunnanense Aellen in Handel-Mazzetti (1929). This subspecies was described from Yunnan (China) and differs by the absence of the seed keel. The lectotype of Chenopodiumalbumsubsp.yunnanense was selected in Sukhorukov and Kushunina (2014).

####### Description.

Annual up to 1(1.5–2) m with erect, scarcely branched stem. Leaves dark green, petiolate (petioles up to 3 cm), blades 3–4 × 2–3 cm, base truncate or broadly cuneate, margins often red; lower leaves triangular, entire to lobate; middle leaves trilobate with elongated mid-lobe (less than two times as long as the lateral ones) gradually tapering to apex and slightly upward-directed lateral lobes, lobes mostly entire; upper leaves also trilobate or entire, narrowly oblong. Inflorescence green, aphyllous or rather bracteose. Fruit ~1.5 mm, pericarp separating from the seed (not easily) with minute papillae that are hardly restored after soaking, almost smooth in dry fruit. Seed prominently keeled, surface with radial striae (Fig. 10E, F).

####### Habitat.

Disturbed places, often as a weed in crop fields; 2400–4500 m a.s.l.

####### Phenology.

Flowering: June-September; fruiting: August-October.

####### Distribution.

See Fig. 11.

**Figure 11.**
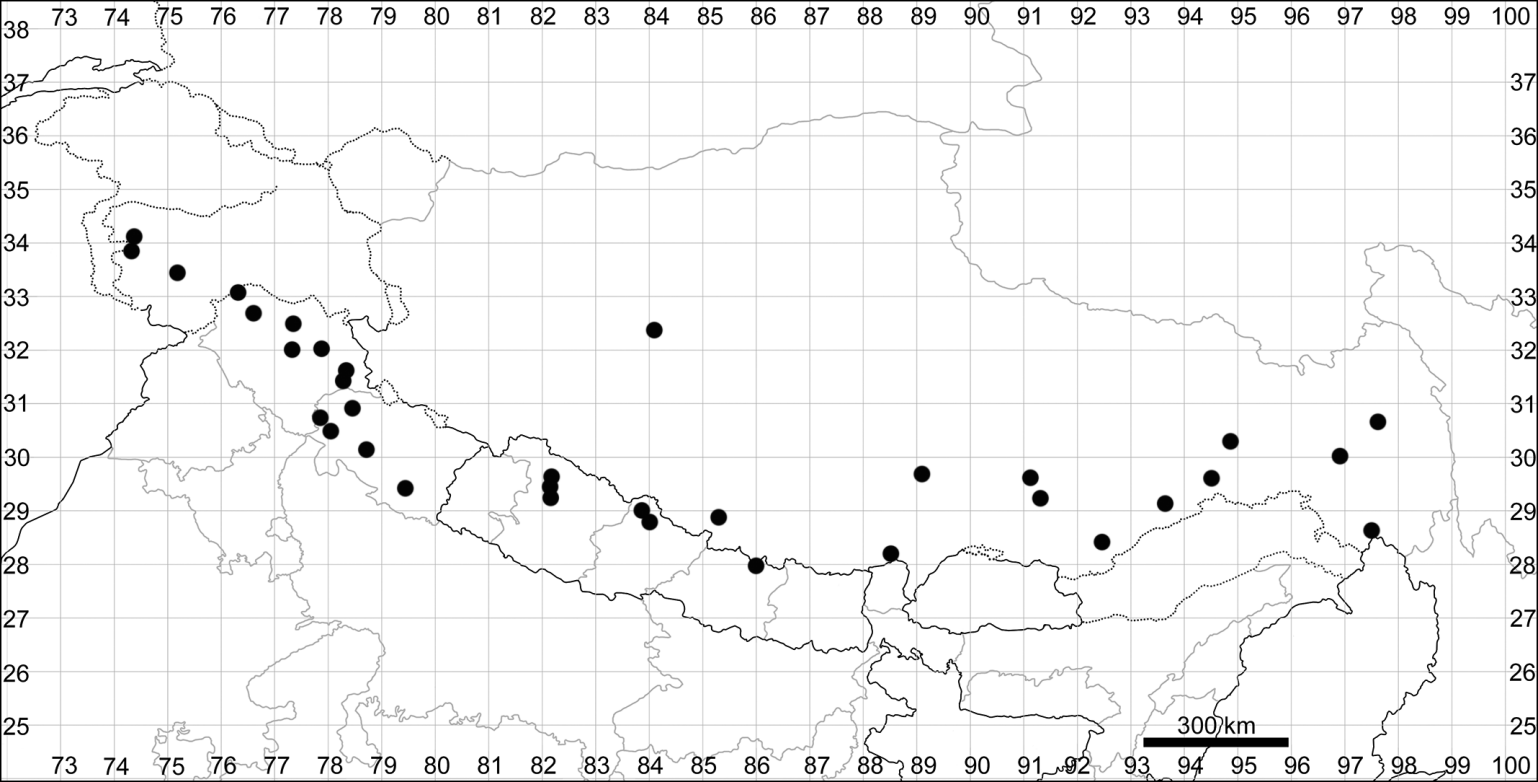
Distribution map of *Chenopodiumperttii*.

####### Specimens examined.

**CHINA**: **Xizang**: **Ngari Prefecture**: Gêrzê County, 4250 m a.s.l., Aug 1972, *Li 67* (PE00510178); **Xigazê Prefecture**: Nyalam (Nielamu) County, Zham (Zhangmu), 13 May 1966, *Zhang & Lang 3361* (PE00510187); Gyirong (Jilong) County, Gyirong (Jilong) Town, 2900 m a.s.l., 6 Jun 1972, *Tibet Chinese Herbal Medicine Survey Team 151* (PE00510191, PE00510220); Namling (Nanmulin) County, Qunmo, 4000 m a.s.l., 2 Aug 1972, *Tibet Chinese Herbal Medicine Survey Team 912* (PE00510199, PE00510219); Namling (Nanmulin) County, Gangba, 4000 m a.s.l., 3 Aug 1972, *Tibet Chinese Herbal Medicine Survey Team 993* (PE00510221); **Lhasa City**: nr Lhasa, July 1939, *H.E. Richardson 215* (BM); Lhasa, 3800 m a.s.l., 16 Aug 1965, *Zhang & Lang 1532* (PE00510965); [Damxung (Dangxiong) County] Lake Natso, 30°55'N, 90°59'E, 4740 m a.s.l., 4 Sep 2005, *Nölling & Hanspach NX05-201-10* (herb. Miehe); **Shannan Prefecture**: Zhanang (Chatang) County, 3500 m a.s.l., 13 Aug 1960, *Fu 528* (PE00510203); Lhünzê (Longzi) County, 3800 m a.s.l., 17 Aug 1960, *Fu 564* (PE00510209); **Nyingchi Prefecture**: [Mainling (Milin) County] Tsangpo valley, Timpa, 29°33'N, 94°52'E, 9700 ft a.s.l., 6 Jul 1938, *F. Ludlow* et al. *5157* (E); [Bomi County], Yi’ong (Yigong), 2300 m a.s.l., 15 Jul 1965, *Ying & Hong 650553* (PE00510158, PE00510159, PE00510160, PE00510161); Nyingchi (Linzhi) County, Deying Distr., 2950 m a.s.l., 17 Jun 1972, *Tibet Chinese Herbal Medicine Survey Team 3330* (PE00510163, PE00510222); Mainling (Milin) County, Jiage, 3100 m a.s.l., 10 Jul 1972, *Tibet Chinese Herbal Medicine Survey Team 3660* (PE00510162); Zayü (Chayu) County, 1700 m a.s.l., 4 Jul 1980, *Ni* et al. *0420* (PE00510183); Zayü (Chayu) County, 2600 m a.s.l., 13 Aug 1980, *Ni* et al. *1026* (PE00510182); **Qamdo Prefecture**: Baxoi (Basu) County, Rawu (Ranwu) to Baima, 4200 m a.s.l., 27 Aug 1973, *Qinghai-Tibet Team 73-1279* (PE00510176, PE00510177); Zhag’yab County, 3100 m a.s.l., 7 Aug 1976, *Qinghai-Tibet Team Vegetation Group 9492* (PE00235126);

**INDIA**. **Jammu & Kashmir**, Tangmarg, 6000 ft a.s.l., 17 Aug 1956, *O. Polunin 56-364* (E); Banihal river, 3300–3835 m a.s.l., 14 Sep 1958, *T.A. Rao 7665* (BSD); Loran, 2000–2500 m a.s.l., 17 Sep 1985, *Vohra 78338* (BSD); Raydha La, 18 Sep 1986, *B.M. Wadhwa 83855* (BSD); **Himachal Pradesh**: [Kinnaur Distr.] Kalpa, 2775 m a.s.l., 3 Jun 1962, *N.C. Nair 22343* (BSD); Chamba Distr., Kilar, 2625 m a.s.l., 19 Jul 1964, *N.C. Nair 32634* (BSD); [Kinnaur Distr.] Sangla, 1950 m a.s.l., 20 Sep 1964, *N.C. Nair 33027* (BSD); Lahaul, Chimrat, 2900 m a.s.l., 2 Aug 1990, *McBeath 2275* (E); Kulu Distr., Kasol, 1667 m a.s.l., Sep 2000, *S. Singh* s.n. (BSD); Lahaul, 3000 m a.s.l., Aug 2000, *S. Singh 144* (BSD); Spiti Distr., Pin Valley National Park, 3800 m a.s.l., 3 Sep 2002, *K.C. Sekar 102975* (BSD); **Uttarakhand**: Kumaon, Nainital, 2100–2500 m a.s.l., 8 Oct 1957, *T.A. Rao 4881* (BSD); Mussoorie, 2320 m a.s.l., 6 Aug 1960, *anonym 12470* (BSD); Chakrata, 6750 ft a.s.l., 29 Jul 1961, *U.C. Bhattacharyya 13069* (BSD); Pauri Garhwal Distr., Pauri, 21 Sep 1979, *A.S. Rao 56423* (BSD); Uttarkashi distr., Janki Chatti, 4 Oct 1993, *S.C*. *Majumdar & S. Singh 86486* (BSD);

**NEPAL**: **Midwestern**: **Karnali Zone**: [Jumla Distr.] Jumla vill., 2400 m a.s.l., 27 Sep 2010, *A. Sukhorukov 567* (MW); Jumla vill., 29 Sep 2014, *A. Sukhorukov 62* (MW); Mugu Distr., Jhari vill., 2600 m a.s.l., 4 Oct 2014, *A. Sukhorukov 510* (MW); Mugu Distr., Gamghadi vill., 5 Oct 2014, *A. Sukhorukov* s.n. (G); Mugu Distr., Pina vill., 6 Oct 2014, *A. Sukhorukov 585* (BR); **Western**: **Dhaulagiri Zone**: [Mustang Distr.] Thinigaon, Muktinath Himal, 11500 ft a.s.l., *J. Stainton, W.R. Sykes & L.H.J. Williams 1372* (LE); [Upper Mustang] Tange, 3500 m a.s.l., 22 Aug 1955, *Lobbichler 26* (M); Mustang Distr., Tangbe, 28°54'N, 83°49'E, 3065 m a.s.l., 31 Aug 2001, *G. & S. Miehe & Koch 01-098-05* (herb. Miehe); **Gandaki zone**: Manangbhot Distr., Tilicho base camp (4000 m a.s.l.) to Khangsar (3650 m a.s.l.), 1 Aug 1983, *H. Ohba* et al. *8331076* (E00238402).

####### General distribution.

Himalaya and Tibet. Frequent or common in West Nepal.

##### Poorly known species

###### Chenopodium strictum

Taxon classificationPlantaeCaryophyllalesChenopodiacea

Roth, Nov. Sp. Pl.: 180 (1821)

####### Type.

n.v. (not present in HAL and other herbaria).

This name was previously widely used for the plants growing in Europe, Siberia, Iran and Central Asia (e.g. Iljin 1936, Kinzikaeva 1968, Lomonosova 1992, Uotila 1997). Sukhorukov (2014) discovered that the plants growing in North Himalaya and in Europe differ in their morphological characters and *C.betaceum* Andrz. may be the correct name for the European plants. Furthermore, *C.strictum* material from Himalaya, from where the species was described, is scarce (Jammu and Kashmir, India). Further investigations are needed to discover which plants belong under this name.

###### Chenopodium hookerianum

Taxon classificationPlantaeCaryophyllalesChenopodiacea

Moq. in DC., Prodr. 13(2): 68 (1849)

####### Notes.

The species was described from Nepal, ex herb. Hooker (P00606414) (see link to the specimen https://science.mnhn.fr/institution/mnhn/collection/p/item/p00606414) and the type material bears a plant fragment collected at the flowering stage. This name has never been mentioned in the recent floristic and taxonomic accounts except the Flora of the USSR (Iljin 1936), where it was synonymised with *Chenopodiumgiganteum* (=*C.bengalense*). However, the protologue of C. *hookerianum* (Moquin-Tandon 1849) and a fragment of the type specimen do not demonstrate the features of the species described by Moquin well (plant height, seed characters etc.). We are still not sure how to treat *C.hookerianum*.

###### Chenopodium patulum

Taxon classificationPlantaeCaryophyllalesChenopodiacea

Roth, Nov. Pl. Sp.: 180 (1821)

####### Notes.

The species was described from “India Orientalis” [East India] without a precise location. The type material was not found in any herbaria including HAL. The name is a later homonym of *C.patulum* Mérat described from France (Mérat 1812) and cannot be used according to ICN (Art. 53.1).

###### 2. Chenopodiastrum

Taxon classificationPlantaeCaryophyllalesChenopodiacea

S.Fuentes, Uotila & Borsch, Willdenowia 42(1): 14 (2012)

Type: Chenopodiastrummurale (L.) S.Fuentes, Uotila & Borsch.

####### Description.

Annuals, glabrous or covered with bladder hairs. Leaves triangular or rhombic, petiolate, entire, dentate or lobate, rarely pinnatisect. Inflorescences spreading, mostly leafless, often with scattered yellowish glandular hairs. Flowers bisexual or female with 5 perianth segments that do not change at the fruiting stage. Perianth segments free or connate to the half of their length; midvein clearly elevated adaxially. Stamens 5. Stigmas 2. Fruit 1.2–2.5 mm in diameter; pericarp 1–2-layered with conical or cylindrical papillae, forming alveolate (in fresh fruits) or reticulate (in dry fruits) surface. Seed black, with keel or not, alveolate or punctate, sometimes with deep combs; seed-coat testa with vertical or obliquely orientated stalactites. Latent structural heterospermy expressed as varying testa thickness found in some species; seed embryo horizontal.

At least 10 species in all continents.

##### Key to the species

**Table:** 

1	Leaf blade cuneate, dentate to erose-dentate; seed 1.3–1.4 mm in diameter	**1. *C.murale***
–	Leaf blade truncate or slightly cordate, entire or with a few lobelike teeth; seed 1.5–2.0 mm in diameter	**2. *C.badachschanicum***

###### 1. Chenopodiastrum murale

Taxon classificationPlantaeCaryophyllalesChenopodiacea

(L.) S.Fuentes, Uotila & Borsch, Willdenowia 42(1): 14 (2012)

Fig. 12

≡Chenopodiummurale L., Sp. Pl.: 219 (1753). **Lectotype** (designated by Brenan 1954): Herb. Linn. 313.6 (LINN! image available at http://linnean-online.org/3080/).
=Chenopodiumilicifolium Griff., Not. Pl. Asiat. 4: 337 & Tab. 521 (1854). Described from Shikarpur, Pakistan (type n.v.).

####### Taxonomic notes.

*Chenopodiumilicifolium* was previously synonymised with *Chenopodiumbotrys* (≡*Dysphaniabotrys*); see Press et al. (2000). Despite the absence of the authentic herbarium, we merge *Chenopodiumilicifolium* with *Chenopodiastrummurale* for the following reasons: (1) the drawings of *C.ilicifolium* (Griffith 1854) show the plant with dentate leaves, a short and leafy inflorescence, closed flowers and fruit with a papillate pericarp. These traits are characteristic of *C.murale*. Additionally, the city of Shikarpur is located on the plains where other *Chenopodiastrum* do not occur. The merger of *Chenopodiumilicifolium* with *Chenopodiastrummurale* is consistent with the opinion of Singh (2014, sub *Chenopodiummurale*).

####### Description.

Annual, 10–50 cm, erect, with several lateral branches, slightly mealy. Leaves 2.0–6.0 × 1.0–5.0 cm, dark green, broadly deltoid-ovate, upper leaves narrow and long-acuminate, mucronulate, cuneate, coarsely and irregularly dentate, rarely subentire or erose-dentate; teeth acute and ± incurved. Inflorescences terminal and axillary (leafy) with loosely branched cymes. Perianth segments slightly keeled with a distinct swelling below the apex. Fruit 1.3–1.4 mm, pericarp tightly adjoining the seed, papillate. Seed with prominent keel and surface densely covered with small pits (Fig. 13A, B).

**Figure 12.**
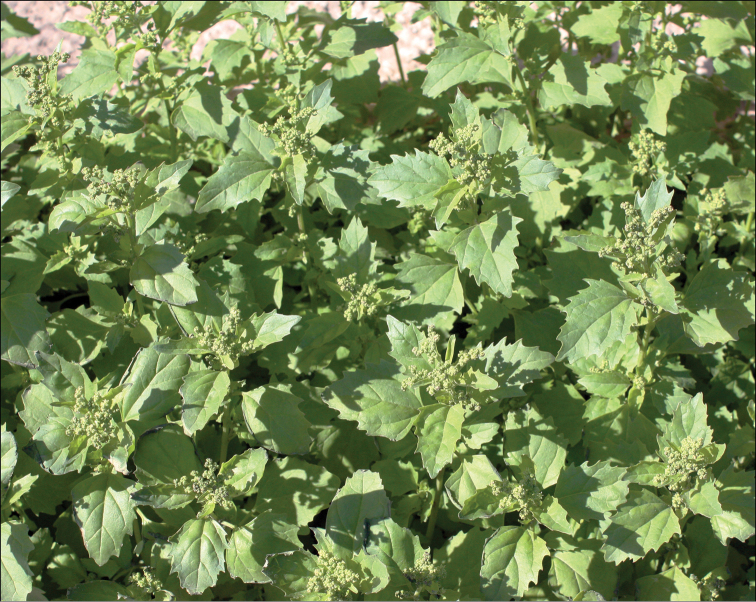
*Chenopodiastrummurale* plants at blooming stage. Photograph by A. Sukhorukov (Dead Sea area, Israel, February 2011).

**Figure 13.**
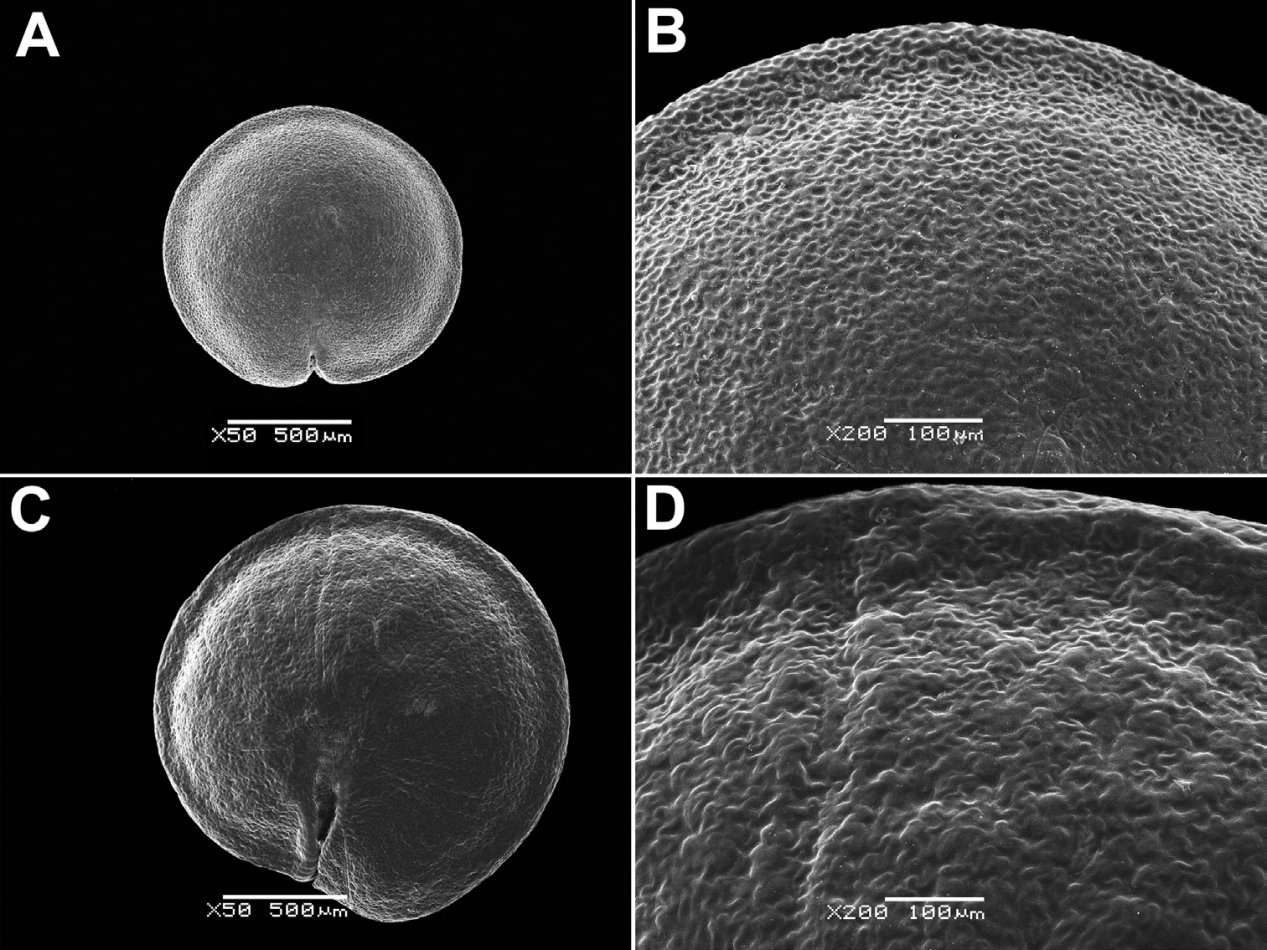
SEM micrographs of *Chenopodiastrummurale* (**A, B**) and *C.badachschanicum* (**C, D**) seeds. Magnification: 50× (**A, C**), 200× (**B, D**).

####### Habitat.

Disturbed places; up to 2000 m a.s.l. Reported as a common species in the foothills of Chamba District, Himachal Pradesh (Singh and Sharma 2006).

####### Phenology.

Flowering and fruiting: December-April (in the plains), March-September in the foothills.

####### Distribution.

See Fig. 14.

**Figure 14.**
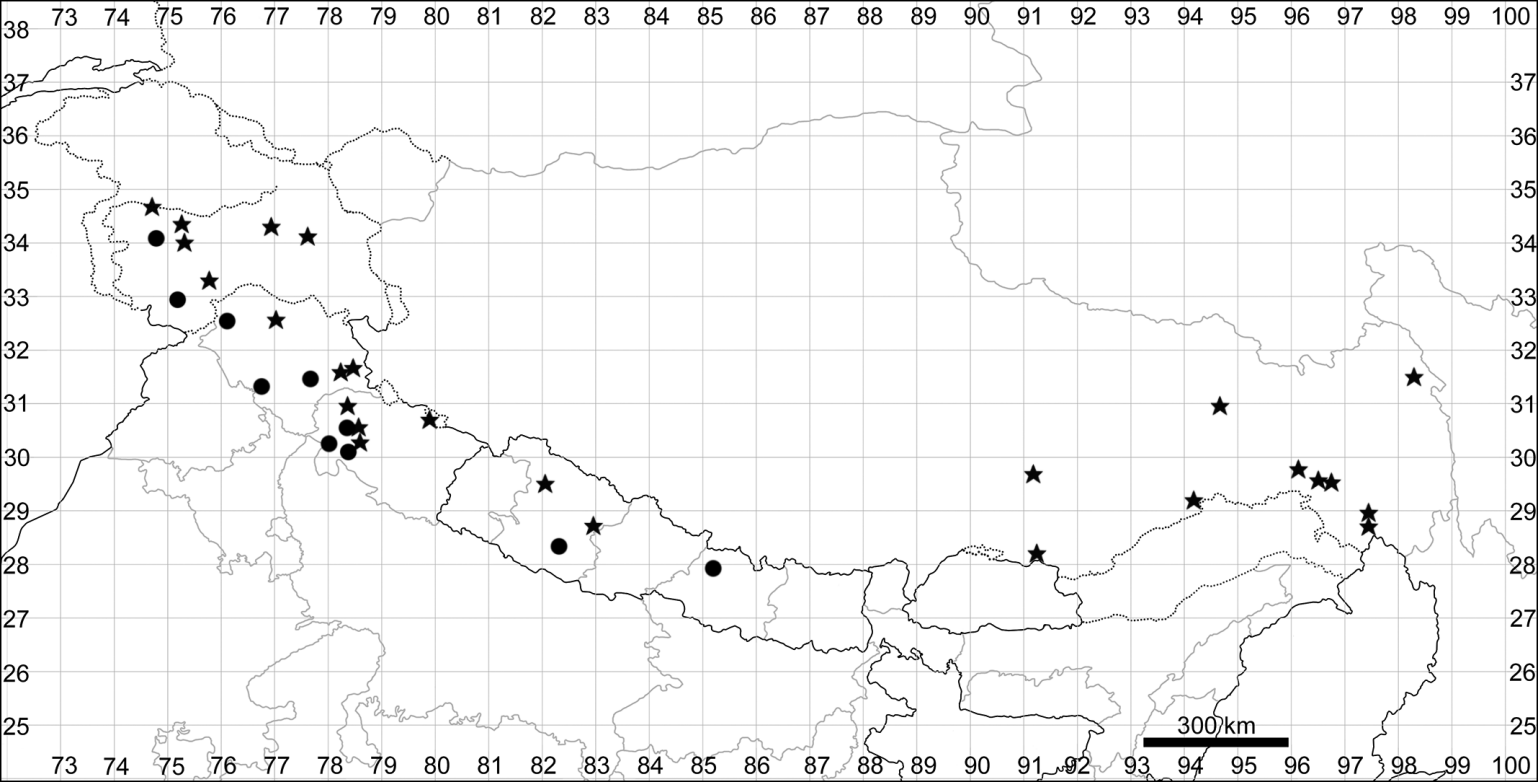
Distribution map of *Chenopodiastrummurale* (circles) and *C.badachschanicum* (stars).

####### Specimens examined.

**INDIA: Jammu & Kashmir**: [without locality and date] *Jacquemont* s.n. (P04993274); Udhampur, 3 Apr 1987, *A. Swami 1089* (BSD); **Himachal Pradesh**: Bilaspur Distr., [without date] *P. Lai 78966* (BSD); [Simla Distr.] Rampur, 14 Jun 1962, *N.C. Nair 22677* (BSD); **Uttarakhand**: Dehradun, 23 May 1972, *H. Naithani 3510* (DD); Garhwal Distr., Jogiyna vill., 19 Feb 1979, *A.K. Goel 64713* (BSD); Tehri Garhwal Distr., Chham vill., 10 Apr 1993, *B.P. Uniyal 79275* (BSD);

**NEPAL**: **Midwestern**: **Rapti Zone**: [Rolpa Distr.] Phalabang, 4500 ft a.s.l., 24 Mar 1952, *O. Polunin, W.R. Sykes & L.H.J. Williams 654* (BM); observed by Sukhorukov in Kathmandu valley and near Bidur village (Bagmati Zone).

####### General distribution.

Widely distributed in tropics and subtropics in all parts of the world. Origin unknown, but Sukhorukov (2014) proposed that it may be Eastern Africa and Arabia, where the morphologically similar *C.fasciculosum* (Aellen) Mosyakin occurs. *Chenopodiastrummurale* is rather common in the plains of India, but there are limited collections from the Himalayan foothills. The species was not observed by AS in Dehradun (Uttarakhand, India) and surroundings in February 2018.

###### 2. Chenopodiastrum badachschanicum

Taxon classificationPlantaeCaryophyllalesChenopodiacea

(Tzvelev) S.Fuentes, Uotila & Borsch, Willdenowia 42(1): 14 (2012)

≡Chenopodiumbadachschanicum Tzvelev, Bot. Mater. Gerb. Bot. Inst. Komarova Acad. Nauk SSSR 20: 434 (1960).

####### Holotype.

TAJIKISTAN, Gorno-Badakhshan Autonomous province, West Pamir, in declivitate lapidosa paulo ruderata in valle fl. Murgab 3–4 km infra ostium fl. Pschart occidentalis, 3300 m alt., 19 June 1958, *N.N*. *Tzvelev 220* (LE, currently on loan in H!).

####### Taxonomic notes.

The “true” *Chenopodiastrumhybridum* reported for some territories (e.g. Chowdhery 1984, Zhu et al. 2003) is not present in Himalaya and Tibet. Additionally, we are uncertain that *C.badachschanicum* described from the Pamir Mountains (Tzvelev 1960) is the correct name for our populations. As recently recognised (Sukhorukov in prep.), the seed surface plays a significant role in the diagnostics of many species of the genus. The type material of *C.badachschanicum* bears small plants (up to 25 cm) with acutely lobed leaves but without ripe seeds. The similar individuals at the fruiting stage were collected in East Kazakhstan, Kyrgyzstan and Tajikistan and their fruits have adherent pericarp and clearly pitted seeds (like those of *C.hybridum*). The plants growing in Himalaya and Tibet are quite uniform in their morphological characters and possess the fruit with loose pericarp and rugulose seed surface. *C.badachschanicum* complex needs further investigations.

####### Description

(based on the specimens growing in our area): Annual up to 100 cm, almost glabrous or only slightly mealy in the upper part. Leaf axils with numerous fertile or sterile brachyblasts. Leaves long-petiolate, continuously shortening upwards; petioles 3.0–10.0 cm; blades 4.0–10.0 cm long, triangular-hastate, entire or with 1–2 unequal lateral lobes in lower part of the leaves, apex attenuate (similar to the leaves of *Atriplexmicrantha* C.A.Mey.). Inflorescence leafy or bracteose, consisting of short lateral branches with scattered yellow glands. Perianth segments halfway connate, slightly keeled in the upper part or smooth; margins in the upper part of the segments covered with bladder hairs intermixed with scattered simple hairs. Fruit 1.3–1.8 mm (usually 1.4–1.7 mm) long, 0.9–1.1 mm thick, pericarp brownish, easily ruptured at maturity, slightly alveolate or smooth in dry fruits (with tiny papillae in fresh or soaked fruits). Seed depressed globular, black, keeled, shiny, rugulose, with sinuous surface as well as scattered indistinct longitudinal striae with shallow pits (Fig. 13C, D).

####### Habitat.

Rocks and screes, meadows; margins of spruce and pine forests; 2000–4400 m a.s.l. The collectors also indicate that the species often grows on mossy substrates.

####### Phenology.

Flowering: July-September; fruiting: August-October.

####### Distribution.

See Fig. 14.

####### Specimens examined.

**CHINA: Xizang**: Kham (Tibet), Yangtse river basin, 12000 ft a.s.l., 31 Aug 1900, *Ladygin 515* (LE); **Lhasa City**: Lhasa, 3760 m a.s.l., 28 Jun 1960, *Fu 286* (PE00511716); Lhasa, 3680 m a.s.l., 27 Aug 1965, *Zhang & Lang 2164* (PE00511521); Lhasa, 12000 ft a.s.l., [without date and collector] *20298* (DD); **Shannan Prefecture**: Lhozhag (Luozha) County, 3718 m a.s.l., 28°10'48.91"N, 91°14'57.28"E, [without date], *Y.S.Chen* et al. *13-1348* (PE01992193); **Nyingchi Prefecture**: Bomi County, Sumzom (Songzong), 3500 m a.s.l., 5 Sep 1965, *Ying & Hong 651140* (PE00511513); Mainling (Milin) County, 3100 m a.s.l., 26 Jul 1972, *Tibet Chinese Herbal Medicine Survey Team 4174* (PE00511517); Zayü (Chayu) County, 2000 m a.s.l., 1 Jul 1980, *Ni* et al. *0328* (PE00511511); Zayü (Chayu) County, 2551 m a.s.l., 28°51'38.79"N, 97°28'49.11"E, 14 Sep 2012, *FLPH Tibet Expedition 12-0800* (PE01967926); Zayü (Chayu) County, 14 Sep 2012, *FLPH Tibet Expedition 12-1172* (PE01967978); **Qamdo Prefecture**: [Baxoi (Basu) County] north of Rawu (Ranwu) Town, 4000 m a.s.l., 29 Aug 1976, *Wu* et al. *5146* (KUN 0587309); Banbar (Bianba) County, 3700–3900 m a.s.l., 8 Sep 1976, *Qinghai-Tibet Team 11223* (PE00511522); Jomda (Jiangda) County, E of Jomda (Jiangda), 31°28'59"N, 98°16'39"E, 3900 m a.s.l., 4 Aug 2009, *D.E. Boufford* et al. *41918* & *41349* (MSB156625, P05158929, PE01899404); Baxoi (Basu) County, 29°31'52.3"N, 96°33'7.6"E, 3781 m a.s.l., 12 Aug 2011, *Yu* et al. *5326* (PE);

**INDIA**: **Jammu & Kashmir**: Wandla to Kalotse [Wanla to Khaltse], 12000 ft a.s.l., 18 Aug 1847, *T.T. Thomson* s.n. (K); Leh, Jul 1848, *Thomson* s.n. (K); Sonamarg, 1 Sep 1876, *C.B*. *Clarke 30899* (K); Gurais valley, 22 Sep 1893, *J.F. Duthie 14083* (LE, WU021390); Pahalgam, 8000 ft a.s.l., 24 Aug 1922, *R.R*. *Stewart* s.n. (K); Kistawar, 22 Sep 1958, *T.A. Rao 7870* (BSD); **Himachal Pradesh**: Kinnaur Distr., 3000 m a.s.l., 31 Aug 1963, *N.C. Nair 30282* (BSD); Kinnaur Distr., Purbani vill., 2500 m a.s.l., 7 Oct 1971, *K.P. Janardhanan 46511* (BSD); **Uttarakhand**: Tehri Garhwal [Distr.], 10000 ft a.s.l., Jul 1883, *J.F. Duthie 130* (K); Lahaul, Kyelang, 3200 m a.s.l., 13 Aug 1970, *U.C. Bhattacharyya 40786* (BSD); Garhwal, 2600 m a.s.l., 15 Aug 1974, *B.D. Naithani 54038* (BSD); Chamoli Distr., Malari, 2 Sep 1975, *B.D. Naithani 55992* (BSD); Uttarkashi Distr., Lanka vill., 3000 m a.s.l., 29 Aug 1983, *U.C. Bhattacharyya 74604* (BSD);

**NEPAL**: **Midwestern**: **Karnali Zone**: [Mugu Distr.] Purana Mugu, Mugu Khola, 13000 ft a.s.l., 23 Aug 1952, *O. Polunin, W.R. Sykes & L.H.J. Williams 3010* (LE); **Rapti Zone**: [Rukum Distr., nr Ranmamaikot] Seng Khola, 13000 ft a.s.l., 2 Oct 1954, *J. Stainton, W.R. Sykes & L.H.J. Williams 4674* (LE); reported for the Mustang District (Yonekura 2008).

####### General distribution.

Himalaya and Tibet: in Nepal, parts of India (Jammu and Kashmir and Uttarakhand), China (Qinghai, Sichuan, Xinjiang, Xizang and Yunnan) and Pakistan (Swat and Quetta). Also present in South Siberia (Altai Mountains).

###### 3. Oxybasis

Taxon classificationPlantaeCaryophyllalesChenopodiacea

Kar. & Kir., Bull. Soc. Imp. Nat. Moscou [14]: 738 (1841)

Type: Oxybasisminutiflora Kar. & Kir., Bull. Soc. Imp. Nat. Moscou [14]: 739 (1841) [=O.chenopodioides (L.) S.Fuentes, Uotila & Borsch, Willdenowia 42(1): 15 (2012)].

####### Description.

Annuals, branched from the base or with a single stem, prostrate or erect, glabrous or covered with bladder hairs. Leaves alternate, entire to lobate, rhombic, triangular or oblong, sometimes lanceolate, green from both sides or whitish abaxially. Inflorescence racemiform with lateral branches mostly appressed to the stem; flowers arranged in dense glomerules. Perianth of 3–5 free or diversely connate, hyaline or greenish segments (both perianth forms are present in some species), keeled or not. Flowers bisexual or sometimes female (lateral flowers). Stamens 2–5. Stylodia 2. Pericarp thin, smooth, mamillate or rarely papillate. Seeds usually small (up to 1.2 mm in diameter), red or black. Embryo horizontal or vertical and both embryo positions may be present in one individual (spatial heterospermy). Structural (latent) heterospermy expressed in the varying thickness of the seed coat is common in almost all representatives; outer cell wall of the testa cells with stalactites.

Twelve to fourteen species in the temperate and mountainous parts of Eurasia, Africa and the Americas. One species in Himalaya and Tibet.

###### 1. Oxybasis glauca

Taxon classificationPlantaeCaryophyllalesChenopodiacea

(L.) S.Fuentes, Uotila & Borsch, Willdenowia 42(1): 15 (2012)

≡Chenopodiumglaucum L., Sp. Pl.: 220 (1753). **Lectotype** (designated by Uotila 1993): Herb. Linn. 313.17 (LINN! image of the lectotype available at http://linnean-online.org/3142/).
≡Botrysglauca (L.) Nieuwl., Am. Midl. Naturalist 3: 275 (1914).

####### Description.

Annual up to 70 cm, branched from the base, sometimes forming cushion-like habit at high elevations; stem often prostrate to ascending, rarely straight. Leaves up to 6 × 2 cm, petiolate, cuneate at base, oblong or lanceolate, dentate or lobate with 2–5 lobes, rarely entire, green adaxially, grey or whitish below. Inflorescence leafy, loose. Perianth segments 3 to 5, almost free with hyaline margins, keeled along midrib, opened at fruiting stage. Fruit 0.65–0.8 mm in diameter, pericarp smooth, whitish, often ruptured. Seed reddish, without keel; embryo in both vertical and horizontal positions (spatial heterospermy), structural (latent) heterospermy expressed in different thickness of seed-coat testa.

####### Habitat.

Rocks, streams or other wet (often sandy) places; 2800–4600 m a.s.l.

####### Phenology.

Flowering: July-September; fruiting: August-October.

####### Distribution.

See Fig. 15.

**Figure 15.**
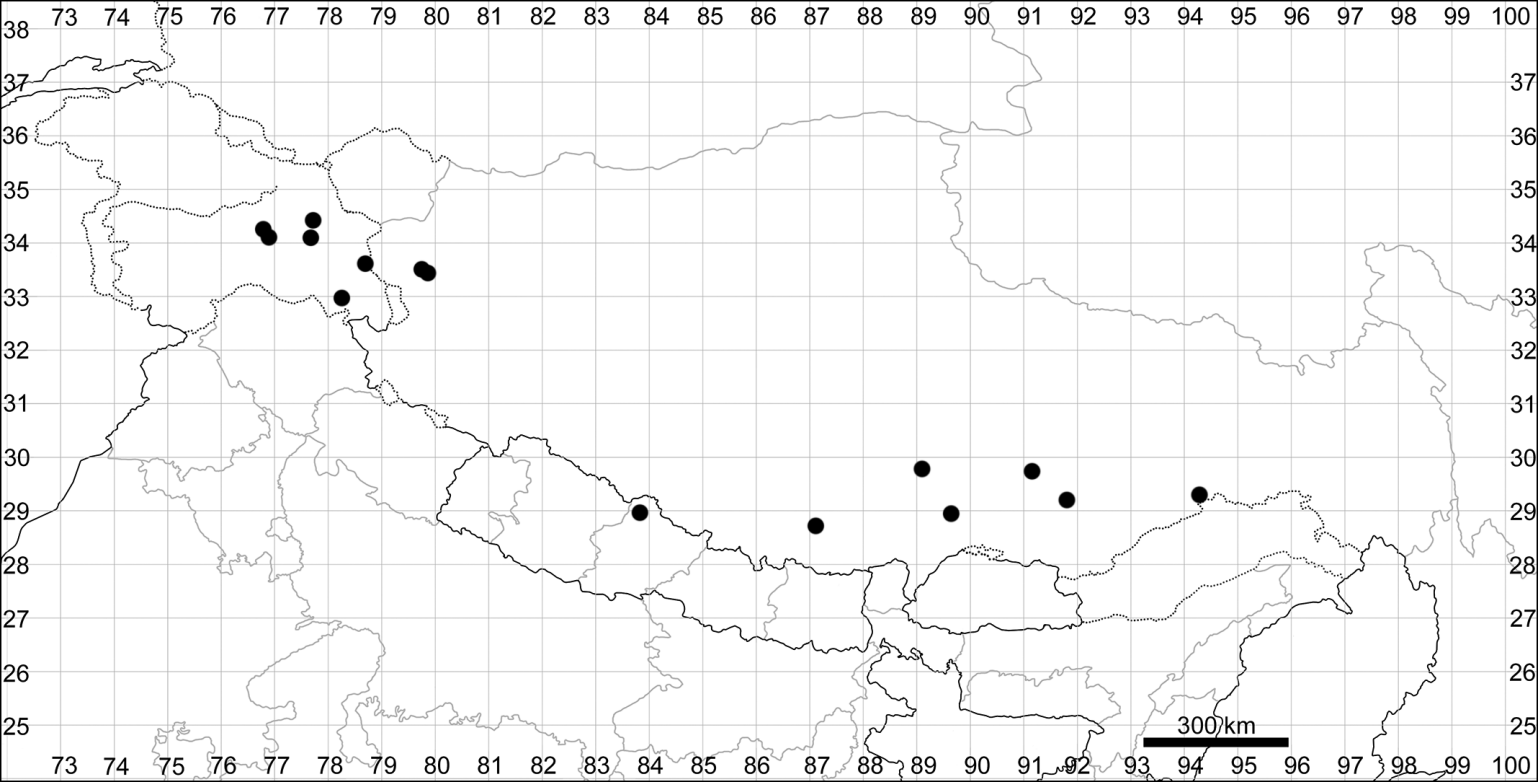
Distribution map of *Oxybasisglauca*.

####### Specimens examined.

**CHINA**: **Xizang**: **Ngari Prefecture**: Rutog (Ritu) County, west of Lake Bangong (Pangong Tso), 4200 m a.s.l., 11 Jul 1974, *Biological Institute Tibet Expedition Team 3604* (HNWP40756); Rutog (Ritu) County, Lake Bangong (Pangong Tso), 4240 m a.s.l., 4 Sep 1976, *Qinghai-Tibet Team Vegetation Group 13673* (PE00511499); **Xigazê Prefecture**: Tingri, Jul 1921, *A. Wollaston 265* & *266* (K); Namling (Nanmulin) County, Gangba, 4000 m a.s.l., 3 Aug 1972, *Tibet Chinese Herbal Medicine Survey Team 983* (PE00511498, HNWP29013); Gyangzê (Jiangzi) County, 3760 m a.s.l., 5 Sep 1974, *Qinghai-Tibet Team 74-2068* (KUN0587252); **Lhasa City**: Lhasa, 15 Apr 1960, *Fu & Zhang 0046* (PE00511501); **Shannan Prefecture**: Zetang, Changzhu, 3550 m a.s.l., 27 May 1986, *K.Y. Lang* et al. *1470* (PE00511496); **Nyingchi Prefecture**: [Nyingchi (Linzhi) County] Tsangpo, nr Lamdo, 29°23'N, 94°22'E, 9800 ft a.s.l., 30 May 1938, *F. Ludlow* et al. *4574* (BM, E);

**INDIA: Jammu & Kashmir**: [without locality and date] *Jacquemont 1151* (P04993386; P04993387); Leh, 1 Aug 1976, *B.M. Wadhwa 59401* (BSD); Tirit vill., 3200 m a.s.l., 18 Aug 1976, *B.M. Wadhwa 59977* (BSD); Lukurg to Chushul, 4275 m a.s.l., 29 Aug 1976, *B.M. Wadhwa 60174* (BSD); Ladakh, Rupshu Region, Tso Moriri, 4590 m a.s.l., 16 Jul 2000, *L. Klimeš 923* (PRA); Ladakh, Rupshu Region, Samad Rokchen, 4550 m a.s.l., 8 Sep 2005, *L. Klimeš 6253* (PRA); Ladakh, Indus valley, Sham (W), Wanla to Tarchit, 3160–3180 m a.s.l., 20 Sep 2006, *L. Klimeš 7280*(PRA); Ladakh, Indus valley, Sham (W), Angshang to Konzke La, 4310–4370 m a.s.l., 21 Sep 2006, *L. Klimeš 7308* (PRA).

The specimens collected in Himachal Pradesh and Uttarakhand and identified as “*Chenopodium glaucum*” belong to other taxa, mostly *Atriplexpallida* (Moq.) Sukhor.

**NEPAL**: **Western**: **Dhaulagiri Zone**: Mustang Distr., Chuksang (2970 m a.s.l.)–Tetang (3000 m a.s.l.)–Gnyu Pass (4100 m a.s.l.), 28°55'N–28°51'N, 83°49'E, 13 Jul 2000, *Y. Iokawa* et al. *20020169* (E00435483).

####### General distribution.

Eurasia (mostly temperate regions), North America, North Africa. In Himalaya and Tibet, the species is found at high altitudes (2800–4600 m a.s.l.) and the plants hardly reach 30 cm in length. Its frequency is not known, but it seems to be rare in our area.

###### 4. Atriplex

Taxon classificationPlantaeCaryophyllalesChenopodiacea

L., Sp. Pl.: 979 (1753).

####### Lectotype

(designated by McNeill et al. 1983): *Atriplexhortensis* L.

####### Description.

Annual herbs, subshrubs or shrubs, monoecious or dioecious, covered with bladder hairs having short basal cell and globular terminal cell; occasionally, other hair types (simple bristle-like or curved hairs) can be detected under higher magnification. Leaves alternate or opposite, simple, flat, petiolate. Inflorescence leafy or not, consisting of few or numerous flowers that are usually unisexual. The male and bisexual flowers are enclosed by (3–4)5 green perianth segments that remain unchanged at fruiting in bisexual flowers; female flowers enclosed by two accrescent perianth segments [valves] (often called ‘bracteoles’ or bract-like cover) that are free or connate to varying degrees. Seeds have a vertical embryo, some seeds rarely with horizontally orientated embryo. Often (in annual species) heterospermy is present (seed coat black and brown/red).

The largest genus amongst the Chenopodiaceae (~260 species).

##### Key to the species

**Table:** 

1	Leaves opposite to alternate, ovoid or triangular, usually reddish and sparsely covered with bladder hairs (except young leaves, which are densely covered with bladder hairs). Plant bears three types of flowers (male and bisexual flowers surrounded by perianth of (4)5 normal segments and female flowers supported only by a bract-like cover formed of two accrescent valves that are only basally connate); seeds heteromorphic (black and brown)	**4. *A.hortensis***
–	Leaves alternate, broadly ovoid, rhombic to triangular, always farinose (at least below) or often additionally covered with brown bladder hairs; no bisexual flowers present (male flowers surrounded by perianth of (4)5 normal segments and female flowers supported by a bract-like cover); segments of bract-like cover at least half connate; seeds red and brown	**2**
2	Inflorescence aphyllous or bracteate; bract-like cover of female flowers smooth or with 2(3) small outgrowths	**2. *A.pallida***
–	Inflorescence leafy (almost) to the top	**3**
3	Plant forming tumble-weed habit; stem erect with spreading branches; leaves rhombic or ovate; bract-like cover of female flowers either smooth or with thorn-like outgrowths (on the same plant)	**1. *A.centralasiatica***
–	Plant not forming tumble-weed habit; leaves oblong; bract-like cover smooth or with 1–2 small outgrowths	**3. *A.pamirica***

###### 1. Atriplex centralasiatica

Taxon classificationPlantaeCaryophyllalesChenopodiacea

Iljin, Act. Inst. Bot. Ac. Sci. USSR, ser. 1, 2: 124 (1936)

####### Holotype.

[KAZAKHSTAN] Lac. Balchasch, prope Aczie [Balkhash Lake, near Acshi], 19 Sep 1930, *E. Czerniakowska 819* (LE!).

####### Taxonomic notes.

Some specimens collected in China (e.g. Xinjiang, Xizang and Qinghai), East Kazakhstan and Kyrgyzstan are characterised by the presence of long-stalked bract-like cover of female flowers and such plants were described as *A.megalotheca* Popov and *A.tianschanica* Pratov. We believe that these two species are indeed varieties of *A.centralasiatica*. In the preliminary, recently constructed ITS tree (Sukhorukov et al. in prep.), the specimens of *A.centralasiatica* and *A.megalotheca* (*A.tianschanica* was not included in the analysis) are intermixed within one subclade (as a part of the C_4_-*Atriplex* clade). A closely related *Atriplexsibirica* possesses bract-like cover with warty or spiny outgrowths, whereas *A.centralasiatica* is distinguished by having two forms of the valves, with and without outgrowths (smooth on dorsal side). The results of the first molecular phylogeny of the genus support the existence of two morphologically similar species, *A.sibirica* and *A.centralasiatica* (Kadereit et al. 2010). The distribution area of *A.sibirica* mostly covers the northern and middle parts of Central Asia and no specimens were seen from Tibet and Himalaya. For this reason, *A.centralasiatica* is still considered to be in its own specific rank.

####### Description.

Annual up to 50 cm, basally branched and forming a tumble-weed habit at the fruiting stage. Leaves triangular or rhombic-triangular, rarely ovate, mostly entire. Inflorescence leafy to the top. Female flowers in axillary clusters and intermixed with male flowers, forming terminal inflorescences. Bract-like cover enclosing the female flowers sessile or pedunculate (peduncles up to 2 cm), rhombic with concrescent (from 1/2 to 2/3) valves 5.0–10.0(15.0) mm long, sclerified and slightly inflated, entire or dentate, either without abaxial outgrowths or with acute outgrowths. Seeds heteromorphic: red seeds convex, 1.5–2.0(2.5) mm in diameter, brown seeds 2.0–2.5(3.0) mm, flattened.

####### Habitat.

Screes, roadsides at altitudes of 3500–4800 m a.s.l. (in Himalaya and Tibet). Widespread in Tibet and in some parts of Ladakh (India).

####### Phenology.

Flowering: July-September; fruiting: September-October.

####### Distribution.

See Fig. 16.

**Figure 16.**
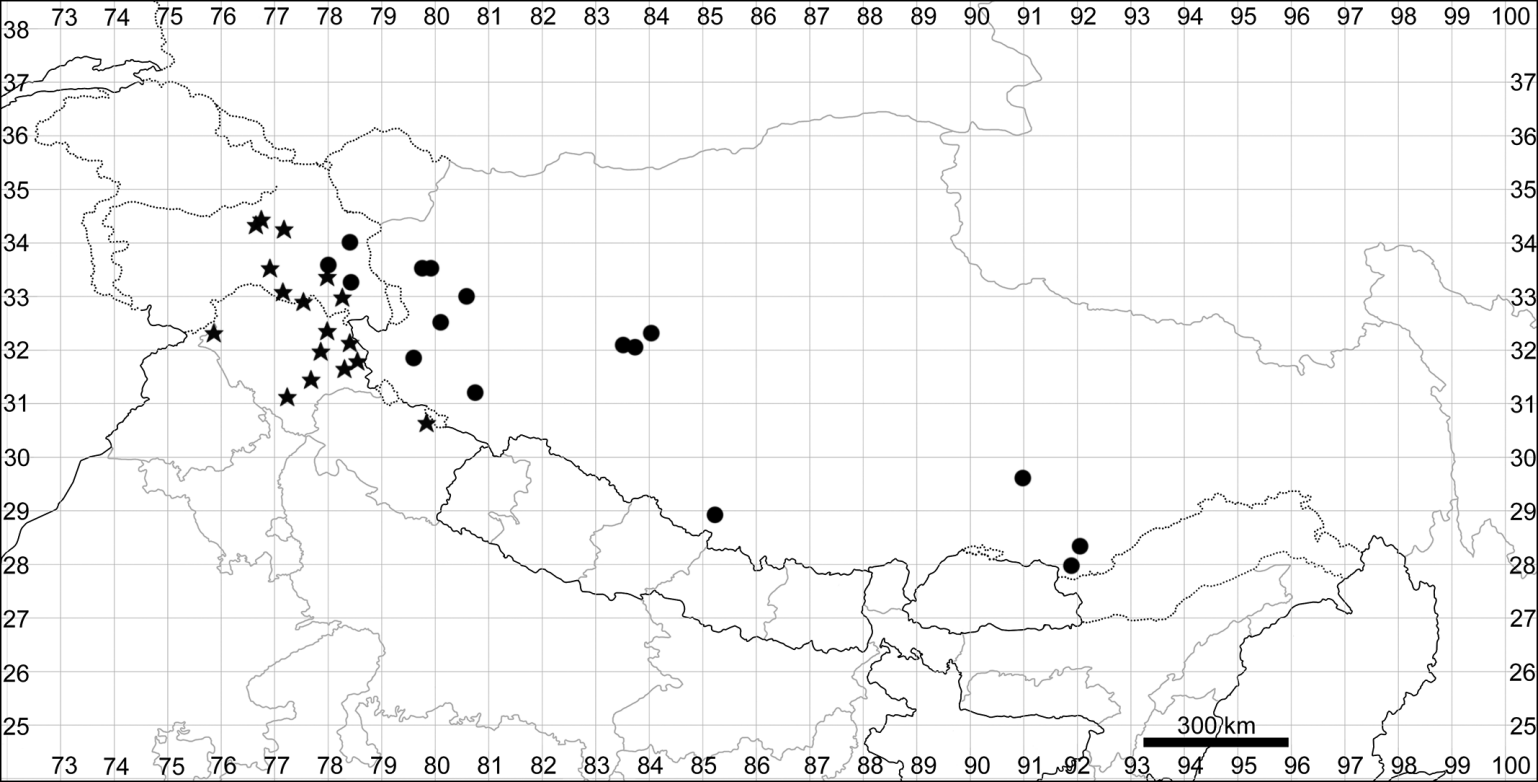
Distribution map of *Atriplexcentralasiatica* (circles) and *A.pallida* (stars).

####### Specimens examined.

**CHINA**: **Xizang**: **Ngari Prefecture**: [Gar (Gaer) County] Shiquanhe, 4300 m a.s.l., 30 Jul 1974, *Biological Institute Tibet Expedition Team 3749* (PE00121143); Gêrzê (Gaize) County, Datan, 4400 m a.s.l., 8 Sep 1974, *Biological Institute Tibet Expedition Team 4320* (PE00121144, HNWP40796); Zanda (Zhada) County, Qangzê (Xiangzi) Distr., 4100 m a.s.l., 5 Jul 1976, *Qinghai-Tibet Team 76-7926* (PE00121133, PE00121134); Zanda (Zhada) County, Qangzê (Xiangzi) Distr., 4200 m a.s.l., 5 Jul 1976, *Qinghai-Tibet Team Vegetation Group 12768* (PE00165736); Zanda (Zhada) County, Qangzê (Xiangzi) Distr., Rabgyailing (Rebujialin), 4100 m a.s.l., 5 Jul 1976, *Qinghai-Tibet Team Meadow Group 76-083* (PE00541020); Gar (Gaer) County, Moincêr (Menshi) Distr., 4300 m a.s.l., 10 Jul 1976, *Qinghai-Tibet Team 76-7988* (PE00121135, PE00121136, KUN0586474); Gar (Gaer) County, nr Moincêr hot spring, 4400 m a.s.l., 10 Jul 1976, *Qinghai-Tibet Team Vegetation Group 13227* (PE00165737, PE00235125); Gar (Gaer) County, Moincêr (Menshi) Distr., 4300 m a.s.l., 10 Jul 1976, *Qinghai-Tibet Team Meadow Group 76-101* (PE00541019); Rutog (Ritu) County, Rabang (Rebang) Distr., 4300 m a.s.l., 28 Aug 1976, *Qinghai-Tibet Team 76-9125* (PE00121139); Rutog (Ritu) County, Rabang (Rebang) Distr., 4500 m a.s.l., 28 Aug 1976, *Qinghai-Tibet Team Meadow Group 76-12971* (PE00541017); Rutog (Ritu) County, Lake Bangong (Pangong Tso), 4250 m a.s.l., 3 Sep 1976, *Qinghai-Tibet Team 76-8743* (PE00121140, PE00121141, PE00121142); Rutog (Ritu) County, Lake Bangong (Pangong Tso), 4220 m a.s.l., 3 Sep 1976, *Qinghai-Tibet Team Meadow Group 76-237* (PE00541015, PE00541016); Rutog (Ritu) County, E Lake Bangong (Pangong Tso), 4250 m a.s.l., 3 Sep 1976, *Qinghai-Tibet Team Vegetation Group 13551* (PE00165735); Gêrzê (Gaize) County, Marmê (Mami) Distr., 4500 m a.s.l., 8 Sep 1976, *Gansu Agriculture University 159* (PE00541018); Gêrzê (Gaize) County, Marmê (Mami) Distr., Camco (Chacuo), 4500 m a.s.l., 8 Sep 1976, *Qinghai-Tibet Team 10152* (PE00121131, KUN0586476); Rutog County, Bangong lake (Pangong Tso), 4300 m a.s.l., 3 Sep 1990, *Y. Fei* et al. *737 & 741* (KUN0264613, KUN0264614); **Xigazê Prefecture**: Gyirong (Jilong) County, 4150 m a.s.l., 28 Jul 1975, *Qinghai-Tibet Team 7083* (PE00121130); **Lhasa City**: Kyichu valley, E of Lhasa, Aug 1904, *H.J. Walton & F.E. Younghusband* s.n. (K); **Shannan Prefecture**: Cona (Cuona) County, 4300 m a.s.l., 24 Aug 1975, *Qinghai-Tibet Team Additional Group 751508* (PE00121137, PE00121138, KUN0587334); South Tibet, Subansiri tributary Lhuenze–Cona (Camp 11), 28°23'N, 92°8'E, 4300 m a.s.l., 1 Aug 1998, *B. Dickoré 10293* (MSB144289); Cona (Cuona) County, 4000 m a.s.l., 16 Aug 1977, *B.Z. Guo* et al. *22787* (WUK0344207);

**INDIA** (new records for the country): **Jammu & Kashmir**: Ladakh, Rupshu, 4 Sep 1970, 4600 m a.s.l., *U.C. Bhattacharyya 41004* (BSD); Ladakh, Lukung vill., 29 Aug 1976, *B.M. Wadhwa 60146 & 60176* (BSD); Ladakh, Indus valley, Stot (E) [Stod River valley] Sumdo to Zildat La, 4760 m a.s.l., 15 Sep 2003, *L. Klimeš 3493* (PRA); Ladakh, Rupshu, Samad Rakchan vill., 33°20'44"N, 78°1'47"E, 4620 m a.s.l., 7 Sep 2005, *L. Klimeš 6249* (PRA).

The records from India (Ladakh) were previously identified as *A.pamirica* Iljin.

####### General distribution.

Widespread in Central Asia and Tibet (Asian part of Russia, China, Kazakhstan, Kyrgyzstan, North India, Mongolia, Tajikistan and E Uzbekistan).

###### 2. Atriplex pallida

Taxon classificationPlantaeCaryophyllalesChenopodiacea

(Moq.) Sukhor., Phytotaxa 226(3): 288 (2015).

≡Chenopodiumpallidum Moq., Chenop. Monogr. Enum.: 30 (1840). **Lectotype** (designated by Sukhorukov and Kushunina 2014): [Probably NE INDIA] Voyage de V. Jacquemont aux Indes Orient., Jacquemont 1377 (P04993339! isolectotypes P00606416! P04993338! P05047853!). Image of the lectotype available at: https://science.mnhn.fr/institution/mnhn/collection/p/item/p04993339 (isolectotypes at: https://science.mnhn.fr/institution/mnhn/collection/p/item/p00606416https://science.mnhn.fr/institution/mnhn/collection/p/item/p04993338https://science.mnhn.fr/institution/mnhn/collection/p/item/p05047853)
=Atriplexschugnanica Iljin, Acta Inst. Bot. Acad. Sc. URSS, ser. 1, 2: 123 (1936). **Lectotype** (designated by Sukhorukov and Tscherneva in Sukhorukov 2006): [TAJIKISTAN] Roschan, Usoj, in ripa flum. Bartanga [river bank of Bartanga river], in decliviis lapidosis [rocky slopes], 20 Aug 1897, *S. Korshinsky 4692* (LE! isolectotype LE!).

####### Description.

Annual up to 70 cm, basally branched with ascending stems. Leaves triangular, oblong or rhombic-triangular, entire or dentate, sometimes with two marginal lobes; upper leaves lanceolate. Inflorescence leafy in lower part. Female flowers in axillary clusters and intermixed with male flowers forming terminal inflorescences. Bract-like cover enclosing the female flowers sessile or shortly pedunculate (peduncles up to 3.0 mm), ovate, rhombic or triangular, 1.2–5.0(7.0) mm long, sclerified but not or slightly inflated, entire or dentate, usually without outgrowths on the back. Seeds heteromorphic: red seeds convex, 0.7–1.2 mm in diameter, brown seeds 1.2–1.8 mm, flattened.

####### Habitat.

Screes, sands and ruderal sites; 2600–4800 m a.s.l.

####### Phenology.

Flowering: August-September; fruiting: September-October.

####### Distribution.

See Fig. 16.

####### Specimens examined.

**INDIA**: **Jammu & Kashmir**: Ladakh, Rupshu, 4600 m a.s.l., 4 Sep 1970, *U.C. Bhattacharyya 41004* (BSD); Ladakh, 24 Jul 1976, *B.M. Wadhwa 58992* (BSD); Ladakh, Fatula, 3990 m a.s.l., 25 Jul 1976, *B.M. Wadhwa 58984 & 58992* (BSD); Ladakh, Lamayuru, 3400 m a.s.l., 26 Aug 1979, *Bruhn* s.n. (M70063); Ladakh, Saspol, 3120 m a.s.l., 8 Sep 1989, *L. Klimeš & M. Šrůtek 22/8* (PRA); Ladakh, Zanskar Region, Tsarap, Kurgiakh vill., 4100–4150 m a.s.l., 18 Aug 2004, *L. Klimeš 4127* (PRA); Ladakh, Zanskar Region, Tsarap, Shadde, 4150–4290 m a.s.l., 25 Aug 2004, *L. Klimeš 4217* (PRA); Ladakh, Zanskar Region, S of Padum, 3570–3590 m a.s.l., 29 Aug 2004, *L. Klimeš 4289* (PRA); Ladakh, Rupshu Region, Tso Moriri, Thakshang, 4670–4710 m a.s.l., 13 Sep 2005, *L. Klimeš 6299* (PRA); Ladakh, Indus valley, Domkhar Dha, Hanu Pur, 3360–3440 m a.s.l., 9 Sep 2006, *L. Klimeš 7019* (PRA);

**Himachal Pradesh**: Kinnaur [distr.], above Leppa [Lippa], 8000–12000 ft a.s.l., Aug 1847, *T.T. Thomson* s.n. (BM, G, K, M); [Kangra distr.] Nurpur, Jul 1856, [*Schlagintweit*] s.n. (LE); Simla distr., Simla [without date and collector] (K); [Kinnaur distr.] Leppa, 2600 m a.s.l., 17 Aug 1890, *J.H*. *Lace 529* (E); [Border between Himachal Pradesh and Jammu & Kashmir] Lahaul [Lahaul Distr.], Serchu [Sarchu], 13000 ft a.s.l., 30 Jun 1941, *N.L*. *Bor 15116* (DD, K); Bashahr vill., 2900 m a.s.l., 5 Sep 1963, *N.C. Nair 30540* (BSD); [Kinnaur Distr.] Pooh, 3050 m a.s.l., 7 Jun 1972, *K.P. Janardhanan 47780 & 47781* (BSD); Spiti valley, Kibber, 4250 m a.s.l., 2 Aug 1972, *U.C. Bhattacharyya 49260* (BSD); Spiti valley, Tabo vill., 19 Aug 1994, *S.K. Musti & S. Singh 88480* (BSD); Kinnaur Distr., Skiba vill., 9 Oct 1991, *K.P. Janardhanan 46553* (BSD); Spiti Distr., Pin Valley National Park, 27 Aug 2002, *K.C. Sekar 100735* (BSD); **Uttarakhand**: Chamoli Distr., Malari vill., 20 Aug 1979, *B. Nailhani 56011* (BSD).

####### General distribution.

Tajikistan, Afghanistan, NE Pakistan, N India. Most likely present in W Xizang (China). Common in Ladakh, many parts of Himachal Pradesh and Uttarakhand (Chamoli Distr.).

###### 3. Atriplex pamirica

Taxon classificationPlantaeCaryophyllalesChenopodiacea

Iljin, Acta Inst. Bot. Ac. Sc. USSR, ser. 1, 2: 124 (1936)

Atriplex pamirica Iljin, Acta Inst. Bot. Ac. Sc. USSR, ser. 1, 2: 124 (1936). **Holotype**: [TAJIKISTAN] Khargosh, in ripa lac. Kara-kul [bank of Kara-kul Lake], 30 Jul 1878, *Yu. Ashurbayev* s.n. (LE!).
≡Atriplextataricavar.pamirica (Iljin) G.L.Chu in Kung & Tsien, Fl. Reipubl. Pop. Sin. 25(2): 46 (1979).

####### Taxonomic notes.

*Atriplexpamirica* is a morphologically highly recognisable species (Sukhorukov 2006). The varietal rank Atriplextataricavar.pamirica, proposed by Chu in Kung and Tsien (1979), cannot be accepted. *Atriplextatarica* is a taller plant with an aphyllous or bracteose inflorescence. The results of molecular phylogeny have confirmed the specific rank of *A.pamirica* (Kadereit et al. 2010).

####### Description.

Annual up to 30(50) cm, branched at the base with ascending stems but not forming tumble-weed habit at the fruiting stage. Stems and leaves covered with white and brown bladder hairs. Leaves grey on both sides, oblong or spatulate, entire or crisp. Inflorescence leafy. Bract-like cover enclosing the female flowers 3.0–7.0 mm long, rhombic, entire or dentate with concrescent (up to 1/3–1/2) valves, valves sessile or pedicellate (pedicels up to 3 mm), sclerified and inflated in the lower part, abaxial surface smooth or with two small outgrowths. Seeds heteromorphic: red seeds convex, 1.0–1.7 mm, brown seeds flattened, 1.2–1.8 mm.

####### Habitat.

Screes or roadsides at altitudes of 3500–5300 m a.s.l.

####### Phenology.

Flowering: July-September; fruiting: September-October.

####### Distribution.

See Fig. 17.

**Figure 17.**
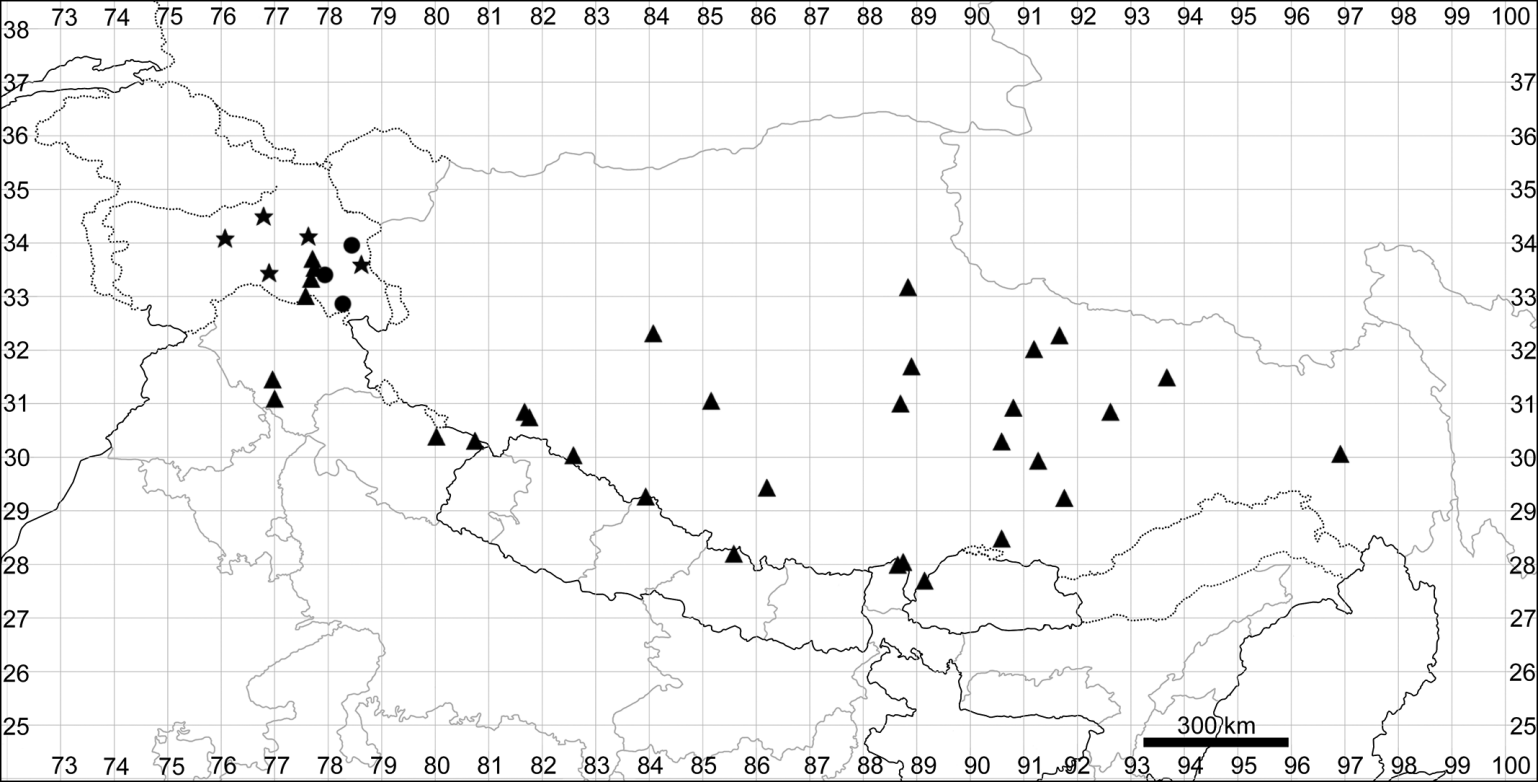
Distribution map of *Atriplexpamirica* (circles), *A.hortensis* (stars) and *Microgynoeciumtibeticum* (triangles).

####### Specimens examined.

**INDIA**: **Jammu & Kashmir**: Ladakh, Rupshu Region, Samad Rakchan, Pongunagu, 33°22'N, 77°57'E, 4670 m a.s.l., 29 Aug 1998, *L. Klimeš 254* (PRA); Ladakh, Rupshu Region, Tso Moriri, 5170–5200 m a.s.l., 12 Aug 2001, *L. Klimeš 1335* (PRA); Ladakh, Pangong Region, Spangmik, 4300–4320 m a.s.l., 9 Sep 2002, *L. Klimeš 2678* (PRA).

####### General distribution.

Tajikistan, Afghanistan, NE Pakistan and N India. Most likely, present in NW Xizang (China).

###### 4. Atriplex hortensis

Taxon classificationPlantaeCaryophyllalesChenopodiacea

L., Sp. Pl.: 1053 (1753)

**Lectotype** (designated by McNeill et al. 1983): Hort. Sicc. Cliff. (BM000647538! image of the lectotype available at http://www.nhm.ac.uk/resources/research-curation/projects/clifford-herbarium/lgimages/BM000647538.JPG).
=A.heterantha Wight, Icon. Pl. Ind. Orient. 5: tab. 1787 (1852). **Holotype**: [INDIA] Coimbatore, Nov 1849 [*Wight*] *2481* (K000898565!).
=A.microtheca Moq. in DC., Prodr. 13(2): 91 (1849) nom. illegit., non Fries 1835. **Type**: not selected.

####### Description.

Annual up to 150 cm, erect, usually branched. Leaf petioles 1.5–4.0 cm, blades 4.0–15.0(22.0) × 3.5–7.0(8.0–20.0) cm, triangular-hastate or ovoid, entire or dentate, basally slightly cordate or rounded, apically obtuse, green or reddish, glabrous or with scattered bladder hairs abaxially. Inflorescence mostly basally leafy, spike-like. As a rule, three kinds of flowers are present: (i) male flowers surrounded by a perianth with (4)5 segments, (ii) bisexual flowers surrounded by a perianth with (4)5 segments (sometimes not observed in the Indian specimens) and (iii) female flowers supported only by two accrescent, basally connate perianth valves forming a bract-like cover up to 12.0(15.0–20.0) mm in diameter, valves sessile or sometimes tapered into a peduncle up to 5 mm long (seen on Indian material). Seeds heteromorphic (three seed types present on one individual). Black seeds (1.6–1.8 mm in diameter) are formed in second (ii) and third (iii) flower types while the brownish ones (2.0–3.5 mm) are usually yielded by female (iii) flowers.

####### Habitat.

Disturbed places (cultivated in vegetable gardens or as a weed); 2500–3600 m a.s.l. (in our area).

####### Phenology.

Flowering: July-September; fruiting: September-October.

####### Distribution.

See Fig. 17.

####### Specimens examined.

**INDIA: Jammu & Kashmir**: Ladakh, Leh, 1856, *Schlagintweit* [several sheets s.n., *nrs 9 & 10*] (BM, G, P04930288); Ladakh, Upschi to Leh, Aug 1856, *Schlagintweit 1290* (E); Ladakh, Chushul, 5500 m a.s.l., 24 Sep 1975, *M.V. Viswanathan 55009* (BSD); Ladakh, Indus valley, Domkhar Dha, Hanu-Thang vill., 2810 m a.s.l., 31 Aug 2002, *L. Klimeš 2554* (PRA); Ladakh, Zanskar Region, S of Padum, 3580 m a.s.l., 1 Sep 2003, *L. Klimeš 3418* (PRA); Ladakh, Leh, 3450 m a.s.l., 28 Sep 2003, *L. Klimeš 3655* (PRA); Ladakh, Suru Region, Baroo, 2730 m a.s.l., 18 Sep 2004, *L. Klimeš 4912* (PRA); Ladakh, Indus valley, Domkhar Dha, Hagnis vill., 3150–3250 m a.s.l., 13 Sep 2006, *L. Klimeš 7169* (PRA);

**NEPAL**: “Napalia” [without precise location and year] 1821, *R. Wight 9089* (K, LE).

####### General distribution.

Eurasia (seems to be native in southern Europe and Turkey only); cultivated and sometimes well-established in Northern Himalaya, North and South America and Australia.

##### Excluded species

All records of the species listed below are erroneous (see for more Sukhorukov 2006) and belong to different species listed above or even to some *Chenopodium*.

*Atriplexlaciniata* sensu auct. non L. It is distributed on the shores of West, South-West and Central Europe and eastern part of North America.

*A.rosea* auct. non L. It is distributed in Europe, North Africa and Turkey; as alien in North and South America.

*A.nitens* sensu Paul (2012) non Schkuhr [=*A.sagittata* Borkh.]. It is known from Europe and temperate Asia (Caucasus, Siberia).

*A.crassifolia* sensu Dhar and Kachroo (1983), Sekar and Srivastava (2009), Paul (2012) non C.A.Mey. An endemic to Eastern Kazakhstan and West Xinjiang, China (Sukhorukov 2006, Nobis et al. 2017).

###### 5. Microgynoecium

Taxon classificationPlantaeCaryophyllalesChenopodiacea

Hook.f. in Bentham & Hook.f., Gen. Pl. 3(1): 56 (1880)

Microgynoecium Hook.f. (1880). Type: Microgynoeciumtibeticum Hook.f.

####### Description.

Monoecious annuals up to 20 cm with scattered, long-stalked bladder hairs; stems branched, prostrate or ascending; at elevations above 5000 m plants are often cushion-like. Leaves fleshy or not, petiolate (petiole up to 1.5–2.0 cm), green, leaf blades up to 1.5 cm, decreasing in size upwards, entire, rhombic, ovate or oblong, mostly entire or sometimes with small lateral lobes. Male flowers inconspicuous with (4)5 hyaline perianth segments and 2–4 stamens with large (ca. 2.0 mm) protruding filaments in anthesis, anthers ~0.5 mm; female flowers hidden in bract (each bract usually covers 3 female flowers) without a conspicuous perianth. Fruits and seeds dimorphic. The fruits of the first type reddish-brown, 0.9–1.0 mm long, 0.4–0.5 mm thick, ear-like pericarp appendages in the upper part of the fruit up to 0.1 mm and much smaller in the lower part; pericarp one-layered, some cells papillate; seed testa 10–12 µm thick, undulate, outer cell walls with stalactites. The fruits of the second type dark, 1.0–1.5 mm long, 0.75–0.80 mm thick (visibly swollen) with ear-like pericarp outgrowths up to 0.1 mm expressed throughout; pericarp one-layered with papillate cells, 12–15 µm; seed coat testa 20–25 µm thick. Seed coat tegmen of both seed types minute; embryo vertical, annular; perisperm present. The fruit anatomy is similar to that of *Archiatriplex* Chu (Kadereit et al. 2010), especially in the presence of pericarp outgrowths over the fruit surface (Sukhorukov 2014).

One species in Himalaya, Tibet, Pamir and Tian Shan.

###### 1. Microgynoecium tibeticum

Taxon classificationPlantaeCaryophyllalesChenopodiacea

Hook.f., Fl. Brit. India 5(13): 9 (1890)

####### Lectotype

(designated by Sukhorukov and Kushunina 2014): [INDIA, Uttarakhand State, Kumaon Division] Topedunga, 15000 ft a.s.l., *S. Strachey & J.E. Winterbottom 1* (K000898740! image of the lectotype available at http://apps.kew.org/herbcat/getImage.do?imageBarcode=K000898740, isolectotypes K! P04921830! P04930937!).

####### Description.

See the genus description. *Microgynoeciumtibeticum* is often confused with *Chenopodiumpamiricum*; both species are present in North Himalaya at high altitudes. In the early blooming stage, *M.tibeticum* differs by having unisexual flowers and long (2.0 mm) filaments of stamens protruding from the perianth (*Chenopodiumpamiricum* has bisexual flowers and shorter filaments). The fruit and seed characters greatly differ between the genera.

####### Habitat.

Screes, grassy slopes or ruderal sites; 3500–5500 m a.s.l.

####### Phenology.

Flowering: August-October; fruiting: September-November.

####### Distribution.

See Fig. 17.

####### Specimens examined.

**CHINA: Xizang**: **Ngari Prefecture**: Gêrzê (Gaize) County, 4250 m a.s.l., Aug 1972, *Li 065* (PE00526697); Burang (Pulan) County, Mapam Yumco (lake), 4500 m a.s.l., 24 Aug 1974, *Biological Institute Tibet Expedition Team 4204* (HNWP40746); Coqên (Cuoqin) County, Cêri (Cishi) Distr., 4600 m a.s.l., 17 Sep 1976, *Qinghai-Tibet Team Lang 10331* (PE00526701); Burang (Pulan) County, E Hor (Huoer), 30°42'52.86"N, 81°42'54.21"E, 4682 m a.s.l., 3 Sep 2012, *FLPH Tibet Expedition 12-0067* (PE); **Nagqu Prefecture**: Xainza (Shenzha) County, 4580 m a.s.l., 19 Jul 1961, *Wang 3582* (PE00526695); Amdo County, 4750 m a.s.l., 14 Aug 1963, *Yang 2233* (WUK); Amdo (Anduo) County, 4750 m a.s.l., 14 Aug 1963, *Z.Y. Qin 10163* (HNWP000180); Baingoin (Ban’ge) County to Lake Qilin (Siling County), 4500–4600 m a.s.l., 17 Jul 1976, *Qinghai-Tibet Team 10630* (PE00526693, KUN0587747); Shuanghu County, 4850 m a.s.l., 21 Jul 1976, *Qinghai-Tibet Team 9751* (PE00526700, KUN0587762); Shuanghu County, 4800 m a.s.l., 2 Aug 1976, *Qinghai-Tibet Team Vegetation Group 12121* (PE00235029), *Qinghai-Tibet Team Vegetation Group 12122* (PE00235045); Xainza (Shenzha) County, 4750 m a.s.l., 1 Sep 1976, *Qinghai-Tibet Team 10099* (KUN0587755); Biru County, 3 Sep 1976, *Qinghai-Tibet Team 11180* (KUN0587749); Amdo, Upper Salween basin, 4 Sep 1989, *B. Dickoré 4675* (MSB157861); Central/East Plateau, Amdo (Ragnag), 32°17'N, 91°43'E, 4820 m a.s.l., 10 Aug 1993, *G. & S. Miehe 9461/05* (herb. Miehe); Nagqu County, 30°48'N, 92°36'E, 4750 m a.s.l., 31 Jul 2004, *G. Miehe* et al.. *04-060-05* (herb. Miehe); [Amdo (Anduo) County] Lake Xiketang, 32°04'N, 91°11'E, 4710 m a.s.l., 2 Aug 2005, *Nölling & Hanspach NX05-039-03* (herb. Miehe); **Xigazê Prefecture**: [Yadong County] Phari [Pagri], 14000 ft a.s.l., 12 Sep 1912, *R. Lepcha 253* (E, G); [Gamba County] Kamba Jong [Kamba Dzong], Sep 1903, *Prain 1172* (B-100655561, K); [Ngamring (Angren) County] Tsabsang Tso, 29°24'N, 86°14'E, 4900 m a.s.l., 2 Sep 2003, *G. & S. Miehe 03-060-03* (herb. Miehe); **Lhasa City**: Lhünzhub (Linzhou) County, Poindo (Pangduo), 4050 m a.s.l., 7 Oct 1972, *Tibet Chinese Herbal Medicine Survey Team 1945* (PE00526692); NW of Lhasa, Nyainqentanglha Shan, 30°17'N, 90°38'E, 4850 m a.s.l., 9 Aug 1989, *B. Dickoré 3489* (K, MSB157862); [Damxung (Dangxiong) County] Namtso Lake, 30°54'N, 90°47'E, 4720 m a.s.l., 14 Sep 2005, *Nölling & Hanspach NX05-256-01* (herb. Miehe); **Shannan Prefecture**: Nêdong (Naidong) County, Zêtang (Zedang) to [Qusum (Qusong) County] Yardoi Chagla (Yaduizhala), 4370 m a.s.l., 16 Jul 1975, *Wu* et al. *75-794* (PE00526704, KUN0587753); [Nagarzê County] Nagarze-Lozhak, 28°29'N, 90°33'E, 5060 m a.s.l., 23 Jul 1994, *B. Dickoré 9879* (MSB157863); **Qamdo Prefecture**: Baxoi (Basu) County, N of Mt. Dêmo La, 4200 m a.s.l., 17 Aug 1980, *Ni* et al. *1216* (PE00526698);

**INDIA**: **Jammu & Kashmir**: Ladakh, Rupshu, Rachogba, 13400 ft a.s.l., 24 Jun 1931, *W. Koelz 2105* (E, K); Ladakh, Gya, Aug 1933, *W. Koelz 6461* (H1037932); Ladakh, Chushul, 5500 m a.s.l., 24 Aug 1975, *M.V. Viswanathan 55021* (BSD); Ladakh, Taglang La, 18 Aug 1988, *H.J. Chowdhery & B.P. Uniyal 86208* (BSD); Ladakh, Zanskar Region, Zara, Jakang vill., 4580 m a.s.l., 25 Aug 1998, *L. Klimeš 194* (PRA); **Himachal Pradesh**: Lahaul, Kyelong [Kilang], 15000 ft a.s.l., 19 Jul 1941, *N.L*. *Bor 15184* & *R.E*. *Cooper 5462* (B, DD, K); [Simla distr.] Manan, 4500 m a.s.l., 14 Aug 1972, *U.C. Bhattacharyya 49452* (BSD); **Uttarakhand**: [Kumaon division] Topedunga, 15000 ft a.s.l., *S. Strachey & J.E. Winterbottom 1* (K, lectotype!); Kumaon, Kutti valley, 14000–15000 ft a.s.l., 1 Aug 1886, *J.F. Duthie 5952* (K, WU); **Sikkim**: Gholamo [Tso Lhamo lake], 6 Sep 1911, *R. Lepcha 5469* (E); Gongchungchu, 17820 ft a.s.l., 16 Aug 1972, *Pradhan* et al. *118* (E);

**NEPAL**: **Midwestern**: **Karnali Zone**: Below Namja La, 15500 ft a.s.l., 22 Aug 1952, *O. Polunin, W.R. Sykes & L.H.J. Williams 5387* (BM); **Western: Dhaulagiri Zone**: Mustang Distr., Lo-Manthang, NW of Lo-Manthang, 4500 m a.s.l., 29°15'N, 83°55'E, 21 Aug 2002, *F. Miyamoto* et al. *20220226* (E00238922); **Central**: **Bagmati Zone**: [Rasuwa Distr.] Langsisa Kharka, 15000 ft a.s.l., 15 Jun 1949, *O. Polunin 348* (BM).

####### General distribution.

Himalaya (rare in Central Himalaya) and Tibet (common). The populations from Tian Shan and Pamir belong to another (undescribed) species (Sukhorukov 2014).

#### Tribe Anserineae Dumort., Fl. Belg.: 20 (1827)

##### 6. Blitum

Taxon classificationPlantaeCaryophyllalesChenopodiacea

L., Sp. Pl.: 4 (1753)

Blitum L., Sp. Pl.: 4 (1753). **Lectotype** (designated by Britton and Brown 1913): Blitumcapitatum L.
=Morocarpus Boehmer in Ludwig, Def. Gen. Pl., ed. 3: 385 (1760);
=Anserina Dumort., Fl. Belg.: 21 (1827); **Type**: Anserinabonus-henricus (L.) Dumort. (≡Blitumbonus-henricus (L.) Reichenb.).
=Monolepis Schrad., Ind. Sem. Hort. Gotting.: 4 (1830). **Type**: Monolepistrifida Schrad., Ind. Sem. Hort. Gotting.: 4 (1830) =Monolepisnuttalliana (Schult.) Green;
=Agathophytum Moq., Ann. Sci. Nat. Bot., ser. 2(1): 291 (1834). **Type**: Agathophytumbonus-henricus (L.) Moq. (≡Blitumbonus-henricus);
=Scleroblitum Ulbr. in Engler & Harms, Nat. Pflanzenfam., ed. 2, 16c: 495 (1934). **Type**: Scleroblitumatriplicinum (F.Muell.) Ulbr. (≡Blitumatriplicinum F.Muell.).

###### Description.

Annual or perennial herbs, glabrous or covered with bladder hairs (scattered glandular hairs may be present). Leaves long-petiolate with triangular, hastate or rhombic blades; basal leaves often in a rosette. Inflorescences leafy or aphyllous in the upper part. Flowers in dense glomerules, bisexual or sometimes unisexual. Perianth of 1–5 free or insignificantly concrescent segments or perianth reduced, green, but sometimes fleshy and red or indurated in the fruiting stage. Stamens 1–5, usually equalling the perianth segments. Stigmas 2–3. Fruits with 1–2(3)-layered, smooth or mamillate pericarp, usually tightly adjoining the seed coat. Seed round or ovoid, red or reddish-black, without keel or with 2 blunt keels. Seed surface smooth or alveolate; testa cells without stalactites, sometimes with hair-like outgrowths. Seed embryo horizontally or vertically orientated or sometimes both embryo positions present on an individual (spatial heterospermy).

Approximately 14 species worldwide.

##### 1. Blitum virgatum

Taxon classificationPlantaeCaryophyllalesChenopodiacea

L., Sp. Pl.: 4 (1753)

Blitum virgatum L., Sp. Pl.: 4 (1753). **Lectotype** (designated by Jafri and Rateeb 1978): Herb. Linn. 14.2 (LINN! image available at http://linnean-online.org/60/).
=Morocarpusfoliosus Moench, Methodus: 342 (1794). **Type**: not designated.
≡Chenopodiumfoliosum (Moench) Asch., Fl. Brandenburg: 572 (1864).

###### Description.

Annual or short-lived perennial up to 60 cm tall, glabrous or covered with scattered glandular hairs in the upper part. Basal leaves numerous, up to 25 cm long, cauline leaves up to 20 cm, long-petiolate with triangular blades, truncate or slightly cuneate at base, margins erose-dentate or lobate. Inflorescence leafy to the apex; bracts dentate to lobate. Flowers in dense clusters up to 8 mm in diameter. Perianth segments 3–5, basally concrescent, green and membranous at anthesis but usually turning red and fleshy at the fruiting stage (sometimes remaining green). Pericarp adhering to the seed coat, hyaline, consisting of 1(2) very thin layers. Seeds ovoid, 1.0–1.2 × 0.7 mm, dark red, with a groove and two blunt keels.

###### Habitat.

Screes and sandy valleys, also cultivated (fruit conglomerations or “berries” are edible or used as a rouge); 2600–4400 m a.s.l.

###### Phenology.

Flowering: June-August; fruiting: July-September.

###### Distribution.

See Fig. 18.

**Figure 18.**
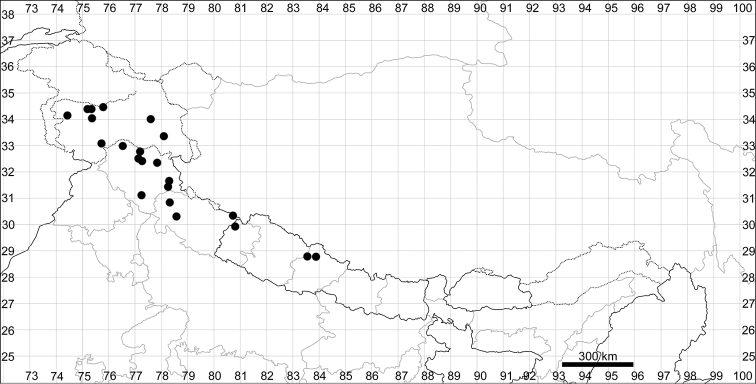
Distribution map of *Blitumvirgatum*.

###### Specimens examined.

**INDIA**: **Jammu & Kashmir** (selected specimens): Gulmarg, 8000–9000 ft a.s.l., 28 Jul 1892, *J.F. Duthie* s.n. (LE); Sonamarg, 8000 ft a.s.l., 30 Aug 1917, *R.R*. *Stewart 3415* (K); Sind valley, Gagangir vill. [Pojarat Gagangir], 8500 ft a.s.l., 21 Aug 1940, *F. Ludlow & G. Sherriff 7956* (BM, LE); Tangmarg, 6000 ft a.s.l., 17 Aug 1956, *Polunin 56-390* (B, BM, E); Jai hills, 30 May 1959, *T.A. Rao 9109* (BSD); Ladakh, Indus valley, nr Matho, 3800 m a.s.l., 10 Aug 1976, *H. Hartmann 55* (MSB137920); [Anantnag distr.] Liddar valley, nr Pahalgam, 5 Aug 1981, *Casimir* s.n. (MSB106102); Ladakh, Rupshu Region, Parang valley, 4580 m a.s.l., 20 Jul 2000, *L. Klimeš 949* (PRA); Ladakh, Dras Region, Umbo La, 4100–4200 m a.s.l., 12 Sep 2004, *L. Klimeš 4680* (PRA); Ladakh, Dras Region, Dras, 4250 m a.s.l., 13 Sep 2004, *L. Klimeš 4707* (PRA); **Himachal Pradesh** (selected specimens): Kinnaur [Distr.], Pangi, 18 Aug 1847, *T.T*. *Thomson* s.n. (K); Lahaul, Fhaga valley, Jun 1856, *Schlagintweit 2874* (LE); Lahaul, Rohtang La, 6 Aug 1916, *R.E*. *Cooper 5259* (E00276798); Simla, 29 Jul 1934, *N. Parmanand 721* (E); Lahaul, Sissu, 10100 ft a.s.l., 6 Jul 1938, *N.L*. *Bor 12373* (E, K); Lahaul, Khoksar, 10300 ft a.s.l., 3 Aug 1941, *N.L*. *Bor 16619* (K); Kinnaur Distr., Pangi, 2850 m a.s.l., 8 Jun 1962, *N.C. Nair 22556* (BSD); [Kinnaur Distr.] Sangla Kanda, 3000 m a.s.l., 22 Sep 1964, *N.C. Nair 34153* (BSD); Spiti Distr., Geching, 16 Aug 1994, *S.K. Murti 88440* (BSD); Simla Distr., Darcha vill., 3470 m a.s.l., 12 Aug 1998, *G.S. Goraya* et al. *1002* (BSD); Spiti Distr., Tangling Nalla, 4039 m a.s.l., 22 Sep 2002, *S. Singh* s.n. (BSD); **Uttarakhand**: Tehri Garhwal, 9000–1000 ft a.s.l., July 1883, *J.F. Duthie 92* (K); Kumaon, Kutti valley, Sep 1884, *J.F. Duthie 566 & 3328* (BM, E, K, LE); Uttarkashi Distr., 3700 m a.s.l., 4 Sep 1983, *U.C. Bhattacharyya 74851* (BSD); Uttarkashi Distr., Patangani vill., 1 Sep 2002, *P.K. Pusalkar 102128* (BSD);

**NEPAL**: **Far Western**: **Mahakali Zone**: Nampa Gadh, 25 Jul 1886, *J.F. Duthie 5914* (G); **Western**: **Dhaulagiri Zone**: Mustang Distr., Muktinath, 12500 ft a.s.l., 26 Jun 1954, *J. Stainton, W.R. Sykes & L.H.J. Williams 1431* (BM, LE); [Mustang Distr.] Larjung, S of Tukuche, Kali Gandaki [river], 8500 ft a.s.l., 10 Jun 1954, *J. Stainton, W.R. Sykes & L.H.J. Williams 1066* (BM, LE, E); [Mustang Distr.] Jomosom, 28°46'N, 83°54'E, 20 May 1974, *J.-F*. *Dobremez 3067* (BM, E00214397); Muktinath, 28°48'N, 83°52'E, 3500 m a.s.l., 22 Jul 1983, *H. Kanai 10631* (BM, E00156637).

###### General distribution.

Temperate Eurasia, native to the mountains of Europe, the Caucasus, South Siberia, Central Asia and Himalaya.

#### Tribe Dysphanieae Pax in Engler & Prantl, Nat. Pflanzenfam. 3, 1b: 92 (1889)

##### 7. Dysphania

Taxon classificationPlantaeCaryophyllalesChenopodiacea

Brown, Fl. Nov. Holland.: 411 (1810)

Dysphania littoralis R.Br., Prodr. Fl. Nov. Holland. 412 (1810) (**Type**).
=Roubieva Moq., Ann. Sci. Nat. Bot., ser. 2(1): 292 (1834); **Type**: Roubievamultifida (L.) Moq. (≡Dysphaniamultifida (L.) Mosyakin & Clemants).
=Ambrina Spach, Hist. Nat. Vég. 5: 295 (1836); **Lectotype** (Simón 1996): Ambrinaambrosioides (L.) Spach (≡Dysphaniaambrosioides (L.) Mosyakin & Clemants).
=Botrydium Spach, Hist. Nat. Vég. 5: 298 (1836) nom. illegit., non Wallroth (1815). **Lectotype** (Scott 1978): Botrydiumaromaticum Spach (=Dysphaniabotrys (L.) Mosyakin & Clemants);
=Neobotrydium Moldenke, Amer. Midl. Naturalist 35: 330 (1946). **Type**: Neobotrydiumbotrys (L.) Moldenke, Amer. Midl. Naturalist 35: 330 (1946) (≡Dysphaniabotrys (L.) Mosyakin & Clemants);

###### Description.

Aromatic annuals or, rarely, perennial herbs and small subshrubs covered with glandular hairs, subsessile yellow or orange glands and simple white hairs. Leaves alternate, entire, lobate or pinnatisect. Inflorescence lax or dense, leafy or not, consisting of cymes that are often reduced to one flower only. Perianth segments 2–5, free or variously connate, the midrib usually with keel. Stamens 1–5. Styles 2, free or basally connate. Fruit subglobose or rarely flattened, 0.3–1.5 mm, its surface reticulate or papillate, rarely smooth, dark coloured but often with whitish longitudinal stripes. Pericarp adjoining the seed coat, rarely bursting, hyaline, consisting of 1–2 very thin layers. Seed reddish or reddish-black, sometimes brown, its testa (outer seed coat layer) lacking vertical tannin-like deposits called “stalactites” in cross-section. Embryo horizontal or vertical, sometimes in both positions within one plant (spatial heterospermy).

More than 50 species worldwide, mostly in the tropics and subtropics. According to the first comprehensive molecular phylogeny (Kadereit et al. in prep.), the genus *Dysphania* is monophyletic and there is no reason to accept the genera *Neobotrydium* or *Ambrina* as was recently proposed (Zhang and Zhu 2016, Zhu and Sanderson 2017). All native species are closely related and belong to a large clade consisting of Eurasian and African representatives. The mountainous areas of Himalaya and Tibet are now considered one of the four centres of *Dysphania* diversity, together with Australia, South America and East Tropical Africa (Sukhorukov and Kushunina 2014, Sukhorukov et al. 2016a). The evident structural fruit and seed dimorphism in *Dysphania* (*D.tibetica*) is stated here for the first time.

##### Key to species

**Table:** 

1	Annuals or short-leaved perennials up to 1.5(2.0) m tall, growing mostly at elevations up to 2300 m; inflorescence dense, spike-like; perianth half connate or nearly so, enclosing the fruit completely and falling off with the fruit; pericarp covered with glandular hairs in its upper part	**1. *D.ambrosioides***
–	Annuals, mostly much smaller, growing at higher elevations; inflorescence loose, thyrsoid; perianth almost free or its segments concrescent up to 1/3 of their length, persistent at fruiting; pericarp with short papillae (visible at high magnification)	**2**
2	Inflorescence branches often terminate with short flattened aristae; subsessile glands scattered; fruits dimorphic (subspherical and ovoid)	**7. *D.tibetica***
–	Inflorescence branches without aristae; glands always present and abundant; fruits (sub)spherical	**3**
3	Leaves entire, sinuate or lobate; seed embryo usually vertical	**2. *D.himalaica***
–	Leaves lobate, pinnatifid or pinnatisect, very rarely almost entire and crisp; seed embryo horizontal	**4**
4	Leaf petioles short (up to 1.0 cm); inflorescence spike-like (with reduced or short lateral branches); perianth with numerous large simple stout hairs up to 0.3 mm, the bases of which can be concrescent, resulting in 2−3-furcate tips; fruits 0.5–0.6 mm in diameter	**6. *D.geoffreyi***
–	Leaf petioles (at least of basal leaves) longer than 0.5 cm; inflorescence not spike-like, with well-expressed lateral branches; stout simple hairs on the perianth (if present) usually not concrescent at the base; fruits 0.6–0.8 mm	**5**
5	Perianth with simple and (stalked) glandular hairs	**6**
–	Perianth with simple hairs and subsessile glands; glandular hairs on perianth absent or very scattered (not visible at lower magnification)	**7**
6	Plant robust, up to 100(120) cm, stem highly branched, densely covered (especially in the upper part) with both simple and glandular hairs up to 1 mm long	**8. *D.neglecta***
–	Plants up to 50 cm, stem mostly branched in the lower part with scattered hairs up to 0.3 mm long	**5. *D.botrys***
7(5)	Simple hairs on perianth segments scattered (mostly localised near the segment tips) or even absent; partial inflorescences recurved, umbel-like	**4. *D.kitiae***
–	Simple hairs on perianth segments abundant	**8**
8	Leaves pinnatisect; glands yellow and/or orange	**3. *D.bhutanica***
–	Leaves pinnatifid or lobate; plant with yellow glands	**9. *D.nepalensis***

###### 1. Dysphania ambrosioides

Taxon classificationPlantaeCaryophyllalesChenopodiacea

(L.) Mosyakin & Clemants, Ukr. Bot. Zhurn. 59(4): 382 (2002)

Fig. 19

≡Chenopodiumambrosioides L., Sp. Pl.: 219 (1753). **Lectotype** (designated by Brenan 1954): Herb. Linn. 313.13 (LINN! image of the lectotype available at http://linnean-online.org/3087/).
≡Ambrinaambrosioides (L.) Spach, Hist. Nat. Veg. 5: 297 (1836);
≡Vulvariaambrosioides (L.) Bubani, Fl. Pyren. 1: 178 (1897);
≡Botrysambrosioides (L.) Nieuwl., Am. Midl. Naturalist 3: 275 (1914);
≡Neobotrydiumambrosioides (L.) M.L.Zhang & G.L.Chu [Zhu], Pl. Diversity 38(6): 327 (2016).

####### Taxonomic notes.

In the species circumscription, we follow the opinion of Mosyakin and Clemants (2002) and Iamonico (2011) and maintain separation from *D.anthelmintica* (L.) Mosyakin & Clemants.

####### Description.

Annual or short-lived perennial up to 1.5(2.0) m, very aromatic, covered (at least the young parts of the plant) with curved simple hairs, yellow (subsessile) glands and glandular hairs with a prominent stalk. Leaves long-petiolate, 5.0–16.0 × 1.0–4.0 cm, elliptic-oblong or lanceolate, dentate or sinuate; upper leaves often entire. Inflorescence usually highly branched, spike-like, bracteate or aphyllous in the upper part. Flowers sessile. Perianth segments (4)5, green, ca. 1.0 mm long, (nearly) half concrescent, concave near the apex, completely enclosing the fruit (Fig. 20A). Pericarp thin, hyaline, tightly adjoining the seed coat but separating from it when rubbed, in its upper part covered with glandular hairs (up to 0.12 mm long) with a large terminal cell (Fig. 20B). Seed dark red or almost black, 0.7 × 0.5–0.6 mm, not keeled. Embryo horizontal, rarely oblique or vertical.

**Figure 19.**
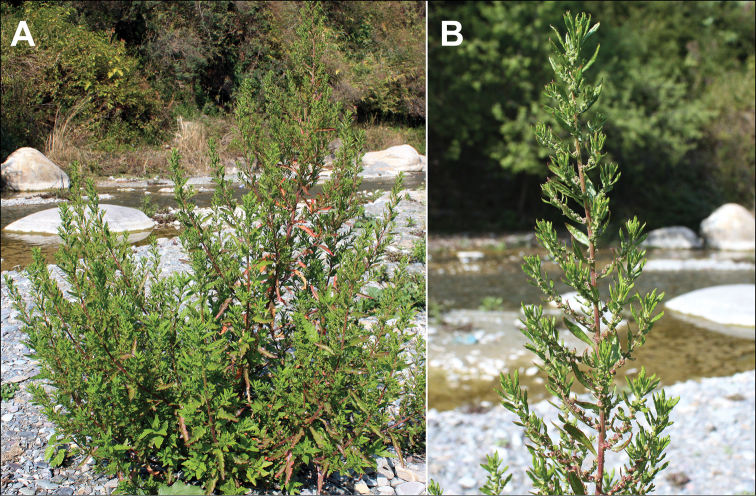
*Dysphaniaambrosioides*: **A** general view **B** close-up of the inflorescence branch. Photographs by A. Sukhorukov (Dehradun, Uttarakhand, India, February 2018).

**Figure 20.**
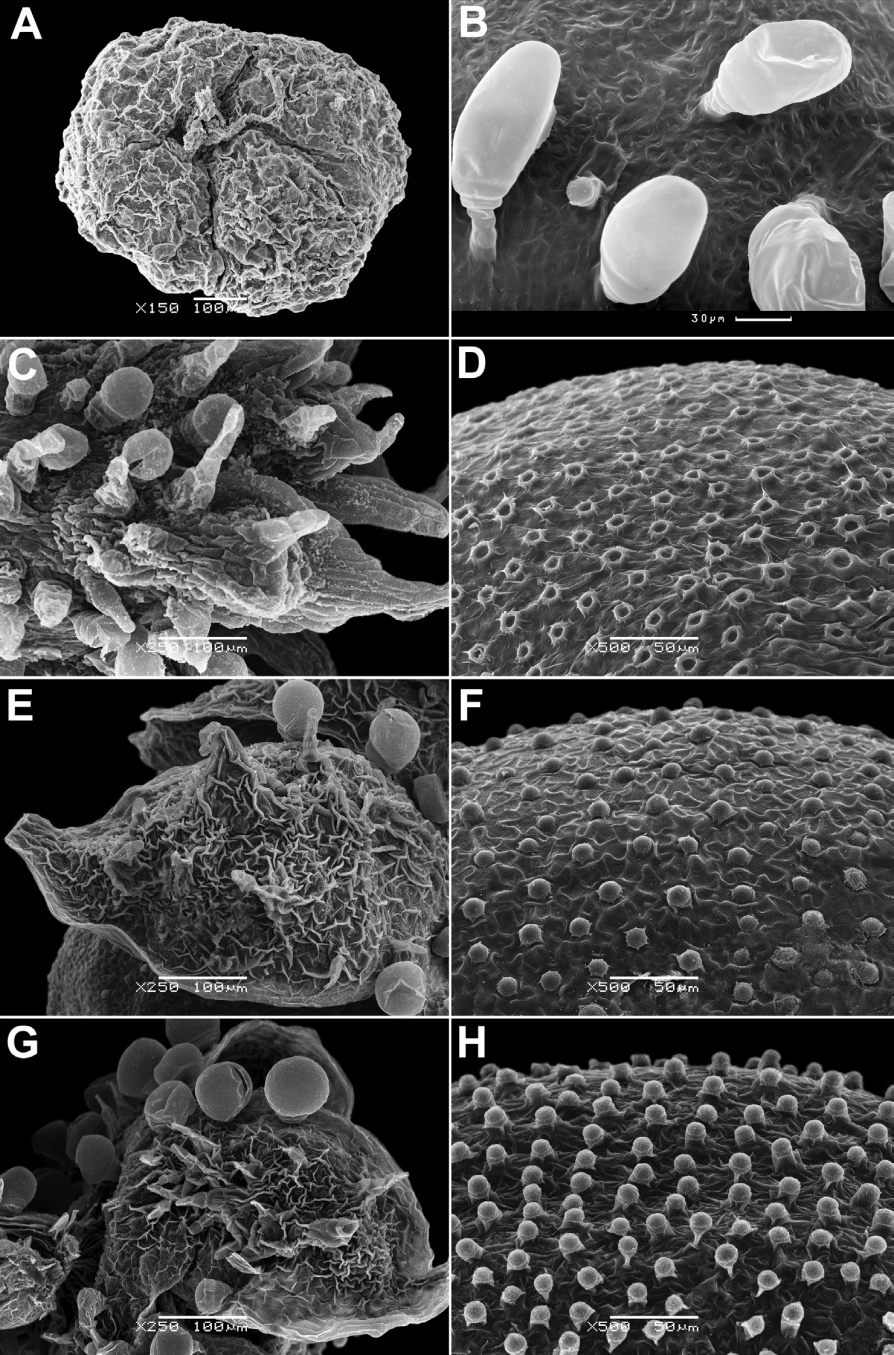
SEM micrographs: **A** perianth of *Dysphaniaambrosioides***B** pericarp of *Dysphaniaambrosioides***C** perianth of *D.himalaica***D** pericarp of *D.himalaica***E** perianth of *D.bhutanica***F** pericarp of *D.bhutanica***G** perianth of *D.kitiae***H** pericarp of *D.kitiae*. Magnification: 150× (**A**), 250× (**C, E, G**), 500× (**B, D, F, H**).

####### Habitat.

Disturbed places and riversides, gravelly substrates; 0–2300 m a.s.l.

####### Phenology.

Flowers and fruits year-round. Fruits develop only at elevations up to 2300 m. At higher elevations, the plants, even if occasionally present, do not produce flowers and fruits (Sukhorukov and Kushunina 2014).

####### Distribution.

See Fig. 21.

**Figure 21.**
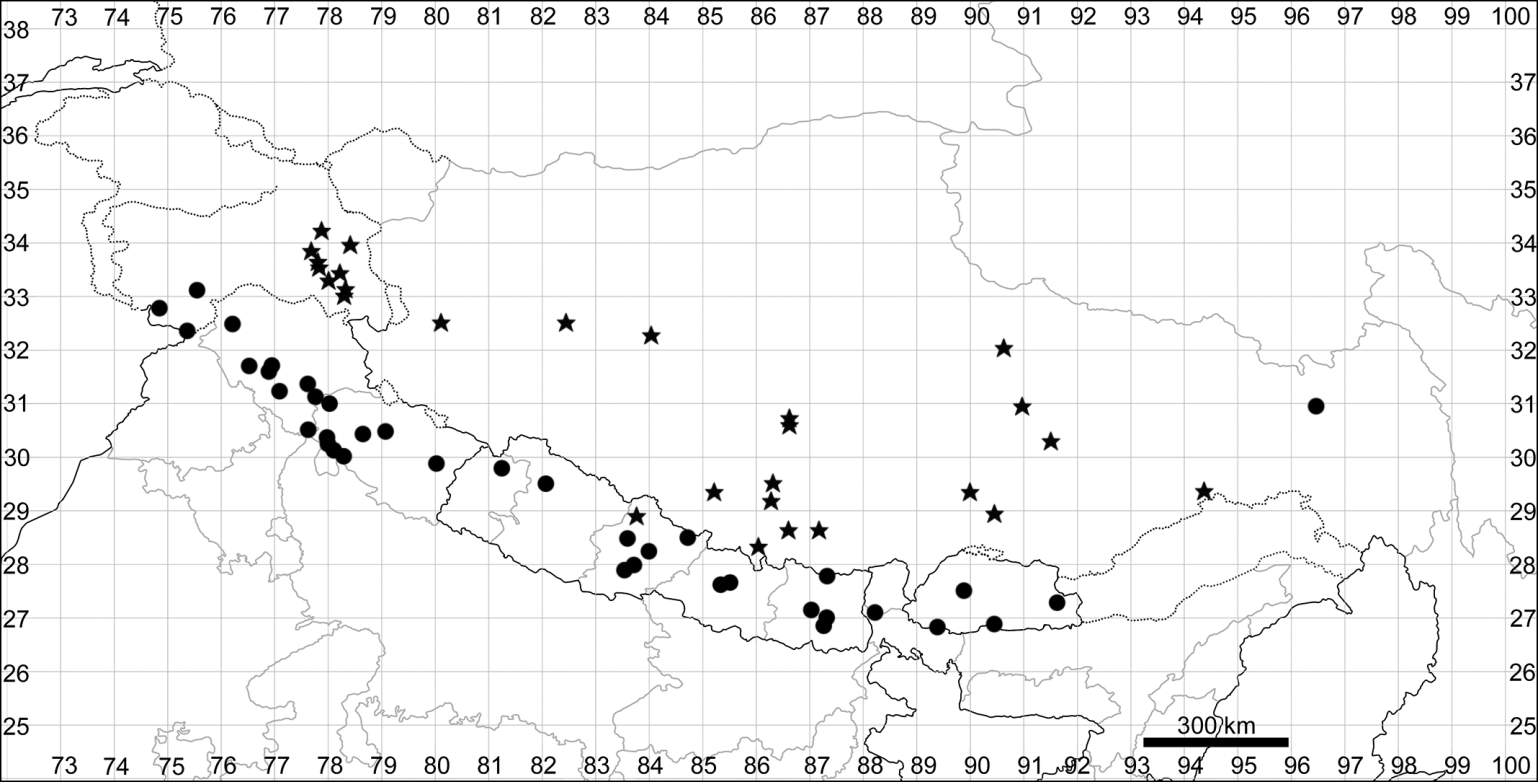
Distribution map of *Dysphaniaambrosioides* (circles) and *D.himalaica* (stars).

####### Specimens examined.

**BHUTAN**: Torsa river, Phuntsholing, 200 m a.s.l., 1 May 1979, *A.J.C. Grierson & D.G. Long 743* (E); Gaylegphug [Gelephu], 300 m a.s.l., 29 May 1979, *A.J.C. Grierson & D.G. Long 1432* (E00168232, K); Tashigang, 27°19'N, 91°34'E, 1330 m a.s.l., 18 Jun 1979, *A.J.C. Grierson & D.G. Long 2066* (E00168230, K); Wangdue Phodrang, 1450 m a.s.l., 12 Jul 1979, *A.J.C. Grierson & D.G. Long 2674* (E00037493); Punakha Distr., Wangdue Phodrang, 1250 m a.s.l., 12 Jul 1991, *C. Parker 4962* (E);

**CHINA: Xizang** (new record for Xizang): **Nyingchi Prefecture**: Bomi County, Tangmai (Tongmai) vill., 2300 m a.s.l., 14 Aug 1960, *anonym 756* (KUN0587049);

**INDIA: Jammu & Kashmir**: Jammu city, 29 Mar 1966, *I.A*. *Gubanov 88* (MW); nr Jakhbar vill., 5 Aug 1986, *P.K. Hajra 82448* (BSD); Doda, 16 Aug 1986, *B.D. Naithani 82708* (BSD); **Himachal Pradesh**: nr Rampur Bushahr, 21 Aug 1963, *N.C. Nair 28289* (BSD); Jeoni, 1400 m a.s.l., 26 Aug 1963, *N.C. Nair 29924* (BSD); Hatkoti, 14 Jul 1965, *N.C. Nair 35764* (BSD); Bilaspur Distr., Ner Chowk, 1000 m a.s.l., 24 Jun 1972, *P. Uotila 18175* (H1101254); Rakh, 1175 m a.s.l., 3 Jul 1974, *B.M. Wadhwa 49482* (BSD); Hamirpur, 1 Aug 1977, *M.V. Vishwanathan 61408* (BSD); Baggi canal area, Mandi, 17 Jun 1984, *H.J. Chowdhery* et al. *75811* (BSD); Simla Distr., Suni, 2 Jun 1986, *P.C. Pani 80906* (BSD); Sirmaur Distr., Sataun, 10 Aug 1986, *R.S. Karki 82287* (BSD); **Sikkim**: [without exact location] 22 Jul 1874, *Treuler 383* (K); [without exact location] 4000 ft a.s.l., 11 Jun 1884, *anonym* s.n. (G); Feb 1909, *anonym 525* (M); **Uttarakhand** (selected specimens): Dehradun, Sep 1930, *Krishnan 131* (K); Dehradun, 26 Oct 1956, *T.A. Rao 1055* (BSD); Dehradun, 9 Jun 1972, *B.D*. *Naithani 3623* (BR); Kumaon, 7 Sep 1973, *anonym 52430* (BSD); Mussoorie, 12 Aug 1978, *G. Pongrohi* et al. *64940* (BSD); Kumaon, Guptakashi, Sep 1978, *G. Panigsahi 65168* (BSD); Tehri Garhwal Distr., Chamiyala, 10 Mar 1979, *A.K. Goel 65955* (BSD); Tehri Distr., Jogiana, 640 m a.s.l., 19 Sep 1979, *A.K. Goel 64717* (BSD); Tehri Garhwal [Distr.], Athoor [Athhoorwala], 7 Jun 1983, *B.S. Aswal 11998* (G452574, K); Uttarkashi Distr., Mori, 17 Sep 1995, *S. Singh 89835* (BSD); Dehradun, Guchhupani, 16 Feb 2018, *A. Sukhorukov 76* (K, MW);

**NEPAL**: **Far-Western**: **Seti Zone**: Bajhang Distr., 19 Aug 1991, *M. Suzuki* et al. *9160762* (BM); **Midwestern**: **Karnali Zone**: Mugu Distr., 1 km S of Rara Lake, 2900 m a.s.l., 29 Sep 2013, *A. Sukhorukov 108* (MW); **Western**: **Dhaulagiri Zone**: Myagdi [Distr.], Annapurna conservation area, trekking route Jomosom-Ghorepani, Tatopani vill., Kali Gandaki river, 1200 m a.s.l., 11 May 2010, *A. Sukhorukov 120* (W2010-0015369, E00428104); **Gandaki Zone**: [Syangja Distr.] Andhi Khola, 2500 ft a.s.l., 1 Oct 1954, *J. Stainton, W.R. Sykes & L.H.J. Williams 8700* (BM); [Kaski Distr.] Pokhara, 17 Oct 1973, *Schwabe* s.n. (B); Gorkha Distr., Gorkha, nr Gorkha Kali temple, 28°00'N, 84°37'E, 1200 m a.s.l., 22 Aug 2008, *H. Ikeda* et al. *20812007* (E00640133); **Lumbini zone**: [Palpa Distr.] Tansing [Tansen], 3000 ft a.s.l., 5 Oct 1959, *J. Stainton, W.R. Sykes & L.H.J. Williams 8747* (E, BM, LE); **Central**: **Bagmati Zone**: Phulchoki, 8500 ft a.s.l., 8 Sep 1967, *Manadhar 7397* (BM); Dhulikel, 30 Oct 2000, *C.R. Fraser-Jenkins* et al. *4631* (H1723699); [Kathmandu Distr.] Godawari, 1400 m a.s.l., 15 Nov 2005, *A. Sukhorukov* s.n. (H1735140, MW); **Eastern**: **Koshi Zone**: [Dhankuta Distr.] Dhankuta, 26°58'N, 87°20'E, 980 ft a.s.l., 6 Jul 1969, *L.H.J*. *Williams 1155* (BM); Dhankuta Distr., 27°00'N, 87°15'E, 2 Jul 1988, *M. Suzuki* et al. *8820036* (BM); [Sunsari Distr.] Dharan, 26°50'N, 87°20'E, 1500 ft a.s.l., 3 Sep 1967, *L.H.J*. *Williams & J. Stainton 8341* (BM); Sankhuwasabha Distr., nr Hatiya, Upper Arun valley, 27°44'N, 87°20'E, 1600 m a.s.l., 13 Oct 1991, *D.G*. *Long* et al. *769* (E); Bhojpur distr., 1610 m a.s.l., 1 Nov 1995, *M. Mikage* et al. *9558269* (E00229908).

####### General distribution.

The Americas (native to South and Central America), Eurasia, Africa and Australia. Frequently found in many parts of Central Himalaya.

###### 2. Dysphania himalaica

Taxon classificationPlantaeCaryophyllalesChenopodiacea

Uotila, Willdenowia 43(1): 68 (2013).

Chenopodium himalaicum Klimesh in herb. PRA (nomen). **Holotype**. INDIA, [Jammu & Kashmir], Ladakh, Region Indus valley, Stot (E) [Stod River valley], Nyi [Nior Nis; Njurnis] to Neboche, 33°28'13"N, 78°14'25"E, 4600–4700 m a.s.l., 2 Sep 2005, code 05-29-16, *L. Klimeš 6175* (PRA! isotypes (5) PRA! H1758789!).
=Neobotrydiumlongii [*langii*] G.L.Zhu in Zhu & Sanderson, Gen. & New Evol. System World Chenopod.: 334 (2017), syn. nov. **Holotype**. CHINA, Tibet [Xizang], Mami division, Gaize Xian (Gêrzê County), Chacuo, 4500 m a.s.l., 8 Sep 1976, *K.-Y. Long [Lang] 10153* (PE00511004!).

####### Taxonomic notes.

During the last visit of the first author (AS) to the herbarium of the Institute of Botany of the Chinese Academy of Sciences (PE) in early 2017, the type of *Neobotrydiumlongii* had not yet been selected and the specimen without a new determination was kept with all other *Dysphania* in a folder under the old name “*Chenopodiumfoetidum*”. The synonymisation of *N.longii* [*langii*, named after K.-Y. Lang] with *D.himalaica* is made here for the first time based on the re-identification of specimen PE00511004 by AS.

####### Description.

Annual up to 15(20) cm, mostly branched from the base. Leaves appressed to the stem, short-petiolate, up to 4.0 × 1.0 cm, narrowly oblong or lanceolate, entire, sinuous or slightly lobate, green, with scattered simple hairs and subsessile yellow glands. Inflorescence leafy in the lower part, covered with short, semi-appressed white simple hairs (up to 0.4 mm long) and scattered subsessile yellow glands. Perianth segments (4)5, 0.9–1.2 × 0.25–0.40 mm, almost free, somewhat swollen near midrib with a short, white or reddish mucro(s) at the tip, their dorsal part with stout simple hairs (up to 0.15 mm), often red at the segment tips and scattered glands up to 0.1 mm (Fig. 20C). Pericarp whitish, minutely papillate, separating from the seed (Fig. 20D). Fruit 0.7–0.9 × 0.7 mm, subspherical; seed reddish, keeled; embryo vertical or sometimes horizontal (spatial heterospermy).

####### Habitat.

Gravelly slopes and ruderal sites at elevations of 3000–4900 m a.s.l.

####### Phenology.

Flowering: August-September; fruiting: September-October.

####### Distribution.

See Fig. 21.

####### Specimens examined.

**CHINA**: **Xizang**: **Ngari Prefecture**: Gêrzê (Gaize) County, 4250 m a.s.l., Aug 1972, *Li 069* (PE00510701, PE00510702); Gêrzê (Gaize) County, Datan, 4450 m a.s.l., 6 Sep 1974, *Biological Institute Tibet Expedition Team 4295* (XJBI00005533); Gêrzê (Gaize) County, 4500 m a.s.l., 8 Sep 1976, *K.-Y*. *Lang 10153* (PE00511004! holotype of *Neobotrydiumlongii*); Gê’gyai (Geji) County, Yanhu District, 4350–4500 m a.s.l., Aug 1977, *Qinghai-Tibet Team Vegetation Group 13531* (PE00235088); **Nagqu Prefecture**: [Nyima (Nima) County] Changthang, Dangra Yum Tso, 30°43'N, 86°35'E, 4590 m a.s.l., 9 Sep 2003, *G. & S. Miehe 03-081-05* (herb. Miehe); [Nyima (Nima) County] Changthang, S of Dangra Yum Tso, Targo river S of Targo Shang, 30°35'N, 86°39'E, 4765 m a.s.l., 10 Sep 2003, *G. & S. Miehe 03-089-01* (KAS); [Amdo (Anduo) County] Lake Xiketang, 32°03'N, 90°40'E, 4720 m a.s.l., 15 Aug 2005, *Nölling & Hanspach NX05-108-01* (herb. Miehe); **Xigazê Prefecture**: Upper Arun valley, N of Xegar (Tingri), 28°37'N, 87°10'E, 5 Oct 1989, *B. Dickoré 5919* (KAS) & *5922* (MSB144244); Side valley, N of Nyalam, Koya, 28°22'N, 86°02'E, 4460 m a.s.l., 24 Aug 1999, *G. & S. Miehe & Koch 99-150-28* (herb. Miehe); New Tingri, 28°35'N, 86°38'E, 4400 m a.s.l., 1 Sep 1999, *S. & G. Miehe & Koch 99-189-08* (herb. Miehe); [Saga County] Upper Yarlung Tsangpo, 29°12'N, 86°16'E, 4850 m a.s.l., 31 Aug 2003, *G. & S. Miehe 03-057-18* (herb. Miehe); [Ngamring (Angren) County] Raka Tsangpo valley, 29°30'N, 86°17'E, 4760 m a.s.l., 2 Sep 2003, *G. & S. Miehe* 03-062-24 (herb. Miehe); [Saga County] Saga Dzong, Upper Yarlug Tsangpo, 29°21'N, 85°14'E, 28 Aug 2003, *G. & S. Miehe 03-043-23* (KAS); **Lhasa City**: NE of Lhasa, Reting Monastery, 30°18'N, 91°31'E, 4300 m a.s.l., 10 Sep 1995, *G.& S. Miehe 95-49-10* (herb. Miehe); Nyêmo (Nimu) County, Yarlung Tsangpo gorge, 29°21'N, 89°59'E, 4300 m a.s.l., 7 Sep 1998, *G. & S. Miehe 98-09205* & *98-09412* (herb. Miehe); [Damxung (Dangxiong) County] Lake Namtso, 30°55'N, 90°58'E, 4760 m a.s.l., 1 Sep 2005, *Nölling & Hanspach NX05-188-06* (herb. Miehe); **Shannan Prefecture**: [Nagarzê (Langkazi) County] Yamco Yumco (Yamzho Yumco, the Yamdrok Lake), Dzamtshu, 28°58'N, 90°27'E, 4450 m a.s.l., 12 Sep 1995, *G. & S. Miehe 95-52-22* (herb. Miehe); **Nyingchi Prefecture**: [Mainling (Milin) County] Lower Tsangpo valley, Tsela Dzong, 29°21'N, 94°21'E, 3050 m a.s.l., 25 Sep 1989, *B. Dickoré 5748* (MSB144246);

**INDIA**: **Jammu & Kashmir**: [nr Parang river] Haule, 14000 ft a.s.l., 17 Sep 1847, *T.T*. *Thomson* s.n. (K); Leh, Angkung to Puga, 33°14'N, 78°16'E, 4550 m a.s.l., 8 Sep 1999, code 99-27-9, *L. Klimeš 778* (H, PRA); Kiameri La to Rumtse vill. along Kyammar Lungpa, 33°35'N, 77°49'E, 4350 m a.s.l., 15 Sep 1999, code 99-34-3, *L. Klimeš 830* (H, PRA); Rupshu Region, Samad Rakchan, Polokongka valley, 33°16'24"N, 78°6'6"E, 4660–4750 m a.s.l., 5 Aug 2001, code 01-8-11, *L. Klimeš 1255* (PRA); Zhung (Leh), Gya to Lato, 33°40'2"N, 77°43'9"E, 4060–4070 m a.s.l., 5 Sep 2001, code 01-38-12, *L. Klimeš 1539* (PRA); Samad Rakchan, Thangmar, 33°20'4"N, 78°1'8"E, 4590 m a.s.l., 5 Aug 2001, code 01-8-8, *L. Klimeš 1271* (PRA); Ladakh, Rumtse, 4220–4230 m a.s.l., 5 Sep 2001, *L. Klimeš 1582* (PRA); Indus valley, Zhung (Leh), Chogdo to Chukirmo, 33°49'24"N, 77°38'9"E, 4180–4310 m a.s.l., 8 Sep 2001, code 01-41-10, *L. Klimeš 1627* (PRA); Lukung, 33°59'5"N, 78°24'6"E, 4300 m a.s.l., 9 Sep 2002, code 02-39-10, *L. Klimeš 6627* (PRA); Ladakh, Pangong Region, Parma vill., 4550 m a.s.l., 23 Sep 2003, *L. Klimeš 3580* (PRA); Zhung Stot (E), Stot (E); Shyok Region, Wari La, confluence of Lurten Lungpa and Lazun Lungpa, 34°14'9"N, 77°51'8"E, 3840 m a.s.l., 15 Sep 2001, code 01-47-40, *L. Klimeš 1868* (PRA); Sumdu Gonma to Kiagar La, 33°10'12"N, 78°21'30"E, 4690 m a.s.l., 7 Sep 2003, code 03-26-3, *L. Klimeš 3461* (PRA); Tso Moriri, Lunglung valley, 33°2'30"N, 78°18'E, 4700 m a.s.l., 8 Sep 2003, code 03-27-5a, *L. Klimeš 3476* (PRA); Samad Rakchan, Thukje vill. to Nyamur, 33°20'13"N, 78°1'40"E, 4560 m a.s.l., 9 Sep 2005, code 05-36-2, *L. Klimeš 6270* (PRA);

**NEPAL**: **Western**: **Dhaulagiri Zone**: Mustang Distr., Chalungpa, Lower Jeula forest, 28°54'N, 83°45'E, 3410 m a.s.l., 28 Aug & 8 Sep 2001, *S. & G. Miehe & Koch 01-087-27 & 01-119-03* (herb. Miehe).

####### General distribution.

Himalaya and Tibet (India, Nepal and China). Common in Jammu & Kashmir and many parts of Tibet (L. Klimeš, G. & S. Miehe, pers. comm.); probably rare in Nepal.

###### 3. Dysphania bhutanica

Taxon classificationPlantaeCaryophyllalesChenopodiacea

Sukhor., Willdenowia 42(2): 171 (2012)

####### Holotype.

BHUTAN, Thimphu distr., Lango, near Paro, frequent weed in apple and other crops, 2300 m a.s.l., 29 Jun 1992, *C. Parker 7263* (E00051983! image available at http://data.rbge.org.uk/herb/E00051983).

####### Description.

Annual up to 100 cm. Stem covered with short simple hairs and subsessile, orange or yellow glands. Leaves pinnatisect, 2.0–9.0 × 1.0–2.5 cm, long-petiolate; their segments oblong or lanceolate, sinuate to lobed, usually densely covered with short simple hairs intermixed with scattered glands. Inflorescence up to 20 cm long, leafy at least in basal and middle parts with scattered curved simple (up to 0.2 mm) hairs and subsessile glands. Perianth segments 5, almost free, 0.7–0.9 × 0.35–0.40 mm; their dorsal part with stout simple hairs (up to 0.08 mm) and orange (rarely yellow) glands (Fig. 20E). Fruit 0.6–0.7 × 0.5 mm in diameter, subspherical, pericarp minutely papillate (Fig. 20F). Seed blackish, keeled; embryo horizontal.

####### Habitat.

Grassy hill slopes and disturbed areas; 2200–3800 m a.s.l.

####### Phenology.

Flowering: June-August; fruiting: July-September.

####### Distribution.

See Fig. 22.

**Figure 22.**
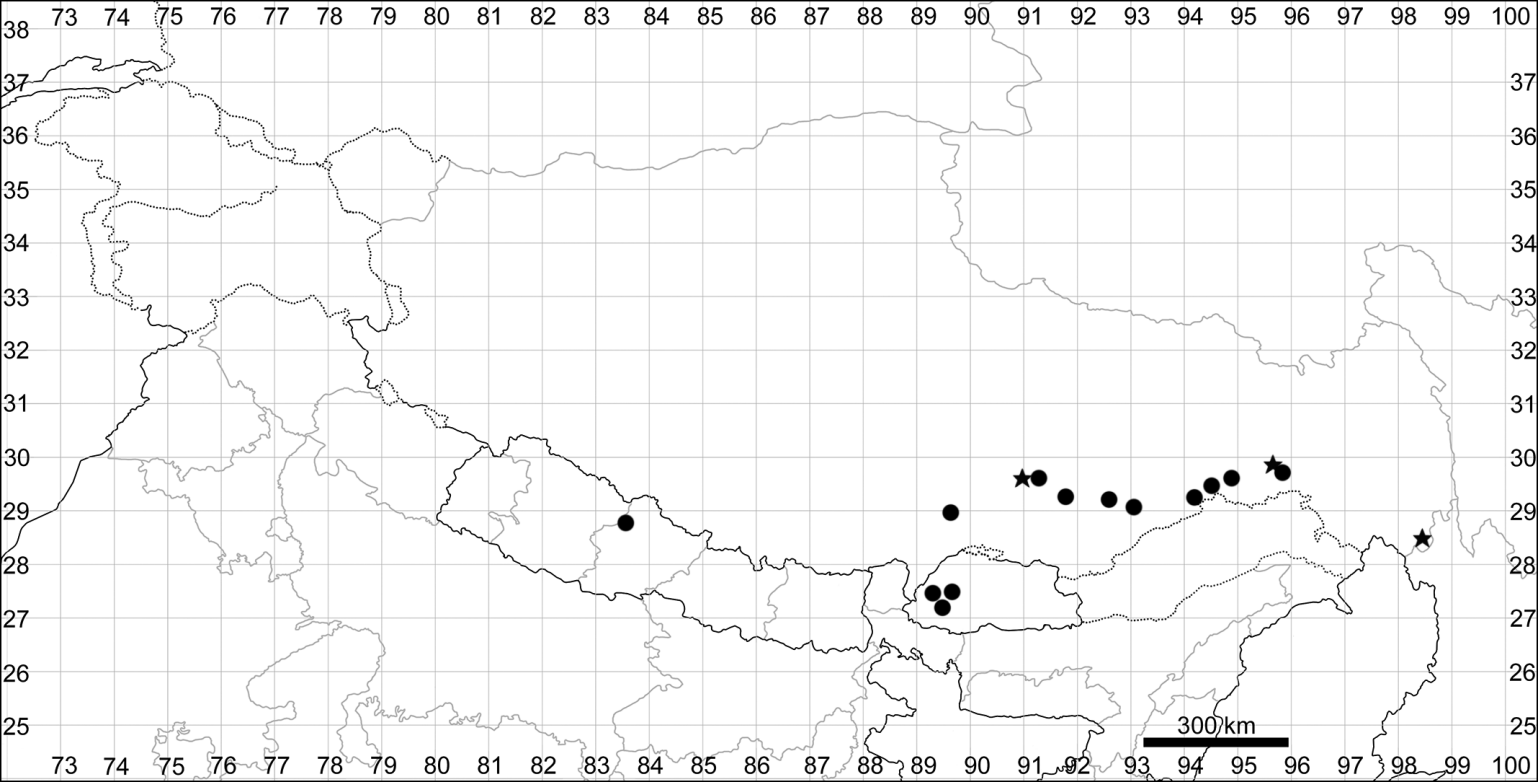
Distribution map of *Dysphaniabhutanica* (circles) and *D.kitiae* (stars).

####### Specimens examined.

**BHUTAN**: [Thimphu Distr.] Thimphu, 8000 ft a.s.l., 9 Aug 1914, *R.E. Cooper 3370* (BM, E00151685); Thimphu, 2408 m a.s.l., 10 Aug 1971, *R. Bedi 657* (K); Thimphu Chu, below Taba, 27°30'N, 89°38'E, 2300 m a.s.l., 22 Jul 1979, *A.J.C. Grierson & D.G. Long 2828* (E00151632, K); Thimphu Distr., Chapcha, 2200–2400 m a.s.l., 1 Jul 1992, *C. Parker 7270* & *7271* (E00051982, E00051981);

**CHINA: Xizang**: **Xigazê Prefecture**: Gyangtse, 1904, *H.J. Walton* s.n. (CAL, P04992918); **Lhasa City**: Lhasa, 3800 m a.s.l., 16 Aug 1965, *Zhang & Lang 1532* (PE00510965); Lhasa, 29°41'N, 91°08'E, 3800 m a.s.l., 13 Aug 1997, *G. & S. Miehe & al*. *97-013-10* (herb. Miehe); **Shannan Prefecture**: Gyaca (Jiacha) County, 3300 m a.s.l., 2 Sep 1972, *Tibet Chinese Herbal Medicine Survey Team 4572* (PE00511073); [Nêdong (Naidong) County] Zêtang (Zedang), 11 Aug 1977, *B.Z.Guo* et al. *22359* (KUN0587209); Nêdong (Naidong) County, Luoqiong vill., 29°15'57.23"N, 91°49'1.76"E, 3569 m a.s.l., 2 Sep 2012, *FLPH Tibet Expedition 12-0610* (PE); **Nyingchi Prefecture**: Tsangpo valley, 10000–11000 ft a.s.l., 5 Sep 1935, *F. Kingdon-Ward 12308a* (BM); Tsangpo valley, Tse, 9800 ft a.s.l., 31 May 1938, *F. Ludlow, G. Sherriff & G. Taylor 4585* (BM); Zhamo (Bomi) County, 2700 m a.s.l., 16 Jul 1965, *Zhang & Lang 312* (PE00511002); Mainling (Milin) County, Pai [Pa] town, 3000–3100 m a.s.l., 12 Sep 1974, *Qinghai-Tibet Team 74-4663* (PE00511046, PE00511047); Nang (Lang) County, 3100 m a.s.l., 4 Aug 1982, *Ni, Ciduo & Cidan 2744* (PE00511029); Mainling County, Qabnag (Qiangna), 22 Aug 1982, *Ni, Ciduo & Cidan 3109* (PE00511027);

**NEPAL**: **Western**: **Dhaulagiri Zone**: [Mustang Distr., Kali Gandaki river valley] Tukuche, 10500 ft a.s.l., 21 Aug 1954, *J. Stainton, W.R. Sykes & L.H.J. Williams 7356* (BM). The plant has yellow glands only and identification is based on the pinnatisect leaf shape.

####### General distribution.

E Himalaya and S Tibet.

###### 4. Dysphania kitiae

Taxon classificationPlantaeCaryophyllalesChenopodiacea

Uotila, Willdenowia 43(1): 75 (2013)

Dysphania kitiae Uotila, Willdenowia 43(1): 75 (2013). **Holotype**. CHINA, Gansu [Prov.], Monastery Chorten-tan, on sunny slopes on clay and rich soil, 7000 ft a.s.l., early September 1901, *V.F. Ladygin 596* (LE!).
=ChenopodiumaristatumL.formamuticum J.Q.Fu Fl. Loess-Plateaus Sin. 1: 610 (2000), nom. illegit.
=Neobotrydiumcorniculatum G.L.Chu & M.L.Zhang, Pl. Diversity 38(6): 325 (2016), syn. nov. **Holotype**: CHINA, Gansu [Prov.], Diebu Xian, Wangzang, foot of slopes, [date of collection not indicated in the protologue] *G.L. Chu 15618* (NWTC, n.v.).

####### Taxonomic notes.

The name ChenopodiumaristatumL.formamuticum (Fu 2000) is published with two holotypes and this is considered an uncorrectable error according to ICN (Turland et al. 2018), art. 40.6. Another name, *Neobotrydiumcorniculatum*, is synonymised here for the first time based on the following observations: (1) description of *N.corniculatum* and the drawing of the entire plant, especially perianth features, correspond with *Dysphaniakitiae*; (2) specimens kept at the PE and WUK herbaria and cited as *Dysphaniakitiae* by Sukhorukov and Kushunina (2014) are the same as those listed by Zhang and Zhu (2016) for *Neobotrydiumcorniculatum*. One of the specimens of *Neobotrydiumornithopodum* G.L. Chu & M.L. Zhang (Zhang and Zhu 2016), cited in the protologue (Hebei prov., leg. *Hsia 2448*, WUK!), is in fact *Dysphaniakitiae* (see citation of the specimens in Sukhorukov and Kushunina (2014)). We have not investigated the holotype of *Neobotrydiumornithopodum*, a species closely related to *Dysphanianepalensis*.

####### Description.

Annual up to 50 cm, strongly aromatic. Leaves 4.0–10.0 × 2.0–4.0 cm, long-petiolate, elliptic-oblong in outline, apex rounded. Inflorescence with indumentum consisting of scattered short simple hairs (up to 0.3 mm long) and subsessile glands. Perianth segments 5, basally connate, 1.0–1.2 × 0.5–0.6 mm; their dorsal part with yellow glands and scattered stout simple hairs (up to 0.1 mm), the latter mostly localised in the upper half of the segments (Fig. 20G). Fruit 0.7–0.9 mm, subspherical. Pericarp with minute conical papillae up to 25 μm (Fig. 20H); seed blackish; embryo horizontal.

####### Habitat.

Grassy slopes and waste areas at altitudes of 2000–3800 m a.s.l.

####### Phenology.

Flowering: July-September; fruiting: August-October.

####### Distribution.

See Fig. 22.

####### Specimens examined.

**CHINA: Xizang**: **Lhasa City**: W of Lhasa, 3800 m a.s.l., 15 Jun 1974, *Mountaineering Scientific Expedition Team 310* (HNWP42358); **Nyingchi Prefecture**: Zhamo (Bomi) County, 2700 m a.s.l., 16 Jul 1965, *Zhang & Lang 314* (PE00511015); Zayü (Chayu) County, Cawarong (Chawalong) Distr., 2800 m a.s.l., 28 Sep 1982, *Qinghai-Tibet Team 10836* (PE01063458).

####### General distribution.

South-West, Central and North-East China (for more, see Sukhorukov and Kushunina (2014)).

###### 5. Dysphania botrys

Taxon classificationPlantaeCaryophyllalesChenopodiacea

(L.) Mosyakin & Clemants, Ukr. Bot. Zhurn. 59(4): 383 (2002)

≡Chenopodiumbotrys L., Sp. Pl.: 219 (1753). **Lectotype** (Jafri and Rateeb 1978): Herb. Linn. 313.12 (LINN).
≡Vulvariabotrys (L.) Bubani, Fl. Pyren. 1: 177 (1897);
≡Botrydiumbotrys (L.) Small, Man. S.E. Fl.: 466 (1933);
≡Neobotrydiumbotrys (L.) Moldenke, Amer. Midl. Naturalist 35: 330 (1946);
≡Teloxysbotrys (L.) W.A.Weber, Phytologia 58(7): 477 (1985);
=Botrydiumaromaticum Spach, Hist. Nat. Veg. 5: 299 (1836).

####### Description.

**Figure 23.**
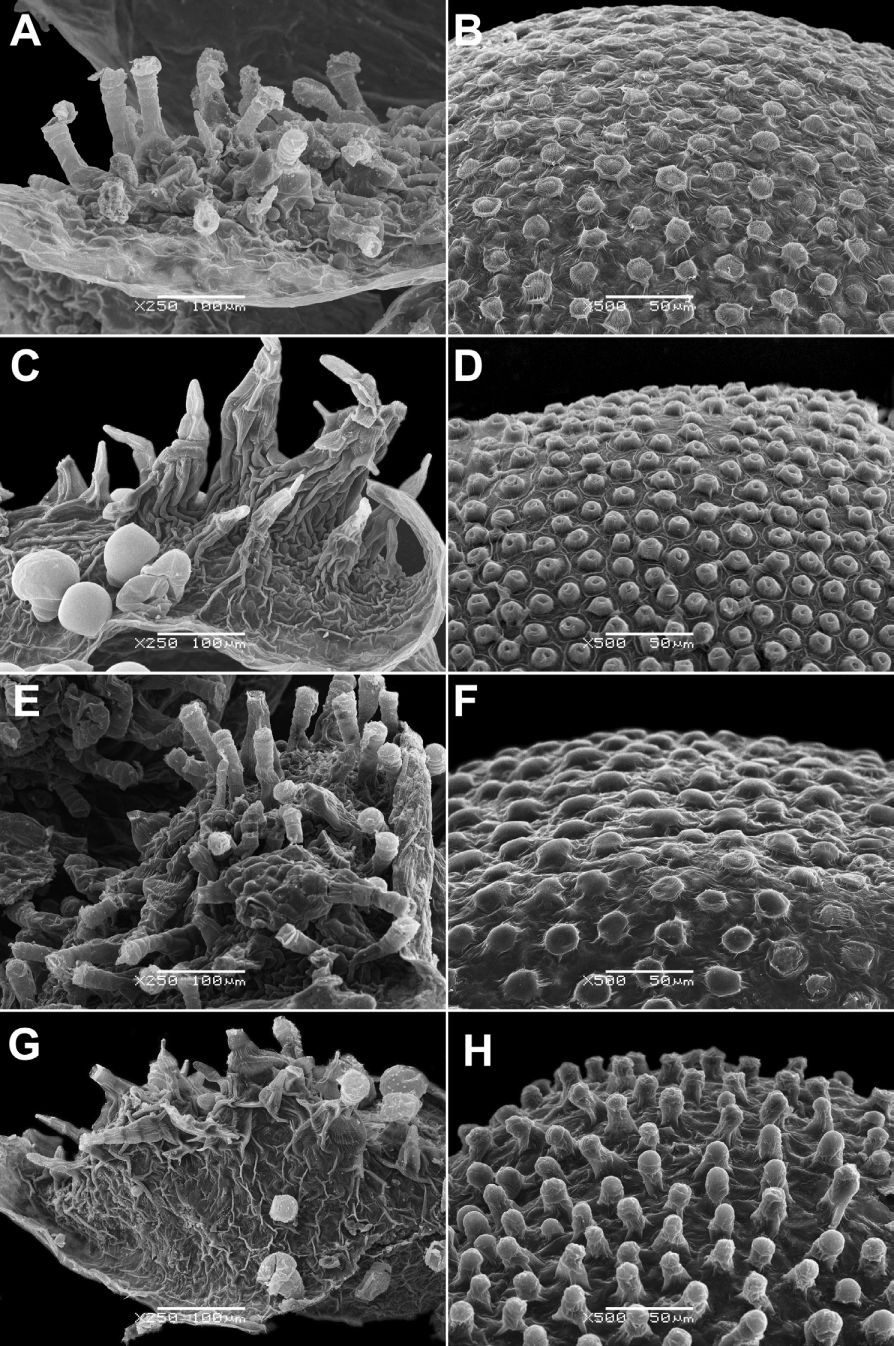
SEM micrographs: **A** perianth of *D.botrys***B** pericarp of *D.botrys***C** perianth of *D.geoffreyi***D** pericarp of *D.geoffreyi***E** perianth of *D.neglecta***F** pericarp of *D.neglecta***G** perianth of *D.nepalensis***H** pericarp of *D.nepalensis*. Magnification: 250× (**A, C, E, G**), 500× (**B, D, F, H**).

Annual up to 50 cm, mostly branched from the base, green or yellowish-green; stem and leaves densely covered with simple (up to 0.3 mm long) and glandular hairs. Lower leaves long-petiolate, up to 6.0(7.0–10.0) cm, pinnatifid; middle and upper leaves shorter, up to 5.0 cm, sometimes almost entire and crisp, very aromatic. Inflorescence up to 20 cm long with ± dense indumentum consisting of curved simple (up to 0.3 mm) hairs partially intermixed with glandular hairs. Perianth segments 5, free, 0.7–0.8 × 0.5 mm; their dorsal part with glandular hairs (up to 0.125 mm long) having scaphoid terminal cell and with scattered stout simple hairs up to 0.075 mm (Fig. 23A). Fruit 0.6–0.8 mm, subspherical; pericarp with minute, wart-like papillae (Fig. 23B); seed blackish, slightly keeled; embryo horizontal.

####### Habitat.

Mountain steppes with *Artemisia* sp. div., grasslands, sands, screes or as a weed; 2000–4000 m a.s.l. Common in many parts of Jammu & Kashmir and Himachal Pradesh.

####### Phenology.

Flowering: July-September; fruiting: August-October.

####### Distribution.

See Fig. 24.

**Figure 24.**
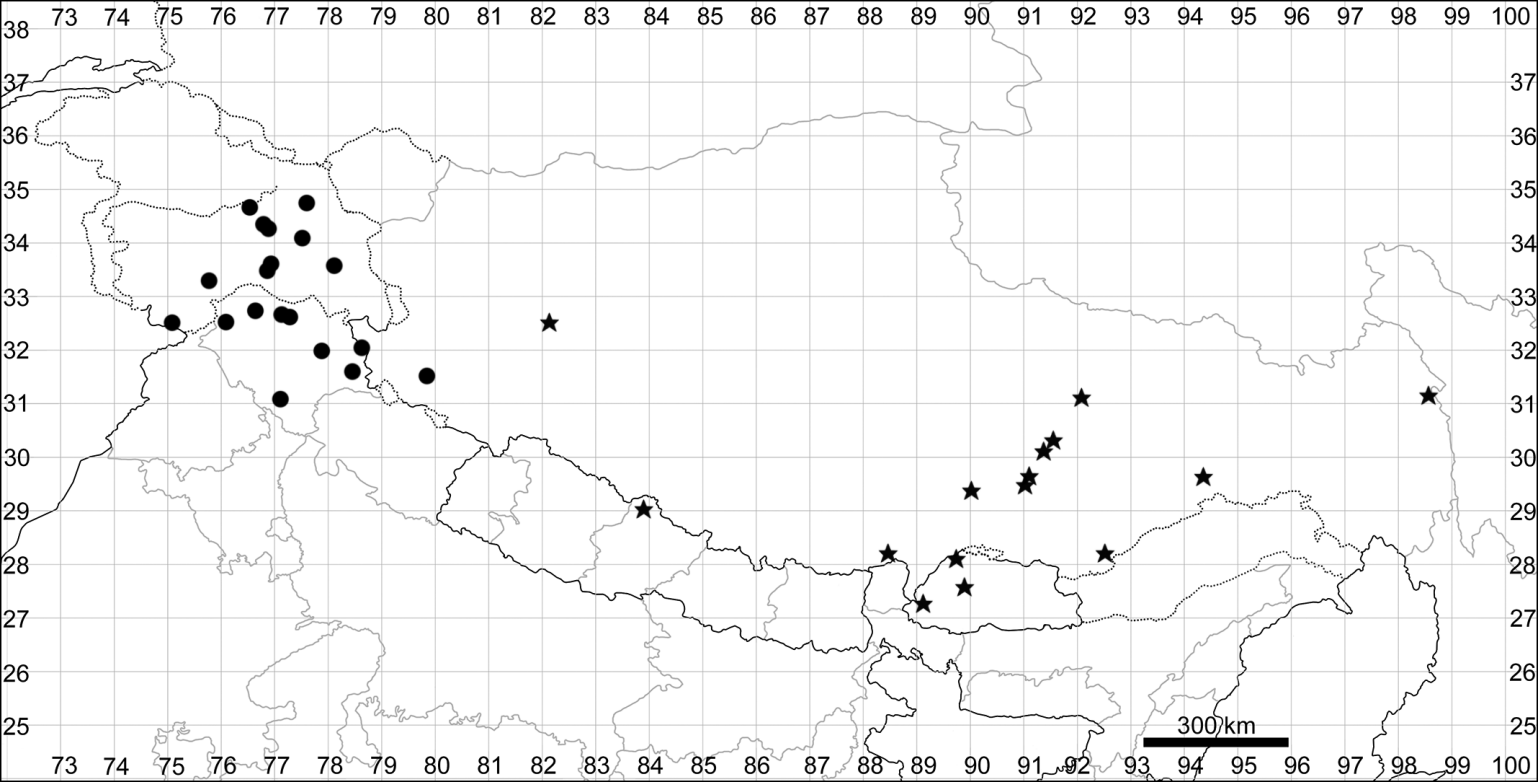
Distribution map of *Dysphaniabotrys* (circles) and *D.geoffreyi* (stars).

####### Specimens examined.

**CHINA**: **Xizang**: **Ngari Prefecture**: Zanda (Zhada) County, Diyag (Diya), 2900 m a.s.l., 2 Jul 1976, *Qinghai-Tibet Team 76-8155* (PE00510699, PE00510700);

**INDIA**: **Jammu & Kashmir**: Ladakh, Kaltse [Khaltse] to Damkar [Damkhar], Jul 1856, *Schlagintweit 1119* (G); Dah, Indus valley, Jul 1856, *Schlagintweit 1203* (LE); NE of Samba, Jul 1856, *Schlagintweit 3262* (G, LE); Ladakh, Nubra valley, Aug 1856, *Schlagintweit 2130* (BM); Chutium, 6000 ft a.s.l., 12 May 1928, *F. Ludlow 277* (G); Zanskar, Burdur, 12000 ft a.s.l., 18 Sep 1931, *W. Koelz 2995* (E, L, LE); Ladakh, Pituk [Pitok], 34°8'N, 77°31'E, 10500 ft a.s.l., 3 Sep 1956, *Vogt 1056* (G); [Kishtwar distr.] Kishtwar, 19 Sep 1958, *Rao 7775* (B, BSD); Ladakh, 3380 m a.s.l., 24 Aug 1976, *H. Hartmann 76* (MSB137916); Ladakh, Leh, Sumdo, 3500–3830 m a.s.l., 9 Sep 2001, *L. Klimeš 1696, 1699 & 1705* (PRA); Ladakh, Indus valley, Domkhar [Damkhar] Dha, 27 Aug 2002, *L. Klimeš 2360* (PRA); Ladakh, Zanskar Region, S of Padum, 3530 m a.s.l., 2 Sep 2003, *L. Klimeš 3442* (PRA); Ladakh, Indus valley, Stot (E) [Stod River valley], Likche, 3630 m a.s.l., 28 Sep 2003, *L. Klimeš 3652* (PRA); **Himachal Pradesh**: [Upper Lahaul] Jangi, 15 Aug 1890, *J.H*. *Lace 514* (E); [Chamba Distr.] Pangi, 8000 ft a.s.l., 2 Aug 1899, *J.F. Duthie* s.n. (K); Lahaul, Jispa, 11000 ft a.s.l., 11 Aug 1930, *W. Koelz 1008* & *1016* (P05158989, P05158992); Simla, 28 Jul 1934, *N. Parmanand 706* (E); Lahaul, Gondla, 10300 ft a.s.l., 2 Jul 1938, *N.L*. *Bor 12439* (K); Lahaul, Udaipur, 2850 m a.s.l., 2 Aug 1990, *R.J.D. McBeath 2270* (E); Spiti distr., Pin Valley National Park, 26 Aug 2002, *K.C. Sekar 100711* (BSD).

####### General distribution.

Temperate Eurasia, North Africa, alien in North America, Australia and New Zealand.

###### 6. Dysphania geoffreyi

Taxon classificationPlantaeCaryophyllalesChenopodiacea

Sukhor., Phytotaxa 203(2): 139 (2015).

####### Holotype.

CHINA, Tibet [Xizang], Lhasa, Daxika, grassy slope, 4150 m a.s.l., herb with green flowers, 5 Sep 1965, *Yong-tian Zhang & Kai-yong Lang 2663* (PE00510980!). Original text in Chinese.

####### Description.

Annual, 5–50 cm tall, stem erect, often scarcely branched and then only in the upper part, branches directed upwards. Leaves pinnatifid or lobate, rarely toothed, appressed to the stem, up to 2.5 cm long, shortly petiolate (petioles 0.2–1.0 cm); lower leaves caducous at the flowering and fruiting stages. Inflorescence leafy, lateral branches appressed to the stem or obliquely directed, narrowly cylindrical but not spreading, axis covered with short simple hairs up to 0.3 mm long mixed with glandular trichomes. Perianth segments 5, 0.6–0.7 × 0.3–0.4 mm, cristate on dorsal surface (especially on midrib) with numerous large simple stout hairs up to 0.3 mm, the bases of which can sometimes be concrescent, resulting in 2−3-furcate tips, intermixed with yellow subsessile glands and scattered glandular hairs (Fig. 23C). Fruit subspherical, 0.5–0.6 mm in diameter; pericarp with tiny volcano-like papillae (Fig. 23D). Seed blackish, keeled, embryo horizontal.

####### Habitat.

Grassy slopes, river banks and sands at 2500–4200(4300–4800) m a.s.l.

####### Phenology.

Flowering: July-September; fruiting: September-October.

####### Distribution.

See Fig. 24.

####### Specimens examined.

**BHUTAN**. Ohra, 11000 ft a.s.l., 28 Aug 1915, *R.E*. Cooper *4733* (BM, E00151614); [Haa Distr.] Ha, 2797 m a.s.l., 18 Jun 1971, *R. Bedi 162* (K); Upper Mo Chu [Punakha] Distr., Gangyvel Chu below Gangyvel, 3820 m a.s.l., 27 Sep 1984, *I. Sinclair & D. Long 5364* (E00151629); Gasa Distr., Umtsako/Makasang Chu, 28°05'N, 89°44'E, 4000 m a.s.l., 14 Aug 2000, *G. & S. Miehe 00-283-35* (herb. Miehe);

**CHINA**. **Xizang**: Kham (Tibet), basin of Yangtse river, Nru-chu, 25 Jul 1900, *V.F*. *Ladygin 371* (LE, MW); Nagong, Shinden Gompa, 13000–14000 ft a.s.l., 17 Aug 1933, *F. Kingdon-Ward 10760* (BM); Dochen, 14000 ft a.s.l., 7 Aug 1936, *Chapman 890* (K); **Ngari Prefecture**: Gê’gyai (Geji) County, Yanhu Distr., 4350–4500 m a.s.l., 24 Aug 1976, *Qinghai-Tibet Team Vegetation Group 13531* (PE00235088); **Nagqu Prefecture**: S of Nagqu, Kema, 31°16'N, 92°07'E, 4480 m a.s.l., 10 Sep 2007, *G. & S. Miehe 07-019-06* (herb. Miehe); **Xigazê Prefecture**: [Gamba County] Kamba Jong [Gamba Zong], 11 Aug 1903, *F.E. Younghusband 243* (B100448362, BM); **Lhasa City**: nr Lhasa, Jul 1939, *Richardson 308* (BM); Lhasa, 12000 ft a.s.l., 5 Sep 1943, *F. Ludlow & G. Sherriff 9919* (BM); Lhasa, [years] 1946–1950, *P. Aufschnaiter* s.n. (BM); Lhünzhub (Linzhou) County, Poindo (Pangduo), 4000–4100 m a.s.l., 7 Oct 1972, *Tibet Chinese Herbal Medicine Survey Team 1891* (PE00510966, PE00511070); [Lhünzhub (Linzhou) County] Upper Kyi Chu, Reting Monastery, 30°18'N, 91°31'E, 4300 m a.s.l., 22 Aug 1997, *G. & S*. *Miehe & al*. *97-027-06* (herb. Miehe); Nyemo County, Yarlung Tsangpo gorge, 29°20'N, 90°03'E, 5 Sep 1998, *G. & S. Miehe 98-07509* (herb. Miehe); [Doilungdêgên County] Kui Chu, Nyimathang, 29°30'N, 91°02'E, 1 Oct 2001, *G. & S. Miehe 01-141-10* (herb. Miehe); **Nyingchi Prefecture**: Nyingchi (Linzhi) County, 2600 m a.s.l., 19 Jun 1972, *Tibet Chinese Herbal Medicine Survey Team 3498* (PE00510994);

**NEPAL**: **Western**: **Dhaulagiri Zone**: Tegar (N of Mustang), 13500 ft a.s.l., 6 Aug 1954, *J. Stainton, W.R. Sykes & L.H.J. Williams 2248* (BM).

####### General distribution.

South Tibet, Eastern and Central Himalaya. The species can also be found in India (especially in Arunachal Pradesh) and North Myanmar.

###### 7. Dysphania tibetica

Taxon classificationPlantaeCaryophyllalesChenopodiacea

(A.J.Li) Uotila, Willdenowia 43(1): 67 (2013)

≡Chenopodiumtibeticum A.J.Li, Fl. Xizang 1: 638 (1983). **Lectotype** (Sukhorukov, designated here): CHINA, Xizang, Gyangze, prope vicum [near a village], 3960 m a.s.l., [5 Sep 1974], *Qinghai-Xizang Complex Expedition 74-2077* (PE00024039! isolectotype PE00934045!). In the protologue, Li (in Li and Ma 1983) cited all specimens with the same label as “type” kept at H[erbarium] P[ekinense], now represented by the abbreviation PE. The labels of “holotype” and “isotype” were later assigned by an unknown botanist.
≡Neobotrydiumtibeticum (A.J.Li) M.L.Zhang & G.L.Chu, Pl. Diversity 38(6): 326 (2016).

####### Description.

**Figure 25.**
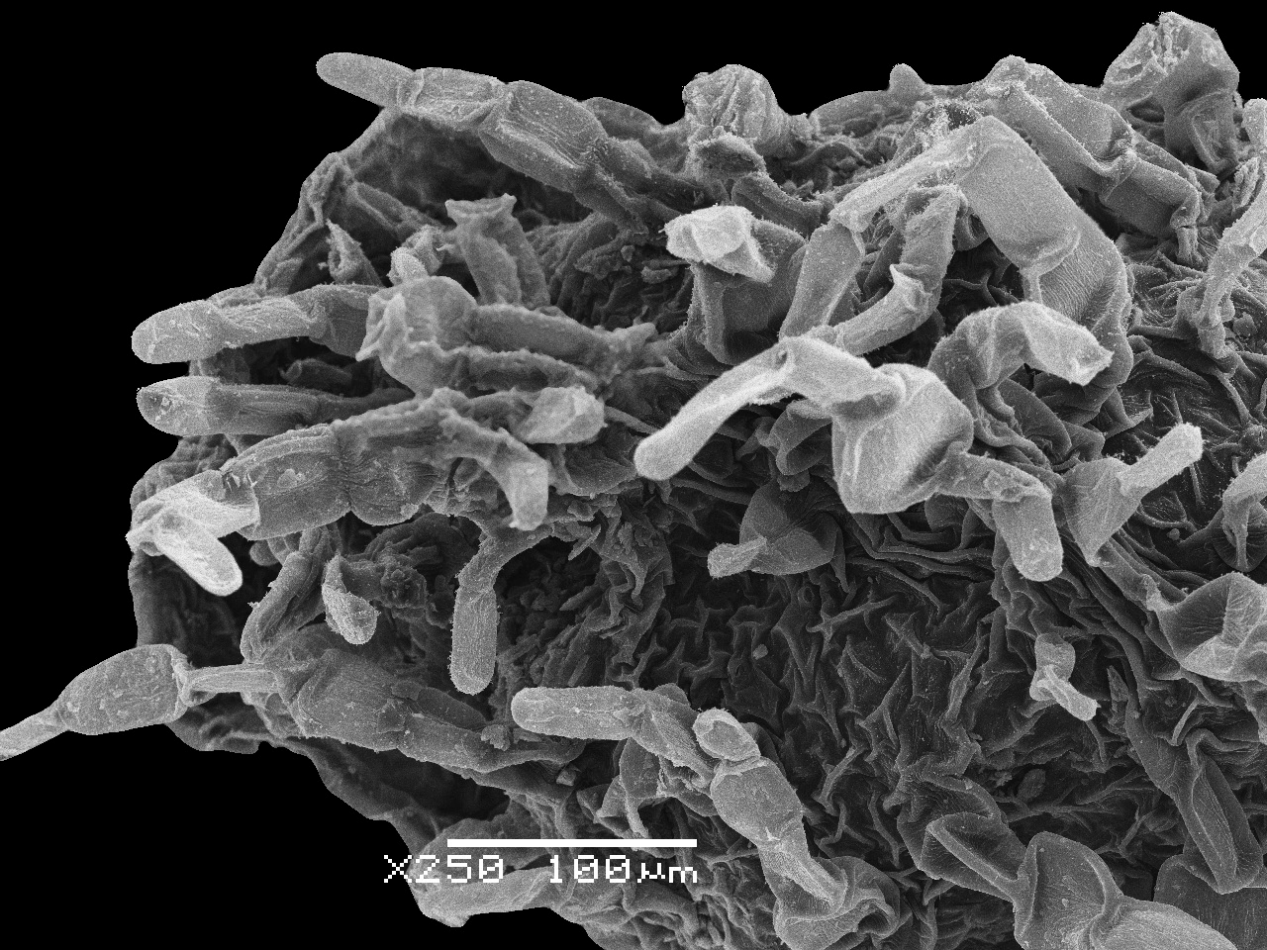
SEM micrograph of *Dysphaniatibetica* perianth segment, magnification 250×.

**Figure 26.**
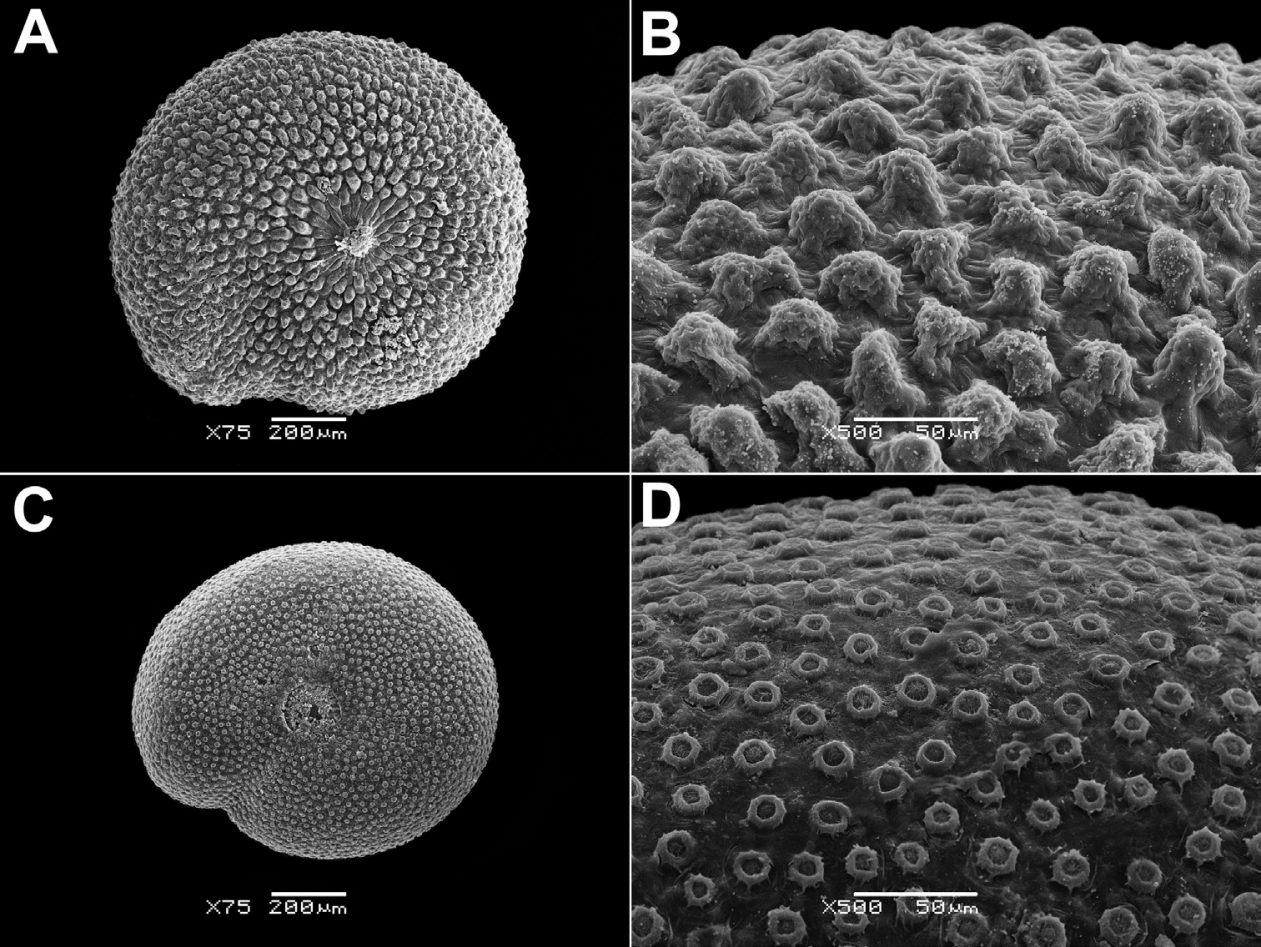
SEM micrographs of *Dysphaniatibetica*: **A, B** fruit with red seed **C, D** fruit with brown seed. Magnification: 75× (**A, C**), 500× (**B, D**).

Annual up to 25 cm, branched from the base with prostrate or ascending stems. Leaves petiolate, up to 4.0 cm, entire to slightly lobate, scarcely pubescent with curved simple hairs. Inflorescence axillary, leafy and short; its branches flattened and slightly winged, usually shortly bifurcate at the top, relatively densely pubescent with semi-appressed or curved simple hairs up to 0.6 mm long, sometimes with scattered glands. Perianth segments 0.7–0.8 × 0.6 mm; their dorsal part with dense indumentum of soft simple hairs (up to 0.3 mm long) and scattered glandular hairs (Fig. 25). Fruits dimorphic: 1.0–1.1 × 0.9 mm, subspherical, pericarp with conical papillae (Fig. 26A, B) and slightly flattened, 0.8–1.0 × 0.7 mm, broadly ovoid, pericarp with crater-like papillae (Fig. 26C, D). Seeds dimorphic: red, slightly keeled, and brownish, not keeled; seed embryo horizontal.

####### Habitat.

Gravelly slopes and ruderal sites at elevations of 3800–5000 m a.s.l. Some specimens from Ladakh contain both *D.tibetica* and *D.himalaica*, and we assume they can often grow together.

####### Phenology.

Flowering: July-September; fruiting: August-October.

####### Distribution.

See Fig. 27.

**Figure 27.**
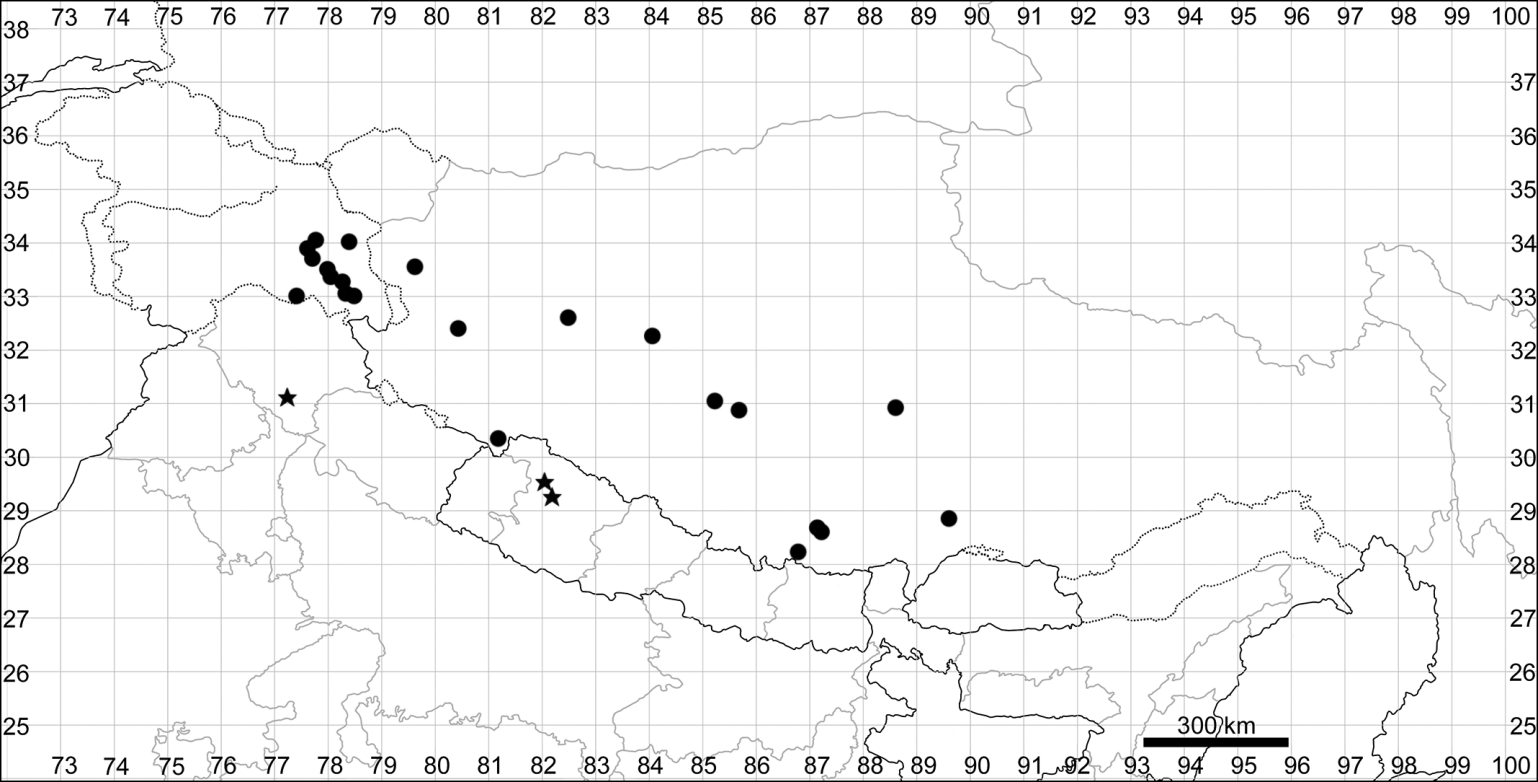
Distribution map of *Dysphaniatibetica* (circles) and *D.neglecta* (stars).

####### Specimens examined.

**CHINA**: **Xizang**: **Ngari Prefecture**: Gêrzê (Gaize) County, Datan, 4450 m a.s.l., 6 Sep 1974, *Biological Institute Tibet Expedition Team 4295* (HNWP40801); Gar (Gaer) County, Sogdoi (Suoduo), 4300 m a.s.l., 27 Jul 1976, *Qinghai-Tibet Team 76-8588* (PE00540036); nr Yanhuqu, 4400 m a.s.l., 24 Aug 1976, *Tibetan Collection Team 8723* (KUN0265022, PE00540041); Gê’gyai (Geji) County, Yanhu Distr., 4370 m a.s.l., 24 Aug 1976, *Qinghai-Tibet Team Vegetation Group 13529* (PE00235089, PE00235093); Coqên (Cuoqin) County, 4600 m a.s.l., 15 Sep 1976, *Qinghai-Tibet Team Vegetation Group 12580* (PE00235091, PE00540038); Burang (Pulan) County, Kejia, 3700 m a.s.l., 29 Aug 1990, *Y. Fei* et al. *659* (KUN0265544); Rutog Xian, Banggong [Pangong] Lake, 4300 m a.s.l., 3 Sep 1990, *Y. Fei* et al. *713* (KUN0265542); **Nagqu Prefecture**: Xainza (Shenzha) County, NW Lake Qilin, 1 Sep 1976, *Qinghai-Tibet Team Vegetation Group 12425* (PE00235090); **Xigazê Prefecture**: “Expedition to Everest”, 16000 ft a.s.l., 1921, *A. Wollaston 47* (K); Tingri County, Jul 1921, *A. Wollaston 265 & 266* (K); Gyangzê (Jiangzi) County, 3960 m a.s.l., 5 Sep 1974, *Qinghai-Tibet Team 74-2073* (KUN0587076, PE00540039), *Qinghai-Tibet Team 74-2077* (KUN0587080); Upper Arun Valley, N of Xegar (Tingri), 28°37'N, 87°10'E, 4400 m a.s.l., 5 Oct 1989, *B. Dickoré 5919* (KAS, n.v., after Uotila 2013);

**INDIA**: **Jammu & Kashmir**: Ladakh, Angkung to Puga, 33°14'N, 78°16'E, 4550 m a.s.l., 8 Sep 1999, code 99-27-9, *L. Klimeš 778* (PRA, together with *D.himalaica*); Rupshu Region, Tso Moriri, 4810 m a.s.l., 11 Jul 2000, *L. Klimeš 893* (PRA); Leh, Lato, 33°40'42"N, 77°43'48"E, 4020 m a.s.l., 5 Sep 2001, *L. Klimeš 1562* (PRA); Zhung (Leh), Gya to Lato, 33°40'2"N, 77°43'9"E, 4060–4070 m a.s.l., 5 Sep 2001, code 01-38-12, *L. Klimeš 1539* (PRA); Ladakh, Leh, Chukirmo, 33°49'30"N, 77°39'6"E, 4150 m a.s.l., 8 Sep 2001, *L. Klimeš 1657* (PRA); Leh, Stagar (Sakti) to Wari La, 34°2'48"N, 77°49'18"E, 4240–4270 m a.s.l., 12 Sep 2001, *L. Klimeš 1741* (PRA); Stot (E) [Stod river valley] Tiri vill., 33°31'30"N, 77°58'36"E, 4330–4460 m a.s.l., 1 Aug 2001, *L. Klimeš 1190* (PRA); Rupshu, 32°58'30"N, 77°24'E, 4600 m a.s.l., 11 Jul 2000, code 00-10-4, *L. Klimeš* s.n. (H1757589); Samad Rakchan, Thukje to Nyamur, 33°20'8"N, 78°1'40"E, 4560 m a.s.l., 9 Sep 2005, *L. Klimeš 6268* (PRA); Tso Moriri, Karzok to Peldo, 32°59'30"N, 78°15'E, 4550 m a.s.l., 13 Sep 2005, *L. Klimeš 6309* (PRA); Tso Moriri, Lapgo river valley, 32°58'42"N, 78°21'18"E, 4810 m a.s.l., 11 Jul 2000, *L. Klimeš 6268* (PRA); Ladakh, Leh, Lato, 4020 m a.s.l., 5 Sep 2001, *L. Klimeš 1545* (PRA); Ladakh, Pangong Region, Lukung, 4300 m a.s.l., 9 Sep 2002, *L. Klimeš 2690* (PRA).

####### General distribution.

Himalaya and Tibet.

###### 8. Dysphania neglecta

Taxon classificationPlantaeCaryophyllalesChenopodiacea

Sukhor., Carpologiya Chenopodiaceae: 347 (2014)

Fig. 3

####### Holotype.

NEPAL, Far West Nepal [Midwestern, Karnali Zone], Jumla Prov. [Distr.], Jumla town, 29°16'28"N, 82°11'01"E, weed in the city, 2400 m a.s.l., 23 Sep 2013, *A. Sukhorukov 241* (BM000832632! isotype MW0591987!).

####### Description.

Annual up to 100(120) cm tall, green or yellowish-green, very aromatic; stem and leaves densely covered with simple (up to 1.0 mm long) and glandular hairs. Lower leaves long-petiolate, up to 10.0 cm long, pinnatifid, middle and upper leaves shorter, up to 5.0 cm long. Inflorescence up to 45 cm long with villose indumentum consisting of simple (up to 1.0 mm long) hairs partially intermixed with glandular hairs. Perianth segments 5, free, 0.8–1.1 × 0.5 mm long, their dorsal part with glandular hairs (up to 0.125 mm) having scaphoid terminal cell, sometimes also with scattered simple hairs (Fig. 23E). Fruit 0.7–0.8 mm, subspherical; pericarp with minute wart-like papillae (Fig. 23F). Seed blackish, keeled; embryo horizontal.

####### Habitat.

Limestones, roadsides, along streams at elevations of (1800–1900)2000–3200 m a.s.l.

####### Phenology.

As in *D.nepalensis* but fruiting 1–2 weeks later, in October (according to the observations in West Nepal).

####### Distribution.

See Fig. 27.

####### Specimens examined.

**INDIA**: **Himachal Pradesh**: Simla Distr., Urni-Chim, 9 Jul 1940, *M.R. Abbi 2849* (G);

**NEPAL**: **Midwestern**: **Karnali Zone**: [Jumla Distr.] Uthu, E of Jumla, 8000 ft a.s.l., 31 Jul 1952, *O. Polunin, W.R. Sykes & L.H.J. Williams 4970* (BM); [Jumla Distr.] 15 km NE from Jumla vill., 1800 m a.s.l., 28 Sep 2010, *A. Sukhorukov* s.n. (MW); Border of Jumla & Mugu Distr., 1 km from Naurigar vill., 2300 m a.s.l., 26 Sep 2013, *A. Sukhorukov* s.n. (G); Jumla Distr., Jumla vill., 6 Oct 2014, *A. Sukhorukov 585* (BR); Jumla Distr., Sinja, Oct 2014, *A. Sukhorukov 57* (M); Mugu Distr., Chauta vill., 2500 m a.s.l., 7 Oct 2014, *A. Sukhorukov 599* (MW).

####### General distribution.

West and Central Himalaya (NE India, Nepal). Common in West Nepal.

###### 9. Dysphania nepalensis

Taxon classificationPlantaeCaryophyllalesChenopodiacea

(Colla) Mosyakin & Clemants, J. Bot. Res. Inst. Texas 2(1): 428 (2008)

Fig. 3

≡Chenopodiumnepalense Colla, Herb. Pedem.: 25 (1836). **Holotype**: [NEPAL] ex herb. Biroli (TO5972!).
=Chenopodiummultiflorum Moq. in DC., Prodr. 13(2): 75 (1849). **Lectotype** (designated by Sukhorukov 2012): [INDIA] Ind. Orient. [Uttarakhand], Garhwal, Jun 1845, *T.T*. *Thomson 1324* (K! image available at http://apps.kew.org/herbcat/getImage.do?imageBarcode=K000898432; isolectotype BM000629118! image available at http://www.nhm.ac.uk/emu-classes/class.EMuMedia.php?irn=181039&image=yes&width=705).

####### Taxonomic notes.

According to the protologue and drawing of the newly described *Neobotrydiumornithopodum* G.L. Chu & M.L. Zhang (Zhang and Zhu 2016), this taxon corresponds to *Dysphanianepalensis* in many details, but we suppose that *D.nepalensis* is not uniform in China. For example, the plants from N, E and NE China as well as those from Mongolia are similar to *D.nepalensis* but have leaves with acute lobes (*D.nepalensis* has obtuse leaf lobes). No other morphological differences between Himalayan/Tibetan and Central Asian populations were found.

####### Description.

Annual up to 50(70) cm, strongly aromatic. Leaves 4.0–10.0 × 2.0–4.0 cm, long-petiolate, pinnatifid or lobate, elliptic-oblong in outline, apex rounded, pubescent mostly abaxially. Inflorescence lax with ± dense indumentum consisting of simple (up to 1.0 mm long) hairs partially intermixed with glandular hairs. Perianth segments 5, 0.8–1.0 × 0.4–0.5 mm, basally concrescent with stout simple hairs (up to 0.1 mm) forming a keel and yellow subsessile glands (Fig. 23G). Fruit 0.7–0.8 × 0.5 mm, subspherical. Pericarp with minute conical papillae up to 25 μm (Fig. 23H). Seed dark red or almost black, keeled. Embryo horizontal.

####### Habitat.

Grassy hill slopes, disturbed places; 2400–4600 m a.s.l.

####### Phenology.

Flowering: June-September; fruiting: August-November.

####### Distribution.

See Fig. 28.

**Figure 28.**
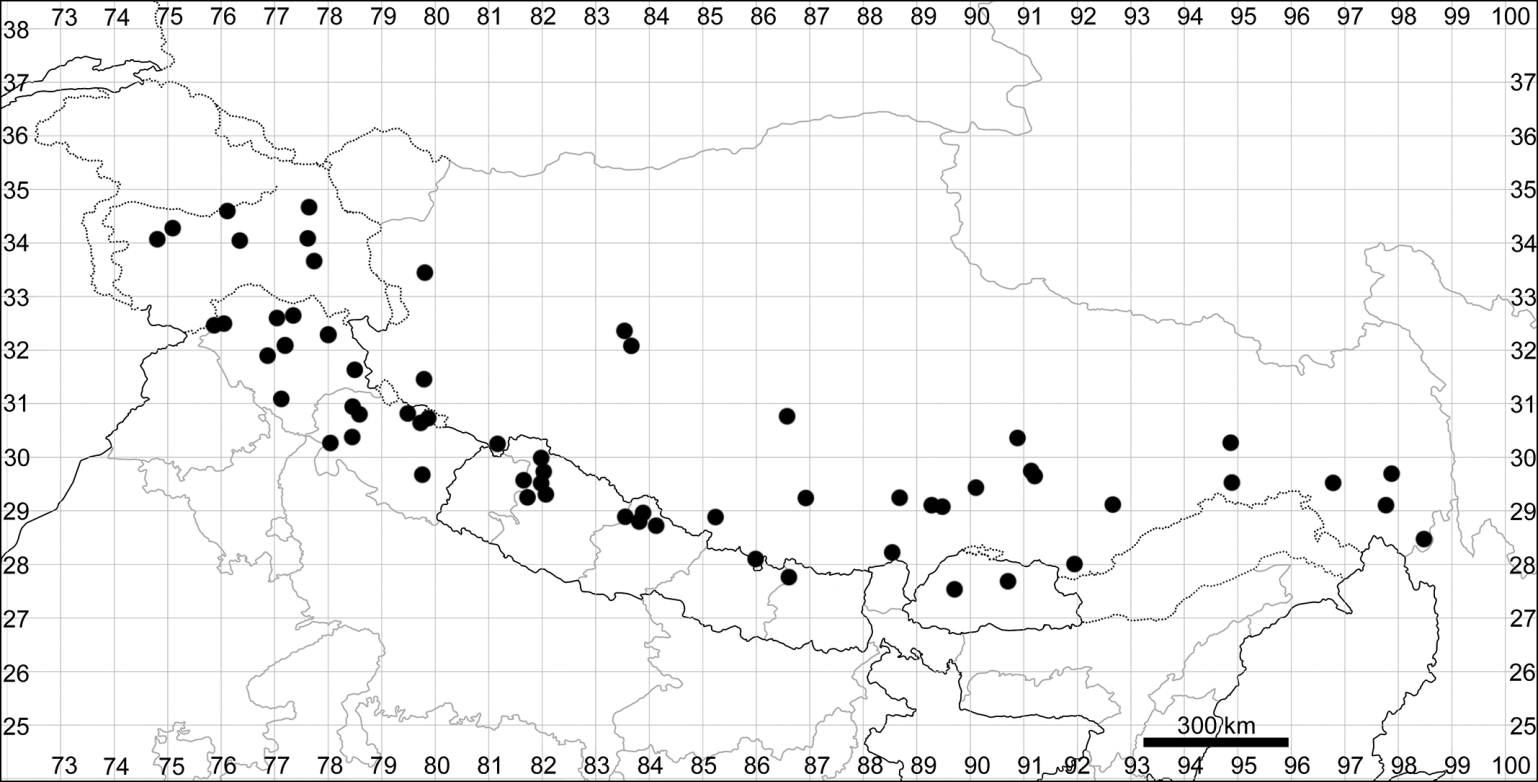
Distribution map of *Dysphanianepalensis*.

####### Specimens examined.

**BHUTAN**: [Thimphu & Paro Districts] Thimphu & Paro rivers, 2400 m a.s.l., 14 Aug 1963, *N.P*. *Balakrishnan 1195* (CAL); [Bumthang Distr.] Bumthang, 2600 m a.s.l., 6 Jun 1992, *C. Parker 7176* (E00051985);

**CHINA**: **Xizang**: **Ngari Prefecture**: Zanda (Zhada) County, Toling (Tuolin), 3700 m a.s.l., 6 Aug 1974, *Biological Institute Tibet Expedition Team 3830* (XJBI00005673); Burang (Pulan) County, 3900 m a.s.l., 18 Aug 1974, *Biological Institute Tibet Expedition Team 3990* (XJBI00005675); Rutog (Ritu) County, Lake Bangong (Pangong Tso), 4220 m a.s.l., 3 Sep 1976, *Qinghai-Tibet Team Meadow Group 76-238* (PE00541034, PE00541035); Gêrzê (Gaize) County, Marmê (Mami) Distr., 4500 m a.s.l., 8 Sep 1976, *Gansu Agriculture University Team 161* (PE00541033); Gêrzê (Gaize) County, Oma (Wuma) Distr., 4550 m a.s.l., 9 Sep 1976, *Qinghai-Tibet Team Vegetation Group 12521* (PE00511042); **Nagqu Prefecture**: [Nyima (Nima) County] Changthang, Dangra Yum Tso, 30°43'N, 86°35'E, 4590 m a.s.l., 9 Sep 2003, *G. & S. Miehe 03-081-05* (herb. Miehe); **Xigazê Prefecture**: Bainang (Bailang) County, 3830 m a.s.l., 7 Jul 1960, *Fu 354* (PE00511044); Gyirong (Jilong) County, Ruga, 3200 m a.s.l., 24 Jun 1972, *Tibet Chinese Herbal Medicine Survey Team 514* (PE00510968); Namling (Nanmulin) County, Gangba, 4000 m a.s.l., 3 Aug 1972, *Tibet Chinese Herbal Medicine Survey Team 972* (PE00510971); Nyalam (Nielamu) County, 3800 m a.s.l., 5 Sep 1972, *Tibet Chinese Herbal Medicine Survey Team 1783* (PE00510969); Yadong (Chomo) County, Renqinggang Distr., 2800 m a.s.l., 9 Sep 1974, *Qinghai-Tibet Team 74-2145* (PE00510975); [Ngamring (Angren) County] Lheding Gompa, 29°14'N, 86°53'E, 4540 m a.s.l., 20 Aug 2003, *G. & S. Miehe 03-015-35* (herb. Miehe); 20–30 km W of Xigazê city, 16 Jul 1994, *M.L. Zhang & S.Z. Zhang 94178* (WUK0780064); **Lhasa City**: Lhasa, 3800 m a.s.l., 16 Aug 1965, *Zhang & Lang 1532* (PE00510990); Lhasa, Daxika to Longnanchaika, 4500 m a.s.l., 31 Aug 1965, *Zhang & Lang 2249* (PE00510989); Nyêmo (Nimu) County, 3900 m a.s.l., 7 Oct 1972, *Tibet Chinese Herbal Medicine Survey Team 4578* (PE00510970); Lhasa, 3650 m a.s.l., 2 Oct 1976, *Qinghai-Tibet Team Vegetation Group 13813* (PE00511041); Lhasa, NW of Sera, 29°43'N, 91°08'E, 16 Sep 1999, *G. & S. Miehe & Koch 99-201-01* (herb. Miehe); [Damxung (Dangxiong) County] Nindung Xian, 30°23'N, 90°54'E, 4285 m a.s.l., 13 Sep 2009, *G. Miehe & Seeber 09-125-16* (herb. Miehe); **Shannan Prefecture**: Gyaca (Jiacha) County, 3340 m a.s.l., 22 Sep 1974, *Qinghai-Tibet Team Vegetation Group 3358* (PE00234845); Cona (Cuona) County, 3200 m a.s.l., 10 Sep 1975, *Qinghai-Tibet Team Additional Group 751960* (PE00510973); Yarlung Tsangpo, Gyaca, 3530 m a.s.l., 29°09'N, 92°35'E, 19 Sep 1998, *G. & S. Miehe 98-14314* (herb. Miehe); **Nyingchi Prefecture**: [Zayü (Chayu) County] Cawarong (Chawalong), 3000 m a.s.l., Sep 1935, *C. W. Wang 66345* (PE00511036); [Bomi County] Yi’ong (Yigong), 2200 m a.s.l., 19 Jul 1965, *Ying & Hong 650676* (PE00510985); Nyingchi (Linzhi) County, Naixi (Nixi), 3100 m a.s.l., 3 Aug 1965, *Zhang & Lang 1292* (PE00510995); [Mainling (Milin) County] Tsangpo gorge, 29°35'N, 94°55'E, 18 Sep 1989, *B. Dickoré 5506* (MSB144247); **Qamdo Prefecture**: Baxoi (Basu) County, near Rawu (Ranwu), 3900 m a.s.l., 29 Aug 1973, *Qinghai-Tibet Team 73-1317* (PE00510991); Zogang (Zuogong) County, 3800 m a.s.l., 1 Jul 1976, *Qinghai-Tibet Team 12080* (PE00510978);

**INDIA**: **Jammu & Kashmir** (selected specimens): Ladakh, Leh, Sep 1856, *Schlagintweit 4* (P04993262); Srinagar, Sep 1856, *Schlagintweit 4413* & *4631* (BM, E00151682, L1671277); Srinagar, Aug 1875, *Aitchinson 4* (K); [Anantnag Distr.] Pahlgam, 14 Aug 1920, *R.R*. *Stewart 5683* (K); Ladakh, Gya, Aug 1933, *W. Koelz 6448* (G); Ladakh, Kargil, 9500 ft a.s.l., Aug 1934, *Kohli 33* (K); Sind valley, Kulan, 7500 ft a.s.l., 31 Aug 1956, *O. Polunin 56-611* (BM); Ladakh, Leh, 15 Aug 1975, *M.V. Viswanathan 54685* (BSD); Ladakh, Rungdum [Rangdum], 4000 m a.s.l., 1980, *Southampton Univ. Team 129* (K); Leh, 3620 m a.s.l., 1 Aug 1992, *H. Hartmann 4015* (G455288); Ladakh, Leh/Sankar, 3500 m a.s.l., 4 Aug 1999, *Beck 9* (MSB144242); Ladakh, Nubra Region, Sumur to Tiggur, 3150 m a.s.l., 18 Sep 2001, *L. Klimeš 1918* (PRA); **Himachal Pradesh** (selected specimens): Kinnaur, 5 Sep 1847, *T.T. Thomson* s.n. (K); Dalhousie, 20 Sep 1874, *C.B*. *Clarke 22781* (BM); Dalhousie, 1880, *J. Drummond 1422* (K); Simla, [without date] *Huegel* s.n. (G); Simla, [without date] *E. Madden 592* (E00151698); Simla, 7000 ft a.s.l., 1 Sep 1886, *H. Collett 586* (K); Chamba, 28 Aug 1896, *G.A*. *Gammie 18299* (LE); Chamba Distr., Kulel, 2 Sep 1896, *G.A*. *Gammie 18455* (K); [Chamba Distr.] Pangi, 8000 ft a.s.l., 2 Aug 1899, *J.F*. *Duthie* s.n. (K); Lahaul, Kolagmes, Jul 1930, *W. Koelz 453* (P05158987); Naggar, 2 Oct 1930, *W. Koelz 1406* (P05159007); Lahaul, Kyelang, 10200–12000 ft a.s.l., 12 Jul 1941, *N.L*. *Bor 15206* (K); Chatri, 2100 m a.s.l., 15 Oct 1987, *R.J.D*. *McBeath 01967* (E); Spiti Distr., Kibber, 13 Aug 1994, *S. Singh 88299* (BSD); **Uttarakhand** (selected specimens): Badhrinath, 10000 ft a.s.l., 11 Aug 1847, *R. Strachey & J.E. Winterbottom 1* (BR, K); Tehri Garhwal [Distr.], Gorges valley, Aug 1889, *J.F. Duthie 352* (BM, G, K); Almora Distr., 9 Oct 1950, *Avasthi 1902* (B); nr Maneri, 26 Aug 1952, *P. Huggins A6* (BM); Garhwal, Jumma, 2800 m a.s.l., 15 Aug 1974, *B.D. Naithani 53894* (BSD); Chamoli Distr., Malari, Aug 1988, *P.K. Hajra 87195* (BSD); Uttarkashi Distr., Kedarganga valley, 28 Jul 2002, *P.K. Pusalkar 102005* (BSD);

**NEPAL**: **Far-Western**: **Seti Zone**: [Bajura Distr.] Kolti [vill.], 5000 ft a.s.l., Sep 1963, *R. Bhandary 1222* (CAL); **Midwestern**: **Karnali Zone**: [Mugu Distr., nr Rara Lake] Gum Garhi, 2400 m a.s.l., 5 Jul 1977, *T.B*. *Shrestha & N.P. Manandhar 241* (E00214393); Kalikot Distr., 7 Aug 1991, *H. Takayama & K. Terada 9160336* (BM); Humla Distr., Humla, *anonym* s.n. (KATH); Humla Distr., Thanke Khola, E of Pipilang, 29°44'N, 82°2'E, 1700 m a.s.l., 14 Jul 2008, *C. Pendry* et al. *A141* (E00392110); [Jumla Distr.] Jumla vill., 29°17'N, 82°05'E, 2400 m a.s.l., 3 Oct 2010, *A. Sukhorukov 464* (G, MW, W2011-0006543, E00607768); Mugu Distr., 5 km SW of Rara Lake, 2500 m a.s.l., 22 Sep 2013, *A. Sukhorukov 729* (G, MW); **Western**: **Dhaulagiri Zone**: Mustang [Distr.] 13000 ft a.s.l., 3 Aug 1954, *J. Stainton, W.R. Sykes & L.H.J. Williams 2165* (BM); [Manang Distr.] Marsyangdi valley, between Pisang & Ongre, 3200 m a.s.l., 25 Sep 1969, *Wraber 36508* (BM); Cha Lungpha, near Sangda, 13200 ft a.s.l., 6 Aug 1977, *G. & S. Miehe 434* (BM); Mustang Distr., Tangbe, 28°54'N, 83°49'E, 31 Aug 2001, *S. & G. Miehe & Koch 01-098-01* (herb. Miehe); Mustang Distr., Annapurna Conservation area, trekking route Jomosom-Nayapul, valley of Kali Gandaki river, 2500 m a.s.l., 25 Sep 2008, *A. Sukhorukov 219* (MW); **Eastern**: **Sagarmatha Zone**: [Solukhumbu Distr.] Khumbu, Paugroche, 3900–4000 m a.s.l., 9 Oct 1962, *J. Poelt* s.n. (M).

####### General distribution.

Himalaya (southwards to Yunnan), Tibetan Plateau and Pamir Mountains. Widespread in Nepal.

##### Excluded species

###### Dysphania schraderiana

Taxon classificationPlantaeCaryophyllalesChenopodiacea

(Schult.) Mosyakin & Clemants, Ukr. Bot. Zhurn. 59(4): 383 (2002)

≡Chenopodiumschraderianum Schult. in Roem. & Schult., Syst. Veg., ed. 15(6): 260 (1820).
=Chenopodiumfoetidum Schrad., Mag. Neuest. Entd. Berlin 2: 79 (1808) nom. illegit., non Lam. 1779.

####### Notes.

This species, which is distributed in East Africa and Arabia and found as alien in many (sub)tropical areas of the world, is absent in our area. All records (Zhu et al. 2003) belong to other (native) *Dysphania* species, mostly *D.nepalensis*.

###### 8. Teloxys

Taxon classificationPlantaeCaryophyllalesChenopodiacea

Moq., Ann. Sci. Nat., Bot. sér. 2, 1: 289 (1834)

Teloxys aristata (L.) Moq. (Type).

####### Description.

Annual up to 25 cm forming tumble-weed habit, non-aromatic, highly branched from the base, branches usually terminating with acicular apices, glabrous or with short papillae and white glandular hairs (mostly present in lower stem parts). Leaves up to 6.0 cm long, narrowly oblong or spatulate, sessile or with petiole-like base, entire, often folded on the ventral side. Inflorescences usually terminate with aristae, sometimes (in wet habitats) without acicular apices; flowers solitary in the axils of falsely dichotomous branches. Perianth segments 5, free at base, with slightly keeled midrib, hyaline or pinkish, glabrous. Styles 2(3). Fruit 0.7–0.8 mm, compressedly spherical. Pericarp tightly adjoining the seed coat and separating from it when rubbed, without papillae. Seeds reddish-black, keeled. Embryo horizontal, rarely obliquely or vertically orientated.

One species mostly distributed in Central Asia and alien in many parts of temperate Eurasia and North America.

###### 1. Teloxys aristata

Taxon classificationPlantaeCaryophyllalesChenopodiacea

(L.) Moq., Ann. Sci. Nat. Bot., sér. 2, 1: 289 (1834)

≡Chenopodiumaristatum L., Sp. Pl.: 221 (1753). **Lectotype** (designated by Iamonico and Jarvis 2012): Herb. Linn. 313.24 (LINN! image available at http://linnean-online.org/3149/).
≡Dysphaniaaristata (L.) Mosyakin & Clemants, Ukrainsk. Bot. Zhurn. 59(4): 383 (2002).
=Chenopodiumvirginicum L., Sp. Pl.: 222 (1753). **Lectotype** (designated by Reveal in Jarvis (2007)): Herb. Linn. 313.25 (LINN, image available at http://linnean-online.org/3150/).

####### Taxonomic note.

An ecological race of *T.aristata* without aristae mostly occurring in wet habitats.

####### Description.

See the genus description.

####### Habitat.

Gravelly substrates at elevations 3000–4000 m a.s.l. A rare plant in Himalaya.

####### Phenology.

Flowering: July-September; fruiting: August-October.

####### Distribution.

See Fig. 29.

**Figure 29.**
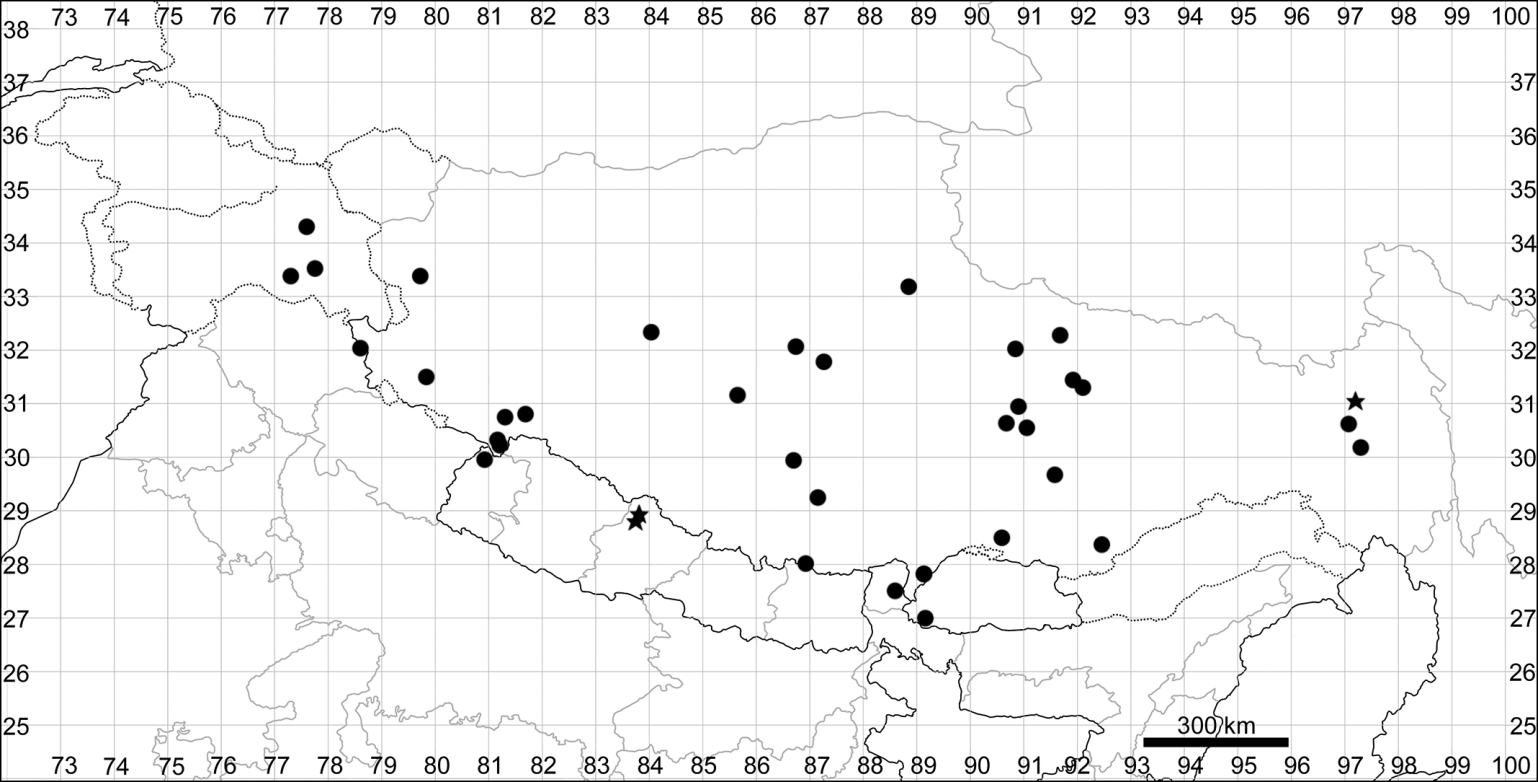
Distribution map of *Teloxysaristata* (stars) and *Axyrisprostrata* (circles).

####### Specimens examined.

**CHINA: Xizang: Qamdo Prefecture**: [Zhag’yab (Chaya) County] Gyitang (Jitang), 3000 m a.s.l., 20 Sep 1976, *Wu* et al. *6002* (KUN0587074);

**NEPAL**: **Western**: **Dhaulagiri Zone**: Mustang Distr., Khinga, 3100 m a.s.l., 21 Aug 1994, *S. Noshiro* et al. *9485511* (E00435336); Mustang Distr., Khinga, 24 Sep 1994, *Nakarmi 180/1994* (TUCH); Mustang Distr., Lower Chalungpa Khola, 28°54'N, 83°47'E, 3300 m a.s.l., 28 Aug 2001, *S. & G. Miehe & Koch 01-087-28* (herb. Miehe); Mustang Distr., Dankardzong, 28°50'N, 83°45'E, 3400 m a.s.l., 10 Sep 2001, *S. & G. Miehe & Koch 01-133-05* (herb. Miehe).

####### General distribution.

Central Asia, alien and ephemerophyte in many temperate regions of Eurasia and North America. In some of the herbaria visited, the specimens from Xizang identified as *Chenopodiumaristatum* (≡*Teloxysaristata*) indeed belong to *Dysphania* species, although its wider occurrence in this province has been predicted. There are no specimens from India, but the species could be found in some areas (especially in Jammu & Kashmir).

#### Tribe Axyrideae G.Kadereit & Sukhor., Am. J. Bot. 97(10): 1682 (2010)

##### 9. Axyris

Taxon classificationPlantaeCaryophyllalesChenopodiacea

L., Sp. Pl.: 979 (1753)

###### Lectotype

(designated by Jonsell and Jarvis in Jarvis et al. 1993): *Axyrisamaranthoides* L.

###### Description.

Monoecious annuals covered with stellate hairs sometimes intermixed with simple multicellular hairs. Leaves short- or long-petiolate, blade ovate, oblong, spatulate or lanceolate, entire, rarely crisp. Male flowers arranged in terminal spike-like inflorescences up to 8.0 cm long, with minute perianths of 3–5 free hyaline segments and with 2–5 stamens; female flowers located in the bract axils, with five perianth segments. Fruits always dimorphic (heterocarpous); pericarp tightly adjoining the seed coat with small ear-like appendages at the apex of the fruit. Seeds also dimorphic (with thick and thin seed-coat testa). Embryo vertical, horseshoe-shaped or annular; perisperm present.

Six species in Eurasia, predominantly in Central Asia; one (*A.amaranthoides*) is found as an alien plant in many parts of Europe and North America. Fruit morphology and anatomy as well as the peculiarities in plant pubescence are considered the most valuable characters for species delimitation (Sukhorukov 2005, 2011).

##### Key to the species

**Table:** 

1	Central ray of stellate hairs on the leaves (especially on petioles), the stem and branches 0.7–2.5(3.0) mm long; black fruits subglobose or spheroidal, brown ones compressed	**3. *A.sphaerosperma***
–	Central ray of stellate hairs (at least of those on the leaves) much shorter (up to 1.0–1.5 mm long); fruits of both (black and brown) types compressed	**2**
2	Plant with a prominent main stem; branches ascending; brown (or reddish-brown) fruits (1.6)2.0–2.4 mm long, oblong	**2. *A.mira***
–	Plant without a pronounced main stem; branches prostrate or ascending; brown fruits 1.3–1.8(2.2) mm long, pear-like	**1. *A.prostrata***

###### 1. Axyris prostrata

Taxon classificationPlantaeCaryophyllalesChenopodiacea

L., Sp. Pl.: 980 (1753)

####### Lectotype

(designated by Sukhorukov 2005): Herb. Linn. 1101.6 (LINN! image available at http://linnean-online.org/10715/).

The synonyms are listed in Sukhorukov (2005).

####### Description.

Annual with several prostrate stems up to 25 cm long; at high elevations (4000–5000 m), the plant often has a pincushion habit with small branches and short internodes. Leaves long-petiolate, spatulate, up to 3.5 cm, entire, with longer (up to 1.5 mm) stellate hairs near the leaf base, substituted in other parts by short-rayed hairs. Male inflorescence up to 2.0 cm. Perianth of the female flowers with scattered simple hairs. All fruits compressed; black fruits 1.5–2.2 mm long, oblong, with hardly noticeable or indistinct ear-like appendages; brown fruits 1.3–1.8(2.2) mm long, pear-like, with small appendages.

####### Habitat.

Stony places, alpine steppes, often between *Kobresia* cushions, ruderal sites; 2800–5500 m a.s.l.

####### Phenology.

Flowering: August-September; fruiting: September-October.

####### Distribution.

See Fig. 29.

####### Specimens examined.

**BHUTAN**: Jemu La, 12 Sep 1912, *R. Lepcha 366* (E);

**CHINA**: **Xizang**: Kham (Tibet), Mekong valley, [year] 1900, *V.F. Ladygin 469* (LE); **Ngari Prefecture**: Zanda (Zhada) County, 4500 m a.s.l., 7 Aug 1974, *Biological Institute Tibet Expedition Team 3853* (HNWP40812); Rutog (Ritu) County, 5000 m a.s.l., 12 Aug 1976, *Qinghai-Tibet Team Vegetation Group 13163* (PE00235057); Gêrzê (Gaize) County, 4570 m a.s.l., 10 Sep 1976, *Qinghai-Tibet Team 10213* (PE00121993, KUN0586542); Coqên (Cuoqin) County, Cêri (Cishi) Distr., 4600 m a.s.l., 17 Sep 1976, *Qinghai-Tibet Team 10329* (PE00121991); Burang County, Kangrinboge Feng, 4700 m a.s.l., 25 Aug 1990, *Y. Fei* et al. *516* (KUN0586539); Burang County, Kejia, 3700 m a.s.l., 29 Aug 1990, *Y. Fei* et al. *653* (KUN0584556); Manasarovar lake (Mapam Yumtso), 4800 m a.s.l., 17 Sep 2006, *Baklanova 826* (MW); Burang (Pulan) County, E of Hor, 4682 m a.s.l., 30°42'52.86"N, 81°42'54.21"E, 3 Sep 2012, *FLPH Tibet Expedition 12-0066* (PE01967958); **Nagqu Prefecture**: Amdo (Anduo) County, 4580 m a.s.l., 2 Aug 1961, *Wang 3683* (PE00121987); Shuanghu County, 4850 m a.s.l., 21 Jul 1976, *Qinghai-Tibet Team 10198* (PE00121990); Shuanghu County, 4800 m a.s.l., 2 Aug 1976, *Qinghai-Tibet Team Vegetation Group* (PE00235045); Shuanghu County, 60 km NW of Nyima (Nima), 4700 m a.s.l., 29 Aug 1976, *Qinghai-Tibet Team Vegetation Group 12381* (PE00121995); Xainza (Shenzha) County, Nyima (Nima) Distr., 4650 m a.s.l., Sep 1976, *Qinghai-Tibet Team 10353* (PE00121996); Upper Salween basin, 31°22'N, 91°56'E, 4570 m a.s.l., 4 Sep 1989, *B. Dickoré 4682* (MSB144305); Central-East Plateau, Amdo (Ragnag), 32°17'N, 91°43'E, 4600 m a.s.l., 10 Aug 1993, *G. & S. Miehe 9466/03* (herb. Miehe); Lake Xiketang, 32°01'N, 90°49'E, 4600 m a.s.l., 31 Jul 2005, *Nölling & Hanspach NX05-030-07* (herb. Miehe); S of Nagqu, Kema, 31°16'N, 92°07'E, 4535 m a.s.l., 10 Sep 2007, *G. & S. Miehe 07-018-04* (herb. Miehe); **Xigazê Prefecture**: Qomolangma Peak (Mount Everest), 5000 m a.s.l., 7 Jul 1959, *anonym 610* (PE00121988); Yadong (Chomo) County, near Pagri (Pali) Town, 4200 m a.s.l., 17 Sep 1974, *Qinghai-Tibet Team 74-2619* (PE00121984, KUN0586544); Nang La, Ngamring, 29°14'N, 87°12'E, 4530 m a.s.l., 25 Aug 2003, *G. & S. Miehe 03-038-05* (herb. Miehe); Changthang, between Shuru Tso & Sangsang, 29°54'N, 86°42'E, 13 Sep 2003, *G. & S. Miehe 03-096-19* (herb. Miehe); **Lhasa City**: Damxung (Dangxiong) County, 4700 m a.s.l., 5 Aug 1985, *Dong 056* (PE00510679); Lhasa valley, 40 km E of Lhasa, 4300 m a.s.l., 5 Aug 1989, *B. Dickoré 3735* (MSB144308); Lake Nam Tso, 5580 m a.s.l., 30°35'N, 90°42'E, 4775 m a.s.l., 26 Jul 2005, *Bless* et al. (herb. Miehe); Lake Namtso, 30°55'N, 90°53'E, 4730 m a.s.l., 6 Sep 2005, *Nölling & Hanspach NX05-215-01* (herb. Miehe); **Shannan Prefecture**: [Nêdong (Naidong) County] Zêtang (Zedang) to Lhünzê (Longzi) County, 4400 m a.s.l., 21 Aug 1960, *Fu 651* (PE00121986); Nagarze-Lhozak, SE of Pomo Co, 28°29'N, 90°33'E, 5060 m a.s.l., 23 Jul 1994, *B. Dickoré 9864* (MSB144306); **Qamdo Prefecture**: nr Bamda (Bangda) airport, 3000 m a.s.l., 16 Sep 1976, *Wu 5952* (KUN0586547); Bamda–Qamdo, N of Chudra, 30°39'N, 97°5'E, 4300 m a.s.l., 7 Jul 1994, *B. Dickoré 9005* (MSB144307);

**INDIA**: **Jammu & Kashmir**: Ladakh, Chortren Chen (Tog Nulla), 14000 ft a.s.l., 18 Aug 1931, *W. Koelz 2652* (E); Ladakh, Khardung La, 18 Aug 1982, *P.K. Hajra 74192* (BSD); Ladakh, Taglang La, 4820 m a.s.l., 13 Aug 1992, *H. Hartmann 4016* (MSB137908); Ladakh, Khardung La, 4440 m a.s.l., 8 Aug 1995, *H. Hartmann 5066* (MSB137907); Ladakh, Zanskar Region, Zara, 25 Aug 1998, *L. Klimeš 196* (PRA); Ladakh, Indus valley, Stot (E) [Stod River valley], Sumdo Gonma, 4770 m a.s.l., 7 Sep 2003, *L. Klimeš 3464* (PRA); **Sikkim**: Leonok, 15000 ft a.s.l., 4 Aug 1909, *Ribu 2812* (E); Gongchungchu, 17820 ft a.s.l., 16 Aug 1972, *R. Pradhan & al*. *120* (E);

**NEPAL**: **Far-Western**: **Mahakali Zone**: Nampa Gadh [probably Gurans Himal], 11000–12000 ft a.s.l., 25 Jul 1886, *J.F. Duthie* s.n. (K); **Western**: **Dhaulagiri Zone**: Mustang [distr.], 14500 ft a.s.l., 5 Aug 1954, *J. Stainton, W.R. Sykes & L.H.J. Williams 2194* (BM, E). Two additional records from Upper Mustang are reported by Yonekura (2008) (n.v.).

####### General distribution.

Central Asia, Siberia, Himalaya and Tibet. One of the widespread *Axyris* species. Common in Xizang (China) but rare in Central and Eastern Himalaya.

###### 2. Axyris mira

Taxon classificationPlantaeCaryophyllalesChenopodiacea

Sukhor., Willdenowia 41(1): 76 (2011)

####### Holotype.

[INDIA, Uttarakhand] Kumaon, Milam glacier, 12500 feet a.s.l., 28 Aug 1848, *R. Strachey & J.R. Winterbottom 2* (LE! isotypes BM! BR!).

####### Description.

Annual up to 40 cm, main stem erect with ascending branches, green or red, densely covered with short-rayed (up to 1.0 mm) stellate hairs at least in its upper part. Leaves short-petiolate, oblong or ovate, up to 6.0 × 2.0 cm, with short-rayed stellate hairs, dark green. Male inflorescence up to 3.0 cm long. Perianth of the female flowers with dense and large simple hairs. All fruits compressed, oblong; black fruits 1.5–1.8 mm long with very small (up to 0.15 mm) appendices on the pericarp that are indistinct in most cases; brown or reddish-brown fruits (1.6)2.0–2.4 mm long.

####### Habitat.

Grassy (sub)alpine vegetation, *Juniperus* communities, sands, sometimes ruderal sites at elevations of 2600–4500 m a.s.l.

####### Phenology.

Flowering: August-September; fruiting: September-early November.

####### Distribution.

See Fig. 30.

**Figure 30.**
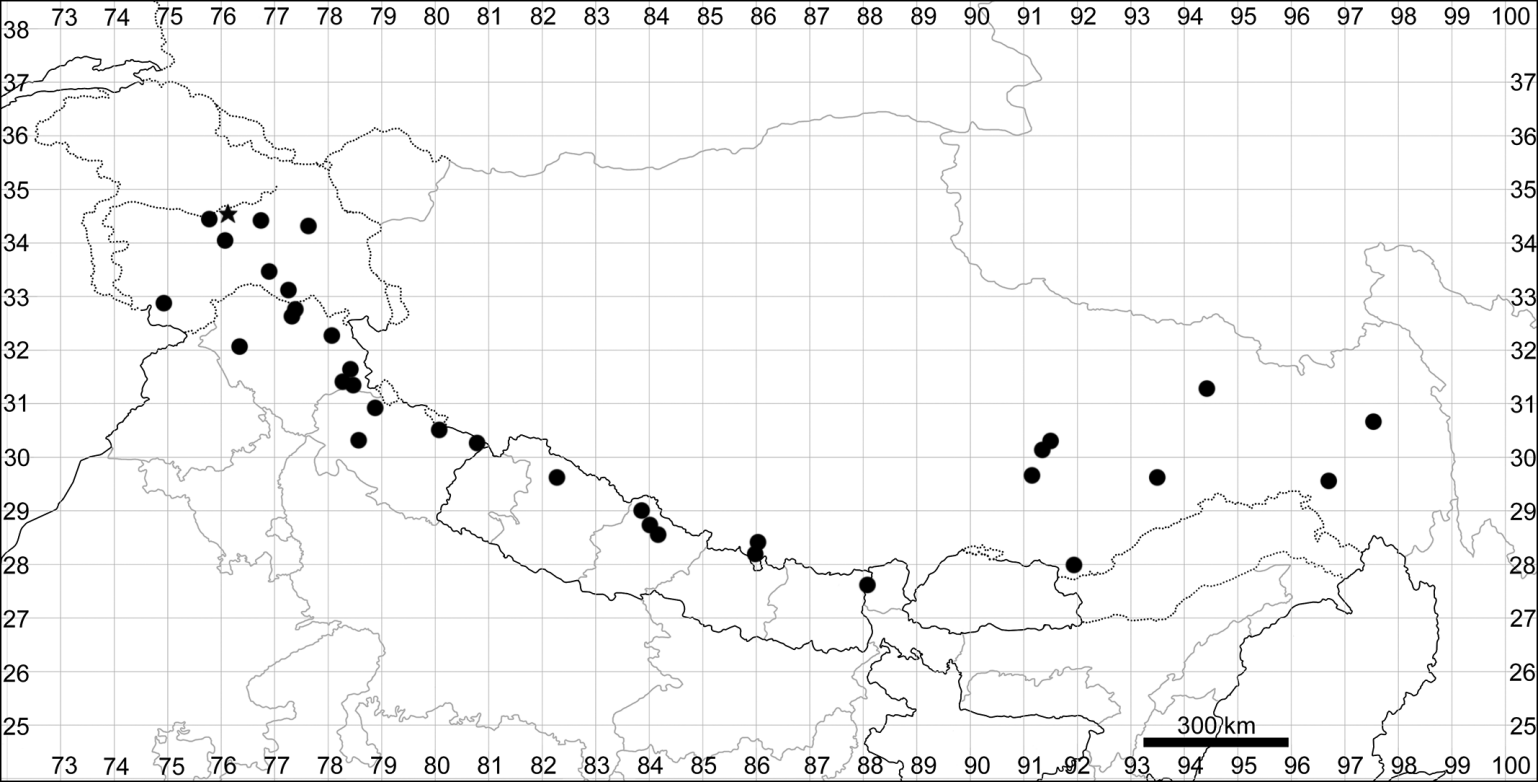
Distribution map of *Axyrismira* (circles) and *A.sphaerosperma* (star).

####### Specimens examined.

**CHINA**: **Xizang**: Kham (Tibet), Mekong valley, 1900, *V.F. Ladygin 417* (LE); **Xigazê Prefecture**: Nyalam (Nielamu) County, 3800 m a.s.l., 4 Sep 1972, *Tibet Chinese Herbal Medicine Survey Team 1755* (PE00121973); N of Nyalam, Koya, 28°22'N, 86°02'E, 24 Aug 1999, *G. & S. Miehe & Koch 99-150-27* (herb. Miehe); **Lhasa City**: Reting, N of Lhasa, 14000 ft a.s.l., 29 Sep 1942, *F. Ludlow & G. Sherriff 9093* (BM, E); Lhünzhub (Linzhou) County, Poindo (Pangduo), 4050 m a.s.l., 7 Oct 1972, *Tibet Chinese Herbal Medicine Survey Team 1928* (PE00121974); [Lhünzhub (Linzhou) County] Upper Kyi Chu, nr Reting Monastery, 30°18'N, 91°31'E, 4300 m a.s.l., 22 Aug 1997, *G. Miehe* et al. *97-027-23* (MSB147363); **Shannan Prefecture**: Cona (Cuona) County, 4300 m a.s.l., 24 Aug 1975, *Qinghai-Tibet Team Additional Group 751507* (PE00121982, PE00121983); **Nyingchi Prefecture**: [Gongbo’gyamda County] Kongpo, between Doshong and Deyang, 9500 ft a.s.l., 28 Jul 1938, *F. Ludlow* et al. *5458* (BM, LE); **Qamdo Prefecture**: Baxoi (Basu) County, Rawu (Ranwu) Town to Kamsa (Kangsha), 3900 m a.s.l., 29 Aug 1973, *Qinghai-Tibet Team 73-1332* (PE00121971, KUN0586538); [Baxoi (Basu) County], N of Rawu (Ranwu) town, 4000 m a.s.l., 29 Aug 1976, *Wu* et al. *5152* (KUN0586537); Banbar (Bianba) County, Shading Distr., 3700–3900 m a.s.l., 8 Sep 1976, *Qinghai-Tibet Team 11224* (PE00121972);

**INDIA**: **Jammu & Kashmir**: Ladakh, Chortren Chen [Tog Nulla], 18 Aug 1931, *W. Koelz* s.n. (W); Ladakh, Domel, 11000 ft a.s.l., 5 Aug 1946, *R.R*. *Stewart 22191* (K); Ladakh, Dras to Mitsahoi, 10000–11000 ft a.s.l., 12 Aug 1946, *R.R*. *Stewart 22336* (K); Ladakh, 3940 m a.s.l., 27 Aug 1976, *H. Hartmann 2122* (G152731; MSB137906); Ladakh, Kardung La, 3800 m a.s.l., 9 Aug 1980, *U.C. Bhattacharyya 71554* (BSD); Ladakh, Indus valley, Domkhar Dha, Khargog, 3930–4040 m a.s.l., 28 Aug 2002, *L. Klimeš 2436* (PRA); Ladakh, Indus valley, Domkhar Dha, 4020–4060 m a.s.l., 19 Aug 2003, *L. Klimeš 3090* (PRA); Ladakh, Suru Region, 4240 m a.s.l., 21 Aug 2003, *L. Klimeš 3142* (PRA); Ladakh, Zanskar Region, S of Padum, 4150 m a.s.l., 23 Aug 2003, *L. Klimeš 3200 & 3204* (PRA); Zanskar, Tsarap, 4550–4600 m a.s.l., 26 Aug 2004, *L. Klimeš 4236* (PRA); **Himachal Pradesh**: Kunawer [Kinnaur Distr.], 10000–14000 ft a.s.l., *T.T. Thomson* s.n. (K, L, P04929721); Kunawer [Kinnaur], 10000–14000 ft a.s.l., 1859, *J.D. Hooker & T.T. Thomson* s.n. (P); Lahaul, Sep 1869, *anonym 114* (K); Kinnaur, *J. Drummond 22291* (P04970122); Kangra Distr., Sep 1933, *W. Koelz 7113* (G); [Kinnaur Distr.] Chitkul, 20 Aug 1934, *N. Parmanand 1042* (E00392967); Lahaul, 10000 ft a.s.l., 7 Aug 1916, *R.E. Cooper & A.K. Bulley 5299* (E); Lahaul Distr., 12500 ft a.s.l., 14 Jul 1938, *N.L. Bor 9468* (E, K); [Kinnaur Distr.] Chitkul, 3300 m a.s.l., 28 Sep 1964, *N.C. Nair 34383* (BSD); Sangla, 2900 m a.s.l., 29 Sep 1964, *N.C. Nair 34116* (BSD); Spiti, 4200 m a.s.l., 30 Jul 1972, *U.C. Bhattacharyya 49157* (BSD); Lahaul, Barlachala, 4450 m a.s.l., Aug 2000, *S. Singh 76* (BSD); **Uttarakhand**: Tehri Garhwal [Distr.], 8000 ft a.s.l., Sep [18]82, *J.F. Duthie 2262* (K); Kumaon, Kutti, 13000–14000 ft a.s.l., 8 Sep [18]94, *J.F. Duthie 3326* (BM, G); Kumaon [Distr.], 14000 ft a.s.l., *T.T. Thomson* s.n. (K); Tehri Garhwal [Distr.], Rudugaira, 13200 ft a.s.l., 17 Sep 1952, *P. Huggins 74* (BM);

**NEPAL**: **Midwestern**: **Karnali Zone**: [Mugu Distr.] Karnali valley, between Mangri and Daura, 8500 ft a.s.l., 16 Aug 1952, *O. Polunin, W.R. Sykes & L.H.J. Williams 5251* (BM, E); **Western**: **Dhaulagiri Zone**: Mustang [Distr.], 15000 ft a.s.l., 5 Aug 1954, *J. Stainton, W.R. Sykes & L.H.J. Williams 2190* (BM); **Gandaki Zone**: Manang Prov. [Distr.], Marsyandi valley, between Pisang & Onigre, 3200 m a.s.l., 25 Sep 1969, *T. Wraber 403* & *36504* (BM); [Manang Distr.] Braga–Thorong pass, 11000 ft a.s.l., Nov 1978, *P.H*. *Davis 115* (K); Manang Distr., Yak-Kharka, 3850 m a.s.l., 17 Aug 1994, *M. Mikage* et al. *9460382* (BM, E00156347); **Eastern**: **Mechi Zone**: [Taplejung Distr.] Talung, 2600 m a.s.l., 3 Oct 1971, *J.-F. Dobremez 71*-*25* (E00014374).

####### General distribution.

Himalaya and Tibet.

###### 3. Axyris sphaerosperma

Taxon classificationPlantaeCaryophyllalesChenopodiacea

Fisch. & C.A.Mey. in Fischer, Meyer & Ave-Lallemant, Ind. Sem. Hort. Petrop. 6: 46 (1839)

####### Lectotype

(designated by Sukhorukov 2005): [RUSSIA, Altai] “Lecti in regione orientali fl. altaicae Tschuja” [Chuya river valley in Altai], [year] 1839, [*anonym* s.n.] (LE!).

####### Description.

Annual up to 40 cm, erect; stem with stellate hairs having a large central ray of 0.7–2.5(3.0) mm long, such hairs especially abundant on stems and leaf petioles. Leaves short-petiolate or subsessile, up to 5 cm, oblong. Male inflorescence up to 2.0 cm long. Perianth of the female flowers with sparse, but large simple hairs. Fruits of two types: subglobose, black, 1.4–1.6 mm with hardly noticeable or indistinct ear-like appendices; the second type ± compressed, ovoid, blackish, 1.6–2.0 mm, also with hardly noticeable or indistinct ear-like appendices.

####### Habitat.

Stony places, alpine vegetation, sometimes ruderal sites at elevations of 3000–4000 m a.s.l.

####### Phenology.

Flowering: July-September; fruiting: September-October.

####### Distribution.

See Fig. 30.

####### Specimens examined.

**INDIA**: **Jammu & Kashmir**: [Ladakh] Purig [Kargil], Jul 1933, *W. Koelz* (H1038357). The identification of this specimen comprising only young plants is based on the pubescence details (for more, see Sukhorukov 2011).

####### General distribution.

Central Asia, Siberia and Himalaya. The southernmost border of the range is in N Himalaya.

##### Species excluded

###### Axyris amaranthoides

Taxon classificationPlantaeCaryophyllalesChenopodiacea

L., Sp. Pl.: 979 (1753)

####### Notes.

This species was long considered in broader sense by the authors of Himalayan floras (Hooker 1890, Hemsley 1902, Strachey and Duthie 1906, Bamber 1916) and included *Axyrishybrida* and *A.prostrata*. Subsequently all records were re-identified as *A.mira* or *A.prostrata* (Sukhorukov 2011). The southernmost records of *A.amaranthoides* were discovered outside of our region, e.g. in the provinces of Shaanxi, Gansu, Xinjiang in China (Sukhorukov 2014).

###### Axyris hybrida

Taxon classificationPlantaeCaryophyllalesChenopodiacea

L., Sp. Pl.: 980 (1753)

####### Notes.

The records of *A.hybrida* in Xizang and Yunnan (Zhu et al. 2003) belong to *A.mira* (Sukhorukov 2011), and the distribution of the former species lies in the northern part of Central Asia (including Asian Russia, Mongolia, E Kazakhstan, Kyrgyzstan, Tajikistan and N and NE China).

###### Axyris villosa

Taxon classificationPlantaeCaryophyllalesChenopodiacea

Moq. in DC., Prodr. 13(2): 117 (1949)

####### Notes.

This name is a synonym of *Bassialasiantha* Freitag & G.Kadereit (Sukhorukov 2011).

###### 10. Krascheninnikovia

Taxon classificationPlantaeCaryophyllalesChenopodiacea

Gueldenst., Nov. Comm. Acad. Petrop. 16: 551 (1772)

K. ceratoides (L.) Gueldenst (**Type**).

####### Description.

Subshrubs or shrubs up to 2 m (much smaller at higher altitudes). Stem and leaves covered with stellate hairs often turning fulvous when dry. Leaves alternate, short-petiolate, entire, linear, oblong or ovate, basally cuneate to slightly cordate, green. Flowers unisexual (plants monoecious); male flowers agglomerated in dense spike-like inflorescences that terminate the branches, perianth hyaline with 3–5 almost free segments covered with stellate but easily caducous indumentum, stamens 3–5; female flowers positioned below the male ones, enclosed by a cover consisting of two at least halfway fused accrescent bracts with long simple hairs and much smaller stellate hairs. Styles 2. Fruits with hyaline, very thin pericarp covered with scattered stellate hairs. Seeds ~2.0 mm with thin seed coat, perisperm abundant. Embryo vertical, horseshoe-shaped.

One species in the deserts of Eurasia and North America. According to recent investigations (Heklau and Röser 2008), the genus comprises one species.

###### 1. Krascheninnikovia ceratoides

Taxon classificationPlantaeCaryophyllalesChenopodiacea

(L.) Gueldenst., Nov. Comm. Acad. Petrop. 16: 555 (1772)

≡Axyrisceratoides L., Sp. Pl.: 979 (1753). Lectotype (designated by Hedge 1997): Herb. Linn. 1101.1 (LINN! image available at http://linnean-online.org/11529/). The full synonymy is given by Heklau and Röser (2008).

####### Taxonomic notes.

Morphologically, two forms of K.ceratoides can be distinguished based on the shape of the leaf base; some specimens in Himalaya have slightly cordate or truncate leaves while others have cuneate leaves. In Tibet, a form with cuneate leaves is found.

####### Description.

See the genus description.

####### Habitat.

Gravelly hill slopes, steppes and cold deserts at elevations of 2700–5300 m (depending on the region), one of the dominants of cold stony deserts (together with Juniperus and Caragana spp.).

####### Phenology.

Flowering: July-September; fruiting: September-October.

####### Distribution.

See Fig. 31.

**Figure 31.**
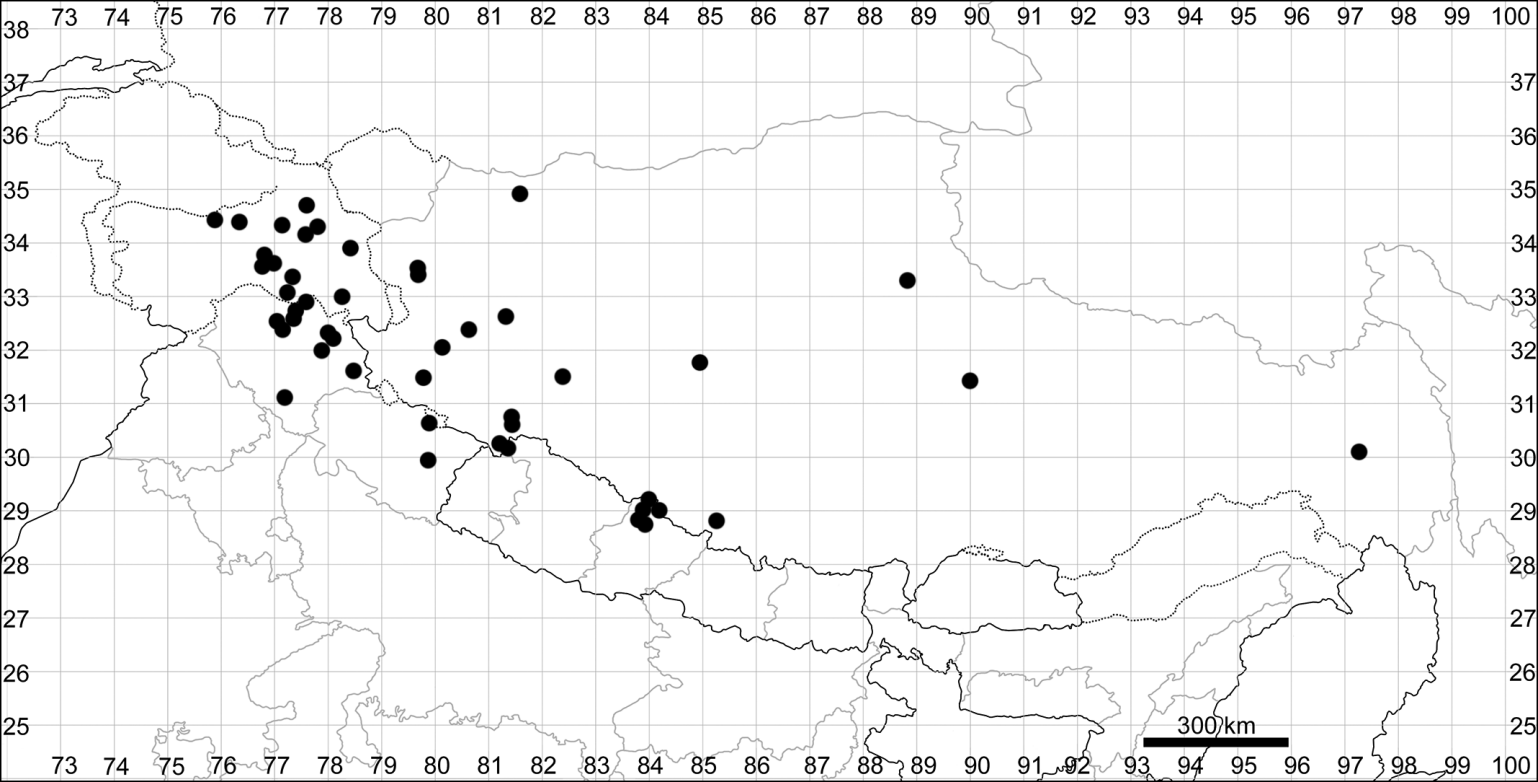
Distribution map of Krascheninnikoviaceratoides.

####### Specimens examined.

**CHINA**: **Xizang**: **Ngari Prefecture**: Rutog (Ritu) County, W of Lake Bangong (Pangong Tso), 4300 m a.s.l., 13 Jul 1974, *Biological Institute Tibet Expedition Team 3653* (HNWP40750); Zanda (Zhada) County, 4000 m a.s.l., 7 Aug 1974, *Biological Institute Tibet Expedition Team 3836* (HNWP40739); Burang (Pulan) County, Mapam Yumtso (Lake), 4530 m a.s.l., 23 Aug 1974, *Biological Institute Tibet Expedition Team 4142* (HNWP40744); Gêrzê (Gaize) County, Datan, 4440–4600 m a.s.l., 8 Sep 1974, *Biological Institute Tibet Expedition Team 4328* (PE00146672); Rutog (Ritu) County, S of Yaxier Tso (Bangdag Tso) Lake, 5100 m a.s.l., 12 Aug 1976, *Qinghai-Tibet Team Vegetation Group 13154A* (PE00146791); Gê’gyai (Geji) County, Yagra (Yare) Distr., 5000 m a.s.l., 13 Aug 1976, *Qinghai-Tibet Team 76-8695* (KUN0586652); Rutog (Ritu) County, 5050 m a.s.l., 3 Oct 1976, *Qinghai-Tibet Team Vegetation Group 13585* (PE00146798); Gê’gyai (Geji) County to Shiquanhe, 4300 m a.s.l., 21 Aug 1990, *Y. Fei* et al. *419* (KUN0264918); Burang County, Kejia, 3700 m a.s.l., 29 Aug 1990, *Y. Fei* et al. *582* (KUN0264916); Rutog County, Banggong Lake, 4300 m a.s.l., 3 Sep 1990, *Y. Fei* et al. *714* (KUN0264912); Upper Karnali, Burang, 30°10'N, 81°20'E, 4440 m a.s.l., 30 Aug 1993, *G. & S*. *Miehe 9610/02* (MSB147333); Upper Hindus basin, Gar Zangpo, S of Gar, 32°1'N, 80°6'E, 5 Sep 1993, *G. & S. Miehe 9659/05* (MSB147336); Changtang, 32°38'N, 81°20'E, 4870–4950 m a.s.l., 7 Sep 1993, *G. & S. Miehe 9674/01* (MSB147334); Burang (Pulan) County, 30°45'23.23"N, 81°21'30.61"E, 4633 m a.s.l., 3 Sep 2012, *FLPH Tibet Expedition 12-0082* (PE01957661); Burang (Pulan) County, 30°9'12.86"N, 81°19'24.69"E, 3671 m a.s.l., 4 Sep 2012, *FLPH Tibet Expedition 12-0137* (PE01976597); **Nagqu Prefecture**: Baingoin (Ban’ge) County, Sêwa, 4800 m a.s.l., 22 Jun 1976, *Qinghai-Tibet Team Vegetation Group 11783* (PE00146802); Baingoin (Ban’ge) County, Sêwa, 5100 m a.s.l., 22 Jun 1976, *Qinghai-Tibet Team 9488* (PE00146795); Shuanghu County, 5100–5300 m a.s.l., 6 Jul 1976, *Qinghai-Tibet Team 9635* (PE00146671, KUN0586678); Shuanghu County, 5000–5150 m a.s.l., 8 Aug 1976, *Qinghai-Tibet Team 9969* (PE00146670); **Xigazê Prefecture**: Upper Trisuli gorge, NW of Mt. Xixabangma, 28°48'N, 85°18'E, 4060 m a.s.l., 25 Aug 1993, *G. & S. Miehe 9569/08* (MSB147332); **Qamdo Prefecture**: Baxoi (Basu) County, along Nu River, 3000 m a.s.l., 5 Jul 1976, *Qinghai-Tibet Team 12207* (KUN0586641);

**INDIA**: **Jammu & Kashmir** (selected specimens): Ladakh, Leh [without date], *W. Moorcroft 6950* (LE, syntype of Axyrismoorcroftiana); Nubra, 15 Aug 1856, Schlagintweit 2441 (P04913029); Ladakh, Tankse, 13000 ft a.s.l., 30 Jul 1931, *W. Koelz 2460* (E); Zanskar, Inle, 17000 ft a.s.l., 21 Sep 1931, *W. Koelz 3008* (E); between Dras & Kharbu, 10000 ft a.s.l., 9 Jul 1937, *C.C*. *Burt 14* (E); Ladakh, Ruphu, 145000 ft a.s.l., 21 Jul 1941, *F. Ludlow & G. Sherriff 8513* (BM, E, LE); Ladakh, Kurgiakh, 4300 m a.s.l., 25 Jul 1973, *U.C. Bhattacharyya 52249* (BSD); nr Leh, Aug 1966, *H. Singh* s.n. (BSD); Ladakh, Mulbekh, 3570 m a.s.l., 20 Jul 1976, *H. Hartmann 2129* (MSB160563); Ladakh, nr Lingshed, Parfi La, 3700–4000 m a.s.l., 4 Sep 1979, Bruhn s.n. (M69976); Ladakh, Chusul, 4300 m a.s.l., 18 Aug 1980, *U.C. Bhattacharyya 71703* (BSD); Ladakh, Zanskar Region, Zara, 4740 m a.s.l., 22 Aug 1998, *L. Klimeš 2836* (PRA); Ladakh, Leh, Ganglas, 3890–3950 m a.s.l., 30 Jul 2001, *L. Klimeš 1164* (PRA); Ladakh, Indus valley, Stot (E) [Stod River valley], 4260 m a.s.l., 17 Aug 2002, *L. Klimeš 2185* (PRA); Ladakh, Rupshu Region, Tso Moriri, 5120 m a.s.l., 6 Aug 2002, *L. Klimeš 2045* (PRA); Ladakh, Pangong, 4300 m a.s.l., 10 Sep 2002, *L. Klimeš 2708* (PRA); Ladakh, Shyok Region, Digar vill., 3710–3790 m a.s.l., 25 Sep 2004, *L. Klimeš 5056* (PRA); Ladakh, Indus valley, Sham (W), Chagatse La, 3500–3600 m a.s.l., 23 Sep 2005, *L. Klimeš 6516* (PRA); **Himachal Pradesh**: Labrang, 9000 ft a.s.l., 18 Aug 1890, *J.H*. *Lace 535* (E); Lahaul, 11000 ft a.s.l., 27 Aug 1916, *R.E*. *Cooper 5515* (E); Lahaul, Sarchu, 13000 ft a.s.l., 30 Jun 1941, *N.L. Bor 15040* (E); Lahaul, Kolung, 12 Aug 1930, *W. Koelz 1034* (P04942061); Lahaul, Bara-lacha La, 14500 ft a.s.l., 18 Jul 1976, *J. Stainton 7685* (E); Lahaul, Kyelang, 5 Aug 1971, 3100 m a.s.l., *U.C. Bhattacharyya 45097* (BSD); Kinnaur distr., [year] 1886, *Drummond 22292* (E); Zarchu, 4321 m a.s.l., Aug 2000, *S. Singh* s.n. (BSD); Pin Valley National Park, 3700 m a.s.l., 11 Jul 2002, *K.C. Sekar 100412* (BSD); Spiti valley, Kaza, 11 Aug 1994, *S.K. Murti & S. Singh 88257* (BSD); Chikam, Spiti valley, 4228 m a.s.l., 22 Sep 2002, *S. Singh* s.n. (BSD); **Uttarakhand**: [Kumaon] Gnari Khorsum, Jul 1856, Schlagintweit 6613 (BM, E); Chamoli Distr., 15 Sep 1975, *B.D. Naithani 56267* (BSD); Chamoli Distr., Malari, [no date and collector] *87564* (BSD);

**NEPAL**: **Western**: **Dhaulagiri Zone**: Mustang Distr., Muktinath, 11500 ft a.s.l., 25 Jun 1954, J. Stainton, W.R. Sykes & L.H.J. Williams 1398 (BM, E, LE); Damodar Kund (N of Muktinath), 31 Jul 1954, J. Stainton, W.R. Sykes & L.H.J. Williams 2107 (BM); Yara (S of Mustang), 12000 ft a.s.l., 2 Aug 1954, J. Stainton, W.R. Sykes & L.H.J. Williams 2134 (BM, E); Mustangbhot, 29°11'N, 83°58'E, 3500 m a.s.l., 22 Aug 1956, *F. Lobbichler* s.n. (BM); [Mustang Distr.], Ghiling, 29°0'N, 83°52'E, 3800 m a.s.l., 17 May 1974, *J.-F*. *Dobremez 3008* (BM, E00214385); Mustang [Distr.], Bara Gaon, 16 Jul 1998, *W.R*. Sykes 336/98 (E00649123); Mustang [Distr.], Lo Tsho Dyun, Tangbe (Tangya) area, 18 Jul 1998, *W.R*. Sykes 317/98 (E00647146); Mustang [Distr.], Bara Gaon, Kagbeni area, 31 Aug 1998, *W.R*. Sykes 306/98 (E00649111); Mustang Distr., Chuksang (2970 m a.s.l.)–Tetang (3000 m a.s.l.)–Gnyu Pass (4100 m a.s.l.), 28°55'N, 83°51'E, 13 Jul 2000, *Y. Iokawa* et al. *20020158* (E00246125); Mustang distr., Dhi towards Lo La, 3880 m a.s.l., 14 Aug 2001, *S. Noshiro* et al. *20103048* (E00238916).

####### General distribution.

Widely distributed in the steppes, deserts and alpine vegetation of Eurasia and North America (in the latter region, K.ceratoides is represented by subsp. lanata).

### Subfam. Betoideae Ulbrich in Engler & Prantl, Nat. Pflanzenfam. ed. 2, 16c: 455 (1934)

#### 11. Acroglochin

Taxon classificationPlantaeCaryophyllalesChenopodiacea

Schrad. in Roem. & Schult., Mant.: 69 (1822)

Acroglochin chenopodioides Schrad. (=A.persicarioides (Poir.) Moq.) (**Type**).

##### Description.

Glabrous or scarcely pubescent annual up to 50(70) cm, branches often terminating in acicular apices. Leaves long-petiolate, up to 10.0 cm, broadly ovate or ovoid, dentate or erose, teeth straight or incurved, tip mucronate, blades glabrous or sparsely covered with simple, often curved hairs. Inflorescences in the leaf axils, quite short, falsely dichotomous. Perianth of 5 free segments, keeled along midrib. Stamens 2, anthers small, 0.2–0.3 mm, without appendages. Stylodia concrescent into column in their lower half. Fruit dehiscent by a lid; pericarp white or greenish with several homocellular layers. Seeds dark-red, depressed-globular, ~1.3 mm in diameter, smooth.

One species in Himalaya and West and Central China.

#### 1. Acroglochin persicarioides

Taxon classificationPlantaeCaryophyllalesChenopodiacea

(Poir.) Moq. in DC., Prodr. 13(2): 254 (1849).

≡Amaranthuspersicarioides Poir. in Lam. & Poir., Encycl.: 311 (1810). **Type**: Nepal/Kashmir (not designated, P?).
=Acroglochinchenopodioides Schrad. in Roem. & Schult., Mant.: 227 (1822). **Type**: n.v. (existence not certain).
=Lecanocarpusnepalensis Nees in Nees & Sinning, Amoen. Bot. Bonn. 2: tab. 2 (1824). **Type**: not found.
=Boehmeriaamarantus H.Lév., Repert. Spec. Nov. Regni Veg. 11: 550 (1913). **Holotype**: CHINA, Kouy-Tcheou [Guizhou] Prov., environs de Gan-Pin, 29 Aug 1897, *Martin & Bodinier* s.n. (E00317870!).
=Amaranthusdiandrus Spreng., Neue Entdeck. Pflanzenk. 3: 20 (1822) syn. nov. **Lectotype** (Sukhorukov, designated here): NEPAL, Sep 1791, Spreng.[*el*] (L1677349!).

##### Note.

Although *Amaranthusdiandrus* has a very clear diagnosis (Sprengel 1822), it was an unresolved name (Das 2016) and is now added to the synonyms of *Acroglochinpersicarioides*.

##### Description.

See the genus description.

##### Habitat.

Hill slopes, disturbed areas at elevations of 1700–3200 m.

##### Phenology.

Flowering: July-September; fruiting: August-October.

##### Distribution.

See Fig. 32.

**Figure 32.**
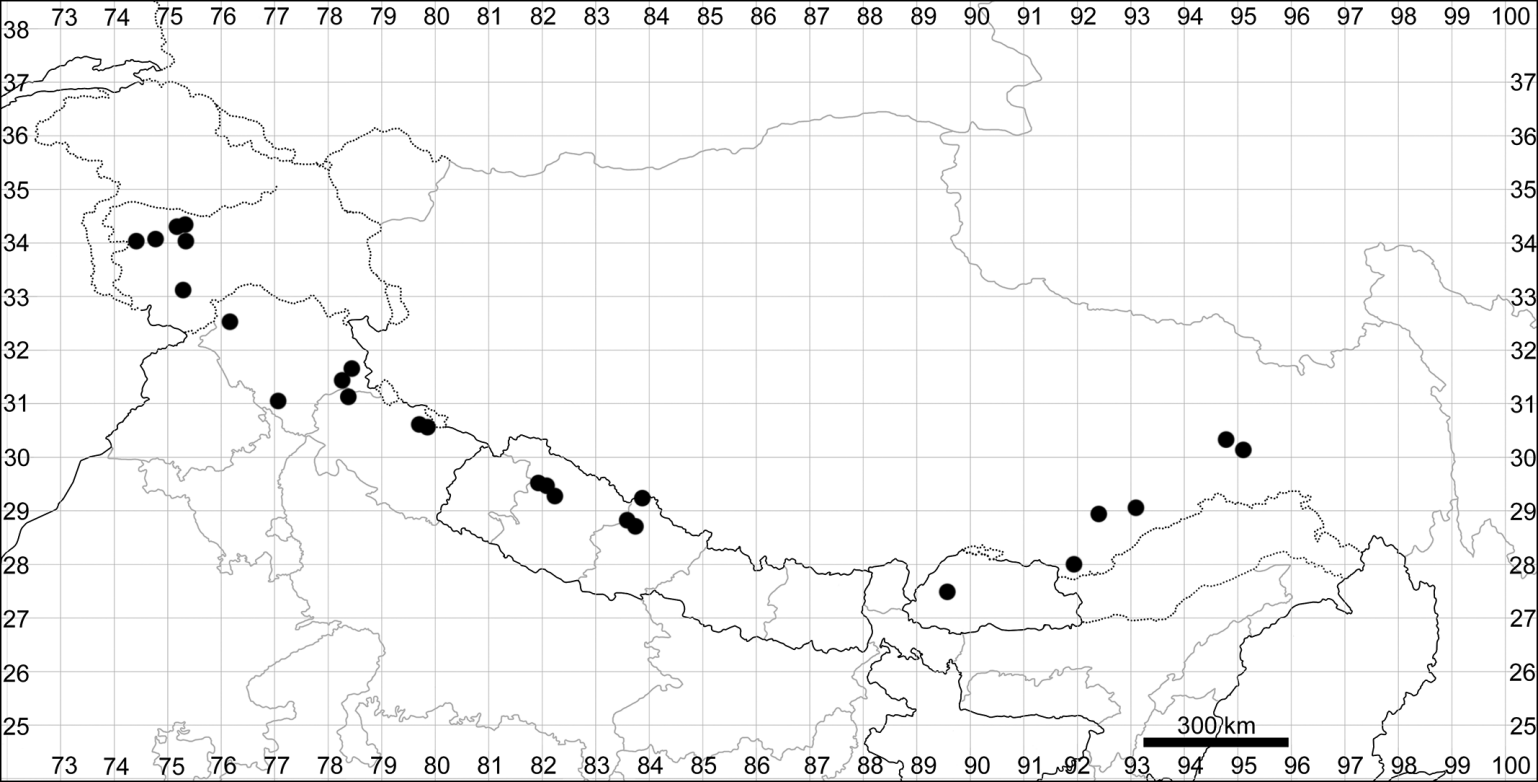
Distribution map of *Acroglochinpersicarioides*.

##### Specimens examined.

**BHUTAN**: Thimphu Distr., Thimphu, 2300 m a.s.l., 1 Oct 1988, *Wood 6756* (E00168224);

**CHINA**: **Xizang: Shannan Prefecture**: Cona (Cuona) County, 2600 m a.s.l., 26 Aug 1975, *Qinghai-Tibet Team Additional Group 751610* (PE00120390, KUN0586377); **Nyingchi Prefecture**: [Bomi County] Yi’ong (Yigong), 2200 m a.s.l., 19 Jul 1965, *Ying & Hong 650684* (PE00120388, PE00120389); [Bomi County] Tangmai (Tongmai) to [Nyingchi (Linzhi) County] Lunang (Lulang), 2210 m a.s.l., 27 Jul 1965, *Zhang & Lang 937* (PE00120386, PE00120387, KUN0586375); Nang (Lang) County, Nang (Lang) vill., 3100 m a.s.l., 16 Aug 1972, *Tibet Chinese Herbal Medicine Survey Team 4402* (PE00120385); Zayü (Chayu) County, 28°54'17.24"N, 92°27'29.78"E, Sep 2012, *FLPH Tibet Expedition 12-1194* (PE01967957);

**INDIA: Jammu & Kashmir**: [Ganderbal Distr.] Sonamarg, 1 Sep 1886, *C.B*. *Clarke 30881* (LE); Pahalgam, 7800 ft a.s.l., 31 Aug 1925, *R.R*. *Stewart 8425* (G); Srinagar, [without date] *Kaul 19657* (G); Kulan, 7500 ft a.s.l., 31 Aug 1956, *O. Polunin 56-610* (BM, E); Batote, 10 Sep 1958, *T.A. Rao 7337* (BSD); Ferozepur Nallah, 6 Sep 1960, *S.K. Malhotra 12227* (BSD); **Himachal Pradesh**: Kinnaur [Distr.], 1884, *Drummond 689* (DD); Bashahr, Sep 1891, *J.H. Lack 1149* (DD); Chamba, 10000 ft a.s.l., 17 Sep 1896, *G.A. Gammie 18621* (DD); [Kinnaur Distr.] Sangla, 29 Aug 1934, *N. Parmanand 1134* (E); Kinnaur Distr., Urmi, 2800 m a.s.l., 30 Aug 1963, *N.C. Nair 30130* (BSD); Baspa valley, Sangla, 20 Sep 1991, *K.P. Janardhanan 46048* (BSD); **Uttarakhand**: Garhwal, Jumma, 13 Aug 1974, *B.D. Naithani 53836* (BSD); [Chamoli Distr.] Dunagiri, 2600 m a.s.l., 16 Aug 1974, *B.D. Nailhani 54055* (BSD); Chamoli Distr., Aug 1988, *P.K. Hajra 87140* (BSD); Govind Pashu Vihar, 10 Sep 2010, *R. Manikawdan 112492* (BSD);

**NEPAL**: **Midwestern**: **Karnali Zone**: [Mugu Distr.] Mugu Khola, between Daura & Mugu, 10000 ft a.s.l., 17 Aug 1952, *O. Polunin, W.R. Sykes & L.H.J. Williams 5275* (BM, DD, E, G); [Jumla Distr.] Bharbhare vill., 25 Sep 2010, *A. Sukhorukov 421* (MW); Jumla Distr., Jumla vill., 29 Sep 2010, *A. Sukhorukov* s.n. (E, MW); [Mugu Distr.] Bhota vill., 2300 m a.s.l., 1 Oct 2010, *A. Sukhorukov 56* (MW); **Western: Dhaulagiri Zone**: many sheets from Mustang Distr., surroundings of Jomosom and Marpha vill. (BM, E, MW, P).

##### General distribution.

Himalaya and Tibet. Common in Central Himalaya at elevations of 2000–2600 m.

### Subfam. Corispermoideae Raf., Fl. Tellur. 3: 45 (1837)

#### 12. Corispermum

Taxon classificationPlantaeCaryophyllalesChenopodiacea

L., Sp. Pl.: 4 (1753)

##### Lectotype

(Hitchcock and Green 1929): *Corispermumhyssopifolium* L.

##### Description.

Annuals, branched from the base, often forming tumble-weed habit, covered with branched hairs, usually glabrescent at the fruiting stage. Leaves sessile, filiform to ovoid, green or greyish, sometimes red from both sides. Perianth absent or of 1–3 hyaline segments. Stamens 1–3, anthers 0.2–0.3 mm long. Stylodia 2 with persistent lower part. Fruit ovoid or roundish, 1.5–6.5 mm, glabrous or covered with branched (usually caducous) hairs, adaxially plain or slightly concave, abaxially convex; marginal wing membranous, well-developed or tiny. Fruit apex roundish, triangular or emarginate. Pericarp either adherent to the seed coat or its outer layer forms wart-like or (rarely) wavy detachments; groups of dark-brown cells filled with tannins are often present in the outer pericarp layer. Seed coat of two or more thin layers.

Fruit anatomy plays the most important role in the diagnostics of the species groups. The following characters can be noted (Sukhorukov 2007b): (1) thickness of the fruit, (2) thickness of the outer pericarp layer, (3) presence of sclereids in the medial fruit part and their quantity and orientation and (4) outline and size of the fruit wing.

The exact species number is still unknown, but it is estimated at approximately 80 (Sukhorukov 2014).

All species encountered in our area are alpine plants growing at elevations of 2800–5000 m.

#### Key to the species

**Table:** 

1	Fruit 4.0–5.5 mm long, emarginate at the top, with marginal wing 0.65–1.40 mm	**2**
–	Fruit up to 3.5(4.0) mm long, wing up to 0.7 mm	**4**
2	Fruit covered with stellate hairs, pericarp surface often with groups of dark-brown (tanniniferous) cells	*** C. lepidocarpum ***
–	Fruit glabrous	**3**
3	Plant moderately pubescent; bracts recurved; fruit wing 0.65–0.90 mm	**6. *C.lhasaense***
–	Plant almost glabrous; bracts not or slightly recurved; fruit wing (0.70)0.90–1.40 mm	**5. *C.pseudofalcatum***
4(1)	Plants dwarfish, up to 6 cm high with single stem or with short lateral branches; leaves linear, up to 1.5(2.0) mm wide	**2. *C.nanum***
–	Plants usually taller, with well-developed lateral branches; leaves linear to oblong	**5**
5	Fruit apex clearly emarginate, wing 0.45–0.60 mm; bracts often recurved at fruiting stage	**1. *C.falcatum***
–	Fruit apex rounded or triangular (but slightly emarginate between the styles)	**6**
6	Fruit 2.8–4.0 mm, apically strongly triangular; stellate hairs on the fruit present or absent; fruit wing 0.3–0.7 mm	**4. *C.dutreuilii***
–	Fruit up to 3.2 mm long, apically rounded or indistinctly triangular with wing up to 0.45 mm	**7**
7	Plants almost glabrous; fruit with stellate hairs and sometimes warts	**8. *C.gelidum***
–	Plants moderately pubescent; fruit glabrous, usually without warts	**8**
8	Fruit wing ~0.3 mm	**9. *C.tibeticum***
–	Fruit wing smaller, 0.15–0.30 mm	**3. *C.pamiricum***

##### 1. Corispermum falcatum

Taxon classificationPlantaeCaryophyllalesChenopodiacea

Iljin, Izv. Glavn. Bot. Sada SSSR 28: 644 (1929)

###### Lectotype

(Sukhorukov, designated here): [CHINA] Tibet, Gyangtse, Jul-Sep 1904, *H.J. Walton* s.n. (K000898762! isolectotypes E00317867! P04931276! clastotypes G! LE01013342!).

###### Note.

Iljin (1929) indicates two type specimens (“Kew [K] and Vienna Univ. [WU]”) in the protologue. We did not find the material in WU and thus select a lectotype in herbarium K with additional authentic material in other herbaria.

###### Description.

**Figure 33.**
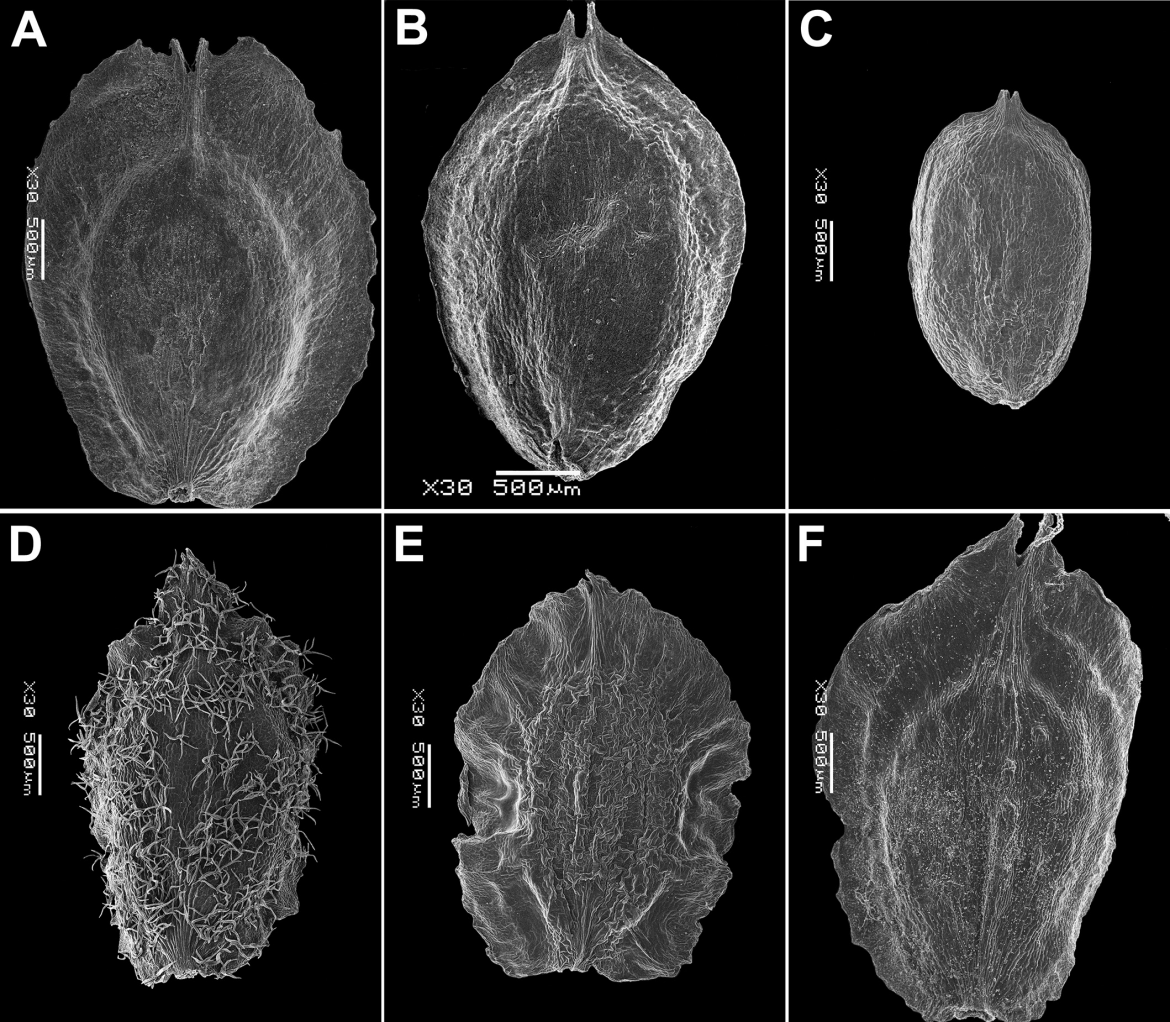
SEM micrographs of *Corispermum* fruits: **A***C.falcatum***B***C.nanum***C***C.pamiricum***D**C.dutreuiliivar.montanum**E**C.dutreuiliivar.dutreuilii**F***C.pseudofalcatum*. Magnification: 30×.

Annual up to 40 cm, slightly pubescent. Leaves lanceolate, oblanceolate or narrowly oblong, often recurved, up to 3.0 × 0.5 cm. Inflorescence elongated, with many flowers. Bracts usually recurved, sickle-shaped, not completely covering the fruit. Fruits 3.5–4.0 × 3.0 mm, glabrous but sometimes with dark-brown cells and scattered warts (Fig. 33A); fruit margin emarginate at the top. Wing 0.45–0.60 mm, denticulate or crisp, narrowly triangular in cross-section. Sclereids in the medium fruit part consist of 1–2 cell layers orientated parallel to the fruit axis.

###### Habitat.

Screes and sandy riverbeds; 3500–4800 m a.s.l.

###### Phenology.

Flowering: July-September; fruiting: August-October.

###### Distribution.

See Fig. 34.

**Figure 34.**
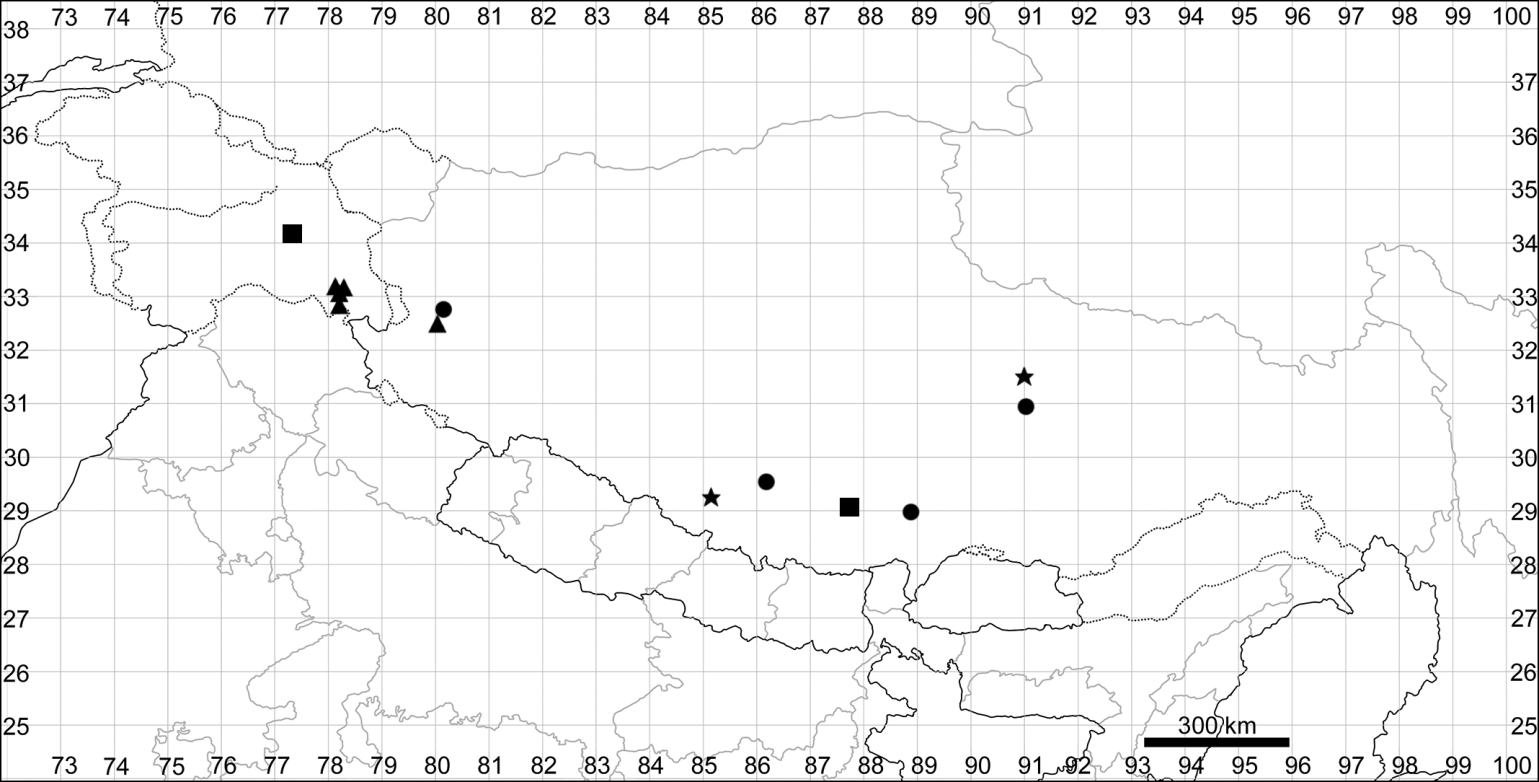
Distribution map of *Corispermumfalcatum* (circles), *C.nanum* (stars), *C.pamiricum* (squares) and C.dutreuiliivar.montanum (triangles).

###### Specimens examined.

**CHINA**: **Xizang: Ngari Prefecture**: Gar (Gaer) County, 4250 m a.s.l., 29 Jul 1976, *Qinghai-Tibet Team 76-8613* (PE00540486); Gar (Gaer) County, nr Shiquanhe river, 4250–4300 m a.s.l., 6 Aug 1976, *Qinghai-Tibet Team Vegetation Group 13429* (PE00540478); **Xigazê Prefecture**: Gyangtse, 28°96'N, 88°8'E, 12 Jul 1953, *P.C. Isoong 5692* (PE00540346); Raka Tsangpo valley, 4760 m a.s.l., 29°30'N, 86°17'E, 2 Sep 2003, *G. & S. Miehe 03-062-10* (E00190600); **Lhasa City**: Namtso lake, 30°52'N, 91°03'E, 4720 m a.s.l., 2 Sep 2005, *Nölling & Hanspach NX05-194-02* (herb. Miehe);

###### General distribution.

Tibet.

##### 2. Corispermum nanum

Taxon classificationPlantaeCaryophyllalesChenopodiacea

Sukhor. & M.Zhang, Phytotaxa 172(2): 84 (2014)

###### Holotype.

CHINA, Xizang, Saga, Nyalam road, 17 Aug 2011, *Yu, Hou & Zhang 5607* (PE2264191!).

###### Description.

Dwarfish, densely pubescent annual up to 6 cm with a single stem or with short lateral branches. Leaves linear, up to 1.5(2.0) mm wide. Inflorescence very short, with several flowers. Bracts slightly shorter than fruits (fruit margins visible). Fruit ovoid, 2.5–2.7 × 2.0 mm, glabrous, apex rounded and only slightly emarginated (Fig. 33B); no visible detachments of the pericarp from the seed coat are present. Wing conspicuous (0.3–0.4 mm), entire, triangular in cross-section. Pericarp in medium fruit part without sclereids or with one layer located parallel to the fruit axis.

###### Habitat.

Sandy places at altitudes of 4000–4700 m a.s.l.

###### Phenology.

Flowering: July-September; fruiting: August-October.

###### Distribution.

See Fig. 34.

###### Specimens examined.

**CHINA: Xizang: Nagqu Prefecture**: Central Plateau, Nagqu to Siling Co (Lake Siling Tso), 31°28'N, 91°0'E, 4580 m a.s.l., lake terraces, 16 Aug 1993, *G. & S. Miehe 9488/03* (MSB147355). The tiny individuals looks similar to the type material, but the fruits are dentate (a typical character for all Tibetan species).

###### General distribution.

Endemic to South Tibet.

##### 3. Corispermum pamiricum

Taxon classificationPlantaeCaryophyllalesChenopodiacea

Iljin, Acta Inst. Bot. Acad. Sc. URSS, ser. 1(3): 165 (1937)

###### Holotype.

[TAJIKISTAN] Buchara prov. [Gorno-Badakhshan Autonomous province], Wachan [Vakhan], in valle fl. Pamir pr. castellum Langar-Gisht, ca. 9500 ft a.s.l., in glareosis riparris [Pamir river valley near Langar-Gisht fortress, 9500 ft a.s.l.], 27 Jul 1901, *Th. Alexeenko 3217* (LE! with a topotype no. *3216*!).

###### Description.

Annual up to 20 cm, usually pubescent. Leaves linear to narrowly oblong, up to 2.0 × 0.5(0.7) cm, with a mucro at the top. Inflorescence short, its branches with 5–15 flowers. Bracts significantly shorter than leaves, ovoid, (almost) completely covering the fruit, sometimes recurved at the fruiting stage. Fruit 2.2–3.0 × 1.7–2.0 mm, 0.40–0.60 mm thick, with entire margin and indistinctly triangular or roundish apex terminating in slightly emarginate beak (Fig. 33C), glabrous and mostly smooth (without detachments), sometimes with groups of dark-brown tanniniferous cells. Wing small, 0.15–0.30 mm, broadly triangular in cross-section. Pericarp without detachments. Sclereids in the medium fruit part composed of 1–2 layers located parallel to the fruit axis.

###### Habitat.

Sandy places at altitudes of 3400–4000 m a.s.l.

###### Phenology.

Flowering: July-September; fruiting: August-October.

###### Distribution.

See Fig. 34.

###### Specimens examined.

**CHINA: Xizang: Xigazê Prefecture**: Lhazê (Lazi) County, Kabei vill., 4000 m a.s.l., 27 Aug 1961, *Zhang 2727* (PE00540415);

**INDIA: Jammu & Kashmir**: Ladakh, Indus valley, Sham (W), Basgo to Nye, 3400–3440 m a.s.l., 7 Aug 2006, *L. Klimeš 6709* (PRA).

###### General distribution.

Tajikistan (Pamir), N Afghanistan, N India and W China (Xizang).

##### 4. Corispermum dutreuilii

Taxon classificationPlantaeCaryophyllalesChenopodiacea

Iljin, Acta Inst. Bot. Acad. Sc. URSS, ser. 1(3): 162 (1937)

###### Lectotype

(Sukhorukov, designated here): [CHINA, Xinjiang, Kashgar Prefecture] Polour, 2582 m a.s.l., 17 Jul 1892, [*J.L*.] *Dutreuil de Rhins* s.n. (LE01013341!).

###### Note.

No herbarium is indicated in the protologue (Iljin 1937). We selected the lectotype using a specimen in LE.

###### Description.

Annual up to 20 cm, glabrescent with ascending branches. Leaves linear to oblong (the lower leaves often spatulate), up to 3.0 × 0.6 cm, often recurved with visible white margins and a mucro at the top. Inflorescence elongated, with many flowers. Bracts leaf-like, lanceolate, significantly exceeding the fruit in length and covering it completely laterally. Fruit 2.8–3.5(4.0) × 2.0–2.5 mm, flat or often folded, acute (triangular) apex with verrucose (warty) surface, without stellate hairs (type variety) or with stellate hairs (var. *montanum*) and clearly visible (0.45–0.70 mm) undulate wing that is narrowly triangular in cross-section. Pericarp detachments up to 100 µm. Sclereids in medium fruit part with 1–2 layers located parallel to the fruit axis, rarely with no sclereids.

###### Distribution.

*Corispermumdutreuilii* seems to be the most common species of the genus in Ladakh and NW Xizang. Both varieties can grow together, e.g. in Ladakh (collection numbers *3465 & 3470* for the type variety and *6265 & 6266* for var.*montanum* (PRA)).

###### Habitat.

Sandy riverbeds, stony deserts; 3500–5000 m a.s.l.

###### Phenology.

Flowering: July-early September; fruiting: August-September.

###### General distribution.

West China (Xizang, Xinjiang, Qinghai), North India, Pakistan, Afghanistan, Tajikistan.

##### Corispermum dutreuilii var. montanum

Taxon classificationPlantaeCaryophyllalesChenopodiacea

Sukhor., Phytotaxa 172(2): 87 (2014)

###### Note.

Fruit warty and additionally covered with stellate hairs (Fig. 33D); fruit thickness 0.6–0.8 mm.

###### Holotype.

CHINA, Tibet, [Ngari pref.], 32°31'N, 80°04'E, Shiquan [Sênggê] river, alt. 3800 m a.s.l., 3 August 1984, *Zhengxi An 1-10092* (XJA!).

###### Taxonomic note.

This variety is close to *C.gelidum* Iljin with the main difference in the fruit length (compare Sukhorukov 2007a, 2007b, Sukhorukov et al. 2014) and both taxa can grow together (PRA!).

###### Distribution.

See Fig. 34.

###### Specimens examined.

**INDIA**: **Jammu & Kashmir**: Ladakh, Rupshu Region, Tso Moriri, Pilung La, 4710–4780 m a.s.l., 20 Aug 1999, *L. Klimeš 588* (PRA); Ladakh, Indus valley, Stot (E) [Stod River valley], Sumdo Gonma to Kiagar La, 4770 m a.s.l., 7 Sep 2003, *L. Klimeš 3460* (PRA); Ladakh, Rupshu Region, Samad Rakchan, Thukje Gompa, 4860–5000 m a.s.l., 7 Sep 2005, *L. Klimeš 6265 & 6266* (PRA); Ladakh, Rupshu Region, Tso Moriri, Lema to Peldo, 4550–4560 m a.s.l., 13 Sep 2005, *L. Klimeš 6306* (PRA).

##### Corispermum dutreuilii

Taxon classificationPlantaeCaryophyllalesChenopodiacea

Iljin var. dutreuilii

###### Note.

Fruit warty but without stellate hairs (Fig. 33E), thin (0.25–0.50 mm) in cross-section.

###### Taxonomic note.

The type variety can probably be synonymised with *C.hilariae* (Iljin 1936) due to (i) glabrous stems and the same general habit and (ii) triangular fruit tip and presence of warts in some fruits (variable character). The subulate fruit apex, reported as a peculiar characteristic for *C.dutreuilii*, seems to vary in many specimens. If the merger of both species is to be confirmed, *C.hilariae* is the priority name. Here, we maintain the specific rank of *C.dutreuilii*, dividing it into two varieties (also, see comments on C.dutreuiliivar.montanum).

###### Distribution.

See Fig. 35.

**Figure 35.**
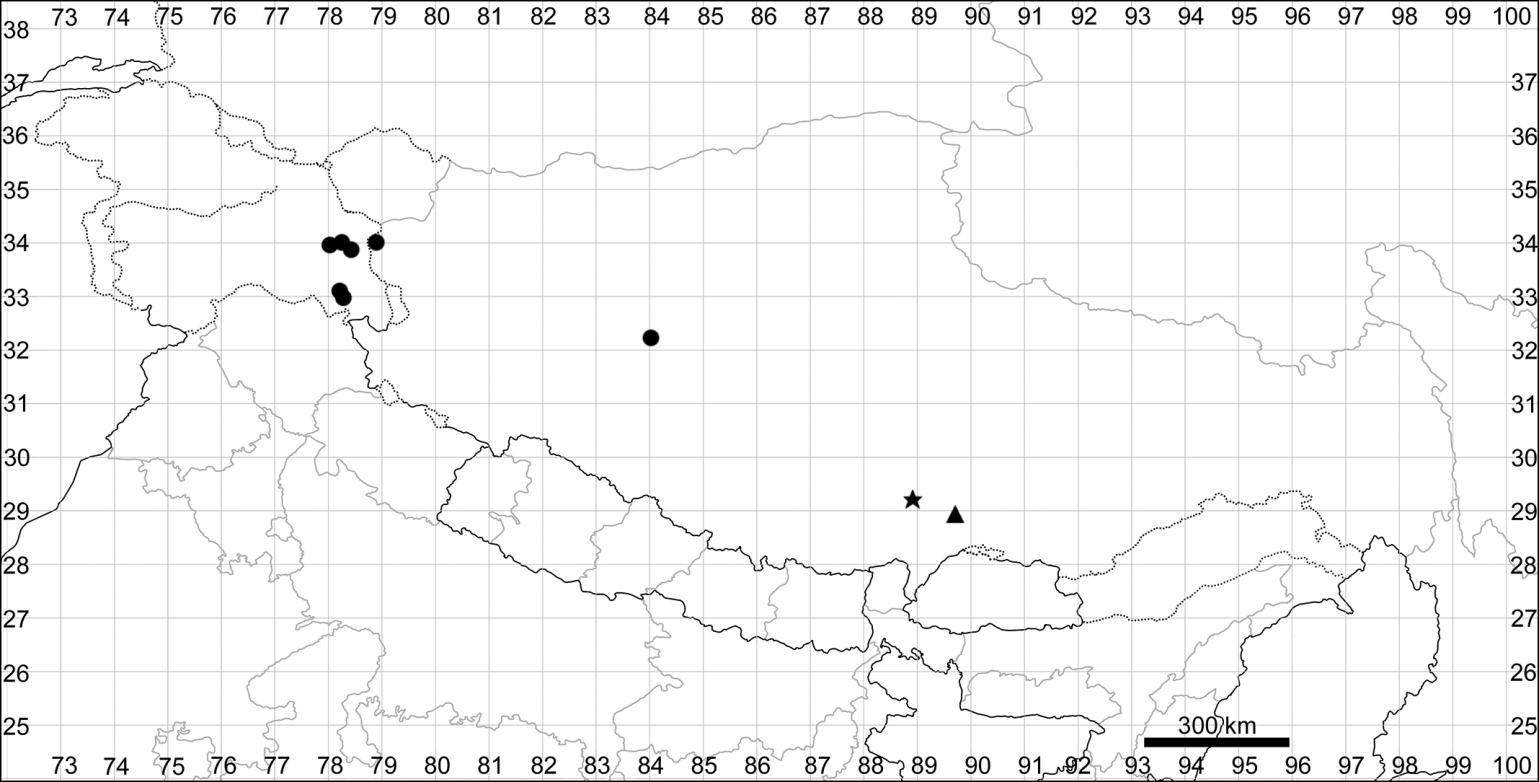
Distribution map of Corispermumdutreuiliivar.dutreuilii (circles), *C.pseudofalcatum* (star) and *C.lhasaense* (triangle).

###### Specimens examined.

**CHINA: Xizang: Ngari Prefecture**: Gêrzê (Gaize) County, 4250 m a.s.l., Aug 1972, *Li 072* (PE00540329); Rutog (Ritu) County, Ni Yagezu, 4500 m a.s.l., 5 Sep 1976, *Qinghai-Tibet Team 76-9164* (PE00540476); Rutog (Ritu) County, NW Lake Bangong (Pangong Tso), Ni Yagezu, 4500 m a.s.l., 5 Sep 1976, *Qinghai-Tibet Team Vegetation Group 13559* (PE);

**INDIA: Jammu & Kashmir**: Ladakh, Shauyk & Rupshu valleys, Oct 1847, *T.T. Thomson* s.n. (K); Ladakh, 6 km W of Pangong Tso [Lake], 4250 m a.s.l., 13 Aug 1997, *H. Hartmann 6083* (MSB137929); Ladakh, Rupshu Region, Tso Moriri, between Korzok & Peldo, 4650 m a.s.l., 11 Aug 2001, *L. Klimeš 1327* (PRA); Ladakh, Pangong Region, Pangong Lake to Chagar Tso, 4300 m a.s.l., 10 Sep 2002, *L. Klimeš 2713* & *2714* (PRA); Ladakh, Indus valley, Stot (E) [Stod River valley], Pilung La, 4860 m a.s.l., 7 Sep 2003, *L. Klimeš 3465 & 3470* (PRA); Ladakh, Rupshu Region, Samad Rakchan, Thukje Gompa, 4860–5000 m a.s.l., 7 Sep 2005, *L. Klimeš 6248* (PRA); Ladakh, Rupshu Region, Samad Rakchan, Thukje vill., 4560 m a.s.l., 9 Sep 2005, *L. Klimeš 6272* (PRA).

##### 5. Corispermum pseudofalcatum

Taxon classificationPlantaeCaryophyllalesChenopodiacea

Tsien & C.G.Ma in Kung & al., Acta Phytotax. Sin. 16(1): 119 (1978)

###### Holotype.

CHINA, Xizang, vicinity of Xigazê (Rikaze), farmland edge, 3800 m a.s.l., [without exact date] 1960, *Fu Guo-Xun 789* (PE00934050!).

###### Description.

Annual to 20 cm, branched at base, (almost) glabrous. Leaves lanceolate, oblanceolate or oblong, 10.0–30.0 × 3.0–5.0 mm, continuously turning into imbricate bracts. Inflorescence elongated. Bracts lanceolate, appressed to the stem or obliquely orientated, not or slightly recurved, completely covering the fruit. Fruit 4.5–5.5 mm long, glabrous but with scattered warts and tanniniferous pigments, apically emarginate (Fig. 33F), thick (0.7–1.0 mm). Wing clearly visible (0.70)0.90–1.40 mm, denticulate, narrowly triangular in cross-section. Pericarp detachments not detected. Sclereids in medium fruit part with 1–2 inner layers (orientated parallel to the fruit axis).

###### Habitat.

Screes and sandy riverbeds; 3000–4200 m a.s.l.

###### Phenology.

Flowering: July-September; fruiting: August-October.

###### Distribution.

This species is known only from the type locality (Fig. 35). Both morphological and carpological characters as well as its record in South Tibet indicate its affinity to *C.lhasaense*.

##### 6. Corispermum lhasaense

Taxon classificationPlantaeCaryophyllalesChenopodiacea

Tsien & C.G.Ma in Kung & al., Acta Phytotax. Sin. 16(1): 119 (1978)

###### Lectotype

(Sukhorukov, designated here): CHINA, Xizang, Lhasa, the Sera Monastery, Liushahe river, in riverway of screes and grits, 3760 m a.s.l., 10 Sep 1960, *Fu Guo-Xun 658* (PE00024043! isolectotypes LE01013344! PE00540403! PE00540404!).

###### Description.

**Figure 36.**
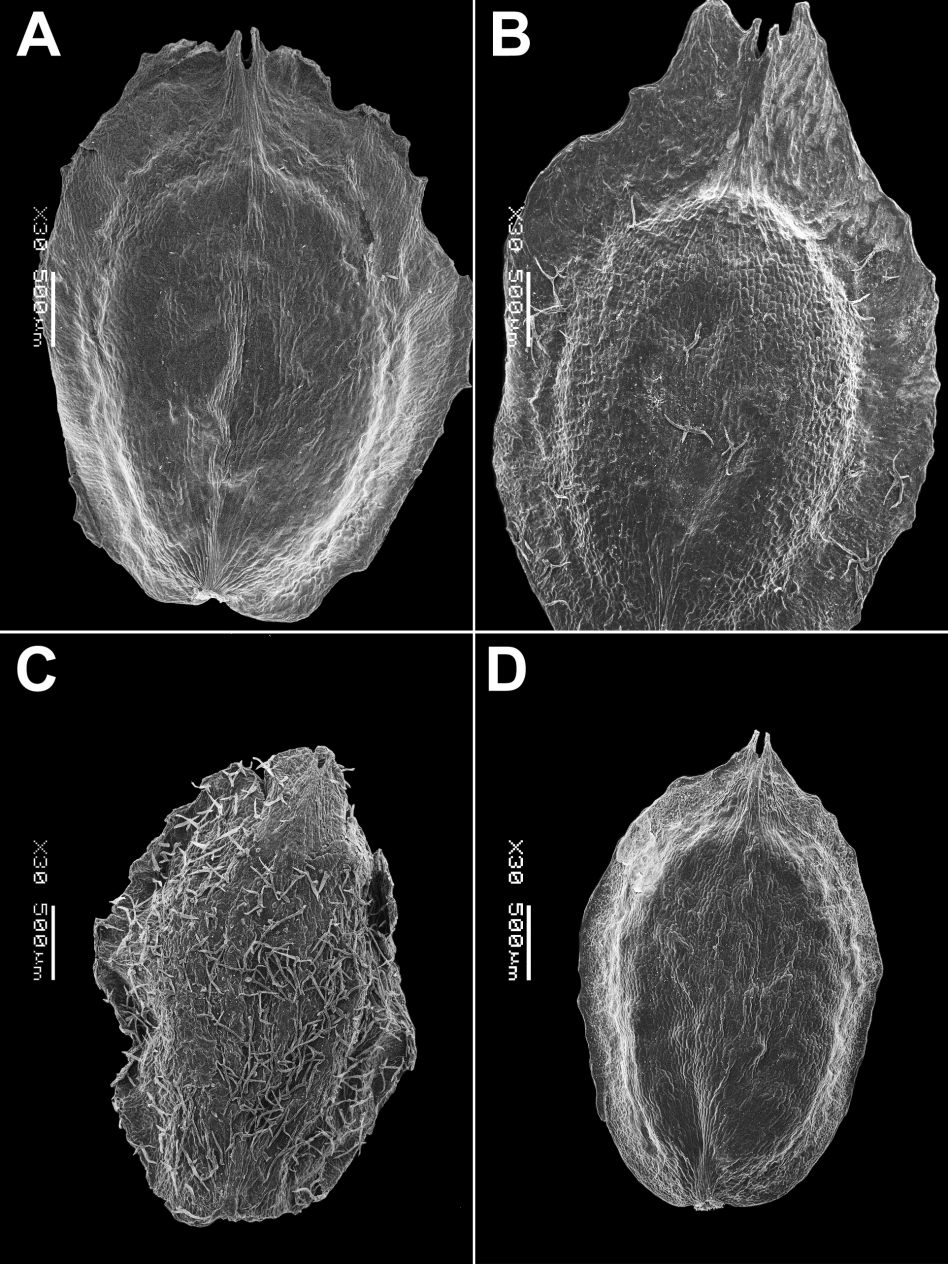
SEM micrographs of *Corispermum* fruits: **A***C.lhasaense***B***C.lepidocarpum***C***C.gelidum***D***C.tibeticum*. Magnification: 30×.

Annual up to 20 cm, glabrescent. Leaves lanceolate, up to 3.0 × 0.3 cm long. Inflorescence dense, 3–5 cm long. Inflorescence elongated. Bracts oblong to ovoid, moderately recurved or sometimes appressed to the stem, completely covering the fruit. Fruit 3.8–4.7 × 3.0–3.5 mm, glabrous (sometimes with dark-brown tanniniferous cells), thick (0.75–0.85 mm) with well-developed wing 0.65–0.90 mm (especially large near the fruit apex; Fig. 36A). Wing irregularly toothed, emarginate, triangular in cross-section. Pericarp without detachments from the seed coat. Sclereids in the medium fruit part consist of 1–3 cell layers orientated parallel to the fruit axis.

###### Habitat.

Screes and sandy riverbeds, 3000–4000 m a.s.l.

###### Phenology.

Flowering: July-September; fruiting: August-October.

###### Distribution.

See Fig. 35.

###### Specimens examined.

**CHINA: Xizang: Xigazê Prefecture**: Gyangtse, Jul/Sep 1904, *H.J. Walton* s.n. (BM).

###### General distribution.

Endemic to South Tibet.

##### 7. Corispermum lepidocarpum

Taxon classificationPlantaeCaryophyllalesChenopodiacea

Grubov, Not. Syst. Herb. Inst. Bot. Ac. Sci. URSS 21: 125 (1961)

###### Holotype.

[CHINA, Xizang] SE Tibet, Temo, Tsangpo valley, 9500 ft a.s.l., 6 Sep 1938, *F. Ludlow*, *G. Sherriff & G. Taylor 6227* (BM000950579! isotype E00317866! clastotypes G! LE!).

###### Description.

Annual up to 20 cm, branched at the base, almost glabrous or glabrescent. Leaves lanceolate or linear, 2.0–4.0 × 0.2–0.4 cm, transforming into imbricate bracts. Inflorescence usually elongated. Bracts lanceolate or linear, often recurved, not completely covering the fruit laterally. Fruits 4.0–5.0 × 3.2–3.5 mm with stellate hairs, pericarp surface often with groups of dark-brown (visually almost black) tanniniferous cells; fruit apex deeply emarginate with two lateral crown-like outgrowths (Fig. 36B). Wing clearly visible (0.85–1.10 mm), irregularly toothed, narrowly triangular in cross-section. Pericarp detachments not detected. Sclereids in medium fruit part with 1–2 outer layers located perpendicular to the fruit axis and 1–3 inner layers (orientated parallel to the fruit axis).

###### Habitat.

Screes and sandy riverbeds; 3000–4000 m a.s.l.

###### Phenology.

Flowering: July-September; fruiting: August-October.

###### Distribution.

See Fig. 37.

**Figure 37.**
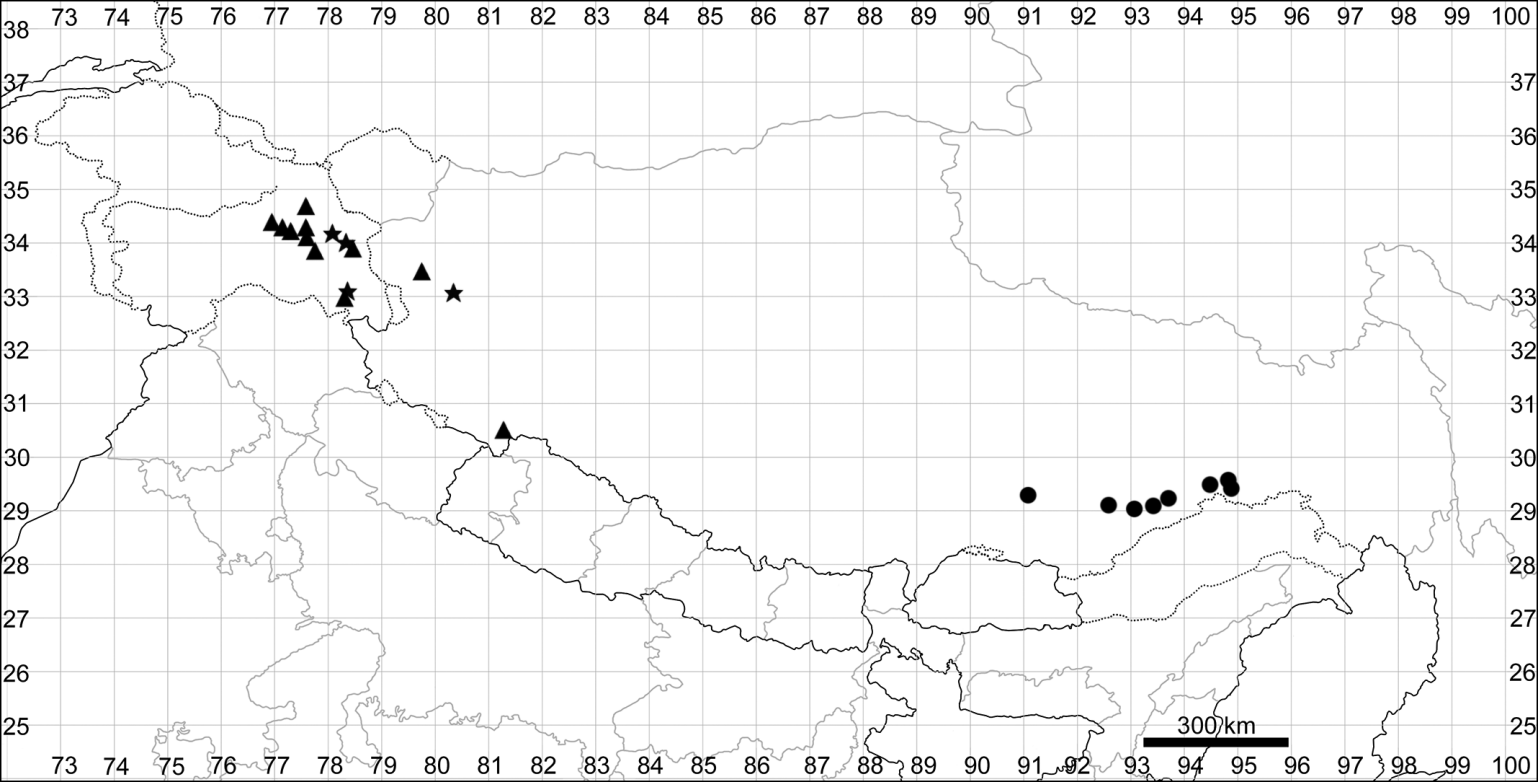
Distribution map of *Corispermumlepidocarpum* (circles), *C.gelidum* (stars) and *C.tibeticum* (triangles).

###### Specimens examined.

**CHINA: Xizang: Shannan Prefecture**: Gyaca (Jiacha) County, 3200 m a.s.l., 25 Aug 1972, *Tibet Chinese Herbal Medicine Survey Team 4393* (PE00540398); Gonggar (Gongga) County, Jêdêxoi (Jiedexiu) town, 31 Aug 1975, *anonym 75-1532* (PE01869900, PE01869902); Way from Gyaca (Jiacha) County to Mainling (Milin) County, 3300 m a.s.l., 18 Sep 1975, *Qinghai-Tibet Team 7953* (PE00540394); **Nyingchi Prefecture**: Mainling (Milin) County, nr [Pai Town] Xuega, 3200 m a.s.l., 30 Aug 1960, *Xia & Zhu 394* (PE00540399); Nang (Lang) County, 3050 m a.s.l., 17 Aug 1972, *Tibet Chinese Herbal Medicine Survey Team 4413* (PE00540396); Mainling (Milin) County, nr Jiage, 23 Sep 1974, *Qinghai-Tibet Team Vegetation Group 3368* (PE00540395); Mainling (Milin) County, Pai distr. (Town), Mt. Doxong (Duoxiong), 2980 m a.s.l., 11 Aug 1975, *Qinghai-Tibet Team Additional Group 751308* (PE00540400, PE00540401).

###### General distribution.

Endemic to South Tibet.

##### 8. Corispermum gelidum

Taxon classificationPlantaeCaryophyllalesChenopodiacea

Iljin, Fl. USSR 6: 875 (1936)

Corispermum gelidum Iljin, Fl. USSR 6: 875 (1936). **Holotype**: [TAJIKISTAN] In ditione lacum Rang-kul, prope vallem Zor-Burljuk [nr Rang-kul Lake, Zor-Burlyuk valley], 29 Aug 1935, *H. Raikova* s.n. (TASH – photo! isotype LE!).
=C.pamiricumIljinvar.pilocarpum Tsien & C.G.Ma in Kung & al., Acta Phytotax. Sin. 16(1): 118 (1978) syn. nov.

###### Lectotype

(Sukhorukov, designated here): CHINA, Xizang, Rutog (Ritu) County, Rabang (Rebang), in lakeside sands, 4360 m a.s.l., 28 Aug 1976, *Qinghai-Tibet Team Vegetation Group 13656-B* (PE00024045! isolectotype PE00640936!).

###### Taxonomic notes.

*Corispermumgelidum* and C.pamiricumvar.pilocarpum are synonymised here for the first time due to the presence of stellate hairs on the fruit surface. Other anatomical characters of the fruit are similar and overlapping (compare with Sukhorukov 2007b, Sukhorukov et al. 2014). However, the authentic specimens of C.pamiricumvar.pilocarpum possess a small (ca. 0.15 mm) fruit wing, whereas the type specimen of *C.gelidum* has a prominent (0.3–0.4 mm) wing.

###### Description.

Annual up to 20 cm, glabrescent. Leaves linear to narrowly oblong, up to 3.0 × 0.5(0.7) cm. Inflorescence short. Bracts shorter than leaves, lanceolate, (almost) completely covering the fruit, appressed to the stem. Fruit 2.6–3.5 × 1.7–2.0 mm, 0.40–0.60 mm thick, flat or folded adaxially, with triangular apex terminating in slightly emarginate beak, with stellate hairs and mostly warty (Fig. 36C), sometimes with groups of dark-brown tanniniferous cells. Wing 0.15–0.45 mm, entire to dentate, broadly triangular in cross-section. Sclereids in the medium fruit part composed of 1–2 layers located parallel to the fruit axis.

###### Habitat.

Sandy places at altitudes of 3500–4800 m.

###### Phenology.

Flowering: July-September; fruiting: August-October.

###### Distribution.

See Fig. 37.

###### Specimens examined.

**CHINA: Xizang**: **Ngari Prefecture**: Rutog (Ritu) County, Rabang (Rebang) Distr., 4300 m a.s.l., 28 Aug 1976, *Qinghai-Tibet Team 76-9128* (PE00540421); Rutog (Ritu) County, Rabang (Rebang) Distr., 4500 m a.s.l., 28 Aug 1976, *Qinghai-Tibet Team Meadow Group 76-12970* (PE00541028); both specimens were labelled as Corispermumpamiricumvar.pilocarpum;

**INDIA**: **Jammu & Kashmir**: Ladakh, Shyok (E), Pangong Lake to Chagar Tso, 4320 m a.s.l., 10 Sep 2002, *L. Klimeš 2712* (PRA); Ladakh, Shyok (E), Takthak, 4100–4110 m a.s.l., 11 Sep 2002, *L. Klimeš 2730* (PRA); Ladakh, Rupshu Region, Tso Moriri, 4550–4560 m a.s.l., Lema to Peldo, 13 Sep 2005, *L. Klimeš 6307* (PRA).

###### General distribution.

Tajikistan (Pamir) and adjacent parts of China, India and Pakistan.

##### 9. Corispermum tibeticum

Taxon classificationPlantaeCaryophyllalesChenopodiacea

Iljin, Izv. Glavn. Bot. Sada SSSR 28: 644 (1929)

Corispermum tibeticum Iljin, Izv. Glavn. Bot. Sada SSSR 28: 644 (1929). **Lectotype** (Sukhorukov, designated here): [probably INDIA, Jammu & Kashmir] Hab. Tibet Occ., alt. 10000–15000 ft a.s.l., [*J.D.]Hooker f. & T.T. Thomson* s.n. (K! lower right plant). **Note**. The Falconer specimens cited in the protologue as the type material of C.tibeticum were labelled by Iljin in 1929 as “an Corispermumorientale?” [“whether C.orientale?”]. In the protologue (Iljin 1929), the specimens were cited as a type material of C.tibeticum, but the Falconer syntype kept at K is not informative and rather deviates from the typical C.tibeticum by its almost wingless fruits. We propose to select a lectotype from other specimens (syntypes) cited by Iljin in the protologue. The lectotype designated here shows the pubescent plant with lanceolate or narrowly oblong leaves, fruits with a blackish body and a small wing. Some other collections cited in the protologue (Shayuk Valley, 3 Oct 1847, *T.T. Thomson*, K; Indus valley, 1 Oct 1847, *Hooker & T.T. Thomson*, K) and mounted as one specimen are C.dutreuilii.
=C.ladakhianum Grey-Wilson & Wadhwa, Kew Bull. 42(2): 471 (1987), syn. nov. **Holotype**. [INDIA] Jammu & Kashmir, Ladakh, Khardung, 3900 m a.s.l., Aug 1976, *Wadhwa 59931* (K! isotype BSD000000038!). **Note**. Grey-Wilson and Wadhwa (1987) compared Corispermumladakhianum with C.lehmannianum; the latter species grows in the plains of West-Central Asia (most common in Kazakhstan, Uzbekistan and Turkmenistan) and Iran. Other taxa were not mentioned in the discussion. Corispermumladakhianum matches C.tibeticum in all characters.

###### Taxonomic notes.

Although *C.tibeticum* looks very similar to *C.hyssopifolium* (and many old specimens were previously identified as such), the fruit anatomy does not confirm the close affinity of both species. As stated earlier, the fruits of *C.hyssopifolium* always have several sclereid layers in different locations (Sukhorukov 2007b, 2014) and the distribution patterns of *C.tibeticum* and *C.hyssopifolium* are very distant (North Himalaya and Eastern Europe, respectively). Some specimens from Ladakh, that were examined, possess elongated fruits appearing similar to those of the *C.declinatum* group. However, the fruit anatomy of *C.tibeticum* drastically differs from that of *C.declinatum* Stephan ex Iljin or *C.tylocarpum* Hance (for more, see Sukhorukov 2007a, 2007b) and it is typical of *C.tibeticum* and relatives (*C.dutreuilii* Iljin, *C.hilariae* Iljin and *C.pamiricum* Iljin). Some forms of *C.tibeticum* with the triangular fruit beak appear similar to *C.dutreuilii*.

The presence of *C.declinatum* and *C.tylocarpum* (syn.: *C.gmelinii* Bunge) in Tibet and North Himalaya (L. Klimeš, in herb.) is not confirmed.

###### Description.

Annual up to 30 cm, moderately pubescent, looking greyish even at maturity and the fruiting stage. Leaves lanceolate, spatulate or narrowly oblong, up to 4.0 × 0.5(0.7) cm with a mucro at the top. Inflorescence elongated, its branches with many (up to 30) flowers. Bracts significantly shorter than leaves, ovoid, (almost) completely covering the fruit. Fruit 2.8–3.2 × 2.5 mm, 0.50–0.65 mm thick, body sometimes blackish with entire margin and broadly triangular apex terminating in slightly emarginate beak, glabrous and mostly smooth (without detachments), sometimes with small warts (Fig. 36D). Marginal wing clearly expressed, ~0.30 mm, triangular in cross-section. Sclereids in the medium fruit part in one layer located parallel to the fruit axis or rarely absent.

###### Habitat.

Sandy riverbeds and stony deserts; 3000–4800 m a.s.l.

###### Phenology.

Flowering: July-September; fruiting: August-early October.

###### Distribution.

See Fig. 37.

###### Specimens examined.

**CHINA: Xizang: Ngari Prefecture**: [Gar County] Pangong Lake, 4430 m a.s.l., 24 Jul 1892, *Pekcz* s.n. (LE); [Burang County] Manasarovar Lake (Mapam Yumtso), south shore of Langa Tso (Lake), 30°30'N, 81°17'E, 4680 m a.s.l., 29 Aug 1993, *G. & S. Miehe 9598/03* (MSB147354);

**INDIA**: **Jammu & Kashmir**: Ladakh, nr Leh, Sep 1848, 10000–15000 ft a.s.l., *T.T. Thomson* s.n. (K); Nubra [valley], Sep 1848, 10000–15000 ft a.s.l., *T.T. Thomson* s.n. (K); [Leh distr.,] Nurla, 10000–11000 ft a.s.l., Jul 1905, *A. Meebold 3957* (G); Ladakh, Saspul, 3320 m a.s.l., 8 Sep 1989, *L. Klimeš & Sintek* s.n. (MSB137930); Ladakh, Rupshu Region, Tso Moriri, 4650 m a.s.l., 11 Aug 2001, *L. Klimeš 1328* (PRA); Ladakh, Pangong Region, Spangmik to Pangong Lake, 4300 m a.s.l., 10 Sep 2002, *L. Klimeš 10 & 13* (PRA); Ladakh, Indus valley, Sham (W), Mebtah, 3700–3750 m a.s.l., 22 Sep 2005, *L. Klimeš 6494* (PRA); Ladakh, Indus valley, Sham (W), Sumdo to Phobe La, 3420–3500 m a.s.l., 24 Sep 2005, *L. Klimeš 6519* (PRA); Ladakh, Indus valley, Sham (W), Nimu, 3180 m a.s.l., 24 Sep 2006, *L. Klimeš 7377* (PRA).

###### General distribution.

N Himalaya, NW Tibet, Karakoram and Pamir.

#### Species excluded

##### Corispermum korovinii

Taxon classificationPlantaeCaryophyllalesChenopodiacea

Iljin, Izv. Glavn. Bot. Sada SSSR 28: 641 (1929)

###### Note.

The species is reported in Klimeš and Dickoré (2005) from Ladakh, India. All examined specimens (PRA) belong to other *Corispermum* species and the distribution area of *C.korovinii* covers the plains of Eastern Kazakhstan and West China (Xinjiang).

##### 13. Agriophyllum

Taxon classificationPlantaeCaryophyllalesChenopodiacea

M.Bieb. ex C.A.Mey., Verz. Pfl. Cauc. Casp. Meer: 163 (1831)

Agriophyllum arenarium M.Bieb. (=Agriophyllumpungens (Vahl) Link ex A.Dietr.) (Type).

###### Description.

**Figure 38.**
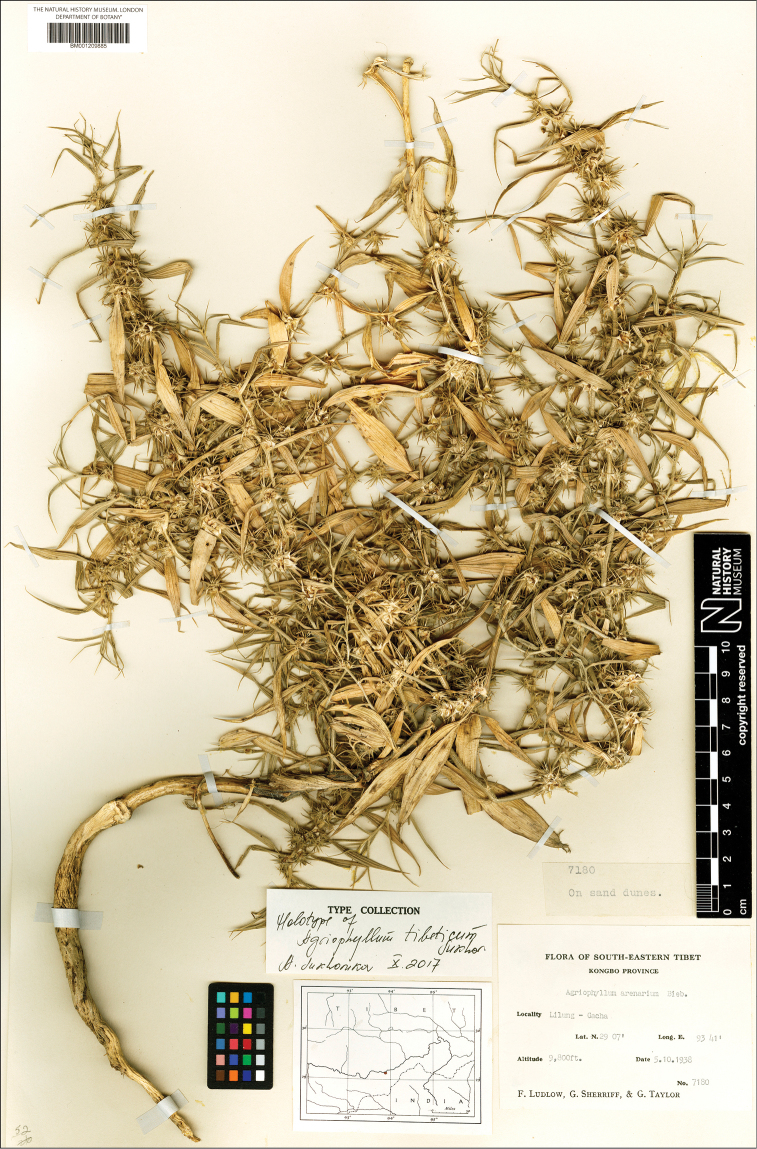
Holotype of *Agriophyllumtibeticum* Sukhor. sp. nov. (BM001209885).

Annuals, usually with tumble-weed habit, covered with dendroid hairs (Fig. 38) and sometimes with simple hairs. Leaves sessile or petiolate with linear to orbicular blades, acuminate, soft at earlier stages then usually turning hard and stout, persistent. Inflorescences short, capituliform; each flower with a stout bract, ebracteolate, mostly bisexual. Perianth of 1–5 free hyaline segments. Stamens 1–5. Fruits glabrous or pubescent with easily caducous dendroid hairs. Pericarp leathery, thin and ruptured in the medium fruit portion. The lower part of stylodia persistent and hardened, often with lateral horn-like outgrowths. Seeds ovoid, lenticular, yellow or brownish, sometimes with dark tanniniferous spots. Embryo vertical, horseshoe-shaped; perisperm copious.

Eight species (including a new species described below) distributed in Irano-Turanian region (7 spp.) and Tibet (1 sp.).

##### 1. Agriophyllum tibeticum

Taxon classificationPlantaeCaryophyllalesChenopodiacea

Sukhor. sp. nov.

urn:lsid:ipni.org:names:77194255-1

Agriophyllum tibeticum Sukhor. sp. nov. **Holotype**: [CHINA, Xizang, Nyingchi Prefecture] SE Tibet, Kongbo [Gongpo’gyamda] County, Lilung-Gacha, 29°07'N, 93°41'E, 9800 ft a.s.l., on sand dunes, 5 Oct 1938, *F. Ludlow, G. Sherriff & G. Taylor 7180* (BM001209885! isotypes – E! G!). Fig. 38.
=Agriophyllumsquarrosum auct. non (L.) Moq.

###### Taxonomic notes.

The specimens of *A.tibeticum* were identified as *A.arenarium* or *A.squarrosum* (both names refer to *A.pungens*) because of their similar habit and oblong leaves. However, the stems of *A.tibeticum* are much shorter and their upper parts are covered with long (up to 2.5 mm), persistent dendroid hairs (or at least not easily caducous) at the fruiting stage. The hairs of *A.pungens* and *A.gobicum* are up to 0.8–1.0 mm long and easily caducous at the fruiting stage. Additionally, these two species are distributed in the plains of Central Asia: *A.pungens* is widely distributed in the Irano-Turanian Region and *A.gobicum* is localised in Mongolia and North China. Thus, there is a significant gap between the distribution of typically alpine *A.tibeticum* and all other species of the genus.

###### Description.

**Figure 39.**
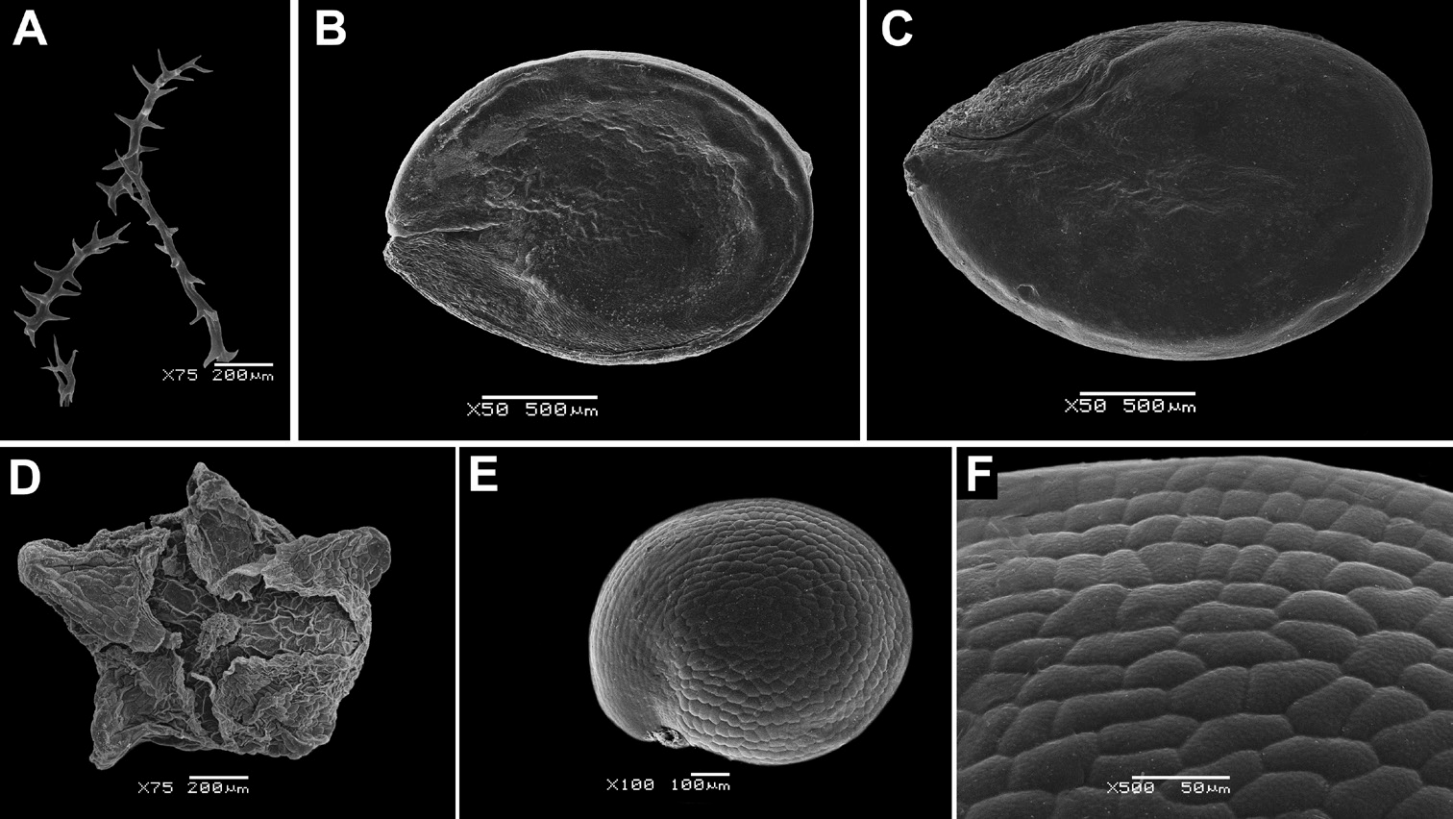
SEM micrographs: **A***Agriophyllumtibeticum*, hairs from the stem **B***A.tibeticum*, concave side of the seed **C***A.tibeticum*, convex side of the seed **D***Suaedaolufsenii*, fruit enclosed in the perianth; **E, F***S.olufsenii*, red seed. Magnification: 50× (**A, B**), 75× (**C, D**), 100× (**E**), 500× (**F**).

Annual up to 20(30) cm with tumble-weed habit; stem very densely pubescent with dendroid hairs (up to 2.5 mm in length, Fig. 39A) and often whitish when young with partially persistent hairs in the fruiting stage. Lower leaves oblong or oblong-lanceolate, continuously decreasing in size and shape in the inflorescence, 2.0–8.0 × 0.5–1.5 cm, tapered in petiole, with (5)6–10 prominent nerves, densely pubescent when young. Inflorescence leafy; flower clusters remote in the lower part of the inflorescence and densely arranged in the upper part. Anthers ~0.3 mm. Pericarp covered with dendroid hairs, glabrescent at fruiting. Seed lens-shaped (Fig. 39B, C), 1.9–2.1 mm × 1.3–1.4 mm, yellow with brownish tanniniferous spots.

Differs from the two related species, *A.pungens* (Vahl) Link ex A.Dietr. and *A.gobicum* Bunge (if the latter is accepted at the specific rank), by persistent dendroid pubescence (with longer hairs) in the inflorescence at the fruiting stage as well as by its disjunct distribution in South Tibet.

###### Habitat.

Sand dunes and stony riverbeds; 2800–3700 m a.s.l.

###### Phenology.

Flowering: August-September; fruiting: September-early October.

###### Distribution.

See Fig. 40.

**Figure 40.**
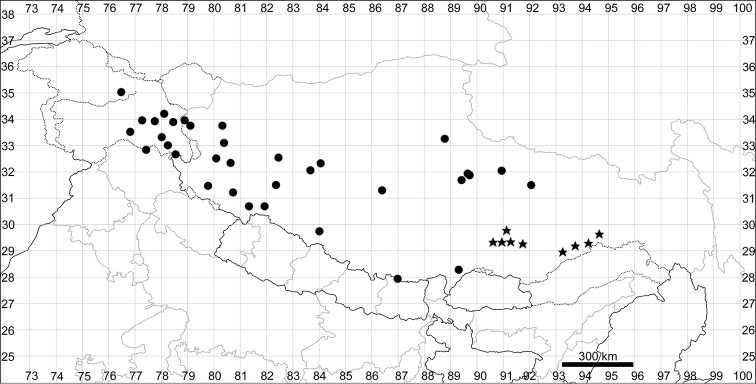
Distribution map of *Agriophyllumtibeticum* (stars) and *Suaedaolufsenii* (circles).

###### Specimens examined.

**CHINA**: **Xizang**: **Lhasa City**: Vicinity of Lhasa, Sep 1939, *Richardson 360* (BM); Lhasa, 5 Sep 1943, *F. Ludlow & G. Sherriff 9921* (BM, E, LE); **Shannan Prefecture**: Gonggar (Gongga) County, river bank of the Yarlung Zangbo (River), 3600 m a.s.l., 23 Sep 1975, *Qinghai-Tibet Team 7659* (KUN0586383); [Nêdong (Naidong) County] Zêtang (Zedang), 3500 m a.s.l., 10 Aug 1977, *B.Z. Guo* et al. *22306* (NAS00303010); [Gonggar (Gongga) County] Yarlung Zangpo at Kyi Chu junction, SW of Lhasa, 29°17'N, 90°41'E, 3550 m a.s.l., 12 Sep 1995, *G. & S. Miehe 95-58-02* (MSB147358); **Nyingchi Prefecture**: Tsangpo valley, 10000–11000 ft a.s.l., 7 Sep 1935, *F. Kingdon-Ward 12313* (BM); Kongbo [Kongpo] County, Temo, Tsangpo valley, 29°35'N, 94°38'E, 9500 ft a.s.l., 8 Sep 1938, *F. Ludlow* et al.. *6238* (BM, E); Mainling (Milin) County, Pai Distr. (town), 11 Aug 1975, *Qinghai-Tibet Team Additional Group 751310* (KUN0586386); Mainling County to Nang County, 1 km W of Gyemdong, 28°59'N, 93°17'E, 3040 m a.s.l., 8 Aug 1994, *B. Dickoré 10696* (MSB144288).

###### General distribution.

Endemic to South Tibet.

#### Species excluded:

##### Agriophyllum pungens

Taxon classificationPlantaeCaryophyllalesChenopodiacea

(Vahl) Link ex A.Dietr., Sp. Pl. 1: 124 (1831)

###### Note.

Reported from Xizang in Zhu et al. (2003), as *A.squarrosum* (L.) Moq. (all specimens seen belong to *A.tibeticum*).

### Subfam. Suaedoideae Ulbr., Nat. Pflanzenfam, 16c: 554 (1934)

#### 14. Suaeda

Taxon classificationPlantaeCaryophyllalesChenopodiacea

Forssk. ex Scop., Intr. Hist. Nat.: 333 (1777) nom. cons.

Suaeda Forssk. ex Scop., Intr. Hist. Nat.: 333 (1777) nom. cons. **Type**: Suedavera Forssk. ex J.F.Gmel.
=Dondia Adans., Fam. Pl. 2: 261 (1763).
=Schanginia C.A.Mey. in Ledebour, Fl. Alt. 1: 394 (1829); **Type**: Schanginialinifolia (Pall.) C.A.Mey. (≡Suaedalinifolia Pall.).
=Schoberia C.A. Mey. in Ledeb., Ic. Pl. Fl. Ross. 1: 11 (1829); **Lectotype** (designated by Schenk and Ferren 2001): Schoberiaacuminata C.A.Mey. (≡Suaedaacuminata (C.A.Mey.) Moq.).
=Alexandra Bunge, Linnaea 17: 120 (1843); **Type**: Alexandralehmannii Bunge (≡Suaedalehmannii (Bunge) Kapralov, Akhani & Roalson).
=Chenopodina Moq. in DC., Prodr. 13(2): 159 (1849); **Type**: not designated.
=Brezia Moq. in DC., Prodr. 13(2): 167 (1849); **Type**: Breziaheterophylla (Kar. & Kir.) Moq. (≡Suaedaheterophylla (Kar. & Kir.) Bunge).
=Belovia Moq. in DC., Prodr. 13(2): 168 (1849); **Type**: Beloviabaccifera Moq. (=Suaedasplendens Gren. & Godr.).
=Borszczowia Bunge, Trudy Imp. S.-Peterburgsk. Bot. Sada 5: 643 (1878); **Type**: Borszczowiaaralocaspica Bunge (≡Suaedaaralocaspica (Bunge) Freitag & Schütze).

##### Description.

Annuals, subshrubs or shrubs, glabrous or slightly pubescent with curved or short straight hairs. Stems green or red. Leaves terete or semi-terete, rarely flattened, obtuse or mucronate, with diverse anatomy and photosynthetic pathways. Inflorescences branched, flowers in clusters subtended by a leaf-bract and tiny hyaline bracteoles. Perianth of 5 segments or lobes, sometimes fused almost to the top, free or adhering to the fruit. Fruit glabrous, without papillae. Seeds monomorphic or (in annual species) dimorphic (structural heterospermy). Embryo horizontal or vertical, sometimes both types are present on the same individual (spatial heterospermy).

More than 100 species distributed in all continents.

#### 1. Suaeda olufsenii

Taxon classificationPlantaeCaryophyllalesChenopodiacea

Paulsen, Vidensk. Meddel. Naturhist. Foren. Kjøbenhavn [s.n.]: 194 (1903)

Fig. 41

Suaeda olufsenii Paulsen, Vidensk. Meddel. Naturhist. Foren. Kjøbenhavn [s.n.]: 194 (1903). **Holotype**. [TAJIKISTAN, Badakhshan] Pamir, in angustiis Chargush [Khargush], in litore salso lacus [on the saline shore of a little lake], 4200 m a.s.l., 2 Sep 1898, *O. Paulsen 1230* (C10009368, image seen! isotype LE n.v.).
≡S.corniculata (C.A.Mey.) Bunge var. *olufsenii*(Paulsen) G.L.Chu in Kung & Tsien, Fl. Reipubl. Pop. Sin. 25(2): 128 (1979).

##### Taxonomic notes.

The examined specimens have perianth with unequal projections at the fruiting stage that is consistent with the species description by Paulsen (1903). However, Lomonosova and Freitag (2008) indicated equal projections of the perianth segments based on the specimens collected in the Altai Mountains. We assume that plants from Altai are different from the populations growing in Pamir, Tibet and North Himalaya.

##### Description.

Annual up to 25 cm, glabrous or young parts are slightly pubescent with curved hairs, branched from the base with prostrate stems. Leaves up to 15.0 × 2.0–4.0 mm, semi-terete without or with an inconspicuous mucro. Inflorescence bracteous. Bracts rather flat, ovoid or elliptical. Flowers 3–12 in clusters. Perianth at fruiting stage with unequal horizontal wing-like projections (Fig. 39D). Stigmas 2. Seeds dimorphic: dark-red seeds 0.9–1.0 mm in diameter, marginally acutish, surface with remarkable polygonal sculpture (Fig. 39E, F), brown seeds 1.1–1.5 mm, with rugose surface.

**Figure 41.**
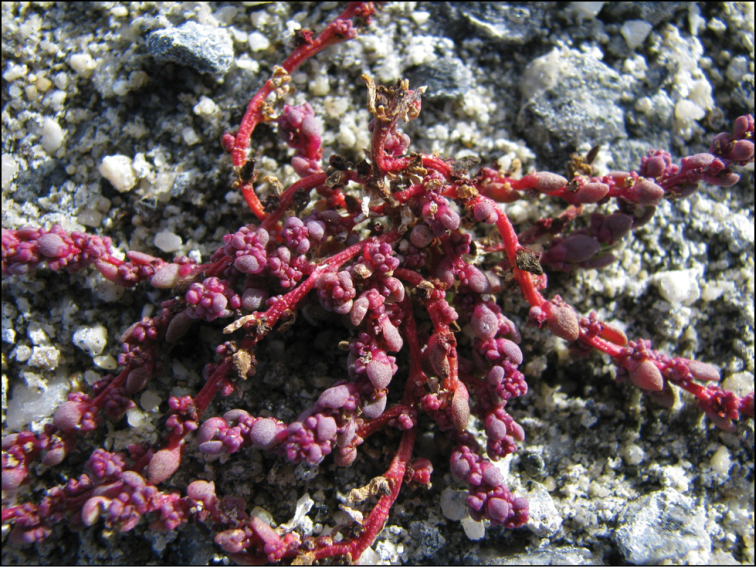
*Suaedaolufsenii* at fruiting stage. Photograph by Prashant Awale (ca. 4300 m a.s.l., near Pangong Lake, Jammu & Kashmir, India, September 2011).

##### Habitat.

Screes, sands and disturbed places; 3400–5300 m a.s.l.

##### Phenology.

Flowering: July-September; fruiting: September-October.

##### Distribution.

See Fig. 40.

##### Specimens examined.

**CHINA: Xizang**: **Ngari Prefecture**: [Gar (Ga’er) County] Shiquanhe, 4300 m a.s.l., 30 Jul 1974, *Biological Institute Tibet Expedition Team 3750* (PE00527157); Burang (Pulan) County, Mapam Yumco (Lake), 4530 m a.s.l., 23 Aug 1974, *Biological Institute Tibet Expedition Team 4153* (PE00527139); Gêrzê (Gaize) County, Datan, 4400 m a.s.l., 8 Sep 1974, *Biological Institute Tibet Expedition Team 4319* (HNWP40537); Gar (Gaer) County, Moincêr (Menshi), 4300 m a.s.l., 10 Jul 1976, *Qinghai-Tibet Team 76-7987* (KUN0587544), *Qinghai-Tibet Team Meadow Group 76-102* (PE00541009); Gar (Gaer) County, Moincêr (Menshi), 4400 m a.s.l., 10 Jul 1976, *Qinghai-Tibet Team Vegetation Group 13223* (PE00527117); Zanda (Zhada) County, 4400 m a.s.l., 19 Jul 1976, *Qinghai-Tibet Team Vegetation Group 12696* (PE00527119); Rutog (Ritu) County, Domar (Duoma) Distr., 5200 m a.s.l., 15 Aug 1976, *Qinghai-Tibet Team 76-9078* (PE00527154); Gê’gyai (Geji) County, Yagra (Yare) Distr., 4780 m a.s.l., 20 Aug 1976, *Qinghai-Tibet Team 76-8686* (KUN0587535), *Qinghai-Tibet Team Vegetation Group 13516* (PE00527118); Gê’gyai (Geji) County, Yanhu Distr., 4350 m a.s.l., 24 Aug 1976, *Qinghai-Tibet Team Meadow Group 13528* (PE00527125); Rutog (Ritu) County, Rabang (Rebang), 4360 m a.s.l., 28 Aug 1976, *Qinghai-Tibet Team Vegetation Group 13658* (PE00527120); Rutog (Ritu) County, nr Banmozhang, 4250 m a.s.l., 4 Sep 1976, *Qinghai-Tibet Team 76-8753* (PE00527134, KUN0587541); Rutog (Ritu) County, Ni Yagezu, 4500 m a.s.l., 5 Sep 1976, *Qinghai-Tibet Team 76-9147* (PE00527155, KUN0587547); Gêrzê (Gaize) County, Marmê (Mami) Distr., 4500 m a.s.l., 8 Sep 1976, *Gansu Agriculture University 158* (PE00541006); Gêrzê (Gaize) County, Marmê (Mami) Distr., 4500 m a.s.l., 8 Sep 1976, *Qinghai-Tibet Team 10422* (PE00527151); Gêrzê (Gaize) County, Kangtog (Kangtuo), 4570 m a.s.l., 10 Sep 1976, *Qinghai-Tibet Team 10208* (PE00527153, KUN0588258); Gê’gyai (Geji) County to Shiquanhe, 4400 m a.s.l., 21 Aug 1990, *Y. Fei* et al. *429* (KUN0275068); Burang (Pulan) County, E of Hor, 4787 m a.s.l., 30°41'48.48"N, 81°58'4.93"E, 3 Sep 2012, *FLPH Tibet Expedition 12-0064* (PE01967962); **Nagqu Prefecture**: Baingoin (Ban’ge) County, Bangkog Co (Ban’ge Lake), 4400 m a.s.l., 16 Jul 1961, *Wang 3555* (PE00527127); Nagqu County, 4490 m a.s.l., 16 Aug 1961, *Wang 3763* (PE00527128); Shuanghu County, 4800 m a.s.l., 18 Jul 1976, *Qinghai-Tibet Team 10197* (PE); Shuanghu County, 4850 m a.s.l., 21 Jul 1976, *Qinghai-Tibet Team 9767* (PE00527147, KUN0587537); Shuanghu County, Laiduo, 4850 m a.s.l., 22 Jul 1976, *Qinghai-Tibet Team 10200* (PE00527140); N Xizang, Jialin, 5100 m a.s.l., 24 Jul 1976, *Gansu Agriculture University 092* (PE00541005); Shuanghu County, 4800 m a.s.l., 26 Jul 1976, *Qinghai-Tibet Team Vegetation Group 12039* (PE); Baingoin (Ban’ge) County, 20 km NE of Bangkog Co (Ban’ge Lake), 4700 m a.s.l., 29 Jul 1976, *Qinghai-Tibet Team 10680* (PE00527123); Xiketang Lake, 32°01'N, 90°57'E, 4560 m a.s.l., 28 Jul 2005, *Nölling & Hanspach NX05-017-04* (herb. Miehe); **Xigazê Prefecture**: [Gyangste County] Kala [Tso], 14000 ft a.s.l., 8 Aug 1936, *Chapman 500* (K000899752); Qomolangma (Mount Everest), 28 Jul 1960, *Fu 508* (PE00527133); Zhongba County, Gaqoi (Gangjiu) distr., 4600 m a.s.l., 29 Jul 1975, *Qinghai-Tibet Team Vegetation Group 7420* (PE00527124);

**INDIA: Jammu & Kashmir**: [nr Parang river] valley below Haule, 17 Sep 1847, *T.T. Thomson* s.n. (K); Ladakh, Tso Kar, Rupshu, 4500 m a.s.l., 5 Sep 1970, *U.C. Bhattacharyya 41013* (BSD); Ladakh, Shyok, 4200 m a.s.l., 30 Aug 1975, *M.V. Viswanathan 55058* (BSD); Ladakh, Muklip, 3975 m a.s.l., 29 Aug 1976, *B.M. Wadhwa 60151* (BSD); Ladakh, Karu, 3400 m a.s.l., 12 Aug 1980, *U.C. Bhattacharyya 71610* (BSD); Ladakh, Chusul valley, Aug 1982, *P.K. Hajra 74219* (BSD); Tso Kar, Rupshu, 4600 m a.s.l., 22 Aug 1995, *H. Hartmann 5035* (MSB160553); Pangong Tso, 4120 m a.s.l., 13 Aug 1997, *H. Hartmann 6081* (MSB160552); Ladakh, Zanskar Region, Markha Hankar vill., 4500 m a.s.l., 21 Aug 1998, *L. Klimeš 106* (PRA); Ladakh, Indus valley, Stot (E) [Stod River valley], 4180 m a.s.l., 16 Aug 1999, *L. Klimeš 540* (PRA); Ladakh, Indus valley, Stot (E), 4850–4940 m a.s.l., 2 Aug 2001, *L. Klimeš 01-5-12* (PRA); Ladakh, Rupshu Region, Tso Moriri, between Karzok & Peldo, 4650 m a.s.l., 11 Aug 2001, *L. Klimeš 1320* (PRA); Ladakh, Rupshu Region, Chumur, 4660–4860 m a.s.l., 21 Aug 2001, *L. Klimeš 1382* (PRA); Ladakh, Shyok (W & C), 3710–3780 m a.s.l., 15 Sep 2001, *L. Klimeš 1845* (PRA); Ladakh, Pangong Region, Spangmik to Pangong Lake, 4300 m a.s.l., 10 Sep 2002, *L. Klimeš 2704* (PRA); **Himachal Pradesh**: Lahaul, Kenlung, 15000 ft a.s.l., 19 Jul 1941, *N.L. Bor 15182* (DD).

##### General distribution.

W China, N India, N Pakistan and Tajikistan; also reported from E Kazakhstan, Kyrghyzstan, Mongolia and Russia (Altai).

#### Species excluded

##### Suaeda microsperma

Taxon classificationPlantaeCaryophyllalesChenopodiacea

Fenzl in Ledeb., Fl. Ross. 3(2): 785 (1851)

###### Note.

Cited in Dhar and Kachroo (1983), Chowdhery (1984) and Paul (2012) for Indian Himalaya.

The data on the presence of this species in India is erroneous since it is distributed in the plains of Central Asia (Kazakhstan and Uzbekistan to the foothills of Tajikistan in the south). All *Suaeda* specimens from North India belong to *S.olufsenii*.

### Subfam. Camphorosmoideae A.J.Scott, Fedd. Repert. 89(2–3): 102 (1978)

#### 15. Bassia

Taxon classificationPlantaeCaryophyllalesChenopodiacea

All., Mélanges Philos. Math. Soc. Roy. Turin 3: 177 (1766)

Bassia aegyptiaca All. (=B.muricata (L.) Asch.) (**Type**).

##### Description.

Annuals or subshrubs with simple, alternate, entire, flattened or terete leaves with diverse types of Kranz anatomy. Inflorescence spiciform, leafy, formed of few-flowered axillary clusters. Flowers solitary or in clusters of 2–5, hermaphrodite or unisexual (mostly female) with 5 perianth segments that are free or half-way connate and which develop wing-like, spiny or tuberculate outgrowths at the fruiting stage or rarely lack outgrowths. Style very short with 2–3 filiform stigmas. Fruit round or ovoid, compressed with smooth pericarp ± tightly adjoining the seed coat; seeds with horizontal horseshoe-shaped embryo; perisperm abundant.

##### Note.

The genera that are now included in *Bassia* are cited in Kadereit and Freitag (2011).

~20 species in Eurasia and North Africa.

#### Key to the species

**Table:** 

1	Plants subshrubs or sub-shrublets; leaves semi-terete; bracts equal to or slightly larger than flower clusters	**3. *B.prostrata***
–	Plants annuals; leaves flattened; bracts clearly longer than the flower clusters	**2**
2	Plants whitish or grey due to dense pubescence on stem, leaves and perianth	**2. *B.odontoptera***
–	Plants green or reddish-green, pubescent or almost glabrous; perianth glabrous or ciliate	**1. *B.scoparia***

##### 1. Bassia scoparia

Taxon classificationPlantaeCaryophyllalesChenopodiacea

(L.) A.J.Scott, Fedd. Repert. 89(2–3): 108 (1978)

≡Chenopodiumscoparium L., Sp. Pl.: 221 (1753). **Lectotype** (designated by Jafri and Rateeb 1978): Herb. Linn. 313.20 (LINN! image available at http://linnean-online.org/3145/).
≡Kochiascoparia (L.) Schrad., Neues J. Bot. 3(3–4): 85 (1809). Comprehensive list of synonymic names of B.scoparia is presented in Sukhorukov (2014). Here we add an additional synonym.
=Bassiafiedleri Aellen in Hegi, Ill. Fl. Mitt.-Eur., ed. 2, 3(2): 713 (1961) syn. nov. Aellen (in Hegi 1961) stated a new name instead of Bassiadivaricata (Kar. & Kir.) Kuntze (1891) [now Gruboviadasyphylla (Fisch. & C.A.Mey.) Freitag & Kadereit], non Bassiadivaricata F. Muell. (1882). The name Bassiafiedleri was accepted as a synonym of Gruboviadasyphylla (Kadereit and Freitag 2011). However, the analysis of the material in G collected by O. Fiedler in Germany (as an alien plant) and treated by P. Aellen clearly shows that Bassiafiedleri is conspecific with Bassiascoparia.

###### Description.

Erect annual up to 150(200) cm; stem and branches green or sometimes reddish, moderately to densely covered by long, soft, simple multicellular hairs. Leaves flat, 20.0–50.0(80.0) × 1.5–7.0(10.0) mm, subpetiolate at base, narrowly oblong to lanceolate or linear, three-nerved, densely pilose. Bracts longer than flower clusters. Inflorescences spiciform, foliose, axes with fine spreading hairs. Flowers mostly in clusters of 2–5, unisexual or hermaphrodite, surrounded by basal tufts of hairs or not. Perianth fused halfway, ciliate or glabrous, with small horizontal wings or tubercles at the fruiting stage, sometimes without any projections. Fruit compressedly ovoid, ~2.0 mm long, dark brown. Seeds with horizontal embryo.

###### Habitat.

Disturbed areas at elevations of 2500–3500 m, stony slopes or sands; rare.

###### Phenology.

Flowering: July-September; fruiting: August-October.

###### Distribution.

See Fig. 42.

**Figure 42.**
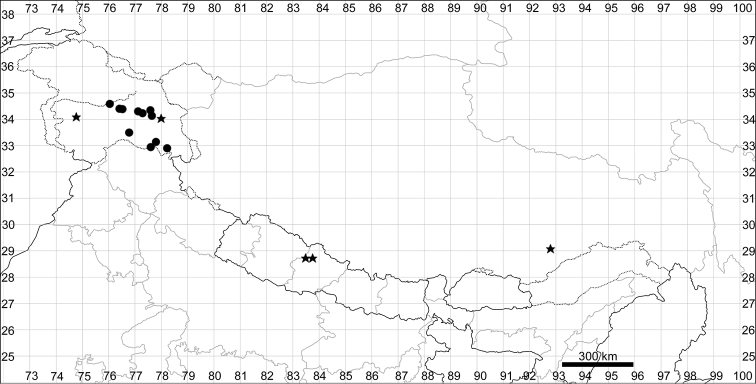
Distribution map of *Bassiascoparia* (stars) and *B.odontoptera* (circles).

###### Specimens examined.

**CHINA**: **Xizang**: **Nyingchi Prefecture**: Nang (Lang) County, Zhongda, 3150 m a.s.l., cultivated, 3 Sep 1972, *Tibet Chinese Herbal Medicine Survey Team 4520* (PE00526535);

**INDIA: Jammu & Kashmir**: Srinagar, 1856, *Schlagintweit* s.n. (BM); Ladakh, Kargil, 2800 m a.s.l., 4 Sep 1975, *M.V. Viswanathan 55634* (BSD); Ladakh, 3450 m a.s.l., 30 Jul 1979, *H. Hartmann 2739* (G, MSB160571) [var. *trichophylla*; probably escaped from cultivation]; Ladakh, Suru region, Kargil, 2670–2690 m a.s.l., 18 Sep 2004, *L. Klimeš 4967* (PRA).

**NEPAL**: **Western**: **Dhaulagiri Zone**: [Mustang Distr.] Tukuche, Kali Gandaki river, 8500 ft a.s.l., 12 Jun 1954, *J. Stainton, W.R. Sykes & L.H.J. Williams 1072* (BM); Mustang [Distr.], Jomosom vill., 2800 m a.s.l., 26 Sep 2009, *A. Sukhorukov* s.n. (E, MW).

###### General distribution.

Most likely, originated in Central Asia (Kazakhstan to West China and Mongolia), alien elsewhere in the temperate parts of Eurasia, Australia and Americas.

##### 2. Bassia odontoptera

Taxon classificationPlantaeCaryophyllalesChenopodiacea

(Schrenk) Freitag & G.Kadereit, Taxon 60(1): 73 (2011)

≡Kochiaodontoptera Schrenk, Bull. Acad. Petersb. 1: 361 (1843). **Lectotype** (Sukhorukov, designated here): [probably East KAZAKHSTAN] “desert. Songor.” [Songorian desert] leg. *Schrenk* s.n. (P04994622!).
=Kochiairanica Bornm., Bull. Herb. Boissier, ser. 2, 8: 546 (1908). **Syntypes**: Persia [IRAN], prov. Kerman, Kerman, in cultis, 1900 m a.s.l., 20 Aug 1892, *J. Bornmüller 5074* (P032634! LE! MW!) as Salsolairanica.
≡Bassiairanica (Bornm.) Bornm., Fedd. Repert. 17: 276 (1921).

###### Description.

Annual up to 25 cm with often reddish stems covered with spreading hairs. Leaves up to 1.0 cm, oblong to ovate, densely covered with (semi)appressed hairs (both stem and leaves are partially glabrescent at fruiting). Inflorescences bracteose with bracts slightly longer than the flower clusters. Flowers (2–5) in clusters. Perianth fused up to the half, densely pubescent with wing-like projections at fruiting, 3.0–5.0 mm across (with wings). Wings white or reddish, entire to erose-dentate or lobate (but not pinnatisect). Fruit compressedly ovoid, 0.7–0.8 mm long. Seeds with horizontal embryo.

###### Habitat.

Sands and stony plateaux at elevations of 2700–4700 m; common in many places of Jammu and Kashmir as indicated by the collectors.

###### Phenology.

Flowering: July-September; fruiting: August-early October.

###### Distribution.

See Fig. 42.

###### Specimens examined.

**INDIA**: **Jammu & Kashmir**: Zanskar, Kargil, 13500 ft a.s.l., Jul 1933, *W. Koelz 5580* (H1038426); Ladakh, Namika La, 28–29 Jul 1933, *W. Koelz 6189* (G); Ladakh, Saspul, 10500 ft a.s.l., 27 Aug 1931, *W. Koelz 2705* (E, K); Kargil [Distr.], 12000 ft a.s.l., 29 Jul 1937, *Murt 83* (E); Ladakh, Bragnag, 13000 ft a.s.l., 31 Aug 1931, *W. Koelz 2779* (E, LE); Ladakh, Whisky Nullah, 30 Aug 1970, 4000 m a.s.l., *U.C. Bhattacharyya 40941* (BSD); Ladakh, Mulbekh, 22 Jul 1976, *B.M. Wadhwa 58917* (BSD); Ladakh, between Leh & Saspul, 1979, *H. Hartmann 2741* (MSB137916); Ladakh, Tungri, 3650 m a.s.l., 12 Aug 1979, *H. Hartmann 2740* (MSB137915); Ladakh, Kardung La, 3800 m a.s.l., 9 Aug 1980, *U.C. Bhattacharyya 71535* (BSD); Ladakh, Sabu, 3400 m a.s.l., 26 Jul 1987, *H. Hartmann 3062* (MSB137917); Ladakh, Zanskar Region, Sarchu, 4200 m a.s.l., 8 Aug 2000, *L. Klimeš 1126* (PRA); Ladakh, Leh, Ganglas to Pulu, 22 Sep 2001, *L. Klimeš 1995* (PRA); Ladakh, Dras Region, 2820–2800 m a.s.l., Kharboo [Kharbu], 20 Aug 2005, *L. Klimeš 5900* (PRA); Ladakh, Indus valley, Sham, 3050 m a.s.l., 30 Aug 2005, *L. Klimeš 6166* (PRA); Ladakh, Rupshu Region, Tso Moriri, 4550 m a.s.l., 13 Sep 2005, *L. Klimeš 6316* (PRA); Ladakh, Indus valley, Sham (W), 3210–3330 m a.s.l., 23 Sep 2006, *L. Klimeš 7369* (PRA).

###### General distribution.

Iran, Afghanistan, North Pakistan and Central Asia (Turkmenistan and Tajikistan).

##### 3. Bassia prostrata

Taxon classificationPlantaeCaryophyllalesChenopodiacea

(L.) A.J.Scott, Fedd. Repert. 89(2–3): 108 (1978)

≡Salsolaprostrata L., Sp. Pl.: 222 (1753). **Lectotype** (Tan 1997): Herb. Linn. No. 315.15 (LINN).
≡Kochiaprostrata (L.) Schrad., Neues J. Bot. 3(3–4): 85 (1809). Other synonyms are listed in Sukhorukov (2014).

###### Description.

Subshrub or sub-shrublet up to 100 cm (much smaller in mountainous areas) with basally branched caudex. Leaves up to 25 mm long, linear or lanceolate, densely pubescent. Bracts reduced, mostly up to 5 mm long, equal to or slightly longer than flower clusters. Perianth densely pubescent, fused up to the half, with wing-like projections of white or reddish colour at fruiting stage turning brown before dissemination, 3.5–6.0 mm across (including wings). Seeds with horizontal embryo.

###### Habitat.

Subalpine vegetation (often in *Artemisia* steppes) and screes at altitudes of 3000–4500 m a.s.l.; locally common.

###### Phenology.

Flowering: July-September; fruiting: August-October.

###### Distribution.

See Fig. 43.

**Figure 43.**
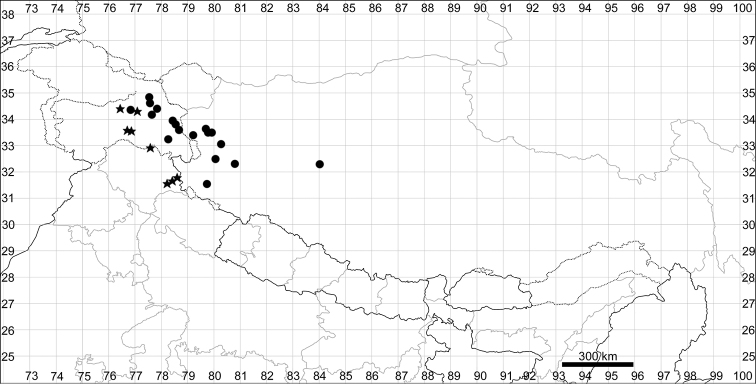
Distribution map of *Bassiaprostrata* (stars) and *Gruboviadasyphylla* (circles).

###### Specimens examined.

**INDIA**: **Jammu & Kashmir**: Ladakh, above Namika La, 3800 m a.s.l., 21 Jul 1976, *H. Hartmann 2128* (G152725, MSB160565); Ladakh, above Tungri, 3760 m a.s.l., 15 Aug 1979, *H. Hartmann 2742* (G250780, MSB160566); [Kargil Distr.] Zanskar, Skagam, 11500 ft a.s.l., 22 Jul 1981, *Grace* et al. *(Southampton Bot. Exped.) 134* (BM, K); Ladakh, between Likir & Nurla, 3700 m a.s.l., 19 Jul 1987, *H. Hartmann 3061* (MSB160568); Ladakh, Zanskar, Zara, 3 Sep 1998, *L. Klimeš 296* (PRA); **Himachal Pradesh**: Kinnaur Distr., above Leppa [Lippa], 8000–12000 ft a.s.l., Aug 1847, *T.T. Thomson* s.n. (K); [Kinnaur Distr.] Jangi to Pangi, 9000 ft a.s.l., Sep 1891, *J.H*. *Lace 1151* (E); Ladakh, Zanskar, Sarchu, 8 Aug 2000, *L. Klimeš 1127* (PRA); Kanam, 5 Sep 1963, *N.C. Nair 30551* (BSD); [Kinnaur Distr.] Pooh, 7 Jun 1972, *K.P. Janardhanan 47784* (BSD); Kinnaur Distr., Sutlej valley, 6 km W of Puh [Pooh], 31 Aug 2008, *H. Freitag 34008* (MJG009668).

###### General distribution.

Temperate Eurasia.

#### Excluded species

##### Bassia indica

Taxon classificationPlantaeCaryophyllalesChenopodiacea

(Wight) A.J.Scott, Fedd. Repert. 89(2–3): 89 (1978)

≡Kochiaindica Wight, Icon. PL. Ind. Orient. 5: tab. 1791 (1852). **Lectotype** (Sukhorukov, designated here): [INDIA, Tamil Nadu state] Coimbatore, Oct 1847, herb. *Wight 2479* (K000400258!).

###### Notes.

This species was reported for Jammu & Kashmir and Himachal Pradesh, India (Dhar and Kachroo 1983, Chowdhery 1984), but all examined specimens belong to *Bassiascoparia* and other Chenopodiaceae. However, the presence of *B.indica* is possible in the foothills of North Himalaya (0–1500 m a.s.l.), as it is known from neighbouring areas, e.g. state of Haryana, India (BSD! LE!) and Punjab Province, Pakistan (K!).

##### 16. Grubovia

Taxon classificationPlantaeCaryophyllalesChenopodiacea

Freitag & G. Kadereit, Taxon 60(1): 72 (2011)

Grubovia dasyphylla (Fisch. & C.A.Mey.) Freitag & G.Kadereit (**Type**).

###### Description.

Annuals, usually with tumble-weed habit. Stems and leaves with white, simple multicellular hairs that are appressed and obliquely orientated and with scattered yellow short hairs mostly located in the leaf axils. Leaves terete, green, obtuse, with water-storage tissue and C_3_-type anatomy. Flowers in leaf axils, solitary or in pairs. Perianth of 5 at least half-way concrescent segments, usually pilose; all or three of five segments develop wing-like or spiny projections. Stamens 5, with small anthers. Fruit with hyaline pericarp adherent to the seed coat. Seed embryo horizontal. Perisperm abundant.

Here, we indicate for the first time that the presence of short yellow hairs is the remarkable morphological characteristic of *Grubovia* that is important for the delimitation of this genus. *Grubovia* comprises three species mostly distributed in Central Asia. *Gruboviadasyphylla* has the largest distribution that also includes Tibet and North Himalaya.

##### 1. Grubovia dasyphylla

Taxon classificationPlantaeCaryophyllalesChenopodiacea

(Fisch. & C.A.Mey.) Freitag & G.Kadereit, Taxon 60(1): 72 (2011)

Fig. 44

≡Kochiadasyphylla Fisch. & C.A.Mey. in Schrenk, Enum. Pl. Nov. Songar.: 12 (1841). **Lectotype**: not selected (LE).
≡Echinopsilondasyphyllum (Fisch. & C.A.Mey.) Moq. in DC., Prodr. 13(2): 136 (1849);
≡Bassiadasyphylla (Fisch. & C.A.Mey.) Kuntze, Rev. Gen. Pl. 2: 546 (1891);
=Echinopsilondivaricatum Kar. & Kir., Bull. Soc. Nat. Moscou [14]: 736 (1841). **Lectotype** (designated by Iljin ex Gubanov et al. 1998): [KAZAKHSTAN] In argilloso-salsis ad littoral meridionalia lacus Noor-Saissan, [year] 1840, *Karelin & Kiriloff 983* (LE!), isolectotypes K000898797! K000898798! MW!
≡Chenoleadivaricata (Kar. & Kir.) Hook.f., Fl. Brit. India 5(13): 10 (1890);
≡Bassiadivaricata (Kar. & Kir.) Kuntze, Revis. Gen. Pl. 2: 546 (1891) nom. illegit., non F.Muell. 1882.

###### Taxonomic note.

Both *Kochiadasyphylla* and *Echinopsilondivaricatus* were described in the same year (1841). The priority of the name *Kochiadasyphylla* (now *Gruboviadasyphylla*) is based on the publication date of the book by Fischer and Meyer (22 July 1841) against the name *Echinopsilondivaricatum* published by Karelin and Kiriloff in the *Bulletin de la Société Imperiale des Naturalistes de Moscou*, vol. 4 (8 September 1841).

###### Description.

**Figure 44.**
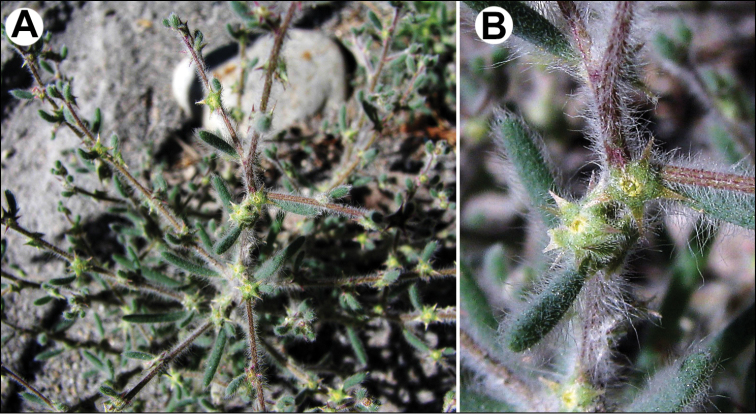
*Gruboviadasyphylla*: **A** general view of the plant at blooming stage **B** close-up of the flowers. Photographs by Prashant Awale (near Khalsar village, Nubra valley, Jammu & Kashmir, India, September 2011).

Annuals up to 30 cm with tumble-weed habit. Stems red or green, pilose at younger stage and moderately glabrescent at fruiting (white or greyish hairs often turning fulvous after drying). Leaves 1.0–4.0 × 0.1–0.3 cm, with appressed or spreading white hairs up to 6–7 mm long. Perianth segments 5, pilose (Fig. 44B), sometimes glabrescent at fruiting stage with spiny (subulate or acicular), star-like projections up to 3.0 mm long that can be glabrous or with simple white hairs. Stamens 5. Fruit ovoid.

###### Habitat.

Rocky or sandy areas at elevations of 3000–4600 m. A rare plant in North India (Hartmann 1983, sub *Basssiafiedleri*).

###### Phenology.

Flowering: July-September; fruiting: August-October.

###### Distribution.

See Fig. 43.

###### Specimens examined.

**CHINA: Xizang: Ngari Prefecture**: Gêrzê (Gaize) County, 4250 m a.s.l., Aug 1972, *Li 064* (PE00146155); Gar (Gaer) County, nr Shiquanhe river, 4260 m a.s.l., 6 Aug 1976, *Qinghai-Tibet Team Vegetation Group 13430* (PE00146158); Rutog (Ritu) County, Domar (Duoma) District, 4300 m a.s.l., 20 Aug 1976, *Qinghai-Tibet Team Vegetation Group 13628* (PE00146160); Rutog (Ritu) County, Rabang (Rebang) Distr., 4300 m a.s.l., 24 Aug 1976, *Qinghai-Tibet Team 76-9119* (PE00146162); Rutog (Ritu) County, Lake Bangong (Pangong Tso), 4220 m a.s.l., 3 Sep 1976, *Qinghai-Tibet Team Meadow Group 76-236* (PE00541030), *Qinghai-Tibet Team 76-8740* (KUN0586560); Rutog (Ritu) County, E of Lake Bangong (Pangong Tso), 4250 m a.s.l., 3 Sep 1976, *Qinghai-Tibet Team Vegetation Group 13548*(PE00146638); Rutog (Ritu) County, nr Rejiao, 4350 m a.s.l., 9 Sep 1976, *Qinghai-Tibet Team Vegetation Group 13563* (PE00234846); Gê’gyai (Geji) County to Shiquanhe, 4400 m a.s.l., 21 Aug 1990, *Y. Fei* et al. *427* (KUN0264804); Zanda (Zhada) County, Zanda valley, 3600 m a.s.l., 31°29.973'N, 79°45.934'E, 6 Sep 2012, *FLPH Tibet Expedition 12-0256* (PE01967930);

**INDIA**: **Jammu & Kashmir**: Ladakh, Kaltse to Damkar, Jul 1856, *Schlagintweit* s.n. (BM000521099); Ladakh, Indus valley nr Pituls, 3 Sep 1956, *Vogt 1052* (LE); Ladakh, Chusul, 3400 m a.s.l., 12 Sep 1970, *U.C. Bhattacharyya 41119* (BSD); Ladakh, 17 km E of Khahi, 3160 m a.s.l., 26 Jul 1976, *H. Hartmann 2123* (G152730; MSB137912), nr Leh, 15 Aug 1982, *P.K. Hajra 74102* (BSD); Upper Ladakh, 3650 m a.s.l., 15 Jul 1992, *H. Hartmann 4019* (G455283, MSB137911); Ladakh, Nubra valley, Panamik, 3230 m a.s.l., 5 Aug 1995, *H. Hartmann 5033* (MSB137910); Ladakh, W of Pangong Tso lake, 4300 m a.s.l., 13 Aug 1997, *H. Hartmann 6077* (MSB137909); Ladakh, Nubra valley, Diskit, 3200 m a.s.l., 16 Aug 1998, *G.S. Goraya* et al. *1104* (BSD); Ladakh, Indus valley, Stot (E) [Stod River valley], Puga, 4430 m a.s.l., 8 Sep 1999, *L. Klimeš 777* (PRA); Ladakh, Shyok (W & C) Region, 3710–3780 m a.s.l., 15 Sep 2001, *L. Klimeš 1860* (PRA); Ladakh, Nubra Region, Yulskam vill., 3250 m a.s.l., 18 Sep 2001, *L. Klimeš 1937* (PRA); Ladakh, Indus valley, Leh, 21 Sep 2001, *L. Klimeš 1970* (PRA); Ladakh, Pangong Region, Parma vill., 4590 m a.s.l., 22 Sep 2003, *L. Klimeš 3570* (PRA).

###### General distribution.

N Himalaya, Tibet and Central Asia.

### Subfam. Salsoloideae Raf., Fl. Tellur. 3: 45 (1837)

The subfamily (excluding Camphorosmoideae) contains at least 400 species divided into ~30 genera.

#### Tribe Salsoleae

##### 17. Salsola

Taxon classificationPlantaeCaryophyllalesChenopodiacea

L., Sp. Pl.: 222 (1753)

S. kali L. (**Type**).
=Kali Miller, Gard. Dict. Abr., ed. 4: [715] (1754). **Type**: K.turgidum (Dumort.) Gutermann, Phyton (Horn) 51(1): 98 (2011).

###### Taxonomic notes.

The taxa related to *Salsolakali* were considered within the reinstated genus *Kali* (Akhani et al. 2007), which was accepted in subsequent taxonomic treatments (Gutermann 2011, Sukhorukov et al. 2011, Sukhorukov 2014, Brullo et al. 2015, Sukhorukov et al. 2016b). However, Mosyakin et al. (2014, 2017) presented some arguments in favour of a recent nomenclatural proposal to conserve the generic name *Salsola* with *S.kali* (=*Kaliturgidum*) as a conserved type. The Nomenclature Committee for Vascular Plants did not reach a consensus regarding the recommendation or rejection of this proposal (Applequist 2016), but the accepted type of the genus *Salsola* is *S.kali* under the new Art. 10.5–10.7 of the ICN (Turland et al. 2018).

###### Description.

Annuals or subshrubs, glabrous or papillate, with tufts of simple curved hairs in the leaf axils. Leaves mostly alternate or lower leaves opposite, semi-terete or terete, sometimes flattened, usually less than 5 mm in width, stiff or fleshy, with a persistent yellowish mucro up to 3.5–4.0 mm long. Bracts longer than bracteoles or equal in size. Flowers of two types: some located below the main inflorescence and arranged in clusters mostly consisting of two female flowers supported by concrescent and basally hardened bracts and bracteoles, rarely clusters one-flowered (these fall off with bracts and bracteoles), perianth r-shaped, usually with small wings, tubercles or without any projections in the flexure; flowers of the second type grouped in the main inflorescence, hermaphrodite, each flower supported by a free bract and two bracteoles (rarely concrescent), r-shaped or tubuliform at fruiting (in the latter case, the segments in their upper part are convergent, forming a stout or hyaline conus that covers the fruit from above). Perianth of (4)5 segments, with well-developed, equal or unequal wings or tubercles at the fruiting stage. Stamens (in the hermaphrodite flowers) 5, divided in the lower half, without prominent appendages at the tip of the anthers. Stigmas 2, usually equal to style (or a style is very short). Fruits dry or somewhat fleshy in the upper part. Seeds with horizontal or obliquely orientated embryo (sometimes seems to be vertical due to anacrostyly). Perisperm absent.

Approximately 22 species in the steppes, seashores and deserts of Eurasia, North America, Africa and Australia.

##### Key to the species

**Table:** 

1	Bracts appressed to the stem; perianth (of the flowers located in the main inflorescence) at fruiting with tubercles; alternatively, two (out of five) perianth segments bear short wings up to 1.5 mm long	**2. *S.collina***
–	Bracts (often horizontally) spreading, rarely appressed (some specimens of *S.monoptera*); at least 1 of 5 segments with well-developed wing(s)	**2**
2	Perianth segment closest to the bract with conspicuous wing, the others with tubercles; all outgrowths orientated vertically	**6. *S.monoptera***
–	All 5 or at least 2 or 3 segments with horizontally reflexed or obliquely orientated outgrowths	**3**
3	Leaves linear to lanceolate, (1.0)2.0–4.0(5.0) mm in width; perianth conus slightly hardened	**5. *S.austrotibetica***
–	Leaves filiform to linear, not exceeding 1.5(2) mm in width (except the broad leaf base); perianth conus not expressed or hyaline	**4**
4	Bract and bracteoles basally gibbous; winged perianth 2.0–3.0 mm across; only 2 or 3 (of 5) perianth segments with well-developed wings (other segments with tubercles only); anthers 0.3–0.5 mm long	**1. *S.hartmannii***
–	Bract and bracteoles usually not gibbous; winged perianth 3.5–10.0 mm across; all perianth segments form equal or slightly unequal wings; anthers 0.5–0.8 mm long	**5**
5	Plant usually densely papillate; perianth with wings 3.5–6.0 mm across. Plants of Central Himalaya and Tibet	**3. *S.jacquemontii***
–	Plant glabrous or papillate; perianth with wings (6.0)7.0–10.0 mm across. Plants of North Himalaya	**4. *S.tragus***

###### 1. Salsola hartmannii

Taxon classificationPlantaeCaryophyllalesChenopodiacea

Sukhor. sp. nov.

urn:lsid:ipni.org:names:77194256-1

Fig. 45

####### Holotype.

[INDIA, Jammu & Kashmir] E-Ladakh, Alpine Steppe mit *Artemisiaminor*, ca. 1 km W oberhalb Kiagar Tso [alpine steppe with *Artemisiaminor*, ca. 1 km W above Kiagar Tso], 4720 m a.s.l., 27 Aug 1997, *H. Hartmann 6080* (G00410003! isotype – MSB160560!).

####### Taxonomic notes.

The new species was formerly identified as *S.jacquemontii* or *S.collina*. In many characters (e.g. tiny anthers, small outgrowths of the perianth segments), *Salsolahartmannii* resembles *S.monoptera*, but the latter species has only one well-expressed, wing-like projection on a segment closest to the bract, whereas projections on other segments are much smaller (in the form of tubercles). Additionally, the gibbous bracts and bracteoles are exceptionally rare in *S.monoptera*. In contrast, *S.hartmannii* possesses gibbous bracts and bracteoles and two or three perianth segments develop tiny, but clearly visible wings. The new species is also similar to *S.jacquemontii* but has smaller anthers and a smaller diameter of the entire diaspore (winged perianth enclosing the fruit), which is hidden within the subtending bract and bracteoles.

The gibbous bract and bracteoles cannot be considered as strong characteristic features of *S.hartmannii*; some specimens of *S.jacquemontii* may possess such a shape of the bracts and bracteoles. All three species, *S.monoptera*, *S.hartmannii* and *S.jacquemontii*, are morphologically related. *Salsolacollina* is morphologically distant from these species, having appressed bracts without clearly expressed wings on the perianth at fruiting. The distribution patterns are also different: *S.monoptera* is widespread in Central Asia and Tibet, *S.hartmannii* seems to be localised in North Himalaya and Karakoram and *S.jacquemontii* is found in Central Himalaya and Tibet. The presence of *S.jacquemontii* and *S.collina* in North Himalaya (Rilke 1999) is not confirmed.

####### Description.

Annual with prostrate or ascending stems up to 15 cm long, glabrous or scarcely papillate. Leaves terete, 10–20 × 1.0–1.5 mm. Flowers in axillary clusters and arranged in the main inflorescence. Flower clusters (located below the main inflorescence) abundant and consist of two flowers covered with bracts and bracteoles that are connate almost to the top and basally gibbous; perianth segments membranous, fimbriate, r-shaped, their tips more or less appressed to each other, not hardened and not forming a tight conus, with white, small tubercles 0.3–0.5 mm long in the flexure; styles with stigmas ca. 0.5 mm; fruits 1.2–1.6 mm, depressed roundish. Flowers in the main inflorescence with (almost) free, basally gibbous bract and bracteoles, hermaphrodite; perianth segments 5, membranous, fimbriate, r-shaped at the fruiting stage, < 2.5 mm long, above the flexure soft, hyaline, horizontally orientated and not forming a distinct conus; 2 or 3 (of 5) segments develop wing-like appendages (up to 1.5 mm long) that are located almost horizontally or obliquely, two other segments develop tubercles only; the whole diaspore with the perianth wings is 2.0–3.0 mm across and is hidden within the bract and bracteoles; anthers 0.3–0.5 mm long; styles with stigmas ca. 1 mm; fruits lenticular, 1.1–1.5 mm. Seeds with horizontal or obliquely orientated embryo.

**Figure 45.**
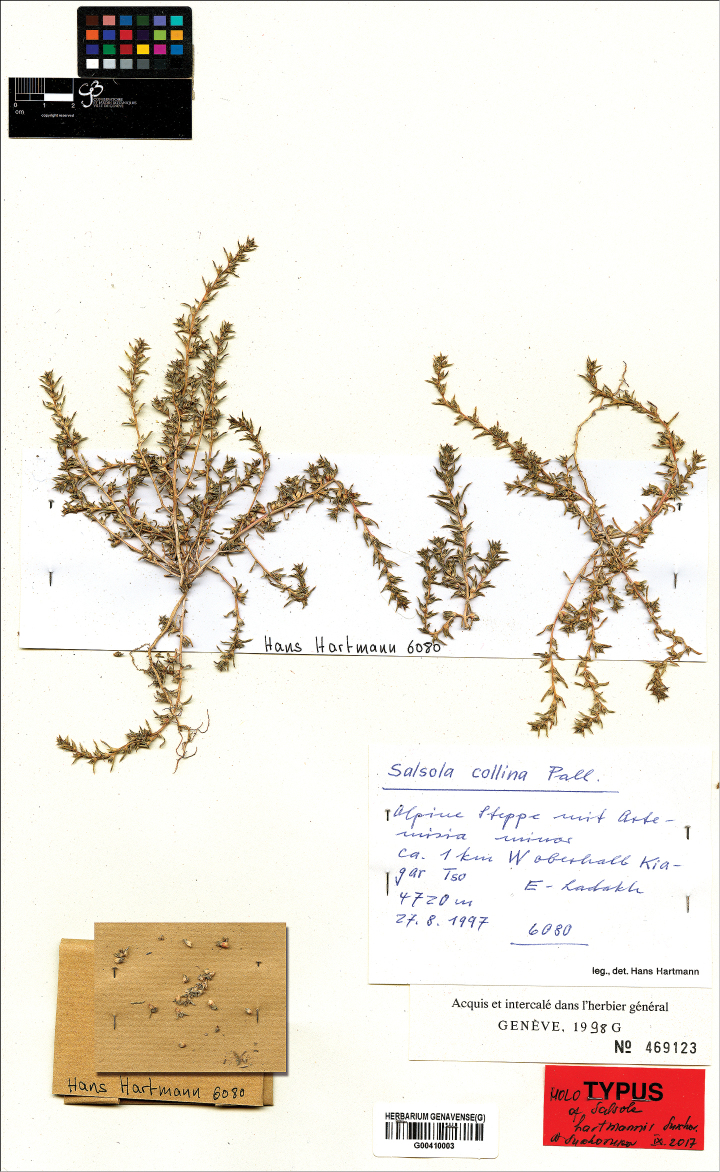
Holotype of *Salsolahartmannii* Sukhor. sp. nov. (G).

####### Habitat.

Gravelly or sandy substrates at altitudes of 3500–5200 m. The species seems to be common in Ladakh and Spiti Valley.

####### Phenology.

Flowering: August-September; fruiting: September-October.

**Etymology**: The specific epithet is after Hans Hartmann, a Swiss geobotanist who provided significant contributions to the flora and vegetation of North India.

####### Distribution.

See Fig. 46.

**Figure 46.**
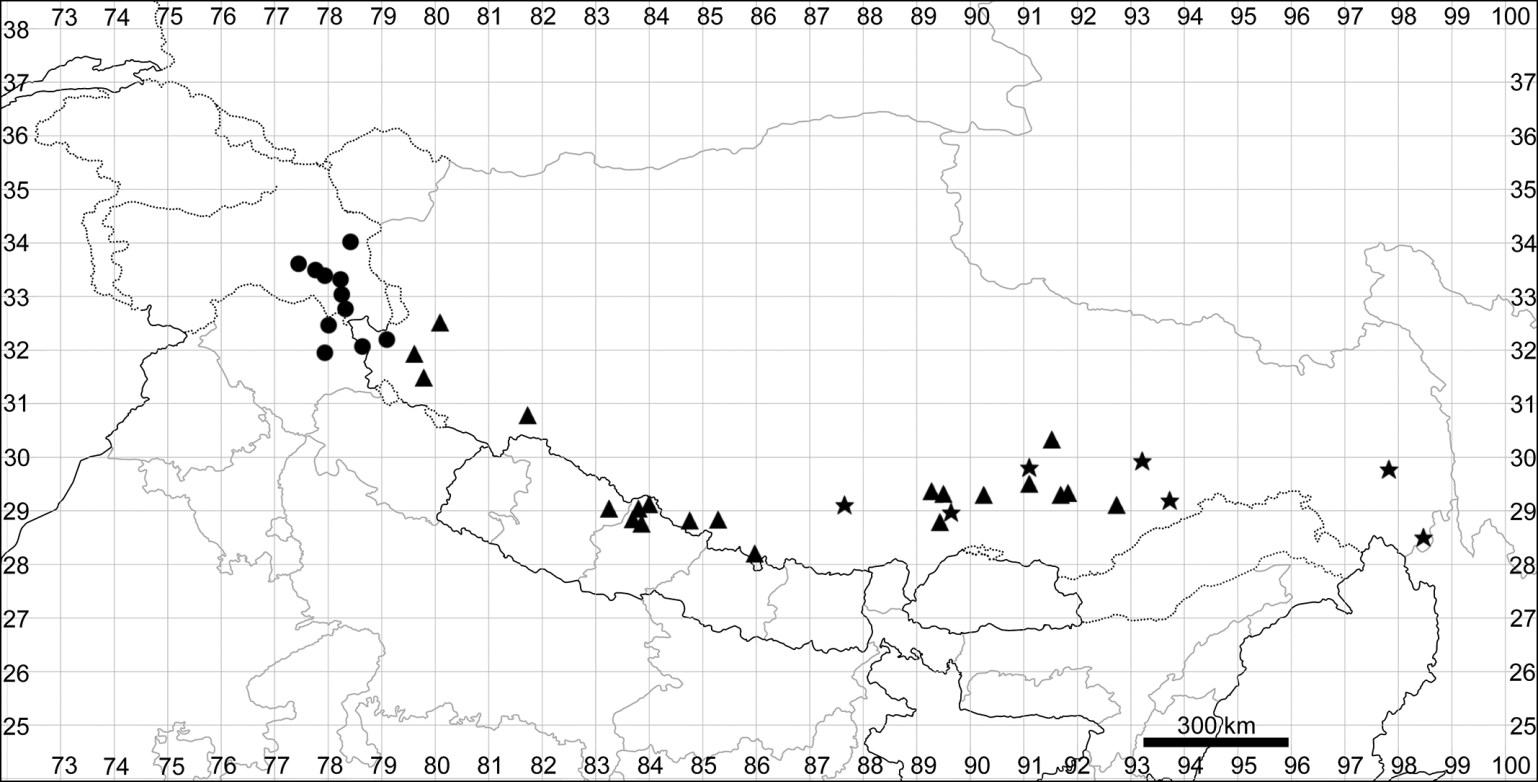
Distribution map of *Salsolahartmannii* (circles), *S.collina* (stars) and *S.jacquemontii* (triangles).

####### Specimens examined.

**CHINA**: **Xizang**: **Ngari Prefecture**: [Zanda County] Tisum, 15000 ft a.s.l., [without date] *R. Strachey & J.E. Winterbottom* s.n. (BR);

**INDIA: Jammu & Kashmir**: Naini valley to Srinagar, [without date and collector] (BM); [Ladakh] Parang valley, 11 Sep 1847, *T.T. Thomson* s.n. (K); Ladakh, Muklip to Lukung, ca. 4000 m a.s.l., 29 Aug 1976, *B.M. Wadhwa 60152* (BSD); [Ladakh] Debring to Tanglang La, 4800–5200 m a.s.l., 3 Sep 1989, *L. Klimeš & M. Šrůtek 28/37* (PRA); Rupshu, N of Tso Kar, ca. 4600 m a.s.l., 22 Aug 1995, *H. Hartmann 5036* (G455293, MSB160561); Ladakh, Zanskar Region, Zara, N of Kurio, 33°35'30"N, 77°28'E, 4250 m a.s.l., 23 Aug 1998, code 98-11-2, *L. Klimeš 161* (PRA); Ladakh, Indus valley, Stot (E) [Stod River valley], between Angkung & Puga, 33°14'N, 78°16'E, 4550 m a.s.l., 8 Sep 1999, *L. Klimeš 776* (PRA); Ladakh, Rupshu Region, Tso Moriri, Lema to Peldo, 33°1'53"N, 78°16'34"E, 4550–4560 m a.s.l., 13 Sep 2005, *L. Klimeš 6301* (PRA); **Himachal Pradesh**: Parang La, 11 Sep 1847, *anonym* s.n. (K); Spiti valley, Samdo to Kaurik, 21 Aug 1994, *S.K. Murti & S. Singh 81588* (BSD); Pin Valley National Park, Mane vill., 3700 m a.s.l., Sep 2002, *K.C. Sekar 100736* (BSD).

####### General distribution.

North India, NW China (Xizang) and North Pakistan ([Gilgit-Baltistan] Nagar, dry stony slope, 9000 ft a.s.l., 22 Aug 1987, *Jamshed 239* (E!)).

###### 2. Salsola collina

Taxon classificationPlantaeCaryophyllalesChenopodiacea

Pall., Ill. Pl.: 34 (1803)

Salsola collina Pall., Ill. Pl.: 34 (1803). **Lectotype** (Rilke 1999): Habitat in Siberia, *Pallas* s.n. (LIV, isotype BM!).
≡SalsolakaliL.subsp.collina (Pall.) O.Bolós & Vigo, Butl. Inst. Catalana Hist. Nat., Secc. Bot. 38(1): 89 (1974).
≡Kalicollinum (Pall.) Akhani & Roalson, Int. J. Pl. Sci. 168(6): 946 (2007). The full list of the synonyms is presented in Rilke (1999).

####### Description.

Erect or prostrate (in mountainous areas) annual up to 70 cm, stems reddish, glabrous or scarcely papillate. Lower leaves up to 80.0 × 0.5–1.0 mm, much shorter in the middle and upper stem parts, linear or filiform, with a mucro 0.6–2.0 mm long. Flowers in axillary clusters and arranged in the main inflorescence. Flower clusters (located below the main inflorescence) abundant, consisting of two flowers surrounded by bracts and bracteoles that are connate almost to the top and not gibbous basally; perianth segments of each flower membranous, glabrous, r-shaped, their tips appressed to each other, hardened and not forming a tight conus, with white, small tubercles 0.3–0.5 mm long or without any projections in the flexure; styles with stigmas ca. 1.0 mm; fruits 1.0–1.3 mm, depressed roundish. Flowers in the main inflorescence with (almost) free, basally not gibbous bract and bracteoles (that are appressed to the stem), hermaphrodite; perianth segments membranous, glabrous, r-shaped at fruiting stage, (1.5)2.0–2.5 mm long, membranous above the flexure and not forming a distinct conus; two abaxial segments usually have short erose wings (up to 1.0(1.5) × 1.5 mm), their tips (above outgrowths) hyaline and not stout; anthers 0.6–0.9 mm long; styles with stigmas ca. 1.0 mm; fruits lenticular, 1.1–1.5 mm. Seed seems to be vertical (due to anacrostyly) but is indeed horizontal.

####### Habitat.

Cold stony deserts at elevations of 2800–4400 m.

####### Phenology.

Flowering: July-September; fruiting: September-October.

####### Distribution.

See Fig. 46.

####### Specimens examined.

**CHINA**: **Xizang**: **Xigazê Prefecture**: Lhazê (Lazi) County, Kabei vill., 4000 m a.s.l., 27 Aug 1961, *Zhang 2726* (PE02048027); Gyangzê (Jiangzi) County, 3960 m a.s.l., 7 Sep 1974, *Qinghai-Tibet Team 74-2107* (PE00166821); **Lhasa City**: Lhasa, Aug 1939, *Richardson 338* (BM); W of Lhasa, 3700 m a.s.l., 3 Sep 1960, *Xia 815* (PE00166830); Lhasa, 3800 m a.s.l., 16 Aug 1965, *Zhang & Lang 1533* (PE00166814); N of Lhasa, 3700–3800 m a.s.l., 23 Aug 1984, *R.F.Huang CG-552* (HNWP109096); **Nyingchi Prefecture**: [Zayü (Chayu) County] Cawarong (Chawalong), 3000 m a.s.l., Aug 1935, *C.W. Wang 65180*(PE00166833); [Gongbo’gyamda (Gongbujiangda) County] Songduo to Apei, 3800 m a.s.l., 16 Sep 1965, *Zhang & Lang 2747* (PE00166817); Mainling (Milin) County, Jiage, 3200 m a.s.l., 11 Jul 1972, *Tibet Chinese Herbal Medicine Survey Team 3686* (PE00166824); Zayü (Chayu) County, Zha’na [close to Cawarong], 2000 m a.s.l., 3 Oct 1982, *Qinghai-Tibet Team 10919* (PE01063467); **Qamdo Prefecture**: [Baxoi (Basu) County] Bamda (Bangda) to Zogang (Zuogong), 3700 m a.s.l., 29 Aug 1976, *anonym 76-574* (PE00513543).

The specimens from India reported by Dhar and Kachroo (1983) and Klimeš and Dickoré (2005) were not examined by us. All investigated specimens belong to *Salsolahartmannii* and *S.tragus*. However, *S.collina* is present in North Pakistan and its occurrence in Jammu and Kashmir is predictable.

####### General distribution.

Temperate Eurasia and East Europe; alien in West/Central Europe, Scandinavia and North America.

###### 3. Salsola jacquemontii

Taxon classificationPlantaeCaryophyllalesChenopodiacea

Moq. in DC., Prodr. 13(2): 188 (1849)

Fig. 47

Salsola jacquemontii Moq. in DC., Prodr. 13(2): 188 (1849). **Lectotype** (designated by Rilke in Freitag and Rilke 1997): “Aux Indés orient.” [In East India] V. Jacquemont 2114 (P00163982! image available at http://science.mnhn.fr/institution/mnhn/collection/p/item/p00163982; isotype P00163983!).
≡Kalijacquemontii (Moq.) Akhani & Roalson, Int. J. Pl. Sci. 168(6): 946 (2007).
=Salsolanepalensis Grubov, Bot. Mat. Gerb. Bot. Inst. Komarova Akad. Nauk SSSR 21: 127 (1961). **Holotype**: NEPAL, [Karnali zone, Dolpa distr.] below Rohagaon, Suli Gad, 8500 ft a.s.l., growing amongst shrubs on dry slopes, 13 Sep 1952, O. Polunin, W.R. Sykes & L.H.J. Williams 3357 (LE, isotypes BM000016772! image available at http://www.nhm.ac.uk/emu-image-download/class.EMuMedia.php?irn=178999&image=yes&width=705; E00688195!).
≡Kalinepalense (Grubov) Brullo, Giusso & Hrusa, Phytotaxa 201(4): 271 (2015).

####### Taxonomic notes.

The morphological differences between *S.tragus* and *S.jacquemontii* are vague (also, see the diagnostic key in Rilke 1999). The latter species, however, is encountered in the Central Himalaya and South Tibet, where *S.tragus* is absent. Generally, *S.jacquemontii* has smaller anthers, perianth and wings at the fruiting stage than those of *S.tragus*. Additionally, the stem is usually straight in *S.tragus* and prostrate in *S.jacquemontii*, although some forms with a straight stem were also observed by AS at lower altitudes (2600–3000 m) in Central Nepal.

####### Description.

Annual up to 50 cm, prostrate or rarely with prominent upright main stem. Stem and branches green or reddish, densely covered with papillae. Lower leaves up to 35 × 2 mm (upper leaves much shorter, up to 15.0 mm long), with a mucro (1.5)2.0–3.0 mm long. Flowers in axillary clusters and arranged in the main inflorescence. Clusters (located below the main inflorescence) consist of two female flowers supported by a bract and two bracteoles that are connate almost to the top and usually not gibbous at the base; perianth segments membranous, fimbriate, r-shaped with their tips appressed to each other, not hardened and not forming a tight conus, with small, reddish or white tubercles up to 0.6 mm long in the flexure; styles with stigmas ca. 1.0 mm long; fruits 1.3–1.6 mm in diameter. Flowers in the main inflorescence with (almost) free, usually not gibbous, bract and bracteoles, hermaphrodite; perianth segments membranous, fimbriate, r-shaped at fruiting stage, < 2.5 mm long, soft above the flexure, hyaline, 0.6–0.8 mm long, horizontally appressed and not forming a distinct conus; three segments with horizontally orientated wing-like appendages (< 3.0 mm long), two other segments with tubercles only (the entire diaspore with the perianth wings is 3.5–6.0 mm across); anthers 0.5–0.7 mm long; styles with stigmas ca. 1.0–1.5 mm long; fruits lenticular, 1.1–1.5 mm in diameter. Seeds with horizontal or obliquely orientated embryo.

**Figure 47.**
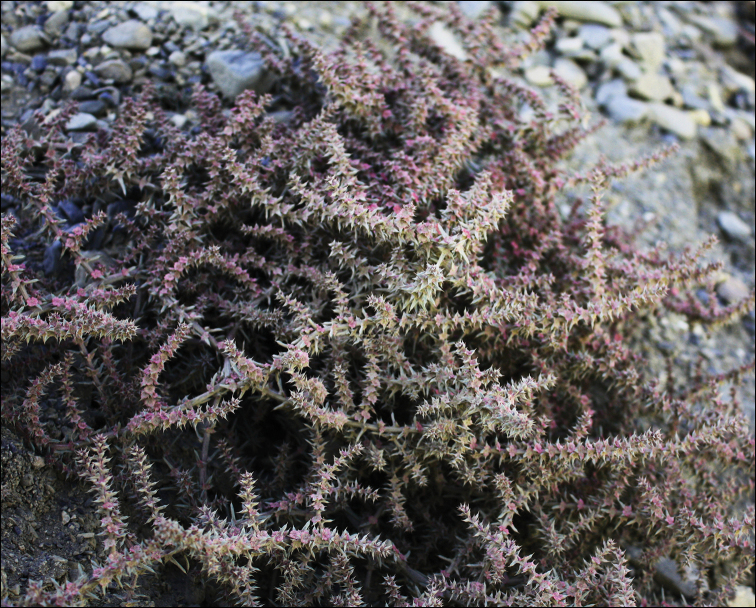
*Salsolajacquemontii* plant at fruiting stage. Photograph by A. Sukhorukov (near Kagbeni vill., Dhaulagiri zone, Nepal, September 2009).

####### Habitat.

Gravelly substrates, limestone at elevations of 2600–4200 m.

####### Phenology.

Flowering: July-September; fruiting: August-October.

####### Distribution.

See Fig. 46.

####### Specimens examined.

**CHINA**: **Xizang**: **Ngari Prefecture**: [Gar (Gaer) County] Shiquanhe, 4300 m a.s.l., 31 Jul 1974, *Biological Institute Tibet Expedition Team 3770* (PE00528010); Zanda (Zhada) County, Toling (Tuolin), 3700 m a.s.l., 6 Aug 1974, *Biological Institute Tibet Expedition Team 3814* (PE00528530); Zanda (Zhada) County, Qangzê (Xiangzi) Distr., 4100 m a.s.l., 5 Jul 1976, *Qinghai-Tibet Team 76-7925* (PE00528011); Zanda (Zhada) County, Qangzê (Xiangzi) distr., 4100 m a.s.l., 5 Jul 1976, *Qinghai-Tibet Team Meadow Group 76-084* (PE00541025); Burang (Pulan) County, E of Hor, 4682 m a.s.l., 30°42'52.86"N, 81°42'54.21"E, 3 Sep 2012, *FLPH Tibet Expedition 12-0069* (PE01967933); Zanda (Zhada) County, Tulin valley, 3960 m a.s.l., 31°28.12'N, 79°46.12'E, 6 Sep 2012, *FLPH Tibet Expedition 12-0250* (PE01967936); **Xigazê Prefecture**: Gyantse plain, 20 Jul 1907, *R.R*. *Stewart* s.n. (E); Nyalam (Nielamu) County, 4200 m a.s.l., 6 Sep 1972, *Tibet Chinese Herbal Medicine Survey Team 1834* (HNWP29239); Gyirong (Jilong) County, Kungtang (Gongdang) Distr., 3900 m a.s.l., 1 Aug 1975, *Qinghai-Tibet Team 4143* (PE00528009); Nielamu [Nyalam], 4300 m a.s.l., 6 Sep 1984, *G. Miehe 1627* (BM); Upper Trisuli gorge, NW of Xixabangma Mt., 28°48’N 85°18’E, 4060 m a.s.l., 25 Aug 1993, *G. & S. Miehe 9569/09* (herb. Miehe); **Lhasa City**: Lhasa, 12000 ft a.s.l., 5 Sep 1943, *F. Ludlow & G. Sherriff 9922* (BM000016628, LE); Lhasa, 3660 m a.s.l., 1 Oct 1961, *Tibet Team 1785* (PE00528008); Nyemo, 29°18'N, 90°18'E, 3800 m a.s.l., 18 Aug 1993, *G. & S. Miehe 9505/03* (herb. Miehe); Lhasa, 29°19'40.4"N, 89°28'30.9"E, 15 Aug 2011, *Yu* et al. *5554* (PE); **Shannan Prefecture**: Nêdong (Naidong) County, Zêtang (Zedang), 3650 m a.s.l., 13 Jul 1975, *Wu* et al. *75-759* (PE00528007); Nêdong (Naidong) County, Luoqiong vill., 3569 m a.s.l., 29°15'57.23"N, 91°49'1.76"E, 2 Sep 2012, *FLPH Tibet Expedition 12-0608* (PE01967940); **Nyingchi Prefecture**: Tsangpo valley, 10000–11000 ft a.s.l., 5 Sep 1935, *F. Kingdon-Ward 12308* (BM); Nang (Lang) County, Zhongda, 3150 m a.s.l., 1 Sep 1972, *Tibet Chinese Herbal Medicine Survey Team 4518* (PE00528529);

**NEPAL**: **Midwestern**: **Karnali Zone**: [Dolpa Distr.] Saldanggam, Chharkabhot, 15500 ft a.s.l., 24 Jun 1952, *O. Polunin*, *W.R*. *Sykes* & *L.H.J*. *Williams 1199* (BM); **Western**: **Dhaulagiri Zone**: [Mustang Distr.] Kagbeni, 10000 ft a.s.l., 8 Jun 1954, *J. Stainton, W.R. Sykes & L.H.J. Williams 5660* (BM, P04952818); [Mustang Distr.] Thinigaon, Mukhtinath Himal, 11500 ft a.s.l., 24 Jun 1954, *J. Stainton, W.R. Sykes & L.H.J. Williams 1369* (BM, LE); [Mustang Distr.] Yara (S of Mustang), 12000 ft a.s.l., 2 Aug 1954, *J. Stainton, W.R. Sykes & L.H.J. Williams 2131* (BM); [Upper Mustang] Tange, 3500 m a.s.l., 22 Aug 1956, *Lobbichler 30* (M); [Mustang Distr.] Kali Gandaki, nr Marpha, 8500 ft a.s.l., 15 Jul 1977, *G. Miehe 165* (BM); Cha Lungpha, 10000 ft a.s.l., 31 Aug 1977, *G. Miehe 563* (BM); Mustang Distr., 3280 m a.s.l., 26 Sep 1995, *Mikage* et al. *9552530* (BM); [Mustang Distr.] Kagbeni, 9 Jul 2000, *Y. Iokawa* et al. *20020016* (E00246126); Mustang Distr., Lower Bhena Khola, 28°59'N, 83°49'E, 25 Aug 2001, *S. & G. Miehe & Koch 01-069-09* (herb. Miehe); [Mustang Distr.] nr Kagbeni & Jomosom vill., 3000 m a.s.l., Sep 2009, *A. Sukhorukov* s.n. (G, MW).

####### General distribution.

Central Himalaya and Tibet.

###### 4. Salsola tragus

Taxon classificationPlantaeCaryophyllalesChenopodiacea

L., Cent. Pl. 2: 13 (1756)

Salsola tragus L., Cent. Pl. 2: 13 (1756). **Lectotype** (designated by Degen 1937): Herb. Linn. 315.3 (LINN). The list of the synonyms is extremely large for this species (Rilke 1999), but some varieties and species described from Europe or Central Asia require additional study.

####### Description.

Annual up to 70 cm high. Stem branched from the base, finely papillate or glabrous as well as the leaves. Leaves 20.0–80.0 × 1.0–2.0 mm, linear, with a mucro (1.5)2.0–3.5 mm long. Flowers in axillary clusters and arranged in the main inflorescence. Clusters (usually located below the main inflorescence) consist of two often unequal female flowers, each flower supported by a bract and two bracteoles and additional bracts are sometimes present in the clusters; bracts and bracteoles fused in their basal part; perianth segments free, membranous, r-shaped, their tips appressed to each other, not hardened, not forming a conus, with small or hardly noticeable, red to pinkish wings in the flexure; styles with stigmas ca. 1.5 mm long; fruits 1.0–1.6 mm, depressed roundish to ovoid. Flowers in the main inflorescence with a free bract and two bracteoles (shorter than bract or equal in length), hermaphrodite; perianth segments membranous, tubuliform (not r-shaped), 3.0–4.0 mm long at fruiting stage, their lower part marginally glabrous or minutely fimbriate; segment tips hyaline, forming a convergent, glabrous conus 1.0–1.5 mm long with well-developed, horizontally located or slightly recurved, entire to crisp wings in the flexure; three outer segments develop roundish wings and two inner segments have narrower, lanceolate outgrowths (the whole diaspore with the perianth wings is (6.0)7.0–10.0 mm across); anthers 0.6–0.8 mm long (on specimens from our region); styles with stigmas 1.0–1.5 mm; fruits 1.5–1.8 mm, roundish. Seeds with horizontal embryo.

####### Habitat.

Rocky or sandy places at altitudes of 2700–4200 m.

####### Phenology.

Flowering: July-September; fruiting: August-October.

####### Distribution.

See Fig. 48.

**Figure 48.**
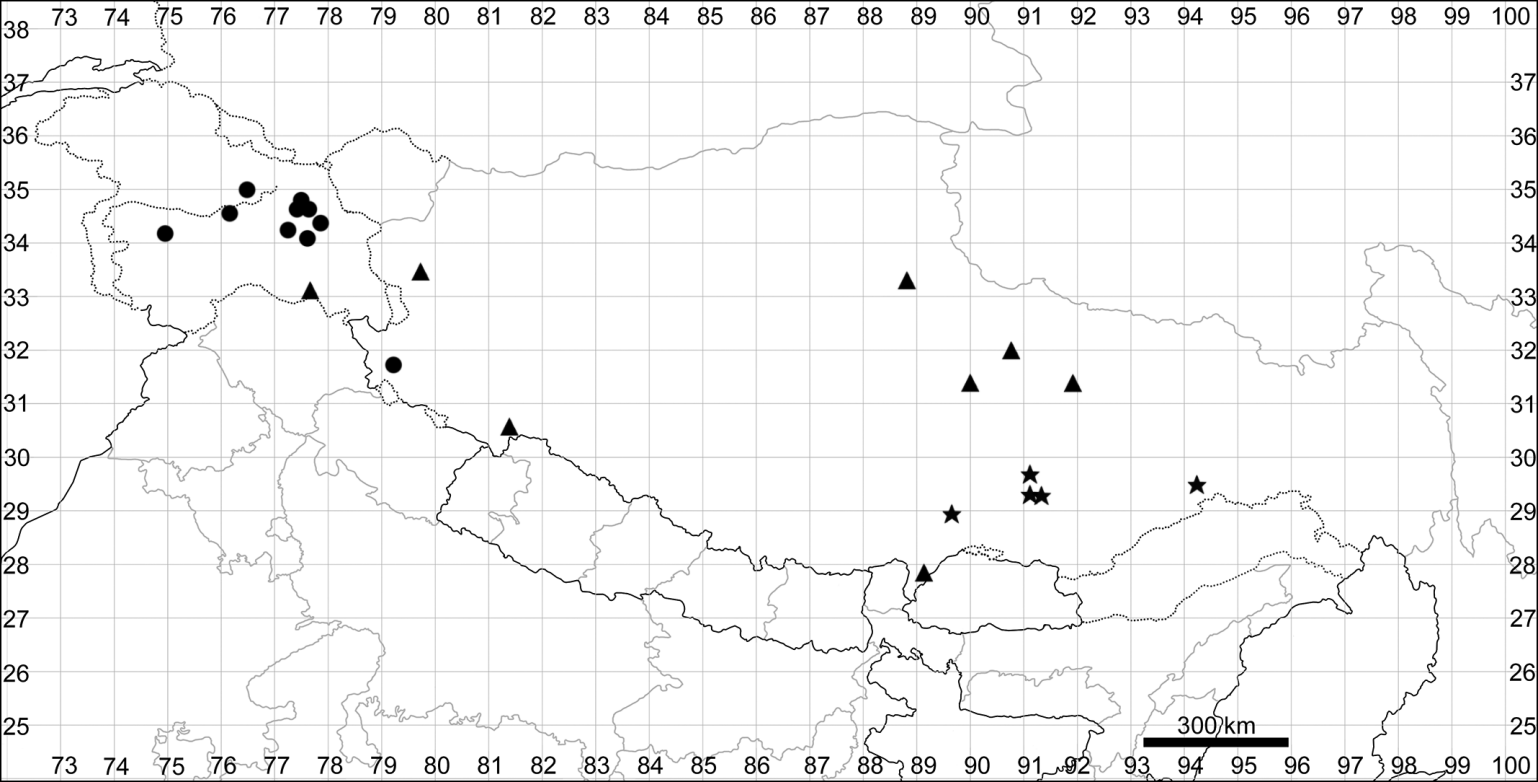
Distribution map of *Salsolatragus* (circles), *S.austrotibetica* (stars) and *S.monoptera* (triangles).

####### Specimens examined.

**CHINA**: **Xizang**: **Ngari Prefecture**: Zanda (Zhada) County, Diyag (Diya), Gulang, 2900 m a.s.l., 2 Jul 1976, *Qinghai-Tibet Team 76-8156* (PE00528531);

**INDIA**: **Jammu & Kashmir**: Shayuk [Shyok] valley, 30 Oct 1847, *T.T. Thomson* s.n. (K); Nubra valley toward Leh, 15 Sep 1848, *T.T. Thomson* s.n. (K); Srinagar, [without date] *Benham* s.n. (BM000016607); Ladakh, Leh to Kaltse, Jul 1856, *Schlagintweit 1034* & *1507* (BM000016644, P05007283); Ladakh, Nubra valley, Diskit, 3200 m a.s.l., 16 Aug 1998, *G.S. Goraya* et al. *1091* (BSD); Ladakh, Tirit vill., 3200 m a.s.l., 18 Aug 1976, *B.M. Wadhwa 59979* (BSD); Leh, 3620 m a.s.l., 13 Jul 1992, *H. Hartmann 4018* (G); Ladakh, Indus valley, Leh, 3650 m a.s.l., 13 Aug 1999, *L. Klimeš 463* (PRA); Ladakh, Shyok (W & C), Agham, 3320 m a.s.l., 16 Sep 2001, *L. Klimeš 1877* (PRA); Ladakh, Suru Region, Kargil, 2720–2860 m a.s.l., 18 Sep 2004, *L. Klimeš 4977* (PRA).

####### General distribution.

Temperate Eurasia, North Africa, alien in the Americas and Australia.

###### 5. Salsola austrotibetica

Taxon classificationPlantaeCaryophyllalesChenopodiacea

Sukhor. sp. nov.

urn:lsid:ipni.org:names:77194257-1

**Figure 49.**
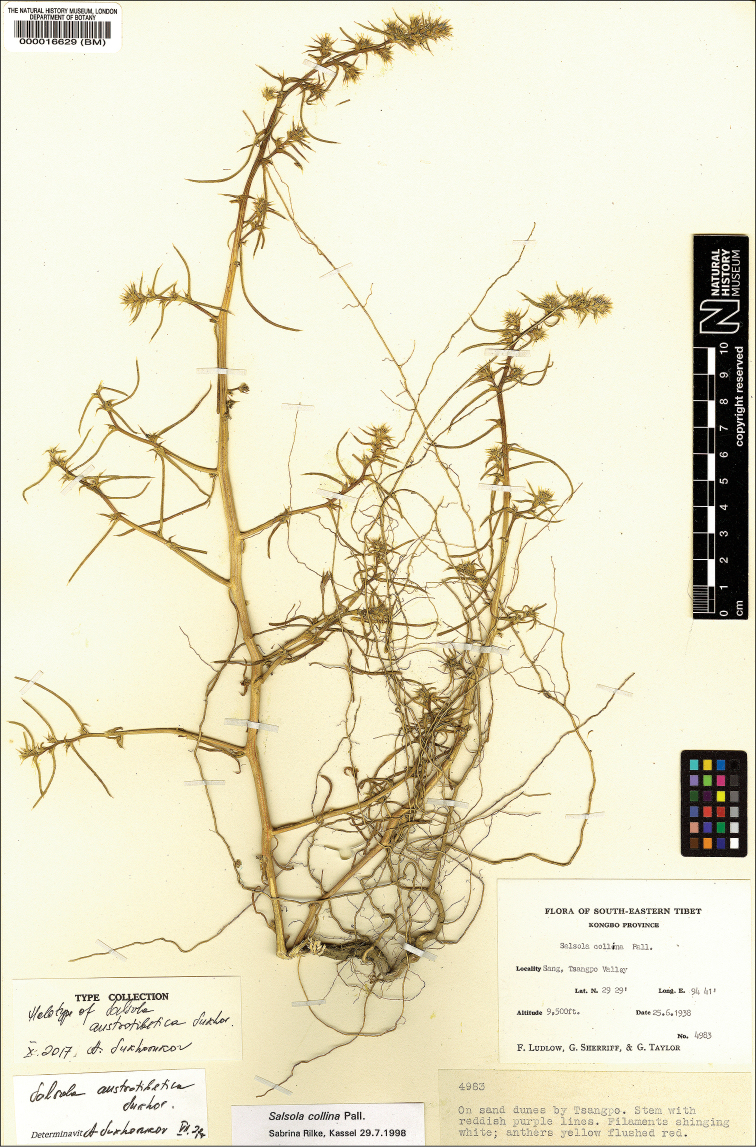
Holotype of *Salsolaaustrotibetica* Sukhor. sp. nov. (BM0000016629).

=Salsolaikonnikovii sensu Rilke (1999) non Iljin; Salsolatragus sensu Zhu et al. (2003) non L. **Holotype**: [CHINA, Xizang, Nyingchi Prefecture, Gongbo’gyamda County] Kongbo Province [Kongpo Region], Sang, Tsangpo valley, 29°29'N, 94°14'E, 9500 ft a.s.l., 25 Jun 1938, *F. Ludlow, G. Sherriff & G. Taylor 4983* (BM000016629). Fig. 49.

####### Taxonomic notes.

The hardness of the conus (perianth part above the wings or tubercles at the fruiting stage) is a very important trait for identifying the representatives of the *Salsola* type section (ex-*Kali*), which is widely used in taxonomic treatments (e.g. Iljin 1936, Tzvelev 1996, Rilke 1999). The length of the conus seems to be another important characteristic for the taxonomy of this group. All specimens of *S.austrotibetica* were formerly identified as *Salsolaikonnikovii* or rarely *S.tragus* (incl. *S.pestifer*). The identification of the Tibetan specimens as *S.ikonnikovii* (Rilke 1999) was mostly based on the similar broader and stiff leaves. *Salsolaikonnikovii* was formerly considered more broadly with a vast distribution covering Central Asia and Tibet (Rilke 1999). Although some other Central Asiatic species were included in *S.ikonnikovii* (*S.beticolor* Iljin, *S.centralasiatica* Iljin, *S.microkali* Popov, *S.potaninii* Iljin and *S.chinghaiensis* A.J.Li), the specific status of these taxa must be re-evaluated following extensive molecular and morphological investigations. All southernmost records of *Salsolaikonnikovii* cited by Rilke (1999) from Tibet [Xizang] are here considered *Salsolaaustrotibetica*.

*Salsolaikonnikovii* s. str. is characterised by a large (10–20 cm) main inflorescence with very densely arranged flowers, winged perianth 5.0–8.0 mm in diameter and perianth segments forming a narrowly triangular and stiff conus of (2)2.5–3.5 mm long. In contrast, the new species has a much smaller (up to 12 cm) inflorescence, perianth with wings 4.0–5.0 mm across and triangular and slightly hardened perianth conus 1.5–2.0 mm long. All plants in the PE herbarium were identified as *Salsolaruthenica* [≡*S.tragus*], another similar-looking species. However, linear or filiform leaves and a completely hyaline perianth conus distinguish *S.tragus* from both S. *ikonnikovii* and *S.austrotibetica*.

The distribution area of *Salsolaaustrotibetica* is restricted to South Tibet (Fig. 48) and that of *S.ikonnikovii* covers Mongolia and the northernmost provinces of China (Nei Mongol, Ningxia and Gansu).

####### Description.

Annual up to 40 cm high. Stem straight, branched from the base with ascending lateral shoots; stem and leaves finely papillate. Leaves on the main stem remote (with internodes 20–40 mm long), 20–50 mm long, 1.0–4.0(5.0) mm wide above the broad base, slightly flattened, lanceolate to linear, thick, with a mucro (1.0)1.5–2.5 mm long. Flowers in axillary clusters and arranged in the main inflorescence. Flower clusters (usually located below the main inflorescence) consist of two often unequal female flowers, each flower supported by a bract and two bracteoles, sometimes additional bracts are present in the clusters; bracts and bracteoles fused in their basal part; perianth segments of each flower membranous, r-shaped, their tips appressed to each other, not hardened, not forming a conus, with red to pinkish, small or hardly noticeable wing-like tubercles in the flexure; styles with stigmas ca. 1.5 mm long; fruits 1.0 to 1.6 mm, depressed roundish to ovoid. Flowers in the main inflorescence lax, hermaphrodite, with two bracteoles (slightly shorter than bract) and a free bract having a broad (up to 1.0 mm) white margin; perianth segments membranous, tubuliform (not r-shaped), 1.7–2.2 mm at flowering stage and elongated to 4.0–5.0 mm at fruiting, their lower part marginally glabrous or finely fimbriate; segment tips slightly hardened but not hyaline, forming a convergent, glabrous conus 1.5–2.0 mm long, with well-developed, obliquely orientated, unequal, erose to lobate wings in the flexure; the whole diaspore with the perianth wings is 4.0–5.0 mm across; anthers 0.7–0.8 mm; styles with stigmas 1.3–1.7 mm; fruit ~1.5 mm, depressed roundish. Seeds with horizontal embryo.

####### Habitat.

Sand dunes, gravelly substrates, and sometimes in dump areas at altitudes of 2700–4200 m.

####### Phenology.

Flowering: July-August; fruiting: August-September.

####### Distribution.

See Fig. 48.

####### Specimens examined.

**CHINA: Xizang: Xigazê Prefecture**: Gyangtse, 1904, *H.J. Walton* s.n. (BM); **Lhasa City**: Reting, 60 miles N of Lhasa, 13000 ft a.s.l., 31 Jul 1942, *F. Ludlow & G. Sherriff 8986* (BM, LE); Lhasa, nr the 49^th^ Hospital, 3700 m a.s.l., 7 Sep 1960, *Xia & Zhu 816* (PE00528532, PE00528533); **Shannan Prefecture**: Gonggar (Gongga) County, Jêdêxoi (Jiedexiu) town, 3600 m a.s.l., 31 Aug 1975, *anonym 75-1536* (PE00541011, PE00541012, PE00541013).

####### General distribution.

Endemic to South Tibet.

###### 6. Salsola monoptera

Taxon classificationPlantaeCaryophyllalesChenopodiacea

Bunge, Bull. Acad. Imp. Sci. Saint-Pétersbourg 25: 364 (1879)

Salsola monoptera Bunge, Bull. Acad. Imp. Sci. Saint-Pétersbourg 25: 364 (1879). **Lectotype** (Sukhorukov, designated here): Mongolia chinensis in itineris ad Chinam, [year] 1840 [Tatarinow s.n.] (LE!). **Note**. Bunge (1879) did not state a herbarium for the type specimen. Rilke (1999) and Grubov (2000) indicated that the holotype is in LE, but the Bunge herbarium is also deposited in some other herbaria, especially in G and P. Choosing a lectotype, we follow the suggestion of McNeill (2014) since no collection number and herbarium are indicated in the protologue.
≡Kalimonoptera (Bunge) Lomon., Konsp. Fl. Aziatsk. Rossii: 101 (2012).

####### Description.

Annuals, very branched from the base. Stems prostrate or ascending, green, often with reddish stripes, papillate or almost glabrous. Leaves very densely arranged, linear, 10.0–25.0 × 0.4–1.0 mm, leaf mucro ~1.0 mm long. Flowers in axillary clusters and arranged in the main inflorescence. Flower clusters (located below the main inflorescence) consist of one flower supported by a bract and two bracteoles, sometimes additional bracts are present in the clusters; bracts and bracteoles half fused or less; perianth segments membranous, r-shaped with their tips appressed to each other, not hardened, not forming a conus, with pinkish or white, small or hardly noticeable wing-like tubercles in the flexure, one segment (closest to the bract) with small tuberculate (up to 1.0 mm) projection; styles with stigmas ca. 1.0 mm long; fruits 1.0–1.2 mm, depressed roundish to ovoid. Flowers in the main inflorescence with a free bract and two bracteoles (slightly shorter than bract), hermaphrodite; perianth segments free, membranous, r-shaped, horizontally appressed and not forming a conus above the flexure; wing-like appendages vertically located and clearly unequal (the most prominent, entire to lobate < 1.5 mm long, wing develops on the segment closest to the bract (rarely in other positions), other segments have smaller entire tubercles up to 1 mm or no projections at all); the whole diaspore with the perianth wings 1.5–2.3 mm across; anthers 0.3–0.4 mm long; styles with stigmas ~1.0 mm; fruit 1.1–1.4 mm, lenticular. Seeds with horizontally or obliquely orientated embryo.

####### Habitat.

Rocky places and sands at an altitude of 3500–4800 m.

####### Phenology.

Flowering: July-September; fruiting: August-October.

####### Distribution.

See Fig. 48.

####### Specimens examined.

**CHINA**: **Xizang**: **Ngari Prefecture**: Rutog County, 4300 m a.s.l., 3 Sep 1990, *Y. Fei* et al. *702* (KUN0275168); Manasarovar Lake (Mapam Yumtso), Langa Tso (Lake), 30°30'N, 81°17'E, 4680 m a.s.l., 29 Aug 1993, *G. & S. Miehe 9598/11* (herb. Miehe); **Nagqu Prefecture**: Shuanghu County, 4800 m a.s.l., 26 Aug 1976, *Qinghai-Tibet Team Vegetation Group 12348* (PE00515977); Upper Salween basin, Nagqu–Damxung, 21 km S of Nagqu, 31°22'N, 91°56'E, 4570 m a.s.l., 4 Sep 1989, *B. Dickoré 4681* (MSB157875, MSB161896); Central Plateau, Siling Tso (Lake), Nagqu, 31°24'N, 90°1'E, 4700 m a.s.l., 13 Aug 1993, *G. & S. Miehe 9474/07* (MSB147391); S of Xiketang Lake, 31°57'N, 90°46'E, 4680 m a.s.l., 18 Aug 2005, *Nölling & Hanspach NX05-123-04* (herb. Miehe); **Xigazê Prefecture**: [Yadong County] nr Phari, 14500 ft a.s.l., Sep 1938, *B.J. Gould 1625* (K);

**INDIA** (new record for the country): Ladakh, Whisky Nallah, 30 Aug 1970, *U.C. Bhattacharyya 40939* (BSD).

####### General distribution.

Central Asia and South Siberia. The species was probably first collected in Xizang in the 1980s–1990s (Rilke 1999), but it is not mentioned in Zhu et al. (2003). The records cited below expand the distribution of *S.monoptera* to the south (compare to Rilke 1999).

###### 18. Halogeton

Taxon classificationPlantaeCaryophyllalesChenopodiacea

C.A.Mey. in Ledeb., Icon. Pl. 1: 10 (1829)

H. glomeratus (M.Bieb.) C.A.Mey. (**Type**).

####### Description.

Annual up to 30 cm with a tumble-weed habit at the fruiting stage; young plants often woolly due to abundant curved simple hairs in the leaf axils. Stem and leaves glabrous or shortly papillate. Leaves terete, up to 15 mm long, with a yellowish, easily caducous mucro up to 5.0 mm. Flowers covered with a bract and two bracteoles with two types of perianth (most of the flowers with five hyaline perianth segments (up to 3.0 mm long) with an orbicular white or pink wing (2.0–3.0 mm, sometimes 0.7–1.5 mm in diameter) near their tips; a small part of the flowers with 5 perianth segments hardened almost to the top without any projections or tuberculate). Stamens 2–3. Anthers 0.5–0.6 mm long. Fruit 1.0–1.2 mm. Seeds with vertical embryo, with the radicula pointing upwards. Perisperm absent.

One species widely distributed in Central Asia, South Siberia, Tibet and North Himalaya.

###### 1. Halogeton glomeratus

Taxon classificationPlantaeCaryophyllalesChenopodiacea

(M.Bieb.) C.A.Mey. in Ledeb., Fl. Altaic. 1: 378 (1829)

Fig. 50

≡Anabasisglomerata M.Bieb., Mém. Soc. Nat. Mosc. 1: 110 (1808). **Lectotype** (designated here by Sukhorukov): Ex Sibiria [From Siberia], Salesow [Zalesov] (LE!).
=H.tibeticus Bunge, Anabas. Rev.: 94 (1862). **Lectotype** (Sukhorukov, designated here): [probably N INDIA or NW Xizang] In regione temperata regni Tibetani occidentalis, alt. 12[000]–14000’ [ft a.s.l.], *T.T. Thomson* s.n. (K! isolectotypes G00177348! G00177349!).
=H.kashmirianus Grey-Wilson & Wadhwa, Kew Bull. 42(2): 473 (1987). **Holotype**: INDIA, Jammu & Kashmir, Leh, towards Indus river, 3505 m a.s.l., Aug 1976, *Wadhwa 59345* (K, not found; isotype – BSD000000040!).

####### Taxonomic notes.

The differences between *H.glomeratus*, *H.tibeticus* and *H.kashmirianus* are not clearly expressed. The plants growing in the mountainous areas of Pamir northwards through Karakoram to Ladakh are finely papillate-pubescent (*H.tibeticus* s. str.) compared with the populations from the Central Asian plains. A distinct trait indicated in the protologue of *H.tibeticus* (Bunge 1862), the arachoideous stems in young plants, is characteristic for *H.glomeratus* and the stems are glabrescent at maturity. The triangular, erose-dentate perianth wings of *H.tibeticus* compared with *H.glomeratus* (Iljin 1936) rather refer to the wing variability of *H.glomeratus* (as in many *Salsola* s. str.) and such a wing shape is indeed rare in the examined specimens. The same characters are reported as differences between *H.kashmirianus* and *H.glomeratus*, but the name and characters of *H.tibeticus* are not mentioned and discussed in the description of *H.kashmirianus* (Grey-Wilson and Wadhwa 1987). Interestingly, the specimens kept at Kew, especially those collected at different stages and mounted on to one herbarium sheet, were identified differently. The younger, pubescent plants were identified by Grey-Wilson and Wadhwa as *H.kashmirianus*, but the glabrescent plants collected at the fruiting stage are labelled as *H.glomeratus*. The isotype of *H.kashmirianus* (BSD!) represents a part of the plant without papillae on the stem and both the papillate and non-papillate plants are present in Himalaya and Tibet. Due to the absence of the clear characters that allow *H.glomeratus*, *H.kashmirianus* and *H.tibeticus* to be separated, we prefer to treat *H.glomeratus* in a broader sense.

The specimens of *H.glomeratus* from the deserts of adjacent Xinjiang (China) are not morphologically uniform and have much smaller perianth segments and wings (0.7–1.5 mm in diameter [H. kunlunense Sukhor. (nomen)]) than those (1.3–1.6 mm long) of *H.glomeratus*.

####### Description.

See genus description.

**Figure 50.**
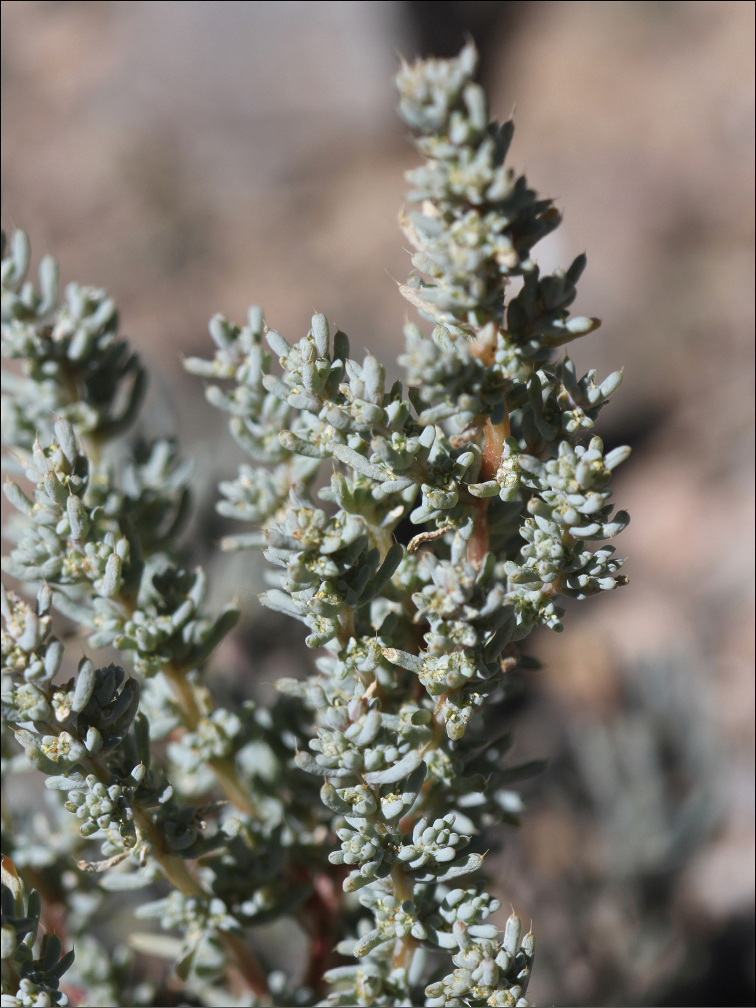
*Halogetonglomeratus* at blooming stage. Photograph by Dr. Oyuntsetseg Batlai (between Khavtag mountain and Baitag mountain range, Altai sum, Khovd province, Mongolia, 10 August 2017).

####### Habitat.

Rocky or sandy, often disturbed places at altitudes of 3200–4500 m. Common in Ladakh.

####### Phenology.

Flowering: July-September; fruiting: August-October.

####### Distribution.

See Fig. 51.

**Figure 51.**
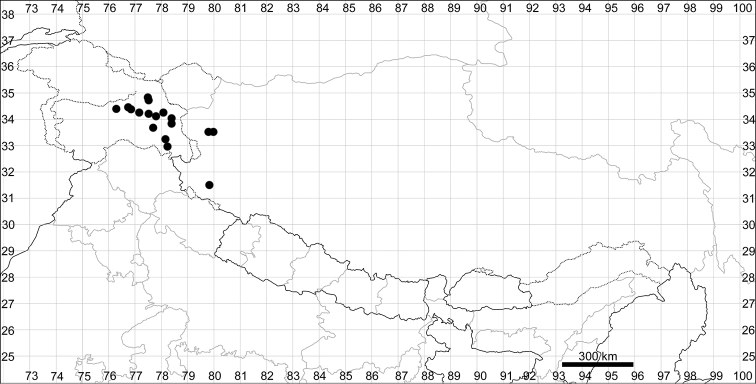
Distribution map of *Halogetonglomeratus*.

####### Specimens examined.

**CHINA: Xizang: Ngari Prefecture**: Rutog (Ritu) County, Lake Bangong (Pangong Tso), 4250 m a.s.l., 3 Sep 1976, *Qinghai-Tibet Team 76-9140, 76-8741* & *76-9142* (PE00540886, PE00540889, PE00540893, KUN0587507, KUN0587509, KUN0587511); Zanda (Zhada) County, Zanda valley, 3600 m a.s.l., 31°29'58"N, 79°45'56"E, 6 Sep 2012, *FLPH Tibet Expedition 12-0260* (PE01967943), *12-0262* (PE01967946), *12-0263* (PE01967925);

**INDIA**: **Jammu & Kashmir** (selected specimens): Nubra valley, 1 Aug 1848, *T.T. Thomson* s.n. (K); Shayuk valley, 15 Sep 1848, *T.T. Thomson* s.n. (K); Ladakh, Leh to Nurla, 1856, *Schlagintweit 1603* (LE, P04558968); Ladakh, 11000 ft a.s.l., Aug 1934, *Kohli* s.n. (K); Ladakh, 10000–16000 ft a.s.l., Sep 1942, *R.R. Stewart* s.n. (K); Ladakh, 11500 ft a.s.l., 24 Aug 1928, *F. Ludlow 500* (BM, E); Ladakh, Indus valley, Sona, 12 Sep 1970, *U.C. Bhattacharyya 41130* (BSD); Leh, Phyang, 3500 m a.s.l., 13 Aug 1975, *M.V. Viswanathan 54606* (BSD); Ladakh, Mulbek, 20 Jul 1976, *B.M. Wadhwa 58823* (BSD); Ladakh, Khaltse, 22 Jul 1976, *B.M. Wadhwa 58916* (BSD); Ladakh, nr Leh, 3380 m a.s.l., 17 Jul 1992, *H. Hartmann 4011* (MSB137933); Ladakh, Nubra valley, Panamik, 3230 m a.s.l., 5 Aug 1995, *H. Hartmann 5034* (G, MSB137935); Ladakh, 15 km W of Pangong Tso [lake], 4200 m a.s.l., 14 Aug 1997, *H. Hartmann 6079* (MSB137931, MSB137932); Ladakh, Indus valley, Stot (E) [Stod river valley], 4180 m a.s.l., 16 Aug 1999, *L. Klimeš 541* (PRA); Ladakh, Rupshu, Tso Moriri, 4690–4740 m a.s.l., 20 Aug 1999, *L. Klimeš 591* (PRA); Ladakh, Indus valley, Zhung (Leh), Gya to Lato, 4060–4070 m a.s.l., 5 Sep 2001, *L. Klimeš 1556* (PRA); Ladakh, Shyok Region, Wari La, 3980–4000 m a.s.l., 15 Sep 2001, *L. Klimeš 1855* (PRA); Ladakh, Leh, 3480 m a.s.l., 2 Aug 2002, *L. Klimeš 2004* (PRA); Ladakh, Indus valley, Domkhar Da, 29 Aug 2002, *L. Klimeš 2440* (PRA); Ladakh, Lukung, 4300 m a.s.l., 9 Sep 2002, *L. Klimeš 2677* (PRA); Ladakh, Pangong Region, 4300 m a.s.l., 10 Sep 2002, *L. Klimeš 2699* (PRA); Ladakh, Indus valley, Sham, 3420–3500 m a.s.l., 24 Sep 2005, *L. Klimeš 6518* (PRA).

####### General distribution.

Central Asia, Siberia, North Himalaya and Tibet; as alien in North America.

###### 19. Haloxylon

Taxon classificationPlantaeCaryophyllalesChenopodiacea

Bunge, Rel. Lehm. (Mém. Sav. Etrang. Petersb.) 7: 468 (1851)

Haloxylon Bunge, Rel. Lehm. (Mém. Sav. Etrang. Petersb.) 7: 468 (1851). **Lectotype** (Pfeiffer 1874): H.ammodendron (C.A.Mey.) Bunge.

####### Description.

Sub-shrublets, shrubs or trees; branches cylindrical, jointed. Leaves opposite, minute or rudimentary, connate. Flowers small, hermaphrodite, axillary and usually solitary, two-bracteolate. Perianth segments 5, free or connate in the lower part with spreading, scarious wings near the tip. Stamens usually 5. Anthers muticous. Staminodes 5, membranous, thin and glabrous, united with the bases of filaments into a lobed, cup-like disc. Ovary with short style and 2–3(5) stigmas. Fruit included in open perianth, globular-depressed, slightly fleshy in the upper part. Seeds horizontal with spirally coiled embryo. Perisperm absent.

Five to seven species mostly distributed in the Irano-Turanian region. The position of *Haloxylonthomsonii* within this genus is still pending due to its unusual life history (sub-shrublet) and one-layered stem epidermis (all other species possess two-layered epidermis).

###### 1. Haloxylon thomsonii

Taxon classificationPlantaeCaryophyllalesChenopodiacea

Bunge in Boiss., Flora Orient. 4(2): 950 (1879)

Fig. 52

Haloxylon thomsonii Bunge in Boiss., Flora Orient. 4(2): 950 (1879). **Type**: n.v.
≡Arthtophytumthomsonii (Bunge) Ulbr. in Engler & Harms, Nat. Pflanzenfam., ed. 2, 16c: 570 (1934).
≡Hammadathomsonii (Bunge) Iljin, Bot. Zhurn. 33: 583 (1948). Hammada cashemiriana Iljin in herb. LE (nomen).

####### Description.

Sub-shrublet up to 30 cm with a robust caudex, branched from the base. Stems glabrous or very shortly papillate, with two furrows. Lower leaves up to 30–35 mm long, fleshy, semi-terete, appressed to the stem or rarely recurved with tufts of hairs in their axils, without mucro or with a hardly noticeable mucro at the top. Bracts usually longer or equal to the flowers. Flowers solitary in the axil of the bract. Perianth segments greenish, 5, at fruiting stage with wings 2.0–2.5 × 2.5–3.5 mm, purple or lilac turning brownish. Anthers 5, ~1 mm long, ovate, apically without an appendage. Style short, 0.25 mm with 2–3 stigmas of the same length. Fruit whitish, depressed orbicular, ~1.5 mm in diameter.

**Figure 52.**
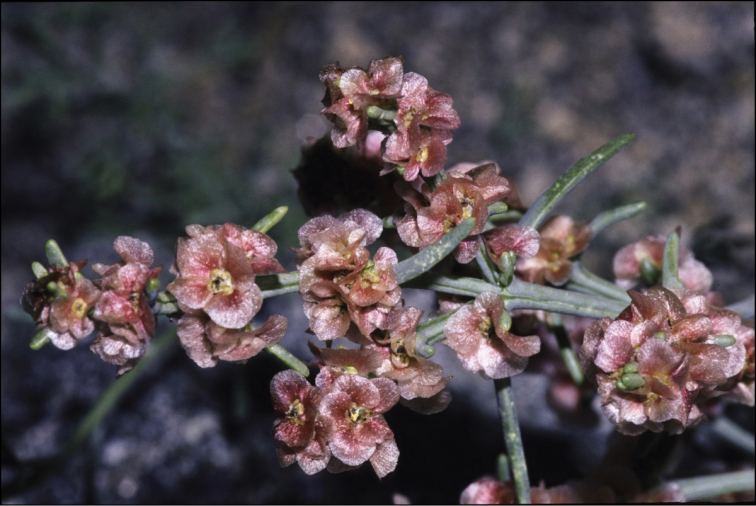
Reproductive shoot of *Haloxylonthomsonii* at fruiting stage, with prominent perianth wings. Photograph by H. Hartmann (Jammu & Kashmir, India, 1995).

####### Habitat.

Rocky areas, semi-deserts and deserts at high elevations (3500–5200 m).

####### Phenology.

Flowering: July-September; fruiting: September-October.

####### Distribution.

See Fig. 53.

**Figure 53.**
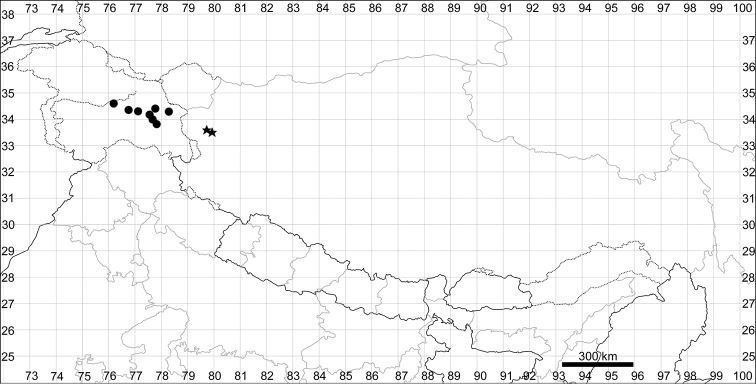
Distribution map of *Haloxylonthomsonii* (circles) and *Sympegmaregelii* (stars).

####### Specimens examined.

**INDIA**: **Jammu & Kashmir** (selected specimens): Shayuk valley, 24 Oct 1847, *T.T. Thomson* s.n. (K); Ladakh, Leh, Sep 1856, *Schlagintweit 1587* (E); Ladakh, Saspul, 10500 ft a.s.l., 27 Aug 1931, *W. Koelz 2706* (DD, E); Ladakh, 12000 ft a.s.l., Aug 1934, *Kohli 4* (K); Ladakh, Lamayuru, 12000 ft a.s.l., 26 Jul 1937, *C.C*. *Burt 52* (E); Leh, Phyang, 3500 m a.s.l., 14 Aug 1975, *M.V. Viswanathan 54640* (BSD); Saspol, between Khalatse & Leh, 3550 m a.s.l., 5 Jul 1976, *Billiet & Leonard 6816* (BR, K); Leh, Upshi, 3600 m a.s.l., 5 Aug 1976, *B.M. Wadhwa 59459* (BSD); Ladakh, Stok [Range], 3656 m a.s.l., 5 Aug 1976, *Maxwell 1747* (E); Ladakh, Leh, 3680 m a.s.l., 16 Jul 1979, *H. Hartmann 2735* (G, MSB137937); Ladakh, Lamyura, 3400 m a.s.l., 26 Aug 1979, *Bruhn* s.n. (M70021); Ladakh, Kargil, 12 Aug 1982, *P.K. Hajra 74055* (BSD); Ladakh, Stok, 3700 m a.s.l., 12 Aug 1992, *H. Hartmann 4017* (G, MSB137939); Ladakh, Matho, 3600 m a.s.l., 1 Jul 1999, *Beck 307* (MSB157837); Ladakh, Leh, 3650 m a.s.l., 13 Aug 1999, *L. Klimeš 440* (PRA); Ladakh, Shyok, Hodtong, 3280–3320 m a.s.l., 26 Sep 2004, *L. Klimeš 5064* (PRA).

####### General distribution.

North India and North Pakistan. Endemic to the Gilgit-Hunza-Indus river basins.

###### 20. Sympegma

Taxon classificationPlantaeCaryophyllalesChenopodiacea

Bunge, Bull. Acad. Imp. Sci. St.-Pétersbourg 25: 371 (1879)

Sympegma Bunge, Bull. Acad. Imp. Sci. St.-Pétersbourg 25: 371 (1879). **Type**: S.regelii Bunge.

####### Description.

Subshrubs with several stems up to 40 cm tall; annual shoots glabrous or with tiny papillae (short simple hairs seen at higher magnification are present in the leaf axils). Leaves alternate, terete, green or glaucous, up to 2 cm long, mostly appressed to the stem, broadened at base, but slightly constricted, straight or incurved above the base with a short persistent mucro. Inflorescences leafy; partial inflorescences pedicellate with opposite bracts enclosing (1–2)3–6 flowers. Perianth glabrous or papillate; perianth segments (4)5, 2.5–3.0 mm long, fused only basally, clearly three-nerved, margins glabrous or tiny papillate, with wing-like projections originating from the upper half of the segments at fruiting stage; wings yellowish, white or pink turning brown in mature fruits. Stamens 5, anthers ~1.5 mm long, with hardly noticeable appendage. Style very broad terminating with two stigmas. Seed with vertically orientated, spiral embryo. Perisperm absent.

Genus of 1–2 species. *Sympegmaelegans* G.L.Chu (Zhu [Chu] and Sanderson 2017) described from Gansu Province (China) was not investigated by us. It reportedly differs from *S.regelii* by slender branches, shorter internodes and mostly solitary flowers (Zhu and Sanderson 2017).

###### 1. Sympegma regelii

Taxon classificationPlantaeCaryophyllalesChenopodiacea

Bunge, Bull. Acad. Imp. Sci. Saint-Pétersbourg 25: 371 (1879)

####### Lectotype

(Grubov 1966): [CHINA, Xinjiang, Qitai County] basin of Bar-kul Lake towards Gucheng, 5 Sep 1875, *P.A. Pyasetsky* s.n. (LE).

####### Description.

See genus description.

####### Habitat.

Screes; 3500–4500 m a.s.l.

####### Phenology.

Flowering: July-September; fruiting: August-October.

####### Distribution.

See Fig. 53.

####### Specimens examined.

**CHINA: Xizang** (new records): **Ngari Prefecture**: Rutog (Ritu) County, nr Bangong Lake, 4230 m a.s.l., 10 Jul 1974, *Biological Institute Tibet Expedition Team 3461* (HNWP40809) & 4 Sep 1984, *Qinghai-Tibet Team 1340* (HNWP146054); East bank of Bangong Lake, 4200 m a.s.l., 15 Aug 1987, *B.S. Li & D. Zheng 11018* (PE01341763).

This is the first record of *S.regelii* in Xizang, and the records in the neighbouring areas of Jammu & Kashmir (India) are predicted. *Sympegma* is a common plant on the screes in North Pakistan and Xinjiang (Grubov 1966, Eberhardt et al. 2007, Hong and Blackmore 2015).

####### General distribution.

W China, E Kazakhstan, Kyrgyzstan, Mongolia and Tajikistan.

##### Species of other genera excluded

###### Halocharis violacea

Taxon classificationPlantaeCaryophyllalesChenopodiacea

Bunge, Anabas. Revis. 63 (1862) [Mém. Acad. Imp. Sci. St.-Pétersbourg, Sér. 7, 4(11)]

####### Notes.

This species has been reported for Ladakh, India (Stewart 1916). No specimens were seen from our territory and the nearest records are from Quetta, Pakistan (Hedge 1997). This plant can be confused with *Halogetonglomeratus* (re-identifications in BSD) and both species are woolly at younger stages. Both genera can be easily distinguished by the following leaf characteristics: the leaves of all *Halocharis* (tribe Caroxyleae) are covered with both short and long simple hairs, while those of *Halogeton* (tribe Salsoleae) are glabrous or covered with short papillae (long, clearly visible hairs are present only at the leaf base). The presence of two hair types is a distinct characteristic of *Halocharis* compared with many other Salsoloideae (Sukhorukov et al. 2016b).

## Supplementary Material

XML Treatment for Chenopodium

XML Treatment for Chenopodium album

XML Treatment for Chenopodium pamiricum

XML Treatment for Chenopodium ficifolium

XML Treatment for Chenopodium harae

XML Treatment for Chenopodium karoi

XML Treatment for Chenopodium bengalense

XML Treatment for Chenopodium novopokrovskyanum

XML Treatment for Chenopodium atripliciforme

XML Treatment for Chenopodium perttii

XML Treatment for Chenopodium strictum

XML Treatment for Chenopodium hookerianum

XML Treatment for Chenopodium patulum

XML Treatment for Chenopodiastrum

XML Treatment for Chenopodiastrum murale

XML Treatment for Chenopodiastrum badachschanicum

XML Treatment for Oxybasis

XML Treatment for Oxybasis glauca

XML Treatment for Atriplex

XML Treatment for Atriplex centralasiatica

XML Treatment for Atriplex pallida

XML Treatment for Atriplex pamirica

XML Treatment for Atriplex hortensis

XML Treatment for Microgynoecium

XML Treatment for Microgynoecium tibeticum

XML Treatment for Blitum

XML Treatment for Blitum virgatum

XML Treatment for Dysphania

XML Treatment for Dysphania ambrosioides

XML Treatment for Dysphania himalaica

XML Treatment for Dysphania bhutanica

XML Treatment for Dysphania kitiae

XML Treatment for Dysphania botrys

XML Treatment for Dysphania geoffreyi

XML Treatment for Dysphania tibetica

XML Treatment for Dysphania neglecta

XML Treatment for Dysphania nepalensis

XML Treatment for Dysphania schraderiana

XML Treatment for Teloxys

XML Treatment for Teloxys aristata

XML Treatment for Axyris

XML Treatment for Axyris prostrata

XML Treatment for Axyris mira

XML Treatment for Axyris sphaerosperma

XML Treatment for Axyris amaranthoides

XML Treatment for Axyris hybrida

XML Treatment for Axyris villosa

XML Treatment for Krascheninnikovia

XML Treatment for Krascheninnikovia ceratoides

XML Treatment for Acroglochin

XML Treatment for Acroglochin persicarioides

XML Treatment for Corispermum

XML Treatment for Corispermum falcatum

XML Treatment for Corispermum nanum

XML Treatment for Corispermum pamiricum

XML Treatment for Corispermum dutreuilii

XML Treatment for Corispermum dutreuilii var. montanum

XML Treatment for Corispermum dutreuilii

XML Treatment for Corispermum pseudofalcatum

XML Treatment for Corispermum lhasaense

XML Treatment for Corispermum lepidocarpum

XML Treatment for Corispermum gelidum

XML Treatment for Corispermum tibeticum

XML Treatment for Corispermum korovinii

XML Treatment for Agriophyllum

XML Treatment for Agriophyllum tibeticum

XML Treatment for Agriophyllum pungens

XML Treatment for Suaeda

XML Treatment for Suaeda olufsenii

XML Treatment for Suaeda microsperma

XML Treatment for Bassia

XML Treatment for Bassia scoparia

XML Treatment for Bassia odontoptera

XML Treatment for Bassia prostrata

XML Treatment for Bassia indica

XML Treatment for Grubovia

XML Treatment for Grubovia dasyphylla

XML Treatment for Salsola

XML Treatment for Salsola hartmannii

XML Treatment for Salsola collina

XML Treatment for Salsola jacquemontii

XML Treatment for Salsola tragus

XML Treatment for Salsola austrotibetica

XML Treatment for Salsola monoptera

XML Treatment for Halogeton

XML Treatment for Halogeton glomeratus

XML Treatment for Haloxylon

XML Treatment for Haloxylon thomsonii

XML Treatment for Sympegma

XML Treatment for Sympegma regelii

XML Treatment for Halocharis violacea
